# Supplementary material for: A COVID-19 peptide vaccine for the induction of SARS-CoV-2 T cell immunity
Source: Nature. 2021 Nov 23;601(7894):617–22. doi: 10.1038/s41586-021-04232-5 (PMC8791831; doi:10.1038/s41586-021-04232-5)
Supplement: Supplementary file 1 — The Supplementary Information contains: Supplementary Methods, Supplementary Study Results, Supplementary Tables 1–8 and Supplementary Fig. 1. [file 41586_2021_4232_MOESM1_ESM.pdf]

nature portfolio

https://doi.org/10.1038/s41586-021-04232-5

Supplementary information

A COVID-19 peptide vaccine for the induction of SARS-CoV-2 T cell immunity

In the format provided by the authors and unedited

Supplementary Appendix to Manuscript Entitled

A COVID-19 Peptide Vaccine for the Induction of SARS-CoV-2 T-Cell Immunity

Table of Contents

Supplementary Methods 2

Detailed inclusion and exclusion criteria for trial participants 2
Stopping criteria and statistical considerations 2
SARS-CoV-2 convalescent individuals and healthy vaccinated volunteers 4
IFN-γ ELISPOT assay *ex vivo* or following 12-day *in vitro* expansion 4
Intracellular cytokine, cell surface marker, and tetramer staining 6
Antibody testing 7
Software and statistical analysis 7
Dose rationale for vaccine peptides 7
Dose rationale for XS15 8
Dose rationale for Montanide™ ISA51 VG 9

Supplementary Study Results 10

Safety assessment of sentinel dosing 10
Safety laboratory assessment 10
Unsolicited adverse events 10

Supplementary Tables 11

Table S1: CoVac-1 peptides and corresponding embedded HLA class I-restricted peptides 11
Table S2: Characteristics of convalescents after SARS-CoV-2 infection 12
Table S3: Characteristics of healthy volunteers after mRNA-, vector-based, or heterologous vaccination. 13
Table S4: Mutations of SARS-CoV-2 variants of concern 14
Table S5: Toxicity grading scale modified according to CTCAE V5.0 16
Table S6: Potential immune mediated conditions 17
Table S7: Adverse events of special interest (AESI) 18
Table S8: CoVac-1-induced $CD8^+$ T-cell responses *ex vivo* 19

Supplementary Figures 20

Figure S1: Gating strategy for flow cytometry-based evaluation of surface marker and intracellular cytokine staining 20

References 21

Supplementary Methods

Detailed inclusion and exclusion criteria for trial participants

Eligible participants were men or women aged 18-55 (Part I) or 56-80 years (Part II). In Part I, participants were free of clinically significant health problems. In Part II, participants with stable medical history were enrolled. Participants had to refrain from blood donations during the course of the study and be willing to minimize body fluid transmission to others for 7 days after vaccination. All participants had to adhere to adequate contraception methods until three months after vaccination.

Exclusion criteria comprised: pregnant or lactating females, participation in another clinical trial, treatment with immunosuppressive drugs, prior or current infection with SARS-CoV-2 (proven serologically or by PCR), known previous anaphylactic reaction to any component or hypersensitivity to any component of the CoVac-1 vaccine, relevant CNS pathology or other neurological disease, positivity for HIV or active hepatitis, lymphocyte count ≤ 1.000/µL, blood donation within 30 days, or administration of immunoglobulins or blood products within 120 days prior to study inclusion, diabetes type II, chronic lung disease requiring drug treatment, increased liver enzymes (≥ 2.5x upper limit of normal), renal failure (GFR < 60 mL/min/1.73 m^2), serious cardiovascular disease, sickle cell anemia, obesity (defined by age adjusted body mass index), or preexisting auto-immune disease except for Hashimoto thyroiditis and mild psoriasis.

Stopping criteria and statistical considerations

This clinical trial had a predefined stopping rule: Observation of one serious adverse event (SAE) associated with administration of CoVac-1. The sample size calculation of 36 participants for the trial was based on this stopping rule and determined to show that the incidence of SAE associated with administration of CoVac-1 does not exceed a predetermined

rate of 5% (= P1). Safety of the CoVac-1 vaccine should be shown if no SAE (= P0) occurs in the study population. An evaluable sample size of 33 achieves 81.6% of power to detect a difference (P1 – P0) of 0.0499 using a one-sided exact test based on the binomial distribution with a target significance level of 0.05 (sample size determination using PASS). These results assume that the population proportion under the null hypothesis (P0) is ≤ 0.0001. Assuming a drop-out rate of 7.5% (percentage of subjects that are expected to be lost at random during the course of the study and for whom no response data concerning existence of SAE will be collected, *i.e.* will be treated as "missing") the total number of 36 subjects should be enrolled in the study to achieve the threshold of 33 evaluable subjects.

Besides the predefined stopping rule, further holding rules were implemented:

- Local solicited adverse events (AEs): If more than 30% of injections are followed by grade ≥ 3 solicited swelling or pain or grade 4 erythema (first occurrence at any time after vaccination) and persisting at grade 3 (swelling or pain)/4 (erythema) for > 48 h to maximum 72 hours depending upon symptom severity and kinetics.
- Systemic solicited AEs: If more than 25% of injections are followed by grade 3 solicited systemic AE beginning within 3 days after injection (day of injection and 2 subsequent days) and persisting at grade ≥ 3 for > 48 h to maximum 72 hours depending upon symptom severity and kinetics.
- Unsolicited AEs: If more than 25% of volunteers develop a grade ≥ 3 unsolicited AE (including laboratory AE and physical observations) that is considered probably or definitely related to injection and persists at grade 3 for > 48 to maximum 72 hours depending upon symptom severity and kinetics.
- A suspected unexpected serious adverse drug reaction (SUSAR) occurs that is life-threatening or results in death.

If a holding rule is met, further recruitment is paused until review by the data safety monitoring board (DSMB). In addition, the DSMB reviewed the safety and immunogenicity data of the Part I of the clinical trial prior to proceeding to Part II.

SARS-CoV-2 convalescent individuals and healthy vaccinated volunteers

To delineate differences of SARS-CoV-2 immune responses in CoVac-1 vaccinated participants to immune responses after natural infection, a reference group of non-hospitalized COVID-19 convalescent individuals, described previously[1,2], was used for comparison. SARS-CoV-2 infection was confirmed by real-time polymerase chain reaction (PCR) after nasopharyngeal swab. Sample collection for human COVID-19 convalescents (HCs, n = 63) was performed 16-52 days after positive PCR. In addition, CoVac-1-induced T-cell responses were compared to spike-specific T-cell responses induced by approved mRNA- and adenoviral vector-based vaccines, as well as by heterologous vaccination (vector- followed by mRNA-based vaccination). Sample collection was performed 18-42 days after the second vaccination. Donor characteristics comprising COVID-19 symptoms for HCs were assessed by questionnaire. Details are provided in Supplementary Tables S2 and S3. Written informed consent was obtained in accordance with the Declaration of Helsinki protocol (local Ethics Committee at University Hospital Tübingen and the Landesärztekammer Hessen; project numbers: 179/2020/BO2 and 2021-2305-evBO).

Peripheral blood mononuclear cells (PBMCs) were isolated by density gradient centrifugation and were stored at -80°C.

IFN-γ ELISPOT assay *ex vivo* or following 12-day *in vitro* expansion

For *in vitro* expansion, PBMCs were pulsed with CoVac-1 peptides[1,2] (5 µg/mL per peptide) and cultured for 12 days adding 20 U/mL interleukin-2 (IL-2, Novartis) on days 3, 5, and 7. Peptide-stimulated (*in vitro* expanded) or freshly thawed (*ex vivo*) PBMCs were analyzed by

interferon (IFN)-γ enzyme-linked immunospot (ELISPOT) assay, as described previously[1]. In brief, 2-6 x 10^5 cells per well (technical replicates) were incubated in 96-well ELISPOT plates coated with anti-IFN-γ antibody (clone 1-D1K, 2 µg/mL, MabTech, Cat# 3420-3-250, RRID: AB_907283) with 1 µg/mL or 2.5 µg/mL for embedded human leukocyte antigen (HLA) class I or HLA-DR-restricted CoVac-1 peptides and overlapping 15-mer peptide pools covering the entire spike protein (Milteny PepTivator® SARS-CoV-2 Prot_S, PepTivator® SARS-CoV-2 Prot_S+, PepTivator® SARS-CoV-2 Prot_S1), respectively. PHA (Sigma-Aldrich) served as positive control. An irrelevant HLA-DR-restricted control peptide (ETVITVDTKAAGKGK, $FLNA\_HUMAN_{1669-1683}$) or 10% dimethyl sulfoxide (DMSO) in double-distilled water served as negative controls. After 24 h of incubation, spots were revealed with anti-IFN-γ biotinylated detection antibody (clone 7-B6-1, 0.3 µg/mL, MabTech, Cat# 3420-6-250, RRID: AB_907273), ExtrAvidin-Alkaline Phosphatase (1:1,000 dilution, Sigma-Aldrich), and BCIP/NBT (5-bromo-4-chloro-3-indolyl-phosphate/nitro-blue tetrazolium chloride, Sigma-Aldrich). Spots were counted using an ImmunoSpot S6 analyzer (CTL). T-cell responses were considered positive if the mean spot count of the technical replicates was ≥ 3-fold higher than the mean spot count of the negative control and defined as CoVac-1-induced if the mean spot count post vaccination was ≥ 2-fold higher than the respective spot count on day 1. The intensity of T-cell responses is depicted as calculated spot counts, which represent the mean spot count normalized to 5 x 10^5 cells minus the normalized mean spot count of the respective negative control.

Intracellular cytokine, cell surface marker, and tetramer staining

Peptide-specific T cells were characterized by cell surface marker and intracellular cytokine staining (ICS) as previously described[1]. In brief, PBMCs were incubated with the CoVac-1 vaccine peptide pool or the negative control peptide (10 µg/mL per peptide), Brefeldin A (Sigma-Aldrich), and GolgiStop (BD Biosciences). PMA and ionomycin (Sigma-Aldrich) served as positive control. Staining was performed using Cytofix/Cytoperm solution (BD), Aqua live/dead (1:400 dilution, Invitrogen), APC/Cy7 anti-human CD4 (1:100 dilution, BioLegend, Cat# 300518, RRID: AB_314086), PE/Cy7 anti-human CD8 (1:400 dilution, Beckman Coulter, Cat# 737661, RRID: AB_1575980), Pacific Blue anti-human tumor necrosis factor (TNF, 1:120 dilution, BioLegend, Cat# 502920, RRID: AB_528965), FITC anti-human CD107a (1:100 dilution, BioLegend, Cat# 328606, RRID: AB_1186036), APC anti-human IL-2 (1:40 dilution, BioLegend, Cat# 500309, RRID: AB_315096), and PE anti-human IFN-γ monoclonal antibodies (1:200 dilution, BioLegend, Cat# 506507, RRID: AB_315440). T-cell responses were considered positive if the detected frequency of cytokine-positive $CD4^+$ or $CD8^+$ T cells was ≥ 3-fold higher than the frequency in the negative control. Frequency of cytokine-positive cells was corrected for background by subtraction of the respective negative control values. Negative values were set to zero. Results were defined as induced response if the frequency of cytokine-positive cells was ≥ 2-fold higher than the respective frequency on day 1. The frequency of CoVac-1-embedded peptide-specific $CD8^+$ T cells was determined by PE/Cy7 anti-human CD8 and HLA:peptide tetramer-PE (5 µg/mL) staining. HLA-matched control cells stained with the tetramer of interest were used to determine the background. For evaluation we applied the same criteria used for ICS. All samples were analyzed on a FACS Canto II cytometer (BD). The gating strategy applied for the analyses of flow cytometry-acquired data is provided in Supplementary Fig. S1.

Antibody testing

The Siemens SARS-CoV-2 Total (COV2T) and Siemens SARS-CoV-2 IgG (SCOVG) assays were performed on an automated ADVIA Centaur XPT system (Siemens Healthineers) according to the manufacturer's instructions. The immunoassays detect anti-SARS-CoV-2 total antibodies (IgG and IgM; COV2T) and IgG antibodies (SCOVG) directed against the S1 domain of the viral spike protein (including the immunologically relevant receptor binding domain). Results of one measurement of each serum sample are reported in Index Values. The final interpretation of positivity is determined by an Index Value ≥ 1.0 given by the manufacturer. Values < 0.1 were set to zero. Quality control was performed following the manufacturer's instructions on each day of testing.

Software and statistical analysis

Flow cytometric data was analyzed using FlowJo 10.7.1 (BD). Graphs were plotted using Inkscape 1.1 and GraphPad Prism 9.2.0. Statistical analyses were conducted using GraphPad Prism 9.2.0 and SAS Version 9.4.

In case an AE, even if determined by definition as solicited, was clearly caused by another reason, the investigator judged the event as unrelated to CoVac-1. These unrelated AEs are displayed as unsolicited AEs (Extended Data Table 2).

Dose rationale for vaccine peptides

Previous vaccination trials were performed at peptide doses ranging from 10 to 5,000 µg per peptide per vaccination. Even though only a few of these trials included a dose finding element, there is a tendency that doses below 100 µg are not effective to induce T-cell responses whilst doses above 500 µg do not seem to generate an increasing immunogenicity. Dose-finding studies performed with viral protein-derived epitopes showed significantly

stronger immune responses in the 250-500 µg range versus the 100 µg dose, without significantly higher immune responses in the 1,000 µg vs. 500 µg group[47]. Concerning safety of peptide vaccines in different doses, no severe side effects were observed even with very high doses of peptides up to 30 mg[48,49]. This is supported by own data of the investigator from various completed and ongoing trials (NCT02802943, NCT04688385, NCT0214922, NCT01265901)[44,46]. Furthermore, a multi-peptide vaccination study for influenza evaluated safety and immunogenicity with two doses of peptides (250 µg and 500 µg). No differences in the safety profile but a significant induction of functional T-cell responses was observed for both peptide dosages, suggesting 250 µg of peptide to be sufficient and safe for a prophylactic viral peptide vaccine[50]. This is further supported by first clinical data from a healthy volunteer vaccinated with viral peptide vaccines (240-300 µg per peptide) including two of the CoVac-1 peptides (250 µg) in combination with XS15 showing potent induction of peptide-specific T-cell responses and a good safety profile[8,10]. Thus, the dose of 250 µg per peptide for the CoVac-1 vaccine was selected.

Dose rationale for XS15

The molecular mode of action of both the Pam_3Cys conjugates and XS15 is an activation of immune cells via the TLR1/2. These immune cells are mainly found in the blood and lymphoid tissues. Due to the XS15 and TLR1/2 interaction, desired as well as toxic effects are expected exclusively from these cells, in particular through an overactivation, which could then lead to a cytokine release syndrome. The dosage of XS15 is based on an *in vitro* assay that investigated both potential toxicity as well as efficiency. In this assay 10 µg/mL XS15 was shown to be most efficient for the stimulation of immune cells. The local formation of a granuloma (size up to 8 mL on day 17 after vaccination)[10] locally after subcutaneous (s.c.) injection of XS15 emulsified in Montanide™ ISA51 VG, in a total volume of 500 µL

suspension, leads to a size-dependent decrease of XS15 concentration. A dose of 50 µg XS15 was selected and achieved the desired concentration of 10 µg/mL at the vaccination site.

In a subsequent toxicity study in mice, a dose of 50 µg XS15 in Montanide™, applied locally s.c. did not reveal any local or systemic toxicity beyond the long known and expected local toxicity of Montanide™ alone. Furthermore, regarding systemic toxicity after s.c. injection of this XS15 dose the following considerations were made: in the absence of Montanide™ ISA51 VG and immediate distribution in the blood (6 L), a maximum blood concentration of 0.008 µg/mL would be expected. At this concentration, no measurable stimulation of immune cells is detected in the above-described *in vitro* test. When used with Montanide™, the formation of a granuloma at the injection site, which has a depot effect for peptides, a gradual release of these peptides or XS15 into the blood can be expected. Therefore, the actual blood concentration of XS15 after administration of 50 µg in a Montanide™/water emulsion is likely to be much lower than the maximum concentration of 0.008 µg/mL described above. Hence, a systemic toxic effect of XS15 is not expected at a dosage of 50 µg s.c. with or without Montanide™, which was proven in first clinical vaccination experiments in a healthy volunteer[8,10].

Dose rationale for Montanide™ ISA51 VG

Montanide™ ISA51 VG has been used in about 300 clinical trials from Phase I to Phase III, which represent >19,000 vaccine doses. Dosing of 0.5 mL after 50/50 mixture with peptides in water is based on two published clinical studies evaluating influenza vaccines in > 2,500 donors showing high immunogenicity and a good safety profile[27,52,53].

Supplementary Study Results

Safety assessment of sentinel dosing

Sentinel dosing took place at the end of November 2020. The safety assessment was performed on day 28 after vaccination. Until day 28 no SAE was reported. The participant developed erythema (grade 1), granuloma (grade 1), swelling (grade 1), itching (grade 1), and vaccination site lymphadenopathy (grade 1). Based on these observed AEs, the sponsor decided to continue recruiting.

Safety laboratory assessment

Safety laboratory AEs were graded according to a CTCAE V5.0 grading scale. Abnormal laboratory parameters were assessed by the investigators for clinical significance. No clinically significant laboratory deviation was reported. If normal at baseline, deviations from normal range were reported for six parameters (hemoglobulin, creatinine, alanine transaminase, aspartate transaminase, fibrinogen, and C-reactive protein). The most frequent deviation was reported for C-reactive protein in 11 participants (31%). 3 participants were reported to have grade 1 elevated liver enzymes. No laboratory abnormality met grade 2 or higher toxicity.

Unsolicited adverse events

There were 58 unsolicited AEs reported, none met the definition of a SAE (Extended Data Table 2). Of those, 81% were mild in severity, one of which was ruled as related to vaccination (herpes simplex reactivation in one subject of Part II), and 17% were moderate, one of which was ruled as related to vaccination (shingles in one subject of Part II). Only one (2%) severe unsolicited AE occurred (hypertension). In total, 3% AEs were judged as related to vaccination.

Supplementary Tables

Supplementary Table S1: CoVac-1 peptides and corresponding embedded HLA class I-restricted peptides.

Peptide ID	Sequence	Protein	Protein class	HLA restriction	Position	Embedded peptide ID	Embedded peptide sequence	HLA restriction of embedded peptides
P1_nuc	ASWFTALTQHGKEDL	ORF9 nuc	structural	DRB1*04, DRB1*11	50-64	P1_A*03	FTALTQHGK	A*03, A*11
P2_nuc	LLLLDRLNQLESKMS	ORF9 nuc	structural	DRB1*04, DRB1*15	221-235	P2_A*02a	LLLLDRLNQL	A*02
						P2_A*02b	LLLDRLNQL	A*02
P3_spi	ITRFQTLLALHRSYL	ORF2 spi	structural	DRB1*01	235-249	P3_C*07	TRFQTLLAL	C*07
						P3_B*15	TLLALHRSY	B*15
						P3_A*02	LLALHRSYL	A*02
P4_env	FYVYSRVKNLNSSRV	ORF4 env	structural	DRB1*04, DRB1*11	56-70	P4_A*24	FYVYSRVKNL	A*24
						P4_A*03	RVKNLNSSR	A*03
P5_mem	LSYYKLGASQRVAGD	ORF5 mem	structural	DRB1*04, DRB1*07	176-190	-	-	-
P6_ORF8	SKWYIRVGARKSAPL	ORF8	accessory	DRB1*01, DRB1*11	43-57	-	-	-

Abbreviations: ID, identification; nuc, nucleocapsid; spi, spike; env, envelope; mem, membrane; ORF, open reading frame.

Supplementary Table S2: Characteristics of convalescents after SARS-CoV-2 infection.

Characteristics	ELISPOT			Peptide titration	ICS	
	CoVac-1	cross-reactive EC	SARS-CoV-2-specific EC		CoVac-1 (*ex vivo*)	CoVac-1 (*ex vivo* / IVE)
Samples – n	24	27	26	5	19	9
Age range – n (%)						
21 - 40 years	9 (37.5)	10 (37)	8 (31)	1 (20)	8 (42)	2 (22)
41 - 55 years	10 (41.7)	8 (30)	8 (31)	2 (40)	8 (42)	2 (22)
56 - 79 years	5 (20.8)	9 (33)	10 (39)	2 (40)	3 (16)	5 (56)
Female – n (%)	12 (50)	17 (63)	15 (58)	3 (60)	9 (47)	5 (56)
Days PCR to sample collection – median (range)	41 (16 - 48)	44 (34 - 52)	45 (34 - 52)	44 (39 - 48)	42 (16 - 48)	36 (29 - 42)
COVID-19 severity* – n (%)						
Asymptomatic	11 (46)	9 (33)	7 (27)	1 (20)	9 (47)	5 (56)
Symptomatic outpatient	13 (54)	18 (67)	19 (73)	4 (80)	10 (53)	4 (44)

Abbreviations: PCR, polymerase chain reaction; EC, epitope composition; ICS, intracellular cytokine staining; IVE, *in vitro* expansion; *COVID-19 severity categories 1) Asymptomatic: samples collected from individuals without symptomatic disease; 2) Symptomatic outpatient: sample collected from individuals reporting fever (≥ 38.0°C) and subjective disease symptoms. n, number.

Supplementary Table S3: Characteristics of healthy volunteers after mRNA-, vector-based, or heterologous vaccination.

	**mRNA-based vaccine** (BNT162b2/mRNA-1273)	**Vector-based vaccine** (AZD1222)	**Heterologous vaccination** (AZD1222 + mRNA-1273)
Samples – n	20	5	5
Age range – n (%)			
21 - 40 years	8 (40)	1 (20)	2 (40)
41 - 55 years	8 (40)	1 (20)	1 (20)
56 - 72 years	4 (20)	3 (60)	2 (40)
Female – n (%)	11 (55)	4 (80)	2 (40)
Days from 2nd vaccination to sample collection – median (range)	20 (19 - 42)	23 (18 - 27)	21 (21 - 22)

The messenger ribonucleic acid (mRNA)-based vaccine group includes samples from donors vaccinated twice either with mRNA-1273 (n = 3) or with BNT162b2 (n = 17). Donors from the adenoviral vector-based vaccine group received two doses of AZD1222. Donors of the heterologous vaccination regime group received one dose of AZD1222 followed by one dose of mRNA-1273. n, number.

Supplementary Table S4: Mutations of SARS-CoV-2 variants of concern.

Protein	Position	Mutation	B.1.1.7-Alpha	B.1.351-Beta	P.1-Gamma	B.1.617.2-Delta
ORF1	265	T265I		x		
	1,001	T1001I	x			
	1,188	S1188L			x	
	1,567	T1567I				x
	1,655	K1655N		x		
	1,708	A1708D	x			
	1,795	K1795Q			x	
	2,230	I2230T	x			
	2,799	H2799Y		x		
	2,900	S2900L		x		
	3,353	K3353R		x		
	3,646	T3646A				x
	3,655	M3655I		x		
	3,675	SGF3675-3677del	x		x	
	4,715	P4715L		x		x
	5,530	G5530C				x
	5,753	M5753I				x
	5,912	T5912I		x		
	6,711	K6711R				x
	6,713	S6713A				x
ORF2 spi	18	L18F		x	x	
	19	T19R				x
	20	T20N			x	
	26	P26S			x	
	69	HV69-70del	x			
	80	D80A		x		
	95	T95I				x
	138	D138Y			x	
	142	G142D				x
	144	Y144del	x			
	154	E154K				x
	157	FR157-158del				x
	190	R190S			x	
	215	D215G		x		
	**242**	**L242H**		**x**		
	**246**	**R246I**		**x**		
	417	K417T			x	
	417	K417N		x		
	478	T478K				x
	452	L452R				x
	484	E484Q				x
	484	E484K		x	x	
	501	N501Y	x	x	x	
	570	A570D	x			
	614	D614G		x		x
	655	H655Y			x	
	681	P681H	x			
	681	P681R				x
	701	A701V		x		
	716	T716I	x			
	950	D950N				x
	982	S982A	x			
	1,027	T1027I			x	
	1,071	Q1071H				x
	1,118	D1118H	x			
ORF3	26	S26L				x
	57	Q57H		x		
	171	S171L		x		
ORF4 env	71	P71L		x		
ORF5 mem	82	I82S				x
ORF7a	43	N43Y				x
	82	V82A				x
	120	T120I				x
ORF8	27	Q27*	x			
	**52**	**R52I**	**x**			
	73	Y73C	x			
	92	E92K			x	
	121	I121L		x		
ORF9 nuc	3	D3L	x			
	80	P80R			x	
	203	R203M				x
	205	T205I		x		
	**235**	**S235F**	**x**			
	377	D377Y				x
ORF9c	52	L52F		x		

List of variant-defining and -associated mutations included in the four SARS-CoV-2 variants of concern (B.1.1.7-Alpha, B.1.351-Beta, P.1-Gamma, B.1.617.2-Delta including mutations of B.1.617). Mutations marked in bold affect CoVac-1 peptides. Abbreviations: nuc, nucleocapsid; spi, spike; env, envelope; mem, membrane; no, number; ORF, open reading frame.

Supplementary Table S5: Toxicity grading scale modified according to CTCAE V5.0.

Local solicited AEs	CTCAE Term	Grade 0 (normal)	Grade 1 (mild)	Grade 2 (moderate)	Grade 3 (severe)	Grade 4 (life-threatening)
**Erythema**	Injection site reaction	< 25 mm	25 - 50 mm	51 - 100 mm	> 100 mm	Life-threatening consequences: urgent intervention indicated
**Swelling**	Injection site reaction	< 25 mm	25 - 50 mm	51 - 100 mm	> 100 mm	Necrosis
**Pain**	Injection site reaction	None	Tenderness with or without associated symptoms	Interferes with activity	Prevents daily activity	Life-threatening consequences: urgent intervention indicated emergency room visit or hospitalization
**Induration/ granuloma**	Injection site reaction	None	25 - 50 mm	51 - 100 mm	> 100 mm	Life-threatening consequences: urgent intervention indicated
**Ulceration**	Injection site reaction	None	Combined area of ulcers < 1 cm	Combined area of ulcers 1 - 2 cm	Combined area of ulcers > 2 cm	Necrosis

Abbreviations: AE, adverse event; CTCAE, Common Terminology Criteria for Adverse Events.

Supplementary Table S6: Potential immune mediated conditions[54].

Neuroinflammatory disorders	Musculoskeletal disorders	Skin disorders	Liver disorder	Gastrointestinal disorders	Metabolic & endocrine disorders	Vasculitides	Others
Cranial nerve inflammatory disorders, including paralyses/paresis (e.g., Bell's palsy)	Systemic lupus erythematosus	Psoriasis	Autoimmune hepatitis	Crohn's disease	Autoimmune thyroiditis including Hashimoto thyroiditis	Large vessels vasculitis, including giant cell arteritis such as Takayasu's arteritis and temporal arteritis	Autoimmune haemolytic anaemia
Acute disseminated encephalomyelitis, including site-specific variants: encephalitis, encephalomyelitis, myelitis, myeloradiculoneuritis, cerebellitis	Systemic sclerosis (with limited or diffuse cutaneous involvement)	Vitiligo	Primary biliary cirrhosis	Ulcerative colitis	Grave's or Basedow's disease		Autoimmune thrombocytopenia
Multiple sclerosis	Dermatomyositis	Erythema nodosum	Primary sclerosing cholangitis	Ulcerative proctitis	Diabetes mellitus type I		Antiphospholipid syndrome
Transverse myelitis	Polymyositis		Autoimmune cholangitis	Celiac disease	Addison's disease		Pernicious anaemia
Optic neuritis	Anti-synthetase syndrome	Cutaneous lupus erythematosus					Raynaud's phenomenon
Narcolepsy	Rheumatoid arthritis	Alopecia areata					Uveitis
	Juvenile chronic arthritis including Still's disease	Lichen planus					Autoimmune myocarditis/cardiomyopathy
	Polymyalgia rheumatica	Sweet's syndrome					Sarcoidosis
	Psoriatic arthropathy	Morphoea					Stevens-Johnson syndrome
	Relapsing polychondritis						Sjögren's syndrome
Myasthenia gravis including Lambert-Eaton myasthenic syndrome	Mixed connective tissue disorder						Idiopathic pulmonary fibrosis
							Goodpasture syndrome
Immune mediated peripheral neuropathies and plexopathies, including Guillain-Barré syndrome, Miller Fisher syndrome and other variants, chronic inflammatory demyelinating polyneuropathy, multifocal motor neuropathy and polyneuropathies associated with monoclonal gammopathy	Spondyloarthritis, including ankylosing spondylitis, reactive arthritis (Reiter's Syndrome) and undifferentiated spondyloarthritis	Autoimmune bullous skin diseases including pemphigus, pemphigoid and dermatitis herpetiformis				Medium sized and/or small vessels vasculitis, including polyarteritis nodosa, Kawasaki's disease, microscopic polyangiitis, Wegener's granulomatosis, Churg–Strauss syndrome (allergic granulomatous angiitis), Buerger's disease (thromboangiitis obliterans), necrotising vasculitis and anti-neutrophil cytoplasmic antibody (ANCA) positive vasculitis (type unspecified), Henoch-Schonlein purpura, Behcet's syndrome, leukocytoclastic vasculitis	Autoimmune glomerulonephritis, including IgA nephropathy, glomerulonephritis rapidly progressive, membranous glomerulonephritis, membranoproliferative glomerulonephritis, and mesangioproliferative glomerulonephritis

Supplementary Table S7: Adverse events of special interest (AESI).

AESI term
Novel proven (PCR-based) SARS-CoV-2 infection accompanied by symptoms
Novel proven (PCR-based) SARS-CoV-2 positivity without symptoms
Novel potential immune mediated condition
Formation of granuloma at the injection site

Abbreviation: PCR, polymerase chain reaction.

Supplementary Table S8: CoVac-1-induced CD8$^+$ T-cell responses *ex vivo*.

Sample ID	Part	CD107a [%]		IL-2 [%]		TNF [%]		IFN-γ [%]	
		day 1	day 28	day 1	day 28	day 1	day 28	day 1	day 28
UPN02	I	0.00	0.00	0.03	0.00	0.01	0.00	0.05	0.00
UPN03	I	0.02	0.04*	0.00	0.01*	0.00	0.01*	0.00	0.00
UPN05	I	0.00	0.11*	0.00	0.00	0.00	0.00	0.00	0.00
UPN06	I	0.00	0.00	0.00	0.00	0.00	0.00	0.00	0.00
UPN10	I	0.00	0.17*	0.00	0.00	0.00	0.00	0.00	0.00
UPN11	I	0.00	0.08*	0.00	0.00	0.00	0.00	0.00	0.10*
UPN12	I	0.00	0.02*	0.00	0.02*#	0.00	0.00	0.00	0.06*
UPN16	II	0.00	0.00	0.00	0.00	0.00	0.00	0.02	0.13*
UPN17	II	0.00	0.13*	0.01	0.00	0.00	0.03*	0.00	0.03*
UPN18	II	0.01	0.10*	0.00	0.00	0.00	0.00	0.00	0.00
UPN20	II	0.04	0.00	0.00	0.00	0.06	0.00	0.02	0.00
UPN23	II	0.00	0.02*	0.00	0.00	0.00	0.00	0.00	0.00
UPN24	II	0.07	0.06	0.00	0.00	0.00	0.00	0.00	0.00
UPN26	II	0.05	0.17*	0.00	0.00	0.00	0.00	0.00	0.00
UPN27	II	0.19	0.14	0.00	0.00	0.00	0.01*	0.04	0.06
UPN28	II	0.00	0.03*	0.00	0.00	0.00	0.00	0.00	0.00
UPN29	II	0.10	0.16	0.00	0.00	0.00	0.00	0.00	0.00
UPN30	II	0.00	0.00	0.00	0.00	0.00	0.00	0.00	0.00
UPN31	II	0.03	0.43*	0.00	0.00	0.00	0.00	0.00	0.00
UPN33	II	0.01	0.12*	0.00	0.00	0.00	0.00	0.02	0.10*

Vaccine-induced CD8$^+$ T-cell responses assessed *ex vivo* using surface markers and intracellular cytokine staining. * The frequency of cytokine-positive cells was ≥ 2-fold higher than the respective frequency on day 1. # The frequency of cytokine-positive cells was ≥ 3-fold higher compared to the respective negative control. Abbreviations: UPN, uniform participant number.

Supplementary Figures

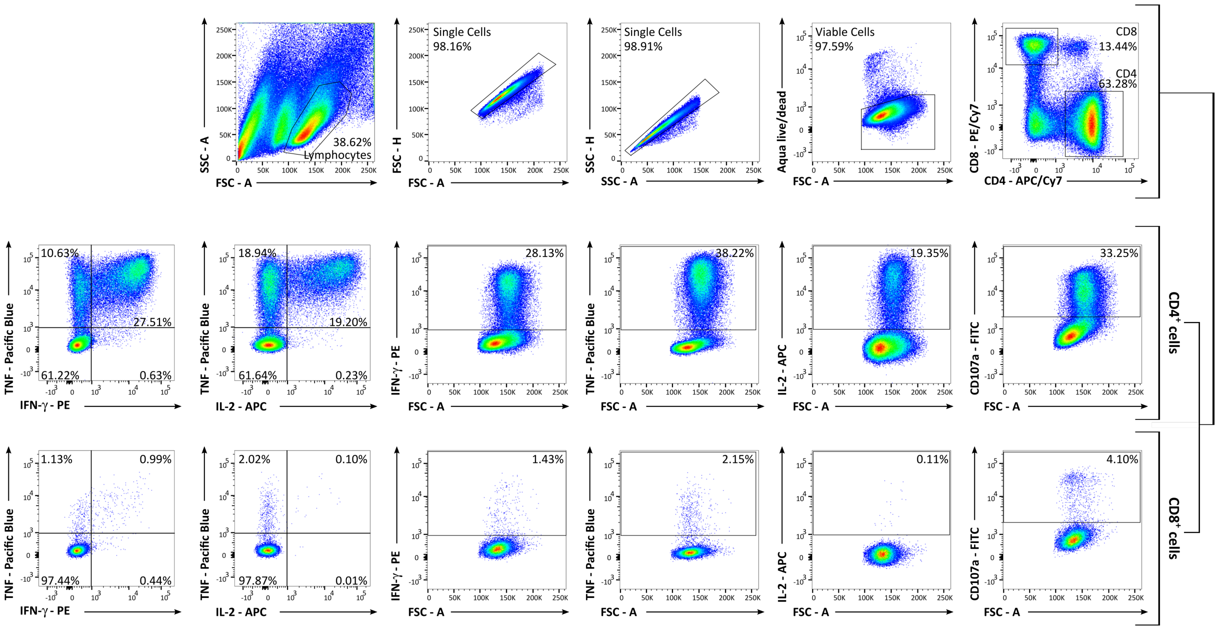

Supplementary Figure S1: Gating strategy for flow cytometry-based evaluation of surface marker and intracellular cytokine staining. Representative example showing the gating strategy for the evaluation of flow cytometry-acquired surface marker and intracellular cytokine staining data. The first gate identifies the lymphocytes (FSC-A vs. SSC-A), which are further gated for single cells (FSC-A vs. FSC-H and SSC-A vs. SSC-H) and viable cells (FSC-A vs. Aqua live/dead). Populations of $CD4^+$ and $CD8^+$ T cells (CD4-APC/Cy7 vs. CD8-PE/Cy7) are analyzed separately for different cytokines (FSC-A vs. IFN-γ-PE; FSC-A vs. TNF-Pacific Blue; FSC-A vs. IL-2-APC; IFN-γ-PE vs. TNF-Pacific Blue; IL-2-APC vs. TNF-Pacific Blue) and the degranulation marker CD107a (FSC-A vs. CD107a-FITC). This gating strategy was applied for the data presented in Figure 2d, Extended Data Table 3, Extended Data Figures 5 and 6c, and Supplementary Table S8.

References

52 Salk, J. E., Bailey, M. L. & Laurent, A. M. The use of adjuvants in studies on influenza immunization. II. Increased antibody formation in human subjects inoculated with influenza virus vaccine in a water in-oil emulsion. *Am J Hyg* **55**, 439-456, doi:10.1093/oxfordjournals.aje.a119534 (1952).

53 Meiklejohn, G. Adjuvant influenza adenovirus vaccine. *JAMA* **179**, 594-597, doi:10.1001/jama.1962.03050080006002 (1962).

54 Tavares Da Silva, F. *et al.* Optimal approaches to data collection and analysis of potential immune mediated disorders in clinical trials of new vaccines. *Vaccine* **31**, 1870-1876, doi:10.1016/j.vaccine.2013.01.042 (2013).
