# Supplementary material for: A COVID-19 peptide vaccine for the induction of SARS-CoV-2 T cell immunity
Source: Nature. 2021 Nov 23;601(7894):617–22. doi: 10.1038/s41586-021-04232-5 (PMC8791831; doi:10.1038/s41586-021-04232-5)
Supplement: Supplementary file 2 — Reporting Summary [file 41586_2021_4232_MOESM2_ESM.pdf]

nature portfolio

Corresponding author(s): Juliane S. Walz

Last updated by author(s): Oct 29, 2021

Reporting Summary

Nature Portfolio wishes to improve the reproducibility of the work that we publish. This form provides structure for consistency and transparency in reporting. For further information on Nature Portfolio policies, see our Editorial Policies and the Editorial Policy Checklist.

Statistics

For all statistical analyses, confirm that the following items are present in the figure legend, table legend, main text, or Methods section.

n/a	Confirmed	
☐	☒	The exact sample size (n) for each experimental group/condition, given as a discrete number and unit of measurement
☐	☒	A statement on whether measurements were taken from distinct samples or whether the same sample was measured repeatedly
☐	☒	The statistical test(s) used AND whether they are one- or two-sided<br>*Only common tests should be described solely by name; describe more complex techniques in the Methods section.*
☐	☒	A description of all covariates tested
☐	☒	A description of any assumptions or corrections, such as tests of normality and adjustment for multiple comparisons
☐	☒	A full description of the statistical parameters including central tendency (e.g. means) or other basic estimates (e.g. regression coefficient) AND variation (e.g. standard deviation) or associated estimates of uncertainty (e.g. confidence intervals)
☐	☒	For null hypothesis testing, the test statistic (e.g. F, t, r) with confidence intervals, effect sizes, degrees of freedom and P value noted<br>*Give P values as exact values whenever suitable.*
☒	☐	For Bayesian analysis, information on the choice of priors and Markov chain Monte Carlo settings
☒	☐	For hierarchical and complex designs, identification of the appropriate level for tests and full reporting of outcomes
☒	☐	Estimates of effect sizes (e.g. Cohen's d, Pearson's r), indicating how they were calculated

Our web collection on statistics for biologists contains articles on many of the points above.

Software and code

Policy information about availability of computer code

Data collection	Patient diary for the first 28 days after vaccination and regular visits at the trial site. Data on reactogenicity and immunogenicity were collected in an electronic case report form. Additional data on explorative endpoints was provided via electronic sheets.
Data analysis	GraphPad Prism 9.2.0, FlowJo software version 10.7.1 and SAS 9.4.

For manuscripts utilizing custom algorithms or software that are central to the research but not yet described in published literature, software must be made available to editors and reviewers. We strongly encourage code deposition in a community repository (e.g. GitHub). See the Nature Portfolio guidelines for submitting code & software for further information.

Data

Policy information about availability of data

All manuscripts must include a data availability statement. This statement should provide the following information, where applicable:

- Accession codes, unique identifiers, or web links for publicly available datasets
- A description of any restrictions on data availability
- For clinical datasets or third party data, please ensure that the statement adheres to our policy

Data supporting the findings of this study including the study protocol and the statistical analysis plan are supplied as source data with this manuscript. Further data, including de-identified participant data are available after final completion of the trial report and are shared according to data sharing guidelines upon reasonable request to the corresponding author.

Field-specific reporting

Please select the one below that is the best fit for your research. If you are not sure, read the appropriate sections before making your selection.

☒ Life sciences ☐ Behavioural & social sciences ☐ Ecological, evolutionary & environmental sciences

For a reference copy of the document with all sections, see nature.com/documents/nr-reporting-summary-flat.pdf

Life sciences study design

All studies must disclose on these points even when the disclosure is negative.

Sample size

The sample size calculation of 36 participants for the trial was based on the stopping rule (occurrence of a vaccine-related serious adverse event (SAE)) and determined to show that the incidence of SAE associated with administration of CoVac-1 does not exceed a predetermined rate of 5% (= P1). Safety of the CoVac-1 vaccine should be shown if no SAE (= P0) occurs in the study population. An evaluable sample size of 33 achieves 81.6% of power to detect a difference (P1 - P0) of 0.0499 using a one-sided exact test based on the binomial distribution with a target significance level of 0.05 (sample size determination using PASS). These results assume that the population proportion under the null hypothesis (P0) is ≤ 0.0001. Assuming a drop-out rate of 7.5% (percentage of subjects that are expected to be lost at random during the course of the study and for whom no response data concerning existence of SAE will be collected, i.e. will be treated as “missing”) the total number of 36 subjects should be enrolled in the study to achieve the threshold of 33 evaluable subjects. In total 36 subjects were included in the study.

Data exclusions

Safety and immunogenicity data were available until day 56 and month 3 after vaccination, respectively. No data were excluded from the analyses. Samples analyzed are indicated in the respective figure caption.

Replication

This is a report of an ongoing clinical trial. So far no attempt to replicate was performed.

Randomization

There was no randomization in this clinical trial as there was only one treatment arm without a control arm.

Blinding

There was no blinding performed in this clinical trial, because all participants received the CoVac-1 vaccine.

Reporting for specific materials, systems and methods

We require information from authors about some types of materials, experimental systems and methods used in many studies. Here, indicate whether each material, system or method listed is relevant to your study. If you are not sure if a list item applies to your research, read the appropriate section before selecting a response.

Materials & experimental systems

n/a	Involved in the study	
☐	☒	Antibodies
☒	☐	Eukaryotic cell lines
☒	☐	Palaeontology and archaeology
☒	☐	Animals and other organisms
☐	☒	Human research participants
☐	☒	Clinical data
☒	☐	Dual use research of concern

Methods

n/a	Involved in the study	
☒	☐	ChIP-seq
☐	☒	Flow cytometry
☒	☐	MRI-based neuroimaging

Antibodies

Antibodies used

Flow cytometry: APC/Cy7 anti-human CD4 (clone RPA-T4, Cat# 300518, BioLegend), PE/Cy7 anti-human CD8 (clone SFCI21Thy2D3, Cat# 737661, Beckman Coulter), Pacific Blue anti-human TNF (clone MAb11, Cat# 502920, BioLegend), FITC anti-human CD107a (clone H4A3, Cat# 328606, BioLegend), APC anti-human IL-2 (clone MQ1-17H12, Cat# 500309, BioLegend), and PE anti-human IFN-γ monoclonal antibodies (clone B27, Cat# 506507, BioLegend).
ELISPOT: anti-IFN-γ antibody (clone 1-D1K, Cat# 3420-3-1000, MabTech), anti-IFN-γ biotinylated detection antibody (clone 7-B6-1, Cat# 3420-6-1000 MabTech).

Validation

All antibodies were purchased from the above stated companies. Validation data / citations can be found on the suppliers' website.
APC/Cy7 anti-human CD4: https://www.biolegend.com/en-us/products/apc-cyanine7-anti-human-cd4-antibody-1933
PE/Cy7 anti-human CD8: https://www.beckman.de/search#q=737661&t=coveo-tab-techdocs&f:@category=[Certificates%20of%20Analysis]&f:@itemnumber=[737661]
Pacific Blue anti-human TNF: https://www.biolegend.com/en-us/products/pacific-blue-anti-human-tnf-alpha-antibody-4149
FITC anti-human CD107a: https://www.biolegend.com/en-us/products/fitc-anti-human-cd107a-lamp-1-antibody-4966
APC anti-human IL-2: https://www.biolegend.com/en-us/products/apc-anti-human-il-2-antibody-1348
PE anti-human IFN-γ: https://www.biolegend.com/en-us/products/pe-anti-human-ifn-gamma-antibody-1536
anti-IFN-γ antibody: https://www.mabtech.com/products/anti-human-ifn-gamma-antibody-1-d1k-purified-3420-3

anti-IFN-γ biotinylated detection antibody: https://www.mabtech.com/products/anti-human-ifn-gamma-antibody-7-b6-1-biotinylated-3420-6#tabs-min-2

Human research participants

Policy information about studies involving human research participants

Population characteristics	Eligible participants were men or women aged 18-55 (Part I) or 56-80 years (Part II). In Part I, participants were free of clinically significant health problems. In Part II, participants with stable medical history were enrolled. Participants had to refrain from blood donations during the course of the study and be willing to minimize body fluid transmission to others for 7 days after vaccination. All participants had to adhere to adequate contraception methods until three months after vaccination.<br>Exclusion criteria comprised: pregnant or lactating females, participation in another clinical trial, treatment with immunosuppressive drugs, prior or current infection with SARS-CoV-2 (proven serologically or by PCR), known previous anaphylactic reaction to any component or hypersensitivity to any component of the CoVac-1 vaccine, relevant CNS pathology or other neurological disease, positivity for HIV or active hepatitis, lymphocyte count ≤1.000/µl, blood donation within 30 days, or administration of immunoglobulins or blood products within 120 days prior to study inclusion, diabetes type II, chronic lung disease requiring drug treatment, increased liver enzymes (≥2.5 x upper limit of normal), renal failure (GFR<60ml/min/1.73m2), serious cardiovascular disease, sickle cell anemia, obesity (defined by age adjusted body mass index), or preexisting auto-immune disease except for Hashimoto thyroiditis and mild psoriasis.
Recruitment	Participants were recruited at the University Hospital Tübingen. Information on the clinical trial was provided via press release (electronic and paper based). A selection bias is not assumed. Recruited participants were screened for eligibility. First the Part I of the trial was completed (including sentinel dosing of the first participant) and after review of reactogenicity and immunogenicity by the data safety monitoring board and approval by the regulatory authorities (Paul Ehrlich Institute and local ethic committee), Part II of the trial was initiated.
Ethics oversight	The trial was approved by the Ethics Committee, University Tübingen (537/2020AMG1) and the Paul Ehrlich Institute and performed in accordance with the International Council for Harmonization Good Clinical Practice guideline. A second approval was obtained prior to recruiting in Part II of the clinical trial.

Note that full information on the approval of the study protocol must also be provided in the manuscript.

Clinical data

Policy information about clinical studies

All manuscripts should comply with the ICMJE guidelines for publication of clinical research and a completed CONSORT checklist must be included with all submissions.

Clinical trial registration	ClinicalTrials.gov: NCT04546841
Study protocol	The study protocol is provided with the submission of the manuscript.
Data collection	Participants were recruited from November 28th, 2020 to January 15th, 2021. Data were collected at screening (up to 7 days before vaccination), day 1 (vaccination, baseline), day 7, day 14, day 28, day 56 and month 3. Both reactogenicity and immunogenicity were analyzed at indicated time points by outpatients visits at the University Hospital Tübingen. In addition, participants reported on reactogenicity until day 28 by paper-based diary.
Outcomes	In this report, safety as the primary endpoint is presented until day 56. Primary safety outcomes reflect the nature, frequency, and severity of solicited adverse events (AEs) until day 56 after vaccination. In addition, the number and percentage of participants with unsolicited events until day 56 were reported.<br>The secondary endpoint immunogenicity is reported by the induction of CoVac-1-specific T-cell responses. Furthermore, explorative endpoints such as characteristics of T-cell responses were analyzed.

Flow Cytometry

Plots

Confirm that:

☒ The axis labels state the marker and fluorochrome used (e.g. CD4-FITC).

☒ The axis scales are clearly visible. Include numbers along axes only for bottom left plot of group (a 'group' is an analysis of identical markers).

☒ All plots are contour plots with outliers or pseudocolor plots.

☒ A numerical value for number of cells or percentage (with statistics) is provided.

Methodology

Sample preparation	PBMCs were incubated with 10 µg/mL of peptide, 10 µg/mL Brefeldin A (Sigma-Aldrich), and a 1:500 dilution of GolgiStop (BD) for 12 - 16 h. Staining was performed using Cytofix/Cytoperm solution (BD), APC/Cy7 anti-human CD4 (BioLegend), PE/Cy7 anti-human CD8 (Beckman Coulter), Pacific Blue anti-human TNF, FITC anti-human CD107a, APC anti-human IL-2, and PE

	anti-human IFN-γ monoclonal antibodies (BioLegend). PMA (5 μg/mL) and ionomycin (1 μM, Sigma-Aldrich) served as positive control. Viable cells were determined using Aqua live/dead (Invitrogen).
Instrument	FACS Canto II cytometer (BD)
Software	FlowJo software version 10.7.1 (BD)
Cell population abundance	No cell sorting was performed prior to functional experiments.
Gating strategy	Viable cells were determined using Aqua live/dead (Invitrogen).

☒ Tick this box to confirm that a figure exemplifying the gating strategy is provided in the Supplementary Information.
