# Supplementary material for: A COVID-19 peptide vaccine for the induction of SARS-CoV-2 T cell immunity
Source: Nature. 2021 Nov 23;601(7894):617–22. doi: 10.1038/s41586-021-04232-5 (PMC8791831; doi:10.1038/s41586-021-04232-5)
Supplement: Supplementary file 3 — This file contains the redacted study protocol and statistical analysis plan. [file 41586_2021_4232_MOESM3_ESM.pdf]

P-pVAC-SARS-CoV-2: Phase I single-center safety and immunogenicity trial of multi-peptide vaccination to prevent COVID-19 infection in adults

Short Title of Clinical Trial	P-pVAC-SARS-CoV-2
Protocol Version	V1.4
Date of Protocol	08.03.2021
EudraCT-Number	2020-002502-75
ClinicalTrials.gov-Number	
Phase	Phase I
Sponsor	University Hospital Tuebingen,<br>[redacted]<br>72076 Tuebingen<br>Germany
Investigational Medicinal Product	Multi-peptide vaccine based on SARS-CoV-2 HLA class II peptides, applied subcutaneously together with the TLR1/2 ligand XS15 emulsified in Montanide ISA 51 VG
Summary of the revision history (amendments)	None

CONFIDENTIAL This protocol contains confidential information and is intended solely for the guidance of clinical investigation. This protocol may not be disclosed to parties not associated with the clinical investigation or used for any purpose without the prior written consent of the coordinating Investigator.

I. Table of Contents

Title Page 1

I. Table of Contents 2

I.a) List of Tables 8

I.b) List of Figures 8

II. Signature Page 9

III. Contacts 11

IV. Abbreviations 14

V. Synopsis 16

1. Introduction 26

1.1. Trial Rationale and Justification 30

1.1.1. Mechanism of action and rationale for a prophylactic SARS-CoV-2 multi-peptide vaccine 30

1.1.2. Rationale for the usage of XS15 as adjuvant in the prophylactic SARS-CoV-2 multi-peptide vaccine 31

1.1.3. Rationale for selected doses 32

1.1.3.1. Dose rationale for peptides 32

1.1.3.2. Dose rationale for XS15 32

1.1.3.3. Dose rationale for Montanide ISA 51 VG 33

1.1.3.4. Rationale for one dose schedule 34

1.1.4. Rationale for trial design 34

1.1.5. Preliminary experiences from Part I of the P-pVAC-SARS-CoV-2 study 35

1.1.5.1. Safety and tolerability data after interim analysis (d28) of Part I of the study 36

1.1.5.2. Immunogenicity data after interim analysis (d28) of Part I of the study 36

1.1.5.3. Comparison of CoVac-1 to approved SARS-COV-2 vaccines (BNT126b2, Biontech SE; mRNA-12738, Moderna, Inc.; ChAdOx1, AstraZeneca) 37

1.2. Benefit / Risk Assessment 38

1.2.1. Initial benefit and risk assessment 38

1.3. Risk and benefit analysis of CoVac-1 after interim safety and immunogenicity analyses of study subjects in Part I of P-pVAC-SARS-CoV-2 41

1.4. Data and Safety Monitoring Board (DSMB): 43

2. Study Objectives 44

2.1. Primary Objective and Endpoint 44

2.1.1. Primary Endpoint 44

2.2. Secondary Objectives and Endpoints 44

2.2.1. Secondary Endpoints 44

2.3. Exploratory Objectives and Endpoints 44

2.3.1. Exploratory Endpoints 45

3. Study Design 46

3.1. Study Duration and Schedule 47

3.2. End of Study 48

4. Study Population 49

4.1. General Criteria for Subject Selection 49

4.1.1. Inclusion Criteria 49

4.1.2. Exclusion Criteria 50

5. General Information on the Investigational Medical Product (IMP) 52

5.1. Peptide Vaccine CoVac-1 52

5.1.1. Peptide cocktail 52

5.1.1.1. SARS-CoV-2-specific peptides (drug substance) 52

5.1.1.1. TLR1/2 ligand XS15 (drug substance) 52

5.1.2. Montanide ISA 51 VG 53

5.2. Manufacturing of the Investigational Medicinal Product 55

5.2.1. SARS-CoV-2-specific peptides (drug substance) 55

5.2.2. XS15 (drug substance) 55

5.2.3. Montanide ISA 51 VG 55

5.2.4. Peptide cocktail CoVac-1 (drug product) 55

5.3. Labeling of the Investigational Medicinal Product 56

5.3.1. Peptide cocktail 56

5.3.2. Montanide ISA 51 VG 56

5.4. Storage of the Investigational Medicinal Product 56

5.5. Drug Accountability, Therapy Compliance and Disposal 57

5.6. Method of Treatment Assignment 57

5.7. Dose Schedule 58

5.7.1. Dose modifications for peptide vaccine 58

5.7.2. Side effects 58

5.7.2.1. Side effects of peptide vaccination 58

5.7.2.2. Side effects of XS15 59

5.7.2.3. Side effects of Montanide ISA 51 VG 59

6. Study Procedures and Examination Method 61

6.1. Study Entry 61

6.1.1. Volunteer's Informed Consent 61

6.1.2. Screening 61

6.1.3. Enrolment 62

6.1.4. Randomisation 62

6.1.5. Concomitant Medication and Treatments 62

6.1.6. Permitted Prior and Concomitant Medications and Treatments 62

6.1.7. Prohibited Prior and Concomitant Medications and Treatments 62

6.1.8. Contraception 63

6.2. Vaccination Phase 64

6.2.1. Visit 1 (Vaccination) (Day 1) 64

6.2.2. Visit 2 (Day 7 +/- 1) 65

6.2.3. Visit 3 (Day 14 +/- 1) 65

6.2.4. Visit 4 (Interim safety) (Day 28 +/- 2) 65

6.2.5. Visit 5 (End of Safety follow-up = EOSf) 66

6.2.6. Visit 6-7 (Follow-up) (Month 3 and 6 +/- 7 days) 66

6.2.7. Volunteer's diary/card 66

6.2.8. Unscheduled Visit 66

6.3. Assessment of Efficacy 67

6.3.1. Efficacy Parameters 67

6.3.2. Methods and Timing for Assessing, Recording, and Analysing of Efficacy Parameters 67

6.4. Assessment of Safety 69

6.4.1. Safety parameters 69

6.4.2. Methods and Timing for Assessing, Recording, and Analysing Safety Parameters 70

6.5. Vaccination holding rules 70

6.6. Premature termination of clinical trial for a trial subject 71

6.7. Premature closure of a trial site 72

6.8. Premature termination of the trial 72

6.9. Follow Up 73

6.10. End of Study for Subjects 73

7. Quality control and Quality assurance 74

7.1. Risk-based approach 74

7.2. Monitoring 74

7.3. Audits/ Inspections 75

7.4. Documentation: Collection, Handling, Storage and Archiving of Data 75

7.4.1. Case Report Form 75

7.4.2. Source Data 76

7.4.3. Data Handling 76

7.4.4. Preparation/Handling/Storage/Accountability of biological samples 76

7.4.5. Handling of missing data and drop outs 77

7.4.6. Storage and Archiving of Data 77

8. Statistical Analyses 78

8.1. Study Population Definition 78

8.1.1. Sample Size and Power Consideration 78

8.2. Analysis Primary Variables 78

8.3. Analysis Secondary Variables 78

8.4. Subgroup Analysis 79

8.5. Interim Analysis 79

8.6. Stopping Rules 79

8.7. Biometric Report 80

9. Safety 81

9.1. Definition of Adverse Events and Side Effects 81

9.1.1. Adverse Events 81

9.1.2. Adverse Drug Reaction 81

9.1.3. Expectedness 82

9.1.4. AESI (adverse events of special interest) 83

9.1.5. Serious Adverse Event and Serous Adverse Reaction 83

9.2. Period of Observation 84

9.3. Documentation and Reporting of Adverse Events 84

9.3.1. Documentation and Reporting of Adverse Events by the Investigator 84

9.3.2. Assessment of Severity and Causality 85

9.3.3. Action taken 86

9.3.4. Sponsors Assessment of the SAEs 86

9.3.5. Follow-up of Initial Report 86

9.3.6. Exception of reporting 87

9.3.7. Suspected Unexpected Serious Adverse Reaction (SUSAR) 87

9.3.8. Expedited Reporting to the Regulatory Authorities 87

9.4. Examination and Report of Changes in the Risk to Benefit Ratio 88

9.4.1. Reporting to Data and Safety Monitoring Board 88

9.4.2. Report to the Investigator 88

9.5. Interim Safety analysis 88

9.6. Annual Safety Report 89

9.7. Deviations from the Protocol 89

9.8. Reporting of Pregnancy 89

10. Regulatory Consideration 92

10.1. Ethical Conduct of Clinical Study 92

10.1.1. Good Clinical Practice, Declaration of Helsinki and legal Provision 92

10.2. Subject Information and Informed Consent 92

10.3. Insurance 92

10.4. Confidentiality 93

10.5. Responsibility of the Investigator 94

10.6. Registration of the Trial 95

10.7. Continuous Information to Independent Ethics Committee 95

10.8. Approval of Protocol and Subsequent Amendments 95

11. Publications 96

11.1. Reports 96

11.2. Publication 96

12. Financing 97

13. Literature 98

14. Appendix 108

14.1. Common Terminology Criteria for Adverse Events (CTCAE) Version 108

14.2. List of central laboratories 108

14.3. Volunteer diary 109

14.4. Volunteer card 120

14.5. Intensity of solicited and unsolicited local and systemic adverse events 121

14.6. List of specific immune mediated diseases (pIMDs) 123

14.7. “Mischanleitung“ for the pharmacy of participating centers 124

I.a) List of Tables

Table 1: Table of Events 24

Table 2: Study Timelines 47

Table 3: SARS-CoV-2 specific HLA-DR vaccine peptides 54

I.b) List of Figures

Figure 1: Overall Study Design 46

Figure 2: Individual Study Procedure 47

Figure 3: Treatment sequence 47

II. Signature Page

The present trial protocol was subject to critical review and has been approved in the present version by the persons signed.

Sponsor: The University Hospital Tuebingen is sponsor for the purpose of § 4 (24) German Drug Law with complementary regulations. The internal responsibility to comply with the obligations of the sponsor in terms of these regulations stays with [redacted]

[redacted]

Name: [redacted]

Function: Sponsor's delegate and person in charge to meet the obligations of the sponsor

[redacted]

[redacted] [redacted]

[redacted]

Declaration of the Principal Investigator

By my signature, I agree to supervise personally the conduct of this study at my study site and to ensure its conduct is in compliance with the protocol, informed consent, the national laws, the ICH Good Clinical Practices Guidelines and the Declaration of Helsinki. I will train the involved personal accordingly.

[redacted]

Function: Principal Investigator, Leiterin der klinischen Prüfung according to § 4 German Drug Law (AMG)

[redacted]

Name: [redacted]

Function: Deputy Principal Investigator

Adress of the Study Center: Clinical Collaboration Unit (CCU) Translational Immunology

[redacted]

Germany

III. Contacts

Sponsor

Universitätsklinikum Tuebingen
Geissweg 3
72076 Tuebingen

Sponsor's Delegate

[redacted]
Phone: [redacted]
Fax: [redacted]
e-mail: [redacted]

Coordinating Investigator (CI)

Leiterin der klinischen Prüfung, according to § 4 German Drug Law (AMG)

[redacted]
Phone: [redacted]
Fax: [redacted]
e-mail:[redacted]

Co-Coordinating Investigator

[redacted]
Phone: [redacted]
Fax: [redacted]
e-mail:[redacted]

Scientific Coordinators

[redacted]
Phone: [redacted]

Fax: [redacted]
e-mail: [redacted]

[redacted]
Phone: [redacted]
Fax: [redacted]
e-mail: [redacted]

Biometrician

[redacted]
Phone: [redacted]
Fax: [redacted]
e-mail: [redacted]

Data management

[redacted]
Phone: [redacted]
Fax: [redacted]

Project management

[redacted]
Phone: [redacted]
Fax: [redacted]
e-mail: [redacted]

Monitoring

[redacted]
Phone: [redacted]
Fax: [redacted]
e-mail: [redacted]

SAE-Management

[redacted]
Phone: [redacted]
Fax: [redacted]
e-mail: [redacted]

IV. Abbreviations

ADR	Adverse Drug Reaction
ADE	Antibody-dependent Enhancement
ADL	Activities of Daily Living
ADV	Adenovirus
AE	Adverse Event
AESI	Adverse Event of Special Interest
AMG	German Drug Law (Deutsches Arzneimittelgesetz)
CCR	Cellular Conversion Rate
CI	Coordinating Investigator
CNS	Central Nervous System
COPD	Chronic Obstructive Pulmonary Disease
COVID-19	Coronavirus Disease 2019
COV	Coronavirus
CMV	Cytomegalovirus
CRF	Case Report Form
CTC(AE)	Common Toxicity Criteria (for Adverse Events)
CTR	Clinical trial report
DBL	Data Base Lock
DSMO	Dimethyl sulfoxide
DSMB	Data and Safety Monitoring Board
EBV	Epstein-Barr Virus
EC	Ethics Committee
EORTC	European Organisation for Research and Treatment of Cancer
EOSf	End of Safety follow-up
FCBP	Female of Child Bearing Potential
FSI	First Subject In
GCP	Good Clinical Practice
GCP-V	Good Clinical Practice Ordinance (GCP-Verordnung)
GMP	Good Manufacturing Practice
GMT	Geometric mean titer

HLA	Human Leukocyte Antigen System
HRT	Hormone Replacement Therapy
IB	Investigator’s Brochure
IC	Informed Consent
ICH	International Conference on Harmonization of Technical Requirements for Registration of Pharmaceuticals for Human Use
ICU	Intensive Care Unit
IMP	Investigational Medicinal Product
ISF	Investigator Site File
LSI	Last Subject In
LSO	Last Subject Out
MERS-CoV	Middle East Respiratory Syndrome Coronavirus
PCR	Polymerase Chain Reaction
PBMC	Peripheral Blood Mononuclear Cell
PEI	Paul-Ehrlich-Institut
pIMD	Potential Immune Mediated Disease
RNA	Ribonucleic acid
SARS-CoV-2	Severe Acute Respiratory Syndrome - Coronavirus 2
SAE	Serious Adverse Event
SmPC	Summary of Product Characteristics (deutsch: Fachinformation)
SDV	Source Data Verification
SOP	Standard Operating Procedure
SPC	Summary of Product Characteristics
SUSAR	Suspected Unexpected Serious Adverse Reaction
TLR	Toll-like receptor
TMF	Trial Master File

V. Synopsis

Sponsor	University Hospital of Tuebingen represented by<br>Medical Director: Prof. Dr. med. M. Bamberg<br>Director of Administration: G. Sonntag
Title	P-pVAC-SARS-CoV-2: Phase I single center safety and immungenicity trial of multi-peptide vaccination to prevent COVID-19 infection in adults
Short Title	P-pVAC-SARS-CoV-2
Coordinating Investigator<br>(Leiter der klinischen Prüfung, According to § 4 German Drug Law (AMG))<br><br>Co-Coordinating Investigator<br><br>Sponsor’s Delegate<br><br>Scientific Coordinator	[redacted]<br><br>[redacted]<br><br>[redacted]<br><br>[redacted]
Indication	Part I: Adults aged 18-55 years<br>Part II: Adults aged 56-80
Number of Volunteers	Total number of volunteers: 36<br>Part I: 12<br>Part II: 24

Inclusion Criteria	1. Adult male or non-pregnant, non-lactating female<br>1. Part I: Age 18-55 at the time of screening<br>2. Part II: Age 56-80 years at the time of screening<br>2. Pre-existing medical condition<br>1. Part I: Free of clinically significant health problems, as determined by pertinent medical history and clinical examination at study screening<br>2. Part II: With or without pre-existing medical condition, not requiring change in therapy or hospitalization before enrollment<br>3. Ability to understand and voluntarily sign an informed consent form<br>4. Ability to adhere to the study visit schedule and other protocol requirements<br>5. Female volunteers of child bearing potential (FCBP) and male volunteers with partners of child bearing potential, who are sexually active, must agree to the use of two effective forms (at least one highly effective method) of contraception. This should be started from the signing of the informed consent and continue until three months after vaccination
Inclusion criteria	6. Postmenopausal or evidence of non-child-bearing status. For women of childbearing potential: negative urine or serum pregnancy test within 7 days prior to study treatment. Postmenopausal or evidence of non-childbearing status is defined as:

	1. Amenorrhoea for 1 year or more following cessation of exogenous hormonal treatments<br>2. Luteinizing hormone (LH) and Follicle stimulating hormone (FSH) levels in the postmenopausal range for women under 50<br>7. Be willing to minimize blood and body fluid exposure from others for 7 days after vaccination<br>1. Use of effective barrier prophylaxis, such as latex condoms, during sexual intercourse<br>2. Avoiding the sharing of needles, razors, or toothbrushes<br>3. Avoiding open-mouth kissing<br>8. Refrain from blood donation during the course of the study
Exclusion Criteria	1. Pregnant or lactating females<br>2. Participation in any clinical study with intake of any investigational drug interfering with the study primary endpoint including:<br>o Active infection<br>o Psychatric disorders<br>o Known systemic anaphylaxis<br>3. Any concomitant disease affecting the effect of the therapeutic vaccine or interfering with the study primary endpoint<br>4. Any immunosuppressive treatment except low dose corticosteroids (equivalent to ≤10mg prednisolone/day)<br>5. Prior or current infection with SARS-CoV-2 tested serologically or by throat/nose swab (PCR)<br>6. History of Guillain-Barré syndrome<br>7. Positive serological HIV, hepatitis B or C test. In case of positive HBsAg, volunteer must provide prove of hepatitis B vaccination, otherwise volunteer must be excluded.<br>8. History of relevant CNS pathology or current relevant CNS pathology (e.g. seizure, paresis, aphasia, cerebrovascular ischemia/haemorrhage, severe brain injuries, dementia, Parkinson's disease, cerebellar disease, organic brain syndrome, psychosis, coordination or movement disorder, excluding febrile seizures as child)

	9. Baseline laboratory with lymphocyte count ≤ 1000/µl<br>10. Only Part I<br>o Acute or chronic, clinically significant psychiatric, hematologic, pulmonary, cardiovascular, or hepatic or renal functional abnormality as determined by the Investigator based on medical history, physical exam, and/or laboratory screening test
	11. All parts of the clinical trial<br>o Diabetes mellitus Typ II requiring drug treatment<br>o Chronic lung disease requiring drug treatment<br>o Any chronic liver disease or unknown liver abnormalities defined as:<br>• ALT and AST ≤ 2.5 x ULN<br>• γ-GT ≤ 2.5 x ULN<br>o Chronic renal failure defined as GFR < 60 $ml/min/1,73m^2$<br>o Serious pre-existing cardiovascular disease such as NYHA ≥ I, coronary heart disease requiring coronary surgery or known pAVK ≥ grade 2<br>o Sickle cell anemia<br>o Obesity (as defined by age adjusted body mass index)<br>12. Hospitalization at study inclusion<br>13. Administration of immunoglobulins and/or any blood products within the 120 days preceding study entry or planned administration during the study period<br>14. History of blood donation within 30 days of enrolment or planned donations within the study period<br>15. Known hypersensitivity to any of the components included in the CoVac-1 vaccine<br>16. Pre-existing auto-immune disease except for Hashimoto thyroiditis and mild (not requiring immunosuppressive treatment) psoriasis

Description of the Medical Products	IMP/Drug product/Peptide vaccine: CoVac-1 applied as one multipeptide cocktails consisting of:<br>1. SARS-CoV-2 peptides: Six promiscuous HLA-DR-restricted peptides (250 µg each) derived from different proteins of SARS-CoV-2<br>2. XS15: The lipopeptide XS15 is a water-soluble synthetic Pam_3Cys-derivative. As TLR1/2 ligand it will be included as an adjuvant in the peptide vaccine.<br>Peptides are synthesized in the GMP-certified Wirkstoffpeptidlabor at the University of Tuebingen (Prof. Stefan Stevanović) and will be formulated at the GMP-Center of the University Hospital Tuebingen. The GMP-certified Wirkstoffpeptidlabor specializes in multipeptide cocktails with variable composition and holds a production permit (Herstellungserlaubnis) for different multipeptide cocktails including the TLR 1/2 ligand XS15.<br>3. Montanide ISA 51 VG: Prior to application, the peptide cocktail (consisting of 6 SARS-CoV-2-derived peptides and XS15) will be emulsified in a water-oil emulsion 1:1 with Montanide ISA 51 VG to a final volume of 500 µl.
	Treatment schedule:<br>A single vaccination with the IMP CoVac-1 (SARS-CoV-2 HLA-DR peptides, XS15 emulsified in Montanide ISA 51 VG) (500 µl) will be applied subcutaneously (s.c.) to the abdominal skin.
Study Design:	Single center Phase I clinical trial<br>Part I:<br>12 subjects will receive an open-label 500 µl subcutaneous injection via needle and syringe of the study IMP (CoVac-1). No more than one subject per day will be enrolled. 28 days following vaccination of the 12th volunteer, there will be an interim analysis of safety and a safety review by the data safety monitoring board (DSMB) as well as an amendment to the regulatory authorities (Paul-Ehrlich Institute and Ethics Committee) before proceeding to Part II.<br>Part II:<br>24 subjects will receive an open-label 500 µl subcutaneous injection via needle and syringe of the study IMP (CoVac-1).

Aim of the Study	To evaluate the safety and immunogenicity of a single use of a SARS-CoV-2-derived multi-peptide vaccine in combination with the TLR1/2 ligand XS15 in adults
Objectives/Endpoints	Primary endpoint:<br>• The nature, frequency, and severity of AEs and/or SAEs associated with administration of CoVac-1:<br>• Solicited: ADRs/AEs occurring from the time of each injection throughout 28 days following the procedure, facilitated by use of a volunteer diary<br>• Unsolicited: AEs from the time of injection throughout 56 days following injection<br>• SAEs from the time of injection until the final study visit for each subject<br>• Incidence of AESIs until the final study visit for each subject<br>Secondary endpoints:<br>• Development of a CoVac-1 specific T-cell response to at least one of the single SARS-CoV-2 T-cell epitopes included in the CoVac-1 vaccine on Visits 2, 3, 4, 5 measured by IFN-γ ELISpot ex vivo and after in vitro T-cell amplification (compared to Visit 1), this includes:<br>• Cellular conversion rate (CCR) at Visits 2, 3, 4, 5 after immunization
	Explorative endpoints:<br>• Characteristics of T-cell response on Visits 2, 3, 4, 5 measured by ELISpot/ICS. This includes:<br>- Phenotyping of SARS-CoV-2 specific T-cells (CD4, CD8 etc.) by flow cytometry<br>- Characterization of cytokine profiles of SARS-CoV-2 specific T cells (TNF, IFN, IL-2, CD107a etc.) by intracellular cytokine staining

	- Recognition rate defined as percentage of peptides inducing a T cell response in one individual<br>- Intensity of T cell response to a single SARS-CoV-2 T cell epitope included in the CoVac-1 vaccine<br>• Induction of long-term SARS-CoV-2 specific T-cell responses 3 and 6 months after peptide vaccination.<br>• Induction of antibodies specific to the SARS-CoV-2 T-cell epitopes included in the CoVac-1 vaccine<br>In case of unexpected detection of CoVac-1 specific antibodies the following assays will be performed:<br>- Individual neutralization antibody titers<br>- Seroconversion rates<br>- Calculation of geometric mean titers (GMT) for neutralizing and binding antibodies<br>• Biomarkers and clinical characteristics influencing immunogenicity.
Statistics, Safety Variables and Stopping Rules	**Safety**:<br>In this phase I study the safety/toxicity of one vaccination will be investigated. For this purpose, it will be investigated whether the incidence of severe adverse events (SAE) associated with administration of CoVac-1 exceeds a predetermined rate of 5% (= P1 = alternative hypothesis) in the whole study population. Safety of the CoVac-1 vaccine is shown if no SAE (= P0 = null hypothesis) occurs in the study population. An evaluable sample size of 33 achieves 81.6% power to detect a difference (P1-P0) of 0.0499 using a one-sided exact test based on the binomial distribution with a target significance level of 0.05. The actual significance level achieved by this test is 0.003. These results assume that the population proportion under the null hypotheses (P0) is 0.0001. Assuming a dropout rate of 7.5% (percentage of subjects that are expected to be lost at random during the course of the study and for whom no response data concerning existence of SAE will be collected, i.e. will be treated as "missing") the total number of 36 subjects should be enrolled in the study in order to end up with 33 evaluable subjects. Sample size computed using PASS 2020 (NCSS, LLC, Kaysville, Utah, USA).<br><br>**Sample size:** 36<br>Part I:

	n=12<br>Interim Safety Analysis after Part I and a substantial amendment to authorities<br>Part II:<br>n=24
Database	A validated GCP conform clinical trial database hosted by the IKEAB Tuebingen (SecuTrial) will be used for data capture and validation in this trial
Participating Centers and Investigators	CCU Translational Immunology, Department of Internal Medicine, University Hospital Tuebingen, ([redacted])
Study Type	• AMG
Competent Regulatory Authorities	• PEI and EC
Monitoring according GCP	Monitoring of the clinical trial will be performed by the ZKS Tuebingen.
Study duration	Total study duration for individual volunteer: 6 months<br>Safety duration for individual volunteer: 8 weeks<br>Follow up (exploratory end points) for individual volunteer:<br>4 months
Length of Study/ Time Lines	Total trial duration: 1 years<br>Duration for individual patient: Safety follow-up: 8 weeks<br>Follow-up: 4 months<br>Number of visits: 8<br>FSI (First Subject In): Q4/2020<br>LSI (Last Subject In): Q1/2021<br>LSO (Last Subject Out): Q3/2021<br>DBL (Data Base Lock): Q3/2021<br>Statistical Analyses Completed: Q4/2021<br>*Trial Report Completed: Q4/2021*

Table 1: Table of Events

Protocol activities and forms to be completed	Screening	Vaccination phase[1]					Follow-up period[2]
					Interim Safety	EOSf	
	≤ - 7 days	Day 1	Day 7 +/- 1 days	Day 14 +/- 1 days	Day 28 +/- 2 days	Day 56 +/- 2 days	3 and 6 months after peptide vaccination
Visit		V1	V2	V3	V4	V5	V6-7
Informed consent[3]	X						
Demographics[4]	X						
Medical history[5]	X						X
Signs/symptoms[6]		X	X	X	X	X	
Enrolment[7]	X						
	**Clinical assessments**						
Vital signs[8]	X	X	X	X	X		
Physical examination[9]	X	X	X	X	X		
Assessment of concomitant medications[10]	X	X	X	X	X	X	
AE assessments[11]		X	X	X	X	X	X
	**Laboratory assessments**						
Hematology (*local lab)*[12]	X	X	X	X	X	X	
Blood chemistry and coagulation (*local lab)*[13]	X	X	X	X	X	X	
Immunoglobulins/Immuno phenotype[14]	X						
Urine analysis (*local lab)*[15]	X						
HBV, HCV, HIV-1, (*local lab)*[16]	X						
Pregnancy test[17]	X						
SARS-CoV-2 testing	X[18]						
	**Treatment**						
Vaccine CoVac-1[19]		X					
	**Efficacy assessment**						
T-cell response[20]		X	X	X	X	X	X
Serological response[21]		X	X	X	X	X	X

Detailed information on schedule and activities are described in the footnotes.

1. The peptide vaccination should be applied as early as possible after screening (max. 7 days) and approved eligibility of the volunteer. Vaccination phase will be 2 months and ends with the end of safety follow-up (EOSf).
2. Follow-up: After vaccination phase, volunteers will enter follow-up, which ends with the last visit 6 months after vaccination (V7, EOS).
3. Informed consent and volunteer registration: every volunteer must date and sign informed consent form to participate in this trial before starting any trial-related procedures.
4. Demographics: gender, year of birth, ethnicity
5. Medical history: The investigator has to collect information on the volunteers' medical history including prior illnesses, hospitalisations, and symptoms of a SARS-CoV-2 infection.

6. Signs/symptoms: vaccine-related and -unrelated signs and symptoms
7. Enrolment: volunteers are enrolled and registered through a screening procedure. Each volunteer will be registered under a specific Vol. ID on a subjects log kept at the trial site.
8. Vital signs: At all visits: ECOG, temperature (in grade centigrade), blood pressure/pulse. At baseline additionally: height (in cm) and weight (in kg). At V4 and V5 additionally: weight (in kg). For detailed surveillance after vaccination, please refer to section 6.2 of the study protocol
9. Physical examination: inspection, abdominal, cardiac and lung auscultation, palpation of the abdomen and lymph node sites, neurological examination, inspection of vaccination site.
10. Concomitant medications should be reported in the respective CRF pages, including drugs used for treating AEs or, if applicable, chronic diseases.
11. AE assessments: events should be documented and recorded continuously. Volunteers have to be followed for AEs from application up to 56 days or until all drug-related toxicities have been resolved, whichever is later, or until the investigator assesses AEs as “chronic” or “stable”. Each AE must be reported indicating the CTC (Version 5.0) grade. If an event stops and later restarts or CTC grading changes, all occurrences must be reported. A specific procedure for definition and reporting of SAEs is described in the protocol.
12. Hematology (local lab): hemoglobin (Hb), red blood cells (RBC), platelet count (PLT) white blood cells (WBC). Differential cell counts should be performed at baseline, at each visit during vaccination phase and thereafter at investigators discretion. Clinical status and laboratory parameters are to be followed using individual institutional guidelines and the best clinical judgment of the responsible physician, which can involve more frequent testing.
13. Blood chemistry and coagulation (local lab): Alkaline phosphatase (AP), total bilirubin, aspartate transaminase (AST/ SGOT), alanine transaminase (ALT/ SGPT), lactate dehydrogenase (LDH), and uric acid, C-reactive protein (CRP), sodium, potassium, calcium, blood urea nitrogen, creatinine, glucose: at baseline and during vaccination phase, thereafter at each visit using individual institutional guidelines and the best clinical judgment of the responsible physician, which can involve more frequent testing. Prothrombin time, aPTT, and fibrinogen will be measured at baseline and at investigator’s discretion during treatment.
14. Immunoglobulin/immunophenotype: Assessment of IgA, IgG and IgM; lymphocyte subsets: T ($CD4^+$ and $CD8^+$) as well as B and NK cells.
15. Urine analysis (local lab): pH, glucose, proteins (qualitative, dipstick accepted): at baseline and at investigator’s discretion during treatment
16. HBV, HCV and HIV-1: at baseline and thereafter at investigator’s discretion
17. Pregnancy testing: For all FCBP, pregnancy testing has to be performed at the screening visit. Negative results must be available prior to vaccination.
18. SARS-CoV-2 testing: Volunteer must be tested for prior or current SARS-CoV-2 infection. Patients should be tested by serological test and throat/nose swab. If screening takes more than 48 hours, throat/nose swab for SARS-CoV-2 infection must be repeated. The vaccine can only be applied if a negative SARS-CoV-2 PCR test is available on the day of vaccination not older than 48 hours.If patients develop SARS-CoV-2 typical symptoms until vaccination, testing should be repeated.
19. Vaccine CoVac-1: Peptide vaccination should be started as soon as possible after the screening visit. Peptide vaccination will be performed once.
20. T-cell response: 60 ml of heparin blood for immunomonitoring and analysis of peptide specific T-cell response will be analyzed by the Walz lab, KKE Translational Immunologie at the Department of Immunology, Tuebingen (central laboratory). Blood will be taken before peptide vaccination on V1, and during vaccination phase and follow-up at each visit.
21. Serological response: 10 ml of serum for analysis of serological response will be analysed by the Immunopathological Laboratory, University Hospital Tuebingen (central laboratory). Blood will be taken before peptide vaccination on V1, and during vaccination phase and follow-up at each visit.

1. Introduction

The novel coronavirus SARS-CoV-2 causes the COVID-19 disease, which especially in elderly, weakened and immunocompromised patients, shows severe and fatal courses.[1-3] In the meantime, SARS-CoV-2 has spread to a worldwide pandemic with yet incalculable medical, economic and socio-political consequences. So far, there are no established therapies and a vaccine is not yet available.

Deaths and serious illness are more common in the older population over 60 years of age.[4] Outbreaks in long-term care facilities have been observed in several countries, which pose particular challenges in terms of containment and isolation within the facility, affecting and threatening those most at risk. For patients over 65 years of age with SARS-CoV-2 infection, a high hospitalization rate of between 28.6% and 43.5% in the age group 65-74 years and between 30.5% and 58.7% in the age group 75-84 years has been described, with an associated high mortality rate of up to 30%.[4]

There are two promising options for reducing the number of severe COVID-19 disease cases in elderly and comorbid people in the future:

- The development of preemptive measures (vaccination) that prevent the disease or reduce its progression.
- A therapeutic intervention in early stages of the disease, especially in the group of ≥ 65-year-olds with the highest risk of a severe course of the disease.

Both approaches can prevent deterioration in disease course, reduce the frequency of hospital admissions and intensive care treatment and thus take the pressure off the health care system.

T-cell based immunity

T-cell immunity plays an essential role in the control of viral infections. $CD4^+$ T-helper cells (Th1) are essential for the regulation and maintenance of the immune response and for the production of antiviral cytokines, while cytotoxic $CD8^+$ T-cells (CTL) are responsible for the elimination of virus-infected cells. The recognition of viral antigens, which are presented as short peptides via the human leukocyte antigen system (HLA), is essential for the activation and function of T cells. To identify and analyze protective T-cell immune responses against viral infections in the human population, a comprehensive identification and characterization of such viral T-cell epitopes is necessary.[5] [6] This knowledge is not only essential for understanding the host's immune response and the mechanisms of long-term protection in case of virus recurrence, but also a prerequisite for the development of new and more efficient therapeutic and preventive immunotherapy approaches. Besides the generation of virus-specific T-cells *ex vivo* with subsequent transfer into the patient,[7-11] the possibility of direct vaccination with T-cell epitopes for the induction of a T-cell response directly *in vivo* is of

particular importance. Such vaccines can be used to generate immune responses against the SARS-CoV-2 without enduring COVID-19 disease. Furthermore, they can also be used therapeutically to prevent severe courses of disease in acute SARS-CoV-2 infected patients by accelerating/generating a virus-specific T-cell response and activating *in vivo* virus-specific B-cells supporting antibody production.

The findings and experience with two other zoonotic coronaviruses - SARS-CoV-1 and MERS-CoV - based on the detection of CoV-specific $CD8^+$ and long-lasting $CD4^+$ memory T-cell responses in convalescents provide evidence that T-cell immunity also plays an important role in the control of coronavirus infections.[12-15] This is even more important since studies on humoral immunity to SARS-CoV-1 provided evidence that antibody responses are short-lived and can even cause or aggravate virus-associated lung pathology.[16 17] For $CD8^+$ and Th1 $CD4^+$ T cells in contrast a crucial role in viral clearance and protection against the deadly SARS-CoV-1 infection was reported especially in terms of reported lung pathology.[12 14 15] Numerous $CD4^+$ and $CD8^+$ T-cell epitopes have been described for SARS-CoV-1 and MERS-CoV, which, due to the sequence homology of the two coronaviruses, suggest potential cross-reactivity and could also be potential T-cell epitopes for the new SARS-CoV-2 virus.[18] With regard to SARS-CoV-2, two very recent studies[19 20] described $CD4^+$ and $CD8^+$ T-cell responses against viral peptide pools in donors that had recovered from COVID-19 as well as individuals not exposed to SARS-CoV-2, indicative of potential T-cell cross-reactivity.[21-23] In own preliminary work , we define SARS-CoV-2-specific and cross-reactive $CD4^+$ and $CD8^+$ T-cell epitopes in a large collection of SARS-CoV-2 convalescents as well as non-exposed individuals and confirmed their relevance for immunity and the course of COVID-19 disease.[24] These SARS-CoV-2 T-cell epitopes show high recognition frequencies in convalescents from SARS-CoV-2 infection, suggesting their important role in the natural course and immune control of COVID-19. These T-cell epitopes represent the basis for the vaccine peptides included in the CoVac-1 vaccine.

Novel findings on SARS-CoV-2 T cell immunity

T cells play the central role in SARS-CoV-2 infection and COVID-19 disease[24-37]. Early detection of SARS-CoV-2 specific $CD4^+$ T cell responses has been correlated with a mild course of COVID-19[38], whereas high antibody levels were correlated with a more severe course of COVID-19[24 39]. CD4 T cell levels negatively correlate with virus RNA loads[36]. High diversity of SARS-CoV-2 specific T cell responses, i.e. the number of different SARS-CoV-2 T cell epitopes recognized by a subject's T cells, is correlated with a mild course of COVID-19 disease[24]. Moreover, T cells are the central component of the immune system to build long-term immunity to SARS-CoV-2 and thus protection from virus re-exposure. Available reports,

up to eight months after COVID-19, point towards a decrease and even loss of SARS-CoV-2-specific antibody responses [25 39-43] and thus raise concerns regarding the protection achieved by humoral immunity, in contrast to maintained cellular/ T cell immunity [26 27]. We could show that long-term T cell immunity is mediated by specific SARS-CoV-2 T cell epitopes, whereas T cell responses to other epitopes decreased or even got lost over time[37]. Notably, T cells can combat COVID-19 even in the complete absence of a humoral i.e. antibody-mediated immune responses: Reportedly, two patients with X-linked agammaglobulinemia, a congenital B cell deficiency syndrome, recovered from moderate COVID-19 lung disease without requirement of SARS-CoV-2 specific treatment[44].

SARS-CoV-2 peptide vaccine

The aim of this study is to investigate the safety and immunogenicity of a peptide vaccine consisting of SARS-CoV-2 specific HLA class II peptides in volunteers without prior or current SARS-CoV-2 infection.

The identification and characterization of T-cell epitopes is a long-standing and unparalleled expertise of the Department of Immunology.[45-47] This unique approach is based on i) the prediction of HLA binding sequences for HLA class I and class II alleles using the world's first prediction tool (www.syfpeithi.de[48]) and newer, more refined methods, all based on SYFPEITHI, ii) the identification of naturally presented HLA class I and class II ligands (immunopeptidomics), iii) the synthesis of synthetic peptides, and iv) the characterization of T-cell epitopes and peptide-specific $CD4^+$ and $CD8^+$ T cell responses. This strategy has been successfully applied in recent years to define and characterize T-cell epitopes derived from various viruses such as CMV, EBV, ADV and influenza as well as tumor-associated antigens of various solid and hematological malignancies [49-53].

Based on this work, the results were translated into therapeutic vaccination and T-cell transfer studies in cancer patients (e.g. NCT02802943) and viral infections[54 55]. This direct translation is made possible by the Wirkstoffpeptidlabor [redacted]) of the Department of Immunology and the GMP facility for individualized drugs at the University Hospital Tuebingen as well as our immune monitoring platform equipped with state-of-the-art, validated T-cell assays and methods.

The existing experience and logistics can be directly used for the treatment and prevention of COVID-19 disease. In preliminary work for this study, $CD4^+$ T cell epitopes have already been characterized in a large cohort of SARS-CoV-2 infected donors validating their high relevance in the natural course of COVID-19. The vaccination cocktail in the study will consist of seven promiscuous HLA class II peptides from the different proteins of the SARS-CoV-2 virus,

predicted to bind to several HLA class II allotypes. Furthermore, especially those peptides were selected that contain embedded HLA class I sequences in order to induce $CD4^+$ T cell responses and $CD8^+$ T cell responses simultaneously. Furthermore, especially for peptides derived from virus surface proteins, only sequences were selected that do not represent antibody epitopes (not accessible to antibodies due to the predicted 3D structure of the protein; for more detail see IB section 4.2.6). This should prevent the formation of antibodies against the vaccinated peptides, which could possibly have a deteriorative effect on COVID-19. Immunogenicity was proven for all HLA class II peptides included in the peptide cocktail in a large cohort of SARS-CoV-2 convalescent donors as well as for single peptides in a first vaccination of a healthy volunteer (for more detail see IB section 4.2.3).

Adjuvants

A further prerequisite for successful peptide vaccination, besides selection of optimal antigen targets, is the use of a suitable adjuvant, which is able to induce potent and long-lasting immune responses. Among the most effective approaches tested in humans is the subcutaneous injection of peptides emulsified in Montanide ISA 51 VG, a water-in-oil-emulsion, combined with the TLR9 ligand CpG.[56] However, CpG is not available for clinical trials, and a peptide/antigen vaccine emulsified in Montanide without any additional adjuvant induces no or only weak immune responses[57]. In the P-pVac-SARS-CoV-2 trial, the novel TLR1/2 ligand XS15 emulsified in Montanide ISA 51 VG will be employed as adjuvant, applied subcutaneously together with the peptide vaccine. XS15 is a water-soluble derivative of the TLR1/2 ligand Pam_3Cys and induced a strong $CD8^+$ and $Th1CD4^+$ T-cell response against free short peptides in Montanide ISA 51 VG after a single s.c. injection in a healthy volunteer as well as in cancer patients.[58] Immune responses could be induced against viral peptides (including SARS-CoV-2 derived peptides), neoepitopes derived from cancer-specific mutations as well as tumor-associated self-peptides. XS15 results in granuloma formation on the vaccination site, where the vaccinated peptides persist for at least 7 weeks. Peptide-specific T cells were detected at the granuloma site, however, with a lower frequency than in peripheral blood, which rules out the risk of T-cell sequestration, dysfunction or deletion at the vaccination site due to the use of XS15 in Montanide ISA 51 VG. Strikingly, the induced immune responses were found to persist for more than 1.5 years.

With regard to the planned study we could also show that this vaccination method is able to induce potent SARS-CoV-2 specific T-cell responses in a human volunteer (for more detail see IB of XS15 (1.0. 27 May 2020)).

1.1. Trial Rationale and Justification

1.1.1. Mechanism of action and rationale for a prophylactic SARS-CoV-2 multi-peptide vaccine

The CoVac-1 vaccine evaluated in the P-pVAC-SARS-CoV-2 study is based on multiple HLA-DR SARS-CoV-2 T-cell epitopes and aims to induce SARS-CoV-2 specific T-cells in the vaccinated donors. Antibodies other than IgM are only produced if T cell help is provided to the B cells. Therefore the rationale of the T-cell inducing CoVac-1 vaccine described here is to induce T-helper cells first, before infection and thus before B cells have first contact to the viral antigen. If the B cells then see antigen after infection, they will present the antigens(s) recognized on their HLA class II molecules, and immediately will receive help from the preactivated and expanded vaccine induced T cells. During natural infection, it would take several days for the T cells to get activated and sufficiently expanded. Thus, the production of antibodies, in particular of IgG and IgA classes, should occur much faster in the vaccinated individuals, so that the virus can be cleared faster. Of special note is here that older individuals have lower numbers of T cells, in particular $CD4^+$ T cells [59 60]. Thus, virus antigen specific $CD4^+$ T cells already preactivated and expanded at the time of infection should be especially benefitting for older individuals. Multiple studies in animal models have clearly demonstrated the requirement of $CD4^+$ T cell help for the generation of protective antibody responses (for example, influenza[61], malaria[62 63], vaccinia[64 65]). Recent studies have also demonstrated that the role of $CD4^+$ T cells in the immune response to viral infections is not limited to help for antibody production; $CD4^+$ T cells are also required to generate optimal $CD8^+$ T cell responses[66-69]. Moreover, $CD4^+$ T cells additionally can act as effector cells by the secretion of cytokines and direct killing of infected cells[70-74]. HLA class II antigens specifically activate $CD4^+$ helper T cells, therefore the CoVac-1 vaccine based on SARS-CoV-2-derived HLA class II peptides will enable a potent cellular and humoral immune response to SARS-CoV-2 preventing severe courses of COVID-19.

The development of a multi-peptide vaccine focusing on the induction of SARS-CoV-2 specific T-cell responses is further supported by several recent publications describing a decrease in neutralizing SARS-CoV-2 antibodies in COVID-19 convalescents after two to four month[39 75]. In contrast a recent study still detected SARS-CoV-1 specific T-cell 17 years after infection suggesting that in contrast to antibodies T cells might enable a long lasting immunity to SARS-CoV-2. In own preclinical data we could further detect SARS-CoV-2 specific T-cell against the T-cell epitopes in the CoVac-1 vaccine in donors after COVID-19 infection even if no antibody responses could be detected. Furthermore, we could show that donors with a high diversity of

T-cell responses to SARS-CoV-2 T-cell epitopes in terms of numbers of epitopes detected by a donors was associated with milder symptoms of COVID-19[76].

1.1.2. Rationale for the usage of XS15 as adjuvant in the prophylactic SARS-CoV-2 multi-peptide vaccine

Beside the selection of optimal antigen targets, a further important prerequisite is the use of suitable adjuvant drugs able to induce potent and long-lasting immune responses. In this clinical study, we will use for the first time the novel TLR1/2 ligand XS15 (emulsified in Montanide ISA 51 VG) which 1) is water-soluble and 2) GMP-amenable, 3) non-toxic and 4) effective in inducing T cell responses *in vivo.* The active molecular component in XS15 is Pam3Cys. This is a natural substance component found in bacteria and as such has already been used in a borreliosis vaccine (Limerix) approved in the USA in over 20,000 healthy people[77 78]. Pam3Cys was covalent with a protein compound (Surface protein A (OspA) from B. burgdorferi). In experimental peptide vaccines, Pam3Cys-peptide conjugates proved to be very efficient, but such molecules are unsuitable for pharmaceutical development, especially for personalized multi-peptide vaccines, as validation of a drug produced from them would be very costly or impossible. For this reason, the water-soluble Pam3Cys derivative XS15 was developed. This derivative has a comparable effect to the above mentioned conjugates in vitro, but is more suitable for pharmaceutical development, because it is water soluble, easily purified by HPLC and detectable by mass spectrometry. Combined with Montanide ISA 51 VG and peptides, XS15 induces efficient T-cell responses after a single injection. This is especially important for its use in prophylactic viral vaccines, as immunization of large cohorts requires highly efficient immunity induction with the lowest number of vaccinations possible. Thus, Montanide/XS15 can be considered as a GMP-amenable version of the well known Complete Freund's Adjuvans [79 80] and therefore represents the optimal adjuvant for the P-pVAC-SARS-CoV-2 study.

Based on animal toxicity data and preliminary evidence (self-administration of vaccines and information gained through administration of XS15 adjuvanted vaccines as an unproven intervention, according to physicians judgement and with informed consent, in keeping with principle 37 of the Declaration of Helsinki), we assume that a dosage of 50 µg XS15 (total dosage) administered as a vaccine together with Montanide ISA 51 VG and synthetic peptides can be considered as a safe and potentially effective strategy (for more detail see IB of XS15 (1.0. 27 May 2020)).[58 81]

1.1.3. Rationale for selected doses

1.1.3.1. Dose rationale for peptides

Previous vaccination trials were performed at peptide doses ranging from 10 to 5,000 µg per vaccination: Even though only a few of these trials included a dose finding element, there is a tendency that doses below 100 µg are not effective to induce T-cell responses whilst doses above 500 µg do not seem to generate an increasing immunogenicity. Dose-finding studies performed with viral protein-derived epitopes showed significantly stronger immune responses in the 300-500 µg range versus the 100 µg dose, without significantly higher immune responses in the 1,000 vs. 500 µg group[82]. This is supported by own data of the investigator and the Immatics Biotechnologies GmbH[83] (for more details refer to the IB of CoVac-1). Preliminary data from a healthy volunteer and cancer patients vaccinated with a personalized peptide vaccine (240-300 µg per peptide) including two of the CoVac-1 peptides (250µg) in combination with XS15 showed potent induction of T-cell responses in 100% of HV and patients and a good safety profile. Concerning safety of peptide vaccines in different doses no severe side effects were observed even with very high doses of peptides up to 30mg[84,85].

Furthermore, a similar multi-peptide vaccination study for influenza evaluated safety and immunogenicity with two doses of peptides (250µg and 500µg). No difference in the safety profile was detected for the two different doses and significant induction of functional T-cell responses were observed for both peptide doses, suggesting the dose of 250µg sufficient and safe for a prophylactic viral peptide vaccine[86].

The dose of ~250 µg per peptide per dose for CoVac-1 vaccine was selected based on these findings and on the feasibility in pharmaceutical development of the vaccines.

1.1.3.2. Dose rationale for XS15

The molecular mode of action of both the Pam3Cys conjugates and XS15 is an activation of immune cells via the toll-like receptor TLR1/2. These immune cells are mainly found in the blood and lymphoid tissues. Desired as well as toxic effects are therefore to be expected above all and presumably exclusively due to the XS15-TLR1/2 interaction with these cells, in particular through an over activation of these cells, which could then lead to a so-called cytokine release syndrome. The dose of XS15 is based on an in vitro assay that investigated both potential toxicity as well as efficiency. In these assay 10 µg/ml XS15 was shown to be the most efficient dose for the stimulation of immune cells (for more details please refer to the IB of XS15). The following considerations regarding the concentration of XS15 after a subcutaneous administration are the basis of dose finding: When used with Montanide ISA 51

VG in a total volume of 500 µl suspension, a granuloma forms rapidly at the injection site, which has a volume of estimated 2 ml. This granuloma further increases up to 8ml on day 17 after vaccination[58]. Thus, the initial local concentration of XS15 is maximally 50 µg/ml which is reduced soon thereafter to 25 µg/ml (50µg in 2 ml) and soon thereafter is diluted even more, since the granuloma increases more, so that a concentration of 10 microgram/ml will soon be reached. Further dilution will follow with the granuloma increase to 6,25mg/ml (50 µg in 8ml). Based on this in vitro experiments and considerations the dose of 50 µg was selected for further in vitro and in vivo toxicity evaluation as well as for first in vivo vaccination experiments.

In the toxicity study of mice, a dose of 50 µg XS15 in Montanide, applied locally s.c., did not reveal any toxicity beyond the long known and expected toxicity of Montanide alone. Therefore, this study proves that XS15 has no local and above all no systemic toxicity under this application method up to the above mentioned dose (for more details please refer to the IB of XS15). Furthermore, considering systemic toxicity of XS15 50µg after s.c. injection the following considerations were made: If this dose (in the absence of Montanide ISA 51 VG) is immediately distributed in the blood (6l), a maximum blood concentration of 0.008 µg/ml would be expected. At a concentration of 0.008 µg/ml no measurable reaction (stimulation of immune cells) is detected in the above described in vitro test.

When used with Montanide, the formation of a granuloma at the injection site, which has a depot effect for peptides, means that a gradual release of these peptides or XS15 into the blood can be expected. Therefore, the actual blood concentration of XS15 after administration of 50 µg in a Montanide/water emulsion is likely to be much lower than the maximum concentration of 0.008 µg/ml described above. Therefore, a systemic toxic effect of XS15 is not expected at a dose of 50 µg s.c. with or without Montanide.

1.1.3.3. Dose rationale for Montanide ISA 51 VG

Montanide™ ISA 51 VG has been used in about 300 clinical trials from phase I to phase III which represents more than 19 000 vaccines. In addition, Montanide™ ISA 51 VG has been approved in a commercial vaccine against non-small cell lung cancer (NSCLC).

Dosing of 0,25ml after 50/50 mixture with peptides is based on two published clinical studies evaluating influenza vaccines in more than 2500 donors showing high immunogenicity and a good safety profile[87 88]. Detailed information on preclinical and clinical safety data for Montanide ISA 51 VG could be found in the respective IB as well as in the attached “Human application form for Montanide ISA 51 VG”.

1.1.3.4. Rationale for one dose schedule

The combination of multi-peptide vaccine with the TLR1/2 ligand XS15 emulsified in Montanide ISA 51 VG with the above described dosing was already evaluated in a healthy volunteer as well as in cancer patients (n=12). Multi-peptide vaccines included beside tumor-associated neoepitopes and self-peptides also viral T-cell epitopes derived from CMV and SARS-CoV-2. In all vaccinated individuals peptide-specific T-cell responses could be detected after one single vaccination. For viral T-cell epitopes including SARS-CoV-2 derived peptides strong T-cell responses could even be detected ex vivo without in vitro amplification of T-cells after one single vaccination. Immune responses after vaccination were shown to last for more than 1,5 years so far. Furthermore, the safety profile of these vaccines with similar composition and dosing as for the CoVac-1 vaccine was very good after a single vaccination, showing only grade 1 local reaction at vaccination side after single injection. Therefore, the first-in-man evaluation of CoVac-1 with a single vaccination seems reasonable to enable efficient induction of immune response with the lowest possible number of vaccination and side effects. Please find below a detailed description of the data from in vivo administration of peptide vaccines in similar composition in a healthy volunteer and cancer patients (for more details please refer to the IB of CoVac-1).

1.1.4. Rationale for trial design

This is a phase I multi-peptide vaccination study using SARS-CoV-2 HLA-DR peptides in combination with the novel TLR1/2 ligand XS15 in healthy volunteers to prove safety and immunogenicity. The primary objective is incidence and severity of AEs (≥ Grade 4) after vaccination in the observational time (until day 28). Furthermore, the trial aims to expand experience on overall safety and immunogenicity in the study cohort.
This is based on the following rationale:

The SARS-CoV 2 pandemic is currently one of the major threats to the world population and requires the rapid development of effective preventive and therapeutic tools. $CD4^+$ and $CD8^+$ T-cells, as comparts of the adaptive immune system, are an important cornerstone in the control of viral infections. As state above, T-cell immunity seems to play a significant role in corona virus infections including SARS-CoV-2 and has a major impact on the course of disease including severe lung pathology as observed in COVID-19. The induction of SARS-CoV-2 specific T-cell responses therefore might represent a valuable preventive and therapeutic tool especially in the group of elderly and comorbid patients to prevent severe courses of SARS-CoV-2 infection. SARS-CoV-2 specific T-cell immunity can be achieved by peptide vaccination

applying SARS-CoV-2 specific promiscuous HLA class II T-cell epitopes. The HLA class II epitopes were selected based on the immunogenicity in a cohort of SARS-CoV-2 convalescent donors, proving their pathophysiological relevance in COVID-19.[76]

In view of the pandemic spread of COVID-19, health care systems face major challenges, as a large number of patients require hospital treatment and intensive care. As soon as the capacities of individual health care systems are exceeded, optimized care for all can no longer be guaranteed.

Containment strategies in Germany include the quarantine of infected persons and the 14-day quarantine of contact persons (incubation period). At the population level, most affected countries have reduced contacts through various measures such as closing schools, shops, restaurants and, in extreme cases, a total curfew. Without effective treatment options for COVID-19 and a vaccine available for the broad population, these measures can not be terminated, which results in immense economic and socio-political damage. This underscores the high need for the development of novel treatment approaches to prevent a severe disease course of SARS-CoV-2 infection.

Therefore this trial has been conceptualized to prove safety and immunogenicity of a peptide vaccine against SARS-CoV-2. The focus in the study population is set to older participants. This is of special interest as these people are considered to be at high risk for severe disease and society has to protect the elderly. Vaccination will be conducted in three different healthy volunteer cohorts (Part I-III), each followed by an interim safety analysis before proceeding:

- Part I: Healthy adult aged 18-55 years
- Part II: Adults aged 56-80. After proving safety and immunogenicity in a cohort of healthy volunteers aged 18-55 (Part I), an interim safety analysis will be conducted and prior to continuation with Part II approval by DSMB and of an amendment by PEI and Ethics Committee must be obtained.

1.1.5. Preliminary experiences from Part I of the P-pVAC-SARS-CoV-2 study

P-pVAC-SARS-CoV-2 is a phase I single-center safety and immunogenicity trial of multi-peptide vaccination with CoVAC-1 to prevent COVID-19 infection in adults. The study is recruiting since November 2020 and has completed the first part (healthy volunteers (n=12), age 18-55 years) in February 2021. One single subcutaneous vaccination of CoVac-1 was applied. Immunogenicity, in term of induction of T-cell responses to one or more of the six HLA-DR SARS-CoV-2 T cell epitopes included in the CoVac-1 vaccine was assessed pre-

vaccination as well as on day 7, 15 and 28 after vaccination (please refer to the IB of CoVac-1 for more details).

1.1.5.1. Safety and tolerability data after interim analysis (d28) of Part I of the study

Preliminary safety data were assessed for all volunteers of Part I of the study (n = 12) after an interim safety follow-up visit (d28). Application of CoVac-1 revealed no relevant systemic side effects, in particular no fever or other systemic inflammatory reactions, no allergic reactions and no signs of vaccine-induced autoimmune disease. As expected and intended for Montanide ISA 51 VG-including vaccines, granuloma formation at the vaccination site was observed in all study subjects (max. grade 2 in 33% of subjects)[89-91]. Further local injection site adverse events were mild and included transient erythema, swelling, itching, pain and skin ulceration. The asymptomatic granulomas persisted until day 28 without affecting daily life activities, in particular the working ability, of study subjects.

An exemplary local site reaction is depicted in the IB (Appendix 9.5). For a detailed description of all ADRs, reported please refer to section 6.8.1 of the IB.

1.1.5.2. Immunogenicity data after interim analysis (d28) of Part I of the study

Preliminary immunogenicity data were assessed of all volunteers of Part I of the study (n = 12) after the interim safety follow-up visit (d28). The single dose application of CoVac-1 revealed induction of T cell responses in 100% of vaccinated subjects (n = 12) at day 28 (Fig. 1). Induction of T cell responses was overserved at very early time points with 11/12 (93%) of subjects showing T cell responses already on day 14 after CoVac-1 vaccination. CoVac-1 induced a high diversity of T cell responses with median 5/6 vaccine peptides (range 4-6 peptides) recognized by T cells of the study subjects. CoVac-1-induced T cell responses were multifunctional with positivity for TNF (12/12 subjects), IFNy (12/12 subjects) and IL-2 (11/12 subjects, Fig. 2). CoVac-1 induced a high frequency of functional SARS-CoV-2 T cells with up to 1.8% IFNy$^+$, 2.7% TNF$^+$ and 2.5% IL-2$^+$ SARS-CoV-2-specific T cells. In addition to CD4$^+$ T cell responses, CoVac-1 also induced CD8$^+$ T cell responses in 75% of donors. These CD8$^+$ T cells targeting HLA class I T cell epitopes embedded in the CoVac-1 HLA-DR vaccine peptides were shown to be of pathophysiological relevance during natural SARS-CoV-2 infection. For a detailed description of interim immunogenicity data, please refer to section 6.2 of the IB.

1.1.5.3. Comparison of CoVac-1 to approved SARS-COV-2 vaccines (BNT126b2, Biontech SE; mRNA-12738, Moderna, Inc.; ChAdOx1, AstraZeneca)

Safety and tolerability

- In contrast to approved vaccine candidates (chills 32%, fever 14% BNT126b2, 50% chills, 8% fever mRNA-12738, chills 34%, fever 24% ChAdOx1 nCoV-19; AZD1222), no systemic inflammatory reactions were reported for CoVac-1[92-94].
- No investigator-initiated drug treatment was required for CoVac-1-induced side effects, whereas paracetamol 1g post vaccination every 4-6 hours for 24 hours after vaccination was routinely advised for participants in the phase 2/3 ChAdOx1 nCoV-19 from Astra Zeneca to reduce possible reactogenicity from vaccination[93].
- None of the side effects reported for CoVac-1 vaccination affected daily life activity or working ability of study subjects. This is in stark contrast to the inflammatory side effects caused by approved vaccine candidates, in particular ChAdOx1 nCoV-19, which cause for example inability to work for up to 72h in a large proportion of vaccinated subjects [95 96].
- Granuloma formation at the vaccination site was also reported, albeit rarely, in subjects after BNT162b2 vaccination. In contrast to CoVac-1 induced granulomas, these local reactions were indeed reported to affect subject's daily life and also required specific treatment (e.g. steroids)[97].

Vaccine design and immunogenicity

- In contrast to approved vaccine candidates, the peptide-based CoVac-1 vaccine includes validated SARS-CoV-2 T cell epitopes that were proven (i) to be frequently detected in convalescents after natural SARS-CoV-2 infection, (ii) to be of pathophysiological relevance for T cell immunity to combat COVID-19 and (iii) to mediate long-term immunity after infection. Thus, CoVac-1 is expected to induce strong and long-lasting SARS-CoV-2 T cell immunity that is comparable to T cell immunity after natural infection.
- In contrast to approved vaccine candidates that induce immune responses limited to the spike protein of SARS-CoV-2, CoVac-1 induces broad T cell immunity targeting multiple viral proteins (e.g. spike, nucleocapsid, membrane, envelope etc.). This is of particular importance in light of emerging mutations that challenge efficacy of current vaccines.

- In contrast to approved vaccine candidates that require two vaccinations, CoVac-1 induces strong T cell responses after one single vaccination.
- CoVac-1 induces earlier and stronger SARS-CoV-2 T cell responses after one single vaccination compared to the approved vaccine candidates. The detailed comparison of vaccine-induced SARS-CoV-2 T cell responses is provided in the IB section 6.2.

1.2. Benefit / Risk Assessment

1.2.1. Initial benefit and risk assessment

The assumed clinical benefit and risk of P-pVAC-SARS-CoV-2 vaccination are based on the following aspects:

- Peptide vaccination using HLA-presented peptides represents an established immunotherapy approach utilized for preventive vaccine development in infectious disease[98 99] as well as for therapeutic approaches in malignant disease. Several peptide vaccination studies in patients with malignant disease including solid tumors[83 100-102] and hematological malignancies[103-106] have proven safety and tolerability of this approach.
- Multi-peptide vaccination represents a low side-effect immunotherapy approach relying on specific immune recognition of HLA-presented peptides[107-109].
- The Wirkstoffpeptidlabor holds certificates for the production of GMP grade synthetic peptides and for the formulation of multi-peptide vaccine cocktails including the TLR1/2 ligand XS15, which allows for a rapid GMP production of the CoVac-1 vaccine. This is of great importance due to the serious threat the SARS-CoV-2 pandemic currently poses to the world population.
- All peptides included in the CoVac-1 vaccine are proven SARS-CoV-2 T-cell epitopes with pathophysiological relevance in the natural course of COVID-19 disease
- CoVac-1 peptide vaccination can induce potent $CD8^+$ and $CD4^+$Th1 T-cell responses against SARS-CoV-2 providing immunity against infection as:
  - $CD4^+$Th1 cells will directly contribute to virus clearance and deliver strong T helper signals to $CD8^+$ T cells primed during natural infection. Furthermore, these SARS-CoV-2 specific $CD4^+$Th1 cells can activate virus antigen-experienced B cells. The resulting enhanced activity could lead to more rapid virus clearance and prevention of a severe course of COVID-19 disease.
  - Vaccine peptides contain embedded CD8 T-cell epitopes predicted to bind to many HLA class I allotypes. Such $CD8^+$ T cells should also contribute to faster

virus clearance.

- Since we found IFNγ-producing SARS-Cov-2 specific T-cells in a healthy volunteer vaccinated with SARS-CoV-2 T-cell epitopes, it is very likely that significantly $CD4^+$Th1 T cells are induced by the vaccine. There should be thus no disease enhancing-effect due induction of Th2-bias as described for other corona viruses[110].
- As development of antibody-dependent enhancement (ADE) has been identified as potential risk[111] for infected patients after vaccination approaches, the following considerations and risk mitigation strategies have been undertaken:
- In contrast to other classical vaccines aiming to induce an antibody response to prevent viral infections, the CoVac-1 vaccine is designed to induce SARS-CoV-2 specific T-cells. According to experience from comparable peptide vaccines in cancer patients it is very unlikely, that such antibodies will be induced after a single vaccination. Induction of antibodies against vaccine peptides were observed in cancer patients with delay, and only after several vaccinations. So far, no antibody induction against the T-cell epitopes included in the CoVac-1 vaccine was observed.
- Furthermore and most importantly, even in the unlikely event of antibody induction against CoVac-1 vaccine peptides, which will be monitored during the study as outlined in the protocol (section 6.3.2), these antibodies cannot recognize viral particles, because none of the vaccine peptides is exposed on the virus particle surface. Thus, neither neutralizing nor ADE-inducing antibodies can be induced by the vaccine. In contrast to ADE mediated by vaccine induced antibodies, which as describe above is extremely unlikely with the CoVac-1 vaccine, there might be a risk of ADE in cases of SARS-CoV-2 infection in which the patient's B cells have already been primed against epitopes of common cold seasonal human coronavirus strains and produce low amounts of antibodies, antibodies with low affinity or antibodies with the wrong affinity. In theory, vaccine-induced $CD4^+$ T-cells might cause or exacerbate immune pathological effects indirectly. As such *in vivo* effects can not be preliminary assessed in an in vitro setting, symptoms attributable to SARS-CoV-2 infection will results in subsequent PCR testing and proven SARS-CoV-2 infection will be reported as AEs of special interest (AESI). These AESIs will be monitored particularly carefully including early hospital admission of patients with COVID-19 after CoVac-1 vaccination. This was outlined in more detail in the study protocol.

- Participant selection is based on medical care and safety considerations:
    - The trial comprises two parts (cohorts of participants) with different age ranges to provide preliminary results on safety in a cohort of young (18-55 years, n=12)

and healthy participants, which is then extended to older (Part II) participants. Of note, the risk of vaccine related (S)AEs is hypothesized to be similar in each age group.

- The design addresses the urgent medical need for protection of people at risk for serve SARS-CoV-2 infection by providing safety and immunogenicity data as well as first efficacy data in terms of SARS-CoV-2 infection in this population.
- After Part I of the clinical trial (last patient has completed V4) a substantial amendment is send to the regulatory authorities besides seeking advice from the DSMB.
- Safety is continuously monitored by an independent DSMB, which will be provided with reports on a regular basis (see DSMB Charter).

- Successful development of a peptide vaccine will help to put an end to quarantine and fear of SARS-CoV-2.
- Confirming safety of the CoVac-1 vaccine in volunteers within the P-pVAC-SARS-CoV-2 study will further allow the transfer of this approach to induce SARS-CoV-2 specific T-cell immunity in a therapeutic setting for patients with SARS-CoV-2 infection.

The assumed clinical benefit and risks of peptide vaccination in combination with the TLR1/2 ligand XS15 in Montanide ISA 51 VG are based on the following aspects:

- Peptide vaccination alone is rarely able to induce clinically effective T-cell responses; thus the peptide vaccine has to be combined with an adjuvant drug to enhance immune responses.
- Several TLR ligands have been shown to potently induce $CD8^+$/Th1$CD4^+$ responses in humans, including CPG (TLR9 ligand), imiquimod (TLR7 ligand) and poly-IC (TLR3 ligand). However, no GMP compliant substance based on these TLR ligands is available that can be applied with a peptide vaccine.
- XS15 is a water-soluble derivative of the TLR1/2 ligand Pam3Cys and induces a strong $CD8^+$ and Th1$CD4^+$ T-cell response against free short peptides emulsified in Montanide ISA 51 VG after a single s.c. injection in healthy volunteers as well as cancer patients.
- Using XS15, immune responses could be induced for viral peptides (including SARS-CoV-2 derived peptides), neoepitopes from cancer-specific mutations as well as for tumor-associated self-peptides.
- XS15 results in granuloma formation on the vaccination site, where the vaccinated peptides persist for at least 7 weeks, which supports the induction of a strong immune response.

- The induced immune responses observed so far persisted for more than 1.5 years.
- Beside formation of granuloma locally on injection side, no relevant side effects of peptide vaccination in combination with XS15 in Montanide ISA 51 VG were observed in a healthy volunteer and cancer patients. In particular, no allergic or anaphylactic reactions or cytokine release syndrome have been observed (detailed information can be found in the IB V1.0 and the IB of XS15 (1.0. 27 May 2020)).
- Montanide ISA 51 VG is an oil adjuvant suitable for human injection that allows the manufacturing of water in oil emulsions. Montanide ISA 51 VG has been used in more than 200 clinical trials including more than 6000 patients. Most common side effects are injection site reactions (68%) including granuloma development, fatigue (54%), fever (41%), gastrointestinal disorders (32%) and injection site or local erythema (28%)[89]. In general, the observed adverse from controlled trials with non-healthy as well as healthy individuals were mild to moderate in intensity.

<u>Conclusion</u>

Taking into account the lack of effective treatment options and the dismal prognosis in SARS-CoV-2 infected high-risk patient populations, especially in comorbid patients aged > 65 years, the expected benefits of a SARS-CoV-2 specific HLA class II peptide vaccination in combination with XS15 emulsified Montanide ISA 51 VG are considered to outweigh the potential risks for the participants, especially since multiple risk mitigation (e.g. interim safety analysis) measures have been incorporated.

1.3. Risk and benefit analysis of CoVac-1 after interim safety and immunogenicity analyses of study subjects in Part I of P-pVAC-SARS-CoV-2

<u>Benefits</u>

The main goal of this study is to develop a vaccine candidate that induces superior SARS-CoV-2 T cell immunity to better combat COVID-19. It has been shown that T cells play an important role for COVID-19 disease outcome and are the central component of the immune system for maintaining long-term SARS-CoV-2 immunity[24-37 39-43]. Thus, inducing broad and long-lasting SARS-CoV-2 T cell immunity is of utmost importance for COVID-19 vaccine development.

The vaccine candidate CoVac-1 was designed with the overarching aim to induce a strong and long-lasting SARS-CoV-2 T cell immunity after one single vaccination, that is comparable to T cell immunity acquired upon natural infection. In contrast to approved vaccine candidates, our peptide-based CoVac-1 vaccine includes validated SARS-CoV-2 T cell epitopes that were proven (i) to be frequently detected and in convalescents after natural SARS-CoV-2 infection, (ii) to be of pathophysiological relevance for T cell immunity to combat COVID-19 and (iii) to mediate long-term immunity after infection. Furthermore, and again in contrast to approved vaccines which only induce immune responses that are limited to the spike protein of SARS-CoV-2, CoVac-1 induces broad T cell immunity targeting multiple viral proteins (e.g. spike, nucleocapsid, membrane, envelope etc.). This is of special importance in the light of emerging mutations that challenge the efficacy of the currently available vaccines inducing immune responses limited to the spike protein.

Preliminary immunogenicity analyses on d28 in the study subjects included in Part I of our P-pVAC-SARS-CoV-2 study documented superior induction SARS-CoV-2 T cell immunity after one single CoVac-1 vaccination as compared to the approved vaccine candidates (BNT16B1, mRNA-1273 and ChAdOx1 nCoV-19), which all require a second booster vaccination [92-94]. Of note, superiority of CoVac-1-induced T cell responses was shown in terms of multiple aspects: (i) diversity of T cell responses, (ii) frequency and intensity of functional SARS-CoV-2-specific T cells, and (iii) short time until occurrence of documented T cell.

These advantages of CoVac-1 are achieved without causing any systemic inflammatory side effects, e.g. fever or chills. Thus, in contrast to the approved vaccines, CoVac-1 does neither affect activities of daily life nor the working ability of study subjects[95 96].

<u>Risks</u>

The main (per definition) adverse event identified for CoVac-1 is the induction of a granuloma locally at injection site (max. grade 2 in 33% of subjects). These totally asymptomatic granulomas were still detectable on day 28 (time of interim safety analysis). However, it should be noted that granuloma development represents an expected and, even more, intended local reaction after vaccination that is required to enable the continuous local priming of SARS-CoV-2 specific T cells and thus the induction of long-lasting T cell responses while at the same time preventing systemic inflammation. Granuloma formation was also rarely reported after mRNA-based vaccines [97], where it required systemic steroid treatment. CoVac-1 induced granulomas, in contrast, did not require any investigator-initiated medication and did not affect the daily life activities, in particular the working ability, of our study subjects.

Conclusion

Together, in our view the available safety and immunogenicity data of CoVac-1 provide a profound rationale for the continued evaluation of CoVac-1 and thus conduct of the second part of the study. This is based, among others, on the comparison to the three vaccine candidates already approved by the EMA, which showed a clear superiority of CoVac-1 to induce SARS-CoV-2 specific T cell immunity, in terms of frequency, intensity and diversity of T cell responses. Thus, especially in the light of emerging mutations and concerns regarding long-term humoral immunity, CoVac-1 represents a highly promising vaccine candidate to combat COVID-19.

The interim safety and immunogenicity data were presented to the DSMB and all DSMB members agreed with and support the conduct of the second study part.

1.4. Data and Safety Monitoring Board (DSMB):

An independent Data and Safety Monitoring Board (DSMB) will be assembled. The DSMB will be composed of independent experts in the field of immunology and infectiology assessing the progress, safety data and critical efficacy endpoints. The mission of the DSMB is to ensure the ethical conduct of the trial and to protect the safety interests of participants in this trial.
The DSMB will receive a report listing and summarizing all the relevant safety data at least twice. The first assessment (first interim safety report, section 9.5) will take place after Part I of the trial including DSMB approval and an amendment at the regulatory authorities (Paul-Ehrlich Institute, PEI) and Ethics Committee (EC). If the IMP is considered safe for continuation by DSMB, Part II of the trial will start recruiting. In addition, the report will provide data concerning recruiting rates, status of the trial and AESIs (section 9.1.4); also non-occurrence will be mentioned. An emergency meeting of the DSMB may be called at any time should questions of volunteer safety arise or holding rules apply, and necessary safety reports will be provided. Meetings may be convened as conference calls/e-mail as well as in person.

2. Study Objectives

2.1. Primary Objective and Endpoint

The primary objective of this trial is to evaluate the safety and tolerability of the CoVac-1 vaccine, a single dose SARS-CoV-2 specific multi-peptide vaccine combined with the TLR1/2 ligand XS15 emulsified in Montanide ISA 51 VG in adults.

2.1.1. Primary Endpoint

The nature, frequency, and severity of AEs and/or SAEs associated with administration of CoVac-1:

- Solicited: ADRs/AEs occurring from the time of each injection throughout 28 days following the procedure, facilitated by use of a volunteer diary
- Unsolicited: AEs from the time of injection throughout 56 days following injection
- SAEs from the time of injection until the final study visit for each subject
- Incidence of AESIs until the final study visit for each subject

2.2. Secondary Objectives and Endpoints

Secondary objectives of this trial are to evaluate the efficacy of the CoVac-1 vaccine in terms of induction of SARS-CoV-2 specific T-cells.

2.2.1. Secondary Endpoints

- Development of a CoVac-1 specific T-cell response to at least one of the single SARS-CoV-2 T-cell epitopes included in the CoVac-1 vaccine on Visits 2, 3, 4, 5 measured by IFN-γ ELISpot ex vivo and after in vitro T-cell amplification (compared to Visit 1), this includes:
  - Cellular conversion rate (CCR) at Visits 2, 3, 4, 5 after immunization

2.3. Exploratory Objectives and Endpoints

Explorative objectives are the duration and characteristics of T-cell responses and the analysis of induction of antibody responses to single SARS-CoV-2 T-cell epitopes included in the CoVac-1 vaccine.

2.3.1. Exploratory Endpoints

- Characteristics of T-cell response on Visits 2, 3, 4, 5 measured by ELISpot/ICS. This includes:
  - Phenotyping of SARS-CoV-2 specific T-cells (CD4, CD8 etc.) by flow cytometry
  - Characterization of cytokine profiles of SARS-CoV-2 specific T cells (TNF, IFN, IL-2, CD107a etc.) by intracellular cytokine staining
  - Recognition rate defined as percentage of peptides inducing a T cell response in one individual
  - Intensity of T cell response to a single SARS-CoV-2 T cell epitope included in the CoVac-1 vaccine
- Induction of long-term SARS-CoV-2 specific T-cell responses 3 and 6 months after peptide vaccination.
- Induction of antibodies specific to the SARS-CoV-2 T-cell epitopes included in the CoVac-1 vaccine measured by ELISA. In case of unexpected detection of CoVac-1 specific antibodies the following assays will be performed:
  - Individual neutralization antibody titers
  - Seroconversion rates
  - Calculation of geometric mean titers (GMT) for neutralizing and binding antibodies
- Biomarkers and clinical characteristics influencing immunogenicity.

3. Study Design

This is an interventional, open-label, phase I trial evaluating the CoVac-1 vaccine, a single dose SARS-CoV-2 specific multi-peptide vaccine combined with the TLR1/2 ligand XS15 emulsified in Montanide ISA 51 VG in adults. The study is divided into two parts, which will recruit consecutively. Prior to initiation of the next part, the previous part must have completed recruiting, and day 28 of the last patient enrolled must have passed. After interim safety analysis and approval from the authorities (section 9.5), the next study part starts recruiting (Figure 1 and 2).

The first volunteer included in the trial will be hospitalized after vaccination and closely monitored. This patient is observed until day 28 and possibly arising safety issues are reported to and decided on by the Sponsor. Thereafter, no more than one subject per day will be treated/vaccinated. 28 days following vaccination of the 12th volunteer, there will be an interim analysis of safety and a safety review by the data safety monitoring board (DSMB) as well as a substantial amendment to the regulatory authorities (PEI and EC) before proceeding to Part II. Part II must not start recruiting prior to approval by authorities. Volunteers of part II are treated simultaneously. Details can be found in figure 3.

To avoid bias in treatment, a manualized process protocol as well as monitoring and treatment reports are implemented. The volunteer selection will be documented. Reasons for refusal will be assessed. To avoid bias in data analysis, monitoring and analysis by intention-to-treat are planned. Data analysis will be conducted by an independent statistician.

Figure 1: Overall Study Design

Figure 2: Individual Study Procedure

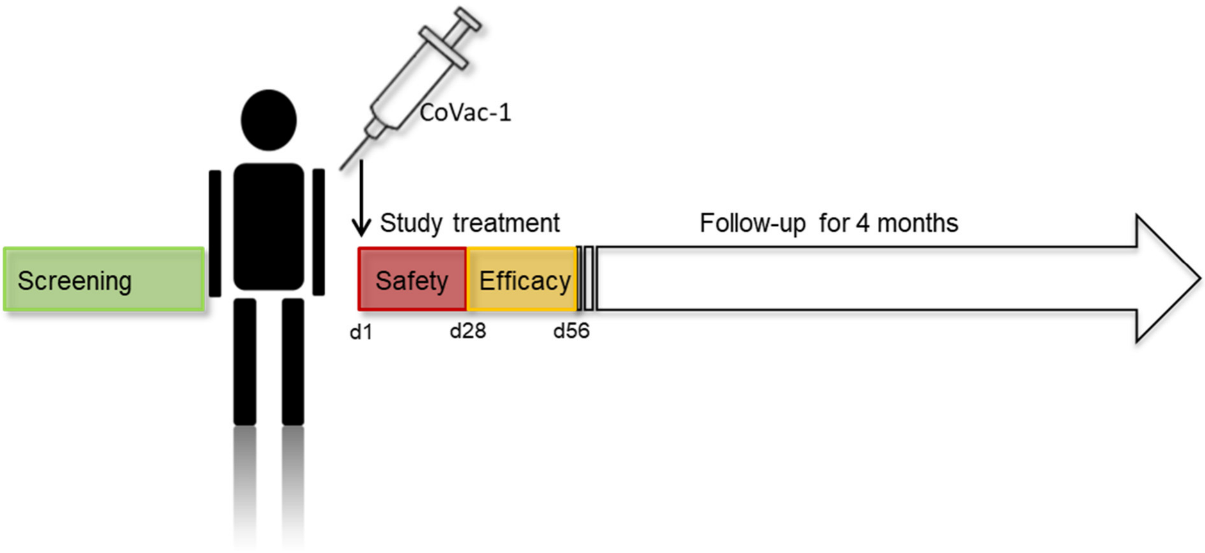

Figure 3: Treatment sequence

3.1. Study Duration and Schedule

The duration of the trial for each subject is expected to be 6 months, including 2 months of safety follow-up after vaccination and 4 months of follow-up.

The overall duration of the trial is expected to be approximately 12 months including the preparatory phase. Recruitment of subjects will start in Q3 2020. The actual overall duration or duration of recruitment may vary. The study timeline is described in Table 2.

Table 2: Study Timelines

Total trial duration	12 months
Duration for individual volunteer	Study treatment: 2 months<br><br>Follow-up: 4 months
FSI (First Subject In)	Q4/2020
LSI (Last Subject In)	Q1/2021

LSO (Last Subject Out)	Q3/2021
DBL (Data Base Lock)	Q3/2021
Statistical Analyses Completed	Q4/2021
Trial Report Completed	Q4/2021

3.2. End of Study

The end of the study is defined as the last visit of the last volunteer.

4. Study Population

Healthy subjects (designated as volunteers):

Healthy adult women and men aged 18-55 (Part I), followed by adult women and men aged 56-80 with age adjusted health condition (Part II).Volunteers will be recruited by means of paper- and online-based calls as considered appropriate by the EC of the University Hospital of Tuebingen.

4.1. General Criteria for Subject Selection

Adult male and female volunteers fulfilling the inclusion criteria outlined below will be enrolled. The trial population will consist of both genders. Gender distribution in the trial is supposed to reflect the distribution in the population; there will be no prior defined quantitative ratio between females and males.

4.1.1. Inclusion Criteria

Subjects meeting all of the following criteria will be considered for admission to the trial:

1. Adult male or non-pregnant, non-lactating female
    1. Part I: Age 18-55 at the time of screening
    2. Part II: Age 56-80 years at the time of screening
2. Pre-existing medical condition
    1. Part I: Free of clinically significant health problems, as determined by pertinent medical history and clinical examination at study screening
    2. Part II: With or without pre-existing medical condition, not requiring change in therapy or hospitalization before enrollment
3. Ability to understand and voluntarily sign the informed consent form.
4. Ability to adhere to the study visit schedule and other protocol requirements.
5. FCBP and male volunteers with partners of childbearing potential, who are sexually active must agree to the use of two effective forms (at least one highly effective method) of contraception. This should be started from the signing of the informed consent and continue until three months after vaccination
6. Postmenopausal or evidence of non-childbearing status. For women of childbearing potential: negative urine or serum pregnancy test within 7 days prior to study treatment. Postmenopausal or evidence of non-childbearing status is defined as:

    1. Amenorrhoea for 1 year or more following cessation of exogenous hormonal treatments
    2. Luteinizing hormone (LH) and Follicle stimulating hormone (FSH) levels in the post-menopausal range for women under 50
7. Be willing to minimize blood and body fluid exposure of others for 7 days after vaccination
    1. Use of effective barrier prophylaxis, such as latex condoms, during sexual intercourse
    2. Avoiding the sharing of needles, razors, or toothbrushes
    3. Avoiding open-mouth kissing
    4. Refrain from blood donation during the course of the study

4.1.2. Exclusion Criteria

Subjects presenting with any of the following criteria will not be included in the trial:

1. Pregnant or lactating females.
2. Participation in any clinical study with intake of any investigational drug interfering with the study primary endpoint
3. Any concomitant disease affecting the effect of the therapeutic vaccine or interfering with the study primary endpoint
4. Any immunosuppressive treatment except low dose corticosteroids (≤10mg prednisolone/day)
5. Prior or current infection with SARS-CoV-2 tested serologically or by throat/nose swab (PCR)
6. History of Guillain-Barré Syndrome
7. Positive serological HIV, hepatitis B or C test. In case of positive HBsAg, volunteer must provide prove of hepatitis B vaccination, otherwise volunteer must be excluded.
8. History of relevant CNS pathology or current relevant CNS pathology (e.g. seizure, paresis, aphasia, cerebrovascular ischemia/hemorrhage, severe brain injuries, dementia, Parkinson’s disease, cerebellar disease, organic brain syndrome, psychosis, coordination or movement disorder, excluding febrile seizures as child)
9. Baseline laboratory with lymphocyte count ≤ 1000/µl
10. Only Part I:
    - Acute or chronic, clinically significant psychiatric, hematologic, pulmonary, cardiovascular, or hepatic or renal functional abnormality as determined by the

Investigator based on medical history, physical exam, and/or laboratory screening test

11. All parts of the clinical trial
    - Diabetes mellitus Typ II requiring drug treatment
    - Chronic lung disease requiring drug treatment
    - Any chronic liver disease or unknown liver abnormalities defined as:
        - ALT and AST ≤ 2.5 x ULN
        - γ-GT ≤ 2.5 x ULN
    - Chronic renal failure defined as GFR < 60 ml/min/1,73m^2
    - Serious pre-existing cardiovascular disease such as NYHA ≥ I, coronary heart disease requiring coronary surgery or known pAVK ≥ grade 2
    - Sickle cell anemia
    - Obesity (as defined by age adjusted body mass index)
12. Hospitalization at study inclusion
13. Administration of immunoglobulins and/or any blood products within 120 days preceding study entry or planned administration during the study period
14. History of blood donation within 30 days of enrolment or planned donations within the study period
15. Known hypersensitivity to any of the components included in the CoVac-1 vaccine
16. Pre-existing auto-immune disease except for Hashimoto thyroiditis and mild (not requiring immunosuppressive treatment) psoriasis

5. General Information on the Investigational Medical Product (IMP)

Definition of terms

Drug substances: Six SARS-CoV-2-derived HLA class II peptides derived and the TLR1/2 ligand XS15

Peptide cocktail: Peptide cocktail for each study volunteer including 6 immunogenic SARS-CoV-2 peptides and the TLR1/2 ligand XS15

IMP/Drug product/ peptide vaccine: CoVac-1: Peptide cocktail emulsified in Montanide ISA 51 VG

IMP administration: subcutaneous injection with 2ml syringe (e.g. BD Emerald) and needle (e.g. BD Eclipse Needle 27Gx1/2)

5.1. Peptide Vaccine CoVac-1

The IMP/drug product in this study is CoVac-1. The final peptide vaccine is a water-in-oil emulsion of the peptide cocktail as described in detail below and Montanide ISA 51 VG. All components will be provided by the Wirkstoffpeptidlabor of the Department of Immunology in Tübingen together with a “mixing kit” allowing for the mixture of the components (peptide cocktail, Montanide ISA 51 VG) by the pharmacy of the participating centers.

5.1.1. Peptide cocktail

5.1.1.1. SARS-CoV-2-specific peptides (drug substance)

Each volunteer enrolled in the P-pVAC-SARS-CoV-2 trial will receive 6 promiscuous HLA-DR peptides (250 µg each) derived from different proteins of SARS-CoV-2. Details on drug substance can be found in Table 3.

5.1.1.1. TLR1/2 ligand XS15 (drug substance)

The lipopeptide XS15 (50 µg), chemical name N-Palmitoyl-S-[2,3-bis(palmitoyloxy)-(2R)-propyl]-(R)-cysteinyl-GDPKHPKSF, a water-soluble synthetic Pam_3Cys-derivative is a TLR1/2 ligand that will be included as an adjuvant in the peptide cocktail.

5.1.2. Montanide ISA 51 VG

Prior to application, the peptide cocktail (consisting of 6 SARS-CoV-2-specific HLA-DR peptides and the TLR1/2 ligand XS15) will be emulsified in a water-oil emulsion 1:1 with Montanide ISA 51 VG. Montanide ISA 51 VG is based on a blend of mannide monooleate surfactant and mineral oil and has been used as an adjuvant in more than 200 human vaccine trials. Montanide ISA 51 VG is rendering stable water-in-oil emulsions when mixed with water-based antigenic media.

Table 3: SARS-CoV-2 specific HLA-DR vaccine peptides

sequence	HLA restriction	peptide length	position	protein	protein name	protein class
ASWFTALTQHGKEDL	DR	15	50-64	ORF9	nucleocapsid protein	structural
LLLLDRLNQLESKMS	DR	15	221-235	ORF9	nucleocapsid protein	structural
ITRFQTLLALHRSYL	DR	15	235-249	ORF9	spike protein	structural
LSYYKLGASQRVAGD	DR	15	176-190	ORF5	membrane protein	structural
FYVYSRVKNLNSSRV	DR	15	56-70	ORF4	membrane protein	structural
SKWYIRVGARKSAPL	DR	15	43-57	ORF8	n.a.	non-structural

5.2. Manufacturing of the Investigational Medicinal Product

5.2.1. SARS-CoV-2-specific peptides (drug substance)

All SARS-CoV-2 vaccine peptides are manufactured by the Wirkstoffpeptidlabor, University of Tübingen, Auf der Morgenstelle 15, 72076 Tübingen, Germany. The Wirkstoffpeptidlabor holds certificates for the production of GMP grade synthetic peptides and for the formulation of multi-peptide vaccine cocktails including the TLR1/2 ligand XS15. All peptides are synthetic peptides manufactured by well-established solid phase peptide synthesis (SPPS) procedures using Fmoc chemistry.

5.2.2. XS15 (drug substance)

XS15 is delivered as bulkware in GMP-quality from the external manufacturer Bachem AG, Hauptstrasse 144, CH-4416 Bubendorf in active ingredient quality.

Bachem's manufacturing process is described in a separate "Documentation on XS15 Hydrochloride" of 31.05.2018 by the company. The Wirkstoffpeptidlabor performs a second lyophilization as additional manufacturing step. This manufacturing step is divided into four sub-steps: Reconstitution, combining, aliquoting and lyophilization.

5.2.3. Montanide ISA 51 VG

Montanide is manufactured by Seppic and by the rewarding manufacturer Elaiapharm, respectively.

5.2.4. Peptide cocktail CoVac-1 (drug product)

The peptide cocktail is manufactured by the Wirkstoffpeptidlabor by aseptic filling at the GMP-Center of the University Hospital Tuebingen. Each peptide is solubilized in DMSO and sterile filtered, the obtained peptide solutions are pooled. Water is added and the obtained solution is sterile filtered and filled into single dose vials.

5.3. Labeling of the Investigational Medicinal Product

5.3.1. Peptide cocktail

Peptide cocktails (including the TLR1/2 ligand XS15) will be packaged into sterile containers labeled with an identification code definitely assignable to the P-pVAC-SARS-CoV-2 study and a vial number that will be assigned to the individual study volunteer. The trial medication will be labeled according to § 5 of GCP-V. Samples of the labels are filed in the trial master file (TMF).

The peptide vaccine cocktail will be packaged together with Montanide ISA 51 VG and the mixing equipment into the “mixing kit” and shipped from the *Wirkstoffpeptidlabor* of the Department of Immunology, Tübingen to the pharmacy of the participating center. Shipment will be documented according to standard operation procedures (SOP). The “mixing kit” will be shipped using isolated packaging with an automated temperature control system, whose logging data have to be returned to the Wirkstoffpeptidlabor of the Department of Immunology together with the acknowledgement of receipt after delivery of the consignment. The device will be read out to document the correct storage temperatures during shipment. Data will be documented according to SOP. The shipment will be performed by an associate of the Wirkstoffpeptidlabor of the Department of Immunology, Tübingen.

5.3.2. Montanide ISA 51 VG

Montanide ISA 51 VG is packed by Seppic and Elaiapharm. Montanide will be packaged together with the peptide cocktail and the mixing equipment into the “mixing kit” and shipped from the Wirkstoffpeptidlabor of the Department of Immunology, Tübingen to the pharmacy of the participating center, as described above.

5.4. Storage of the Investigational Medicinal Product

Trial medication will be stored at the pharmacy of the participating center and must be kept in a locked area with access restricted to designated trial staff. The “mixing kit” including the peptide cocktail and Montanide ISA 51 VG must be stored in accordance with manufacturer’s instructions at -20°C and dry. The investigator must ensure that the investigational products are stored according to the sponsor’s instructions (temperature, light and humidity) and should control the integrity of the packaging upon receipt. If concerns about the quality or appearance of the investigational products arise, the products may not be dispensed. In this case, the principal investigator must be contacted immediately.

5.5. Drug Accountability, Therapy Compliance and Disposal

The investigator or the site personnel will keep an account of the trial medication and acknowledge the receipt of all shipments of the trial medication. Trial medication will be ordered by the investigator and delivered by the Wirkstoffpeptidlabor to the pharmacy of the participating center. The investigator will document the date of dispensary, subject identification, batch/serial numbers or other identification of trial medication. Upon completion or termination of the study, all unused “mixing kits” have to be returned to the Wirkstoffpeptidlabor of the Department of Immunology. The returned products must be accompanied by adequate documentation and identified clearly with trial site and patient number. The return of any unused study medication must be coordinated by the responsible study monitor/study nurse/pharmacy. Empty packaging does not have to be returned. The disposal is in the responsibility of the study center according to the German laws and local and institutional guidelines and procedures for litter disposal.
In case of SAEs related to the vaccination peptides or adjuvant, the study medication will be returned to the Wirkstoffpeptidlabor of the Department of Immunology, Tübingen for further analysis. The returns will be documented according to SOP.

The returned charges will be locked and deleted according to SOP. A declassification of a drug for clinical use for an application in *in vitro* research experiments is not touched by the declaration. This declassification will be documented. Unused charges of vaccination peptides will be returned to the Wirkstoffpeptidlabor of the Department of Immunology, Tübingen and will be stored.

All waste will be discharged according to German waste laws (date of issue 27.09.1994).

The IMP CoVac-1 may only be applied to subjects included in the P-pVAC-SARS-CoV-2 trial. Other individuals must not receive peptides produced for the P-pVAC-SARS-CoV-2 trial.

Investigational products must be dispensed only by trained and authorized personnel according to legal regulations. Physicians outside the study facility may not apply the study drugs.

5.6. Method of Treatment Assignment

After screening and enrolment, volunteers will be assigned to treatment with CoVac-1.

5.7. Dose Schedule

The CoVac-1 vaccine (500 µl) will be administered subcutaneously. Emulsification will be performed by the pharmacy of the participating center according to the “Anmischanleitung Montanide-Emulsion” provided with the “Mixing Kit” by the Wirkstoffpeptidlabor of the Department of Immunology Tübingen. Final vaccine drug product has to be stored at room temperature and to be administered within 24 h after mixing of the components. For qualification of the pharmacy and study center staff regarding ordering and mixing of the peptide vaccine cocktail with Montanide ISA 51 VG, a controlled dry run process will be performed.

The mixing of the peptide vaccine cocktail and Montanide ISA 51 VG will be performed by local pharmacy and the investigator will be provided with a syringe containing the final drug product CoVac-1. A subcutaneous injection of 500 µl (approx. 250 µg per peptide, 50µg XS15) will be applied. A single vaccination per patient will be conducted.

<u>Vaccination instruction</u>

Peptide vaccines should be injected into the skin at the lower part of the abdomen of the volunteers. The site of vaccination (right or left) will be determined by the investigator. At investigators discretion antihistamins such as 4 mg dimetindene can be applied as i.v. injection or infusion about 30 minutes prior to application of the vaccine.

5.7.1. Dose modifications for peptide vaccine

No dose modification is planned in this trial.

5.7.2. Side effects

5.7.2.1. Side effects of peptide vaccination

Peptide vaccination is generally well tolerated. Mild reactions at local vaccination sites are the most common side effects, followed by fatigue[102 112]. Peptide vaccination can lead to immediate anaphylactic reactions with elevation of heart rate, hyperhidrosis and subjective feeling of dizziness, in rare cases with concomitant drop in blood pressure[83 83 102]. Cutaneous erythema at the vaccination site was observed more frequently and may persist for up to five weeks. Also, there is a risk of granuloma formation. Some of the patients reported one episode of fever not lasting more than two days. No grade III or IV toxicities were observed in former peptide

vaccination studies, including an early trial with a peptide based malaria vaccine, which only reported mild local reactions in approximately 50% of volunteers[83 99 102]. Furthermore, no signs for the development of antibody-dependent enhancement (ADE) was reported. Of note, side effects in the reported studies are most likely attributable to the applied adjuvants.

In our ongoing iVAC-CLL01 study using peptide cocktails, most of the patients experienced mild local skin reactions at the vaccination site. No anaphylactic or allergic reaction, or other AE related to the peptide vaccine was observed.

Preliminary safety results of volunteers (n = 12) in part I of the P-pVAC-SARS-CoV-2 study showed as intended and expected developed a local granuloma at injection site in all volunteers (100%). Further local injection site adverse events included transient erythema (100%), swelling (100%), itching (83%), pain (58%) and skin ulceration (8%). Until day 28 no relevant systemic side effects, especially no fever or other inflammatory reactions were reported. No allergic reactions were observed. In some participants fatigue (25%), headache (16%), nausea (16%), myalgia (8%) and arthralgia (8%) were reported.

In the P-pVAC-SARS-CoV-2 study, patients will be monitored for heart rate, blood pressure, temperature and subjective well-being after vaccination for at least 2 hours. The volunteers will be discharged after documentation of these parameters. More detailed information on CoVac-1 vaccine peptides is provided with the current IB (Version 1.0).

5.7.2.2. Side effects of XS15

The TLR 1/2 ligand XS15 will be administered subcutaneously together with the SARS-CoV-2 specific peptides emulsified in Montanide ISA 51 VG. XS15 was never used in a clinical trial before. Common side effects of other TLR ligands used for peptide vaccination are reported to be usually mild, comprising local skin reactions, fatigue, flu-like symptoms like fever, muscular pain and ague. TLR ligands can worsen pre-existing autoinflammatory skin disorders.

Previous application of XS15 in a healthy volunteer and cancer patients (within the scope of individual healing attempts) did, besides local reactions at the vaccination site including formation of granuloma, not cause relevant systemic side effects, in particular no allergic or anaphylactic reactions. More detailed information on XS15 is provided with the current IB (1.0. 27 May 2020).

5.7.2.3. Side effects of Montanide ISA 51 VG

Montanide ISA51 is an oil adjuvant suitable for human injection that will be administered together with the SARS-CoV-2 specific peptides and XS15 subcutaneously. Montanide ISA 51 VG was used as an adjuvant in more than 100 peptide vaccination. Most common side effects

are injection site reaction (68%) including granuloma development, fatigue (54%), fever (41%), gastrointestinal disorders (32%) and injection site or local erythema (28%)[89]. In general, the observed AEs from controlled trials involving non-healthy as well as healthy individuals were mild to moderate in intensity. Further side effects rarely reported were erythema nodosum (2/36 patients, 5%)[113] and the development of sterile abscesses at injection site (10%)[89-91].

More detailed information on Montanide ISA 51 VG is provided with the current IB (Version 3291/GB/03/June 2019).

6. Study Procedures and Examination Method

This study will consist of the following consecutive phases: Study entry, vaccination/treatment and follow-up. Time-points and trial procedures are listed in Table 1.

6.1. Study Entry

6.1.1. Volunteer's Informed Consent

Subjects are informed both in writing and verbally by the investigator before any study-specific procedure is performed. Each volunteer will be informed about the modalities of the clinical study in accordance with the provided volunteer information. The volunteer is given sufficient time (≥ 24 h) to consider participation in the clinical trial and to ask for additional advise if needed. Informed consent from the volunteer will be obtained using a form approved by the responsible EC. The volunteer and informing investigator must each personally date and sign the informed consent form containing an integrated declaration on data privacy protection. The original signed document will be part of the investigator's site file and retained with it, a copy including the insurance policy of the trial will be handed to the volunteer. The informed consent process is documented in the volunteer records.

6.1.2. Screening

Screening will be performed within *one* week (7 days) prior to the administration of the CoVac-1 vaccine. After having signed the informed consent form, volunteers will undergo all assessments listed below:

- Demographics
- Medical history
- Enrolment
- Vital signs
- Physical examination
- Concomitant medications
- Hematology (local lab)
- Blood chemistry and coagulation (local lab)
- Urine analysis (local lab)
- Immunoglobulins/Immunophenotype (local lab), approximately 10 ml blood
- Testing for previous or current SARS-CoV-2 infection: 5ml serum blood will be drawn for antibody testing and a nose/throat swab* will be performed.
- HBV, HCV, HIV-1, (local lab)

- Pregnancy test

* If screening takes more than 48 hours, throat/nose swab for SARS-CoV-2 infection must be repeated. The vaccine can only be applied if a negative SARS-CoV-2 PCR test is available on the day of vaccination not older than 48 hours.

The investigator will review all information obtained from the screening procedures via an eligibility form. The investigator will confirm, in writing, whether the subject fulfils all criteria for eligibility. Volunteers who fulfil all the inclusion criteria and none of the exclusion criteria will be eligible to participate in the trial. Screening failures, i.e. screened volunteers not in compliance with all criteria, are to be excluded and the reason will be recorded in the volunteer records. Information of volunteer's trial participation can be provided to the volunteer’s general practitioner if the volunteer agrees.

6.1.3. Enrolment

A volunteer is considered for screening when he or she has signed the Informed Consent form.

In case of confirmation of volunteer’s eligibility (volunteers must meet all inclusion criteria and must not meet any exclusion criteria), volunteer will be registered under a specific Vol. ID on a subjects log kept at the trial site. Only these volunteers are enrolled in the study, all others are assessed as screening failures.

The study is open-label.

6.1.4. Randomisation

No randomisation will be done in this clinical trial.

6.1.5. Concomitant Medication and Treatments

Relevant additional medications and treatments administered to the subjects on entry to the trial or at any time during the trial are regarded as concomitant medications and treatments and must be documented on the appropriate pages of the CRF.

6.1.6. Permitted Prior and Concomitant Medications and Treatments

The following concomitant medications and treatments are permitted during the trial.

Part I: No concomitant medication, apart from contraception for FCBP.

Part II : Any concomitant medication (already applied at screening) for e.g. other diseases are allowed except for medications stated in section 6.1.7.

6.1.7. Prohibited Prior and Concomitant Medications and Treatments

The following concomitant medications and treatments are prohibited during the trial:

- Immunosuppressive agents apart from (≤ 10 mg prednisolone or equivalent)
- During the trial, other vaccinations or non-urgent medical interventions are prohibited. Initiation of new medications, regardless of indication must be discussed with the investigator and must be noted on the participant’s record.

6.1.8. Contraception

Within this study, all FCBP must have a negative pregnancy test ≤ 7 days prior initiation of study treatment. A FCBP is defined as any female who does not meet the criteria of non-childbearing potential. These are as follows:

- documented hysterectomy, bilateral oophorectomy (ovarectomy), or bilateral tubal ligation
- post-menopausal (a practical definition accepts menopause ≥ 1 year without menses with an appropriate clinical profile, e.g. age > 45 years in the absence of hormone replacement therapy (HRT). In questionable cases, the subject must have a follicle stimulating hormone (FSH) value > 40 mIU/ml and an estradiol value < 40pg/ml.

Sexually active men and women of child-bearing potential must use two methods of reliable contraception including one highly effective (Pearl Index < 1) and one additional effective (barrier) method as described below maintained for up to 3 months after the last dose of study therapy.

The following contraceptive methods with a Pearl Index < 1 are regarded as highly-effective:

- oral hormonal contraception (‘pill’)

Please note: in case that its efficacy is impaired during the trial, e.g. due to vomiting and diarrhoea, additional/other methods as listed below are required to assure adequate safety

- dermal hormonal contraception/contraceptive plaster
- vaginal hormonal contraception (NuvaRing®)
- long-acting injectable contraceptives/implants that release progesterone (Implanon®)
- tubal ligation (female sterilization)
- intrauterine devices that release hormones (hormone spiral)
- double barrier methods
- partner’s vasectomy

Additional effective (barrier) methods are:

- male condom
- diaphragm/cervical cap

The following contraceptive methods are not regarded as safe: condom plus spermicide, simple barrier methods (vaginal pessaries, condom, female condoms), copper spirals, rhythm/basal temperature method and withdrawal method (coitus interruptus).

6.2. Vaccination Phase

Vaccination phase begins as soon as possible (within 7 days) after screening and confirmation of patient's eligibility. If screening takes more than 48 hours, throat/nose swab for SARS-CoV-2 infection must be repeated. The vaccine can only be applied if a negative SARS-CoV-2 PCR test is available on the day of vaccination not older than 48 hours.

Peptide vaccines should be injected into the skin at the lower part of the abdomen of the patients. The site of vaccination (right or left) will be determined by the investigator and documented.

To minimize the risk for severe and unexpected side effects for subjects included in the study, all participants will be monitored for at least two hours after vaccination, including close monitoring of heart rate, blood pressure, temperature, oxygen saturation and subjective well-being. Each monitoring unit must be equipped with a crash cart and an intensive care team should be on standby.

Treatment and monitoring of the first volunteer are performed in an in-patient setting with access to intensive care for 24h. Close monitoring (every 30 minutes vital parameters) will be performed for the first four hours after vaccination. Thereafter, monitoring is performed at hourly intervals until 6 hours after vaccination. Thereafter every 3 hours until 24 hours after application of the vaccine.

6.2.1. Visit 1 (Vaccination) (Day 1)

- Signs/symptoms, baseline
- Vital signs, close monitoring after vaccination (blood pressure, temperature, heart rate and oxygen saturation every 30 minutes for at least 2 hours)
- Physical examination, baseline
- Assessment of concomitant medications

- AE assessment
- Hematology and blood chemistry (local lab), approximately 10 ml blood
- Vaccination (section 5.7)
- T-cell response, baseline obtained before vaccination, approximately 60 ml blood
- Serological response, baseline obtained before vaccination, approximately 15 ml blood

6.2.2. Visit 2 (Day 7 +/- 1)

- Signs/symptoms, as assessed on volunteer's diary and visit
- Vital signs,
- Physical examination, including investigation of vaccination side
- Assessment of concomitant medications
- AE assessment
- Hematology and blood chemistry (local lab), approximately 10 ml blood
- T-cell response, approximately 60 ml blood
- Serological response, approximately 15 ml blood

6.2.3. Visit 3 (Day 14 +/- 1)

- Signs/symptoms, as assessed on volunteer's diary and visit
- Vital signs,
- Physical examination, including investigation of vaccination side
- Assessment of concomitant medications
- AE assessment
- Hematology and blood chemistry (local lab), approximately 10 ml blood
- T-cell response, approximately 60 ml blood
- Serological response, approximately 15 ml blood

6.2.4. Visit 4 (Interim safety) (Day 28 +/- 2)

- Signs/symptoms, as assessed on volunteer's diary and visit
- Vital signs,
- Physical examination, including investigation of vaccination side

- Assessment of concomitant medications
- AE assessment
- Hematology and blood chemistry (local lab), approximately 10 ml blood
- T-cell response, approximately 60 ml blood
- Serological response, approximately 15 ml blood

6.2.5. Visit 5 (End of Safety follow-up = EOSf)

- Signs/symptoms, as assessed on volunteer's diary and visit
- Vital signs,
- Physical examination, including investigation of vaccination side
- Assessment of concomitant medications
- AE assessments
- Hematology and blood chemistry (local lab), approximately 10 ml blood
- T- cell response, approximately 60 ml blood
- Serological response, approximately 15 ml blood

6.2.6. Visit 6-7 (Follow-up) (Month 3 and 6 +/- 7 days)

- Medical history, anamnestic evaluation of SARS-CoV-2 specific symptoms
- T-cell response, approximately 60 ml blood
- Serological response, approximately 15 ml blood

6.2.7. Volunteer's diary/card

Each patient included in the P-pVac-SARS-CoV-2 study will receive a volunteer's card, which states that he/she is participating in the study (Appendix 13.4). This will also include a 24h emergency contact number. Furthermore, each patient will be provided with a volunteer's diary to note their symptoms daily (Appendix 13.3)

6.2.8. Unscheduled Visit

Subjects may contact the investigator at any time for an unscheduled phone or on-site visit should they experience clinical symptoms or signs following injection. At all unscheduled visits, the following minimum assessment will be performed: Questions concerning the history of the present illness as well as the subject's general health and lifestyle. Findings resulting in (S)AEs

will be documented and reported as indicated. All other symptoms/signs will be reported on the next scheduled visit on eCRF.

Upon occurrence of symptoms characteristic of SARS-CoV-2 (i. e. cough, fever (cut-off >39°C), loss of taste and smell, limb pain) at any time until day 56, subjects are supposed to get in touch with the investigator. Investigator will initiate SARS-CoV-2 testing for the volunteer (nose or mouth swab followed by PCR per institutional guidelines). If the test is positive, patients should be treated per investigators discretion. Positive results must be recorded as an AESI (section 9.1.4). Negative results will be followed by a second testing ≥ 24h later. Only upon the second negative test, patients are considered negative, all others must be reported as positive.

If participants are positively tested for SARS-CoV-2, all accompanying symptoms and treatments (e.g. hospitalisation, ICU) are recorded

Medically attended AEs and all SAEs will be recorded, and concomitant medication or vaccination will be noted. After identifying the history of the present illness and performing corresponding exams or laboratory tests, the investigator will decide on the best course of treatment according to standard medical practice.

6.3. Assessment of Efficacy

6.3.1. Efficacy Parameters

<u>Immunological Efficacy:</u>

Induction of SARS-CoV-2-specific $CD8^+$ and $CD4^+$ T cells is evaluated using:

- IFN-γ ELISPOT
- Intracellular cytokine staining for TNF and IFN-γ

Induction of SARS-CoV-2 specific antibodies:

- ELISA

6.3.2. Methods and Timing for Assessing, Recording, and Analysing of Efficacy Parameters

<u>Immunological Efficacy:</u>

Serial measurements of immunological efficacy will be performed prior to peptide vaccination (V1), and V2, V3, V4, at the end of study visit and the follow up visits as outlined in table 1. All scheduled visits have a ± 1 day window unless otherwise stated. 75ml peripheral blood (60 ml Na^+-heparin and 15 ml serum) for immunological assays will be obtained prior to vaccination as indicated in table 1. Immunological assays will be performed in the Department of Immunology or the Immunopathological Laboratory, Department of Internal Medicine, University Hospital Tuebingen based on standard SOPs.

Amplification of SARS-COV-2-specific T cells:

PBMCs from volunteers are pulsed the respective peptide and cultured for 12 days adding IL-2 on days 3, 5, and 7. Peptide stimulated PBMCs are analyzed by enzyme-linked immunospot (ELISPOT) assay on day 12 or by flow cytometry-based tetramer and intracellular cytokine staining as described below.

IFN-γ ELISPOT assay

IFN-γ ELISPOT assays are carried out as described previously.[114] In brief, 96-well nitrocellulose plates are coated with anti-IFN-γ. Plates are blocked and PBMCs (*ex vivo* or after T-cell amplification as described above) are distributed to the wells and re-stimulated with HLA class II peptides. Cytokine staining is performed after incubation period. Analysis is performed according to manufacturer’s instructions. Spots are counted using an Immunospot analyzer (according to the cancer immunoguiding program (CIP) guidelines).[115]

To differ between vaccine induced and natural T-cell induction by SARS-CoV-2 infection we will included, beside the T-cell epitopes included in the CoVac-1 vaccine, additional SARS-CoV-2 T-cell epitopes defined in our preclincial work in the peptide readout [76].

Cellular conversion rate (CCR) is calculated by dividing the number of volunteers with an immune response by the number of tested participants to a time point (Visit 2, 3, 4 and 5). A volunteer is considered as having developed an immune response due to immunization if *ex vivo* IFN-γ ELISPOT assay is positive (as described above) and the spot count is at least 2-fold higher than the baseline assay (Visit 1).

Intracellular IFN-γ and TNF staining

The frequency and functionality of peptide-specific $CD8^+$ T cells is analyzed by intracellular IFN-γ or TNF staining as described previously.[114 116] PBMCs are pulsed with individual peptide and incubated in the presence of Brefeldin A and GolgiStop. Cells are labeled using

Cytofix/Cytoperm, CD8, CD4, TNF and IFN-γ coupled to fluorochromes. Samples are evaluated on a FACS analyzer.

Enzyme-linked immunosorbent assay (ELISA)

To identify SARS-CoV-2 antibody responses induced by the vaccine, ELISA assays will be performed using serum samples (15 ml serum tube) obtained at the time points described in Table 1. Specific antibodies against the seven SARS-CoV-2 T-cell epitopes will be assessed by ELISA assay at the Department of Immunology, Tübingen. To differ between vaccine induced antibody response additional standard Elecsys® Anti-SARS-CoV-2 assay supplied by F. Hoffmann-La Roche AG, Basel, Switzerland or ADVIA Centaur SARS-CoV-2 Total (COV2T) (Siemens Healthcare Diagnostics GmbH) will be performed at central laboratory of the University Hospital Tuebingen.

Occurrence or relevant (≥2-fold) increase of SARS-CoV-2 specific IgG antibodies compared to baseline are considered as positive.

In the unlikely event of antibody induction by the CoVaC-1 vaccine, neutralization capacity of antibodies will be assessed by SARS-CoV-2 Pseudovirus Neutralization Assay (CD, Creative Diagnostics®)

6.4. Assessment of Safety

6.4.1. Safety parameters

(Serious) Adverse Events (see section 9)

- Vital signs: pulse, blood pressure, temperature, and weight
- Physical examination including inspection of the vaccination side
- Clinical laboratory evaluations:
  Hematology: white blood cell (WBC), hemoglobin (Hb), platelet count, absolute neutrophil count (ANC), absolute lymphocyte count (ALC)
  Chemistry: AP, total bilirubin, AST/ SGOT, ALT/ SGPT, LDH, and uric acid, CRP, sodium, potassium, calcium, blood urea nitrogen, creatinine, glucose, C-reactive protein
- Concomitant medications
- (S)AEs by NCI CTCAE Version 5.0 and as in appendix 14.5

6.4.2. Methods and Timing for Assessing, Recording, and Analysing Safety Parameters

Serial measurements of safety will be performed at screening and at scheduled intervals throughout the duration of the study as outlined in table 1. All scheduled visits have a ± 1 day window unless otherwise stated. Abnormalities will be captured as protocol deviations. Lab abnormalities grade 1-2 are only considered AE if they fulfill one of the following criteria:

- Accompanied by clinical symptoms.
- Requiring a change in concomitant therapy (e.g. addition or change in a concomitant medication, therapy or treatment).

All Grade 3-4 laboratory abnormalities fulfilling the criteria for an SAE will be reported as SAEs and will be recorded on the AE pages of the CRF; however, those that are not deemed by the investigator to be part of a diagnosis or syndrome will not be reported to the Health Authorities in an expedited manner. Cause of death is to be recorded in the CRF and the subject's medical record.

6.5. Vaccination holding rules

Safety holding rules for each subject will apply throughout the study period until interim safety analysis (V4). Vaccination of further study subjects in the consecutive study phase will not occur until a safety review has been conducted by the DSMB and only by approval a holding rule can be resolved. If a holding rule is activated, the PI will inform the Sponsor within 48 hours. The Sponsor will inform the responsible authorities (PEI and EC).

If the DSMB permits the resumption of injections, a formal request with pertinent data must be submitted to ECs and PEI. The discontinuation of a holding rule should be communicated to all entities in the same manner and timeframe as described above.

The DSMB safety review will consider:

- The relationship of the AE or SAE to the vaccine or other possible causes of the event.
- If appropriate, additional screening or laboratory testing for other volunteers to identify those who may develop similar symptoms and alterations to the current informed consent form will be discussed.

All injected volunteers will be followed for safety until resolution or stabilization (if determined to be chronic sequelae) of their AE.

<u>The holding rules are as follow:</u>

- Solicited local ADRs: If more than 30% of injections are followed by Grade ≥3 solicited swelling or pain or Grade 4 redness (first occurrence at any time after vaccination)and persisting at Grade 3 (swelling or pain)/4 (redness) for > 48 h to maximum 72 hours depending upon symptom severity and kinetics.
- Solicited systemic AEs: If more than 25% of injections are followed by Grade 3 solicited systemic AE beginning within 3 days after study injection (day of injection and 2 subsequent days) and persisting at Grade ≥ 3 for > 48 h to maximum 72 hours depending upon symptom severity and kinetics.
- Unsolicited AEs: If more than 25% of volunteers develop a Grade ≥ 3 unsolicited AE (including laboratory AE and physical observations) that is considered probably or definitely related to injection and persists at Grade 3 for > 48 to maximum 72 hours depending upon symptom severity and kinetics.
- A suspected unexpected serious adverse drug reaction (SUSAR) occurs that is life-threatening or results in death.

6.6. Premature termination of clinical trial for a trial subject

Reasons for premature termination of trial for an individual trial subject are:

1. Death
2. Withdrawal of consent
3. Volunteer lost to follow-up
4. For women, in case of pregnancy

The PI decides about withdrawal of subjects from trial treatment in case of occurrence of criteria mentioned above. In all cases, the reason for withdrawal must be recorded in the CRF and in the subject’s medical records. In case of withdrawal of a subject at his/ her own request, the reason should be determined and documented.

All examinations scheduled for the last trial day will be performed and documented as far as possible, subject to consent of the volunteer. Subjects will enter the regular follow-up of the trial, unless the subject has withdrawn his/her consent to any further study-related procedure. If a subject is withdrawn from all trial-related procedures (including follow-up visits) e.g. at his/her own request, this will not result in any disadvantages for the volunteer.

All ongoing Adverse Events (AEs)/ Serious Adverse Events (SAEs) of withdrawn subjects have to be followed-up until no more signs and symptoms are verifiable or the subject is on stable condition.

Premature termination should be avoided. In case of a premature termination of study, reasons/circumstances and if applicable the final status have to be documented. If volunteers do not withdraw the consent for further follow-up, they should be followed-up as planned.

6.7. Premature closure of a trial site

Premature closure of a trial site has to be considered if:

- The recruitment rate is not sufficient
- The conduct of the study is not compliant with the protocol or the legal regulations, or
- The data quality is not sufficient

The premature closure of a site will be decided by the sponsor.

Site principal investigators may terminate his/her participation in the study. If this occurs they should provide a written statement of the reasons for terminating participation and must provide the sponsor with all available and up-to-date study data.

The sponsor may also decide to terminate participation of an investigator or study centre for the following reasons:

- Breach of agreement
- Serious non-compliance to protocol or the legal regulations
- Insufficient volunteer recruitment

If a participating center closes, or is closed, prior to termination of the whole trial, the sponsor expects that data from volunteers already entered into the trial will be reported as per protocol. Details on further treatment and follow-up of volunteers on study have to be discussed with the site principal investigator.

6.8. Premature termination of the trial

The trial may be prematurely terminated, if in the opinion of the sponsor and coordinating investigator, there is sufficient reasonable cause. Written notification documenting the reason for study termination will be provided to the investigators.

In case of the following situations a premature termination of the trial has to be considered:

- Observation of one SAE associated with administration of CoVac-1 (Statistical Stopping rule of the study)
- Serious adverse drug reactions / not justifiable toxicity
- Substantial changes in risk-benefit considerations
- New insights from other trials
- Insufficient efficacy
- Insufficient recruitment rate

The DSMB will monitor the study conduct and the safety aspects of the trial on a regular basis, and will give recommendations to the coordinating investigator/ the sponsor whether to stop the trial or to change the trial protocol. The sponsor will then decide on the actions to be taken. According to the German drug law (§42a), the trial may be suspended or prematurely terminated by decision of the competent authority (PEI).

6.9. Follow Up

Volunteers will be followed for up to 4 months after EOSf. Thereafter patients may be contacted by phone call/e-mail to assess infection with SARS-CoV-2.

6.10. End of Study for Subjects

The end of Study for a subject enrolled in this trial is defined as the last study visit.

7. Quality control and Quality assurance

7.1. Risk-based approach

During protocol development, processes and data that are critical to ensure human subject protection and the reliability of trial results were identified.

The identified risks were evaluated against existing risk controls by considering:

- The likelihood of errors occurring
- The extent to which such errors would be detectable
- The impact of such errors on human subject protection and reliability of trial results.

In case of unacceptable risks, risk reduction activities were defined and incorporated e.g. in the protocol, monitoring plan and agreements.

Results will be communicated to those who are involved in or affected by such activities.

The sponsor periodically reviews risk control measures to ascertain whether the implemented activities remain effective and relevant, taking into account emerging knowledge and experience.

7.2. Monitoring

Monitoring for this study is provided by the *Zentrum für Klinische Studien Tuebingen (ZKS Tuebingen).* The monitoring will be conducted according to *ZKS Tuebingen Internal Standard Operating Procedures (SOPs)* and a dedicated monitoring manual for the study. The monitoring timelines include, for all centres, initiation visit, regular monitor visits during the course of the trial as well as a close out visit. Usually, monitoring will end with the last visit after full documentation of the last volunteer enrolled (close out visit). All investigators agree that the monitors regularly visit the trial site, assure that the monitors will receive appropriate support in their activities and will have access to all trial-related documents.
The aims of the monitoring visits are as follows:

- Check informed consent documents
- Monitor trial subject safety (occurrence and documentation/reporting of Serious Adverse Events (SAEs) and Adverse Events (AEs)).
- Check completeness and accuracy of entries on the CRFs.

- Validate entries on the CRFs against those in the source documents (source data verification (SDV)).
- Check the Drug Account
- Check the storage conditions of the IMP
- Evaluate the progress of the trial
- Evaluate compliance with the trial protocol
- Assess whether the trial is being performed according to GCP at the trial site
- Discuss with the investigator aspects of trial conduct and any deficiencies found
- A monitoring visit report is prepared for each visit describing the progress of the clinical trial and any problems

7.3. Audits/ Inspections

In addition to the monitoring activities, audits can be conducted by the sponsor or assigned auditors. These audits may include checking the whole course of the study, documentation, trial centre, investigators and the monitor.

The competent regulatory authorities may also conduct inspections.

With his/her participation in the study, the investigator agrees to support the activities of the auditor/inspector, provide her/him with direct access to the source documents, study documentation and give her/him the opportunity to audit/inspect the study site, laboratory facilities, storage of the investigational product, etc.

7.4. Documentation: Collection, Handling, Storage and Archiving of Data

7.4.1. Case Report Form

The trial Case Report Form (CRF) is the primary data collection instrument for the trial. All data requested on the CRF must be recorded. All missing data must be explained.

For this project, electronic Case Report Forms (eCRFs) will be used. The Clinical Data Management System [secuTrial “SecuTrial”] will be used for data capture, processing and storage of study data. Data entry is performed at the investigational site by clinical staff after having received training and a user manual for the electronic CRF. Training and the user manual will detail procedures to be followed in case of technical problems. Queries resulting from edit checks and/or data verification procedures will be posted electronically in the eCRF.

The Clinical Trial Data Management System (CDMS) is validated and changes are tracked via an audit trail.

The correctness of entries in eCRFs will be confirmed by dated signature of an authorized investigator. The Principal investigator is responsible for ensuring that all sections of the eCRF are completed correctly and that entries can be verified against source data. The Principal investigator has to verify the eCRFs via dated electronic signature after completion of the eCRF.

7.4.2. Source Data

Source data is all information, original records of clinical findings, observations or other activities in a clinical trial necessary for the reconstruction and evaluation of the trial. Source data are contained in source documents. Examples of these original documents and data records include: hospital records, clinical and office charts, laboratory notes, memoranda, volunteers' diaries or evaluation checklists, pharmacy dispensing records, recorded data from automated instruments, copies or transcriptions certified after verification as being accurate and complete, x-rays, CTs, MRIs, ultrasound reports, volunteer files, and records kept at the pharmacy, at the laboratories, and at medico-technical departments involved in the clinical trial.

7.4.3. Data Handling

Authorized clinical staff at the investigational site will enter the data into the eCRF using an access controlled, audit-trailed, ICH/GCP compliant, validated system. Entered data will be subjected to plausibility checks directly implemented in the eCRF, monitoring and medical review. Implausible or missing data will be queried. Database lock will be performed after completion of data entry, data cleaning and a final data review.

7.4.4. Preparation/Handling/Storage/Accountability of biological samples

Biological samples collected under this protocol may be used in accordance with the study informed consent form to conduct protocol related safety and immunogenicity evaluations, exploratory laboratory evaluations related to the SARS-CoV-2 infection the vaccine was designed to prevent, exploratory laboratory evaluations related to vaccine research in general and for research assay validation. All biological samples obtained within the study will be identified solely by means of the individual identification code (Patient ID). Samples will be

either processed directly or for PBMC and serum samples for immunogenicity analysis stored until further analyses. Storage of biological samples on a computer will be done in accordance with local data protection law and will be handled in strictest confidence. For protection of these data, organizational procedures are implemented to prevent distribution of data to unauthorized persons. The appropriate regulations of local data legislation will be fulfilled in its entirety. Samples are stored at the Department of Immunology, Tuebingen. Only investigators or their designees will have access to the samples and corresponding data. Sample tracking and preparation will be performed according to established standard operating procedures. The biological samples will be destroyed at the latest 30 years after the end of the study. If a study subject withdraws consent to participate in the study all samples taken and identifiable are destroyed without prior analysis if requested.

7.4.5. Handling of missing data and drop outs

Missing values will be predicted based on plausible assumptions that account for the uncertainty due to missing data. For patients with unknown status for the primary endpoint, i.e. a volunteer without complete follow-up and without any SAE until the last known study site contact, a detailed report on the course should be presented by the investigator and discussed concerning probable unknown SAEs and the reasons for drop-out. If substantial reason will be found that the person could have experienced a SAE, this will be interpreted as failure and the recruitment should be stopped accordingly. Otherwise the safety of the person will be interpreted as success, i.e. the subject will be interpreted to have not experienced a SAE. If this decision cannot be precisely concluded, patient will be considered as drop-out. All missing data or inconsistencies will be resolved by the responsible investigator.

7.4.6. Storage and Archiving of Data

According to the EU Clinical Trial Regulation 536/2014 all essential trial documents (e.g. CRF) will be archived for at least 25 years after the trial termination. The investigator(s) will archive all trial data (source data and Investigator Site File (ISF) including subject identification list and relevant correspondence) according to the Guideline ICH GCP (E6) and to local law or regulations.

8. Statistical Analyses

8.1. Study Population Definition

8.1.1. Sample Size and Power Consideration

In this phase I study the safety/toxicity of one vaccination will be investigated. For this purpose, it will be investigated whether the incidence of severe adverse events (SAE) associated with administration of CoVac-1 exceeds a predetermined rate of 5% (= P1 = alternative hypothesis) in the whole study population. Safety of the CoVac-1 vaccine is shown if no SAE (= P0 = null hypothesis) occurs in the study population. An evaluable sample size of 33 achieves 81.6% power to detect a difference (P1-P0) of 0.0499 using a one-sided exact test based on the binomial distribution with a target significance level of 0.05. The actual significance level achieved by this test is 0.003. These results assume that the population proportion under the null hypotheses (P0) is 0.0001. Assuming a dropout rate of 7.5% (percentage of subjects that are expected to be lost at random during the course of the study and for whom no response data concerning existence of SAE will be collected, i.e. will be treated as "missing") the total number of 36 subjects should be enrolled in the study in order to end up with 33 evaluable subjects. Sample size computed using PASS 2020 (NCSS, LLC, Kaysville, Utah, USA).

8.2. Analysis Primary Variables

The occurrence of critical events (SAE) associated with administration of CoVac-1 should be reported to the Sponsor (section 9.3.1) and documented immediately in the eCRF (within 48h). The statistical center will evaluate the occurrence of critical events using automatized alerts of the e(CRF) on a daily basis and distribute this information to the Sponsor/DSMB. If one critical event will be observed, the formal statistical stopping rule of the study is reached and no further recruitment is adequate. Otherwise the safety of the procedure will be accepted, if no out of 33 volunteers will experience a critical event.

No further statistical tests with confirmatory aim are planned.

8.3. Analysis Secondary Variables

<u>Safety</u>

The statistical analysis of the secondary endpoint will be done in a descriptive manner. No statistical tests with confirmatory aim are planned. The toxicity and safety will be described by absolute and relative frequencies using CTCAE V5.0-scoring.

<u>Efficacy</u>

The rate of patients with induction of peptide-specific T-cell responses within a maximum of 56 days after vaccination will be the secondary endpoint for efficacy. T-cell responses will be assessed as described in section 6.3.1

The rate of patients with induction of antibody responses within a maximum of 56 days after vaccination will be the secondary endpoint for efficacy. The antibody response will be assessed as described in section 6.3.1

8.4. Subgroup Analysis

Exploratory subgroup analyses are planned for each part (I and II) regarding primary and secondary endpoints.

8.5. Interim Analysis

The primary endpoint will be evaluated in a sequential manner after every consecutive included volunteer has reached day 28. No further formal interim efficacy analysis will be performed during the conduct of the study.

8.6. Stopping Rules

The pre-defined stopping rule for the study is reached if one critical event (SAE as defined in section 9.1.5) associated with administration of CoVac-1 will be observed in the study population resp. if the first critical event will be observed.

The sponsor has the right to terminate the trial prematurely if there are any relevant medical or ethical concerns, or for reasonable administrative reasons. If such action is taken, the reasons for terminating the trial have to be documented in detail. All volunteers who are not considered end of study must undergo a final examination, which must be documented.

Criteria for termination of the study as a whole are:

- An unacceptable profile or incidence rate of adverse events/ adverse events of special interest revealed in this or any other study in which at least one of the investigational products of this trial is administered.
- Significant number of cases of death associated with the study treatment.

- Any other factor that in the view of the sponsor constitutes an adequate reason for terminating the study as a whole.

The Sponsor has to be informed without delay if any investigator has ethical concerns.

8.7. Biometric Report

The biometric report lies within the responsibility of the biostatistician of the clinical trial. The sponsor has to make every effort to acquire a complete data set for statistical analysis. The trial report has to be completed within a reasonable time.

9. Safety

9.1. Definition of Adverse Events and Side Effects

9.1.1. Adverse Events

Any untoward clinical relevant medical occurrence in a volunteer or clinical investigation subject to whom a pharmaceutical product had been administered and which does not necessarily have a causal relationship with this treatment. An AE can therefore be any clinical relevant unfavorable and unintended sign (including an abnormal laboratory finding), clinical relevant symptom, or disease temporally associated with the use of a medicinal (investigational) product, whether or not related to the medicinal (investigational) product.

An AE may be:

- New clinical relevant symptoms/ medical conditions
- New clinical relevant diagnosis
- Clinical relevant changes of laboratory parameters
- Diseases and medical consequences of an accident
- Worsening of medical conditions/ diseases existing before clinical trial start
- Recurrence of disease
- Clinical relevant increase of frequency or intensity of episodical diseases

A pre-existing disease or symptom will not be considered an AE unless there will be an untoward change in its intensity, frequency or quality. This change will be documented by the investigator.

In general, abnormal laboratory findings or clinical events without clinical significance (based on the investigator´s judgement) should not be recorded as AEs.

Surgical procedures themselves are not AEs; they are therapeutic measures for conditions that require surgery. The condition for which the surgery is required may be an AE. Planned surgical measures permitted by the clinical trial protocol and the condition(s) leading to these measures are not AEs, if the condition leading to the measure was present prior to inclusion into the trial.

AEs are classified as "non-serious" or "serious".

9.1.2. Adverse Drug Reaction

An Adverse Drug Reaction (adverse reaction: undesirable effect) is a response to a medicinal product which is noxious and unintended. Adverse reactions may arise from use of the product within or outside terms of the marketing authorisation or from occupational exposure. Use

outside the marketing authorisation includes off-label use, overdose, misuse, abuse and medication errors.

An unexpected Adverse Drug Reaction (ADR) is a reaction which nature or severity is not consistent with the applicable product information available for the IMP.

Expected ADRs arelisted in the appropriate reference documents, e.g. Investigator's Brochures; and below:

A solicited AE/ADR is a predetermined event, which may reflect safety concerns related to the investigational product and is, at least for the local solicited AEs, expected. The solicited ADR/AEs (local and systemic) for this study include:

Local solicited ADRs:

- Swelling at site of injection
- Erythema at site of injection
- Pain or itching at site of injection
- Formation of granuloma at the injection site
- Superficial skin ulceration

Systemic solicited AEs:

- Fever
- Chills
- Myalgia (described to the subject as generalized muscle aches)
- Arthralgia (described to the subject as generalized joint aches)
- Fatigue
- Headache
- Gastrointestinal symptoms (loss of appetite, nausea, vomiting, abdominal pain, and/or diarrhoea)

A grading for severity of ADRs can be found in appendix 14.5 as guidance.

9.1.3. Expectedness

An 'unexpected' adverse event is one the nature or severity of which is not consistent with the applicable product information, e.g. Investigator's Brochure (IB). Furthermore, reports which

add significant information on specificity or severity of a known adverse reaction are counted as ‘unexpected’ events.

9.1.4. AESI (adverse events of special interest)

An adverse event of special interest (AESI), serious or non-serious, is one of scientific and medical concern specific to the sponsor’s product, for which ongoing monitoring and rapid communication (≤ 48 hours) by the investigator to the sponsor can be appropriate. Such an event might warrant further investigation in order to characterize and understand it. Depending on the nature of the event, rapid communication by the trial sponsor to other parties (e.g. regulators) might also be warranted (adapted from CIOMS 2005).

In case of the CoVac-1 vaccine in this study, AESIs include proven SARS-CoV-2 infection and potential immune mediated diseases (pIMDs, see Appendix 14.6)[117]. Instructions for management are provided in section 6.3.

With regard to trial schedule and AESI occurrence, AESIs constitute:

- Novel proven (PCR-based) SARS-CoV-2 infection accompanied by symptoms
- Novel proven (PCR-based) SARS-CoV-2 positivity without symptoms
- Novel potential immune mediated diseases (pIMD) according the listed diseases in Appendix 14.6
- Formation of granuloma at the injection site

AESIs are always to be addressed as part of the patient safety report to the DSMB (section 1.4), also non-occurrence will be mentioned. Depending on the decision of DSMB, the vaccination of further volunteers will be permanently stopped.

9.1.5. Serious Adverse Event and Serous Adverse Reaction

AEs are classified as "non-serious" or "serious".

A serious adverse event (SAE) is one that at any dose:

- Results in death.
- Is life-threatening (the term life-threatening refers to an event in which the subject was at risk of death at the time of event and not to an event which hypothetically might have caused death if it was more severe).
- Requires subject hospitalization or prolongation of existing hospitalization.
- Results in persistent or significant disability/ incapacity.
- Causes a congenital anomaly / birth defect.
- Is medically significant (e.g. suspected transmission of an infectious agent via medicinal product). Moreover, there are other situations - such as

important medical events that may not be immediately life threatening or result in death or hospitalization, but may jeopardize the patient or may require intervention to prevent one of the other outcomes listed above.Important medical event [ICH E2A; EMA/155528/2018]: Examples of such events are intensive treatment in an emergency room or at home for allergic bronchospasm; blood dyscrasias or convulsions that do not result in hospitalisation; development of drug dependency or drug abuse (Important medical event terms list (MedDRA ≥ version 23.0).

9.2. Period of Observation

For the purpose of this trial, the period of observation for collection of AEs extends from the time of administration of the IMP until Visit 5.

All AEs that occur in the course of a clinical trial regardless of the causal relationship must be monitored and followed up until the outcome is known or no more information is achievable.

9.3. Documentation and Reporting of Adverse Events

9.3.1. Documentation and Reporting of Adverse Events by the Investigator

The investigator must document all AEs that occur during the observation period set in this protocol on the pages provided in the case report form. Additional instructions may be provided in the investigator file and in the case report form itself. The following approach will be taken for documentation:

All AEs (whether serious or non-serious) must be documented on the “adverse event” page of the eCRF.

If the AE is serious, the investigator must complete, in addition to the “adverse event” page in the case report form, a “serious adverse event report form” at the time when the SAE is detected. The investigator will document the date when he/she or any employee was first aware of the report. The initial report must be as concise as possible, including reported terms according to “Common Terminology Criteria for Adverse Events (CTCAE)-List” (one term per event), details of the current illness and (S) AE, severity, serious criteria as well as an assessment of the causal relationship between the event and the trial medication.

SAE reports (initial and follow-up reports), even if they are incomplete, should be send within 24 hours upon receipt to representative of the Sponsor:

[redacted]

9.3.2. Assessment of Severity and Causality

The investigator will also provide an assessment of the severity of the event according to CTCAE criteria (Version 5.0) and causal relationship between the event and each of the investigational products or trial procedures.

AEs and SAEs should be evaluated for severity according to the following scale:

- Grade 1 Mild; asymptomatic or mild symptoms; clinical or diagnostic observations only; intervention not indicated.
- Grade 2 Moderate; minimal, local or noninvasive intervention indicated; limiting age-appropriate instrumental Activities of Daily Living (ADL).
- Grade 3 Severe or medically significant but not immediately life-threatening; hospitalization or prolongation of hospitalization indicated; disabling; limiting self care ADL.
- Grade 4 Life-threatening consequences; urgent intervention indicated.
- Grade 5 Death related to AE.

The investigator must determine the causal relationship between the administration of IMP and the occurrence of an AE/SAE as defined below:

Related: There is a reasonable possibility that the SAE may be related to the IMP (e.g. favorable temporal relationship, positive dechallenge: symptoms are receding when IMP is withdrawn or the dose reduced, positive rechallenge: symptoms are reappearing when the IMP is reintroduced or the full dose is re-administered)

Not Related: There is no reasonable possibility that the SAE is related to the IMP (e.g. there is a plausible alternative cause for the SAE that better explains the occurrence of the SAE)

Outcome of AEs

The outcome of an AE at the time of the last observation will be classified as:

**Recovered/ resolved**	All signs and symptoms of an AE disappeared without any sequels at the time of the last interrogation.
**Recovering/ resolving**	The intensity of signs and symptoms has been diminishing and/ or their clinical pattern has been changing up to the time of the last interrogation in a way typical for its resolution.
**Not recovered/ not resolved**	Signs and symptoms of an AE are mostly unchanged at the time of the last interrogation.

**Recovered/ resolved with sequel**	Actual signs and symptoms of an AE disappeared but there are sequels related to the AE.
**Fatal**	Resulting in death. If there are more than one AE, only the AE leading to death (possibly related) will be characterized as ‘fatal‘.
**Unknown**	The outcome is unknown or implausible and the information cannot be supplemented or verified.

9.3.3. Action taken

No action will be taken with regards to the IMP as the vaccine is applied only once.

9.3.4. Sponsors Assessment of the SAEs

All SAE will be subject to a second assessment by the trial Sponsor or authorized second assessors, e.g. CI.

The second assessor will fill out a ‘Second Assessment Form’ for each SAE containing.

- Event serious yes/no
- Relationship between SAE and IMP/study procedure
- Expectedness of SAE according to the reference document: IB CoVac-1 peptide vaccine V1.0 dated 22.5.2020.
- Benefit / risk assessment for the trial regarding change as a result of SAE.

9.3.5. Follow-up of Initial Report

Information not available at the time of the initial report (e.g. end date for the AE or laboratory values received after the report) must be documented on a “Serious Adverse Event” form with the box “Follow-up” checked under “Report type”.

All volunteers who have AEs, whether considered associated with the use of the investigational products or not, must be monitored to determine the outcome as far as possible. The clinical course of the AE will be followed up according to accepted standards of medical practice even after the end of the period of observation, until a satisfactory explanation is found or the investigator considers it medically justifiable to terminate follow-up. Should the AE result in death, a full pathologist’s report should be supplied, if possible.

The sponsor will identify missing information for each SAE report and will require follow up information in regular intervals from the investigators until all queries are resolved or no further information can be reasonably expected. All responses to queries and supply of additional

information by the investigator should follow the same reporting route and timelines as the initial report.

9.3.6. Exception of reporting

As this is a prophylactic vaccination trial with application of CoVac-1 in healthy adults, no exception of reporting for AEs are made.

9.3.7. Suspected Unexpected Serious Adverse Reaction (SUSAR)

SAEs that are both suspected, i.e. possibly related to IMP, and ‘unexpected’, i.e. the nature and/ or severity of which is not consistent with the applicable product information are to be classified as Suspected Unexpected Serious Adverse Reactions (SUSARs).

In case that either the investigator who primarily reported the SAE, or the second assessor classify the SAE as ‘suspected’ (*i.e. not as "definitely not related to IMP”)* and the SAE is also unexpected, it will be categorized as a SUSAR.

All SUSARs are subject to an expedited reporting to the responsible ethics committee(s), the competent higher federal authority (PEI) and to all participating investigators.

9.3.8. Expedited Reporting to the Regulatory Authorities

<u>Fatal and life-threatening SUSARs</u>
The competent authority (PEI) and the EC responsible must be informed by the Sponsor of all fatal or life-threatening SUSARs. This must be done immediately, at the latest seven calendar days after becoming aware of the minimum criteria for reporting. In all cases, attempts must be made to obtain further relevant information, which must be supplied to the competent authority and the EC in overall charge within a further eight days. Furthermore, if a trial subject dies, this information must be additionally passed on to the EC responsible for the region in which the death occurred.

<u>SUSARs that are not fatal or life-threatening</u>
The authority (PEI) and the EC responsible will be informed without delay by the sponsor or CI of all SUSARs, at the latest within 15 calendar days of becoming aware of the minimum criteria for reporting. Further relevant details will be passed on as soon as possible.
If the information at the time of reporting is incomplete, further information to enable adequate assessment of the case will be requested from the reporter or other available sources.

9.4. Examination and Report of Changes in the Risk to Benefit Ratio

Without delay, and at the latest within 15 days of the decision for the need to do so, the Sponsor / CI will inform the competent authority (PEI), the EC responsible of any events or factors that could result in a review of the risk-benefit ratio of the IMP. These consist especially of:

- Individual reports of expected serious ADRs with an unexpected outcome.
- A clinically relevant increase in the rate of occurrence of expected ADRs.
- SUSARs in trial subjects who have already completed the follow-up period of the clinical trial (”end-of-trial visit”).
- Factors emerging in connection with trial conduct or the development of the IMP that may affect the safety of persons concerned.

9.4.1. Reporting to Data and Safety Monitoring Board

The DSMB will be informed of all safety-relevant events by the Sponsor / CI. An interim safety analysis will be sent to the DSMB after completion of Part I and Part II. The DSMB will decide on trial continuation. Additionally, the DSMB will be informed as soon as a IMP-related SAE/SUSAR occurs or a holding rule is reached. Meetings may be convened as conference calls/Emails as well as in person.

9.4.2. Report to the Investigator

The Sponsor / CI will inform investigators of all SUSARs including all relevant further information within the periods set by the authority.

If new information becomes known that is different from the scientific information given to the investigator, all investigators will be informed of this by the sponsor.

9.5. Interim Safety analysis

Two or more interim safety analyses will be undertaken to guide decision and whether to start recruitment in the consecutive trial parts. Upon completion of a study part, screening will be interrupted until safety approval of DSMB is available. The data to be evaluated by the DSMB will include (report):

- Solicited and unsolicited AEs/ADRs, AESIs and SAEs
- Review and, if necessary, assessment of (S)AE relatedness to IMP

The DSMB decision will be documented in a TMF. The information will be distributed to the study sponsor, the drug manufacturer, all investigators/trial site and the ZKS Department Pharmakovigilanz for information.

The interim safety analysis together with the DSMB decision and first data on immunogenicity of CaVac-1 will be send to the authorities (PEI and ethic committee) as a substantial amendment to gain approval for recruiting in Part II and III of the planned study. After responsible authorities approve the submitted documents, the study will continue enrolment as planned.

9.6. Annual Safety Report

Once a year, the Sponsor / CI will supply a report on the safety of trial subjects with all available relevant information concerning volunteer safety during the reference period to the competent authorities. Information required for this purpose will be made available to the ZKS by the Sponsor/ CI at the reporting date. This report will also be supplied to the responsible ethics committee.
The annual safety report will be compiled according to the corresponding ICH guideline E2F „Development Safety Update Report – DSUR“. The safety report will cover all IMPs used in this study.

9.7. Deviations from the Protocol

Any significant deviation from the protocol will be noted.

The PI or a nominated person will evaluate this deviation from the protocol and will decide on the further course of the trial for the respective subject.

9.8. Reporting of Pregnancy

<u>Maternal exposure</u>

If a volunteers becomes pregnant during the course of the study related procedures have to be discontinued immediately.

The outcome of any conception occurring from the date of the vaccination until 1 month after the application should be followed up and documented.

Pregnancy itself is not regarded as an AE unless there is a suspicion that the investigational product under study may have interfered with the effectiveness of a contraceptive medication.

Congenital abnormalities/birth defects and spontaneous miscarriages should be reported and handled as SAEs. Elective abortions without complications should not be handled as AEs. The outcome of all pregnancies (spontaneous miscarriage, elective termination, ectopic pregnancy, normal birth or congenital abnormality) should be followed up and documented even if the patient was withdrawn from the study.

If any pregnancy or suspected pregnancy occurs in the course of the study, it must be reported to ZKS Tuebingen, department pharmacovigilance (on behalf of sponsor) immediately by fax (fax-number: + 49 (0)7071 29 25205) or mail (zks-pv@med.uni-tuebingen.de) on the Pregnancy Report Form.

All pregnancies should be followed up and documented, even if the patient was withdrawn from the study, until outcome (spontaneous miscarriage, elective termination, ectopic pregnancy, normal birth or congenital abnormality). The outcome must be notified immediately by the investigator to the ZKS Tuebingen, department pharmacovigilance (on behalf of sponsor) within 24 hours of first knowledge as a follow-up to the initial report.

For any event during the pregnancy which meets a seriousness criterion, the Investigator will also follow the procedures for reporting SAEs (complete and send the SAE form to the Sponsor by fax within 24 hours of the Investigator's knowledge of the event).
All neonatal deaths that occur within 30 days of birth should be reported, without regard to causality, as SAEs. In addition, any infant death at any time thereafter that the Investigator suspects is related to the exposure to the study drug/IMPs should also be reported to the Sponsor by facsimile within 24 hours of the Investigators' knowledge of the event.

The same timelines apply when outcome information is available.

If the female is found not to be pregnant, continuation of the volunteer within the study will be determined by the investigator(s).
Paternal exposure

Male patients should refrain from fathering a child or donating sperm during the study and for 3 months following the vaccination.

Pregnancy of the patient’s partners is not considered to be an AE. However, the outcome of all pregnancies (spontaneous miscarriage, elective termination, ectopic pregnancy, normal birth or congenital abnormality) should if possible be followed up and documented.

The outcome of any conception occurring from the date of the first dose until 3 months after the last dose should be followed up and documented.

Information on pregnancy must be collected on the “Pregnancy Reporting Form”. In order for Sponsor or designee to collect any pregnancy surveillance information from the female partner, the female partner must sign an informed consent form for disclosure of this information.

10. Regulatory Consideration

10.1. Ethical Conduct of Clinical Study

10.1.1. Good Clinical Practice, Declaration of Helsinki and legal Provision

The procedures set out in this trial protocol, pertaining to the conduct, evaluation, and documentation of this trial, are designed to ensure that all persons involved in the trial act according to Good Clinical Practice (GCP) and the ethical principles described in the applicable version of the Declaration of Helsinki.

10.2. Subject Information and Informed Consent

Each volunteer will be informed about the modalities of the clinical study in accordance with the provided volunteer informed consent (IC). The volunteer is to be informed both in writing and verbally by the investigator before any study-specific procedure is performed. The volunteer must be given sufficient time to decide whether to participate in this comparative study and to ask questions concerning this trial. It must also be made clear to the volunteer that he / she can withdraw from the study at any time without giving reasons and that he / she will not be in any way disadvantaged for this. The subject must give consent in writing. The volunteer and informing physician must each personally date and sign the informed consent form with an integrated declaration on data privacy protection, whereby the physician must not sign before the volunteer. Original signed documents will be part of the investigator's file and retained with it. A copy of the signed informed consent document and study insurance policy must be given to the subject. The documents must be in a language understandable to the subject and must specify who informed the subject. The subjects will be informed as soon as possible if new information may influence his/her decision to participate in the trial. The communication of this information should be documented in the volunteer chart.

10.3. Insurance

Each volunteer is insured against any health impairment occurring as a result of participation in the study in accordance with the laws and regulations of the "German Arzneimittelgesetz". The insurance is covered by [redacted] and valid throughout the conduct of the study including follow-up for each individual volunteer. A copy of the insurance policy and conditions are distributed to the volunteer upon enrolment into the study and the volunteer is advised to adhere to the conditions of the insurance policy to safeguard a valid volunteer insurance.

Travel insurance will be included for all volunteers enrolled in the clinical trial.

10.4. Confidentiality

The data obtained in the course of the trial will be treated according to the European General Data Protection Regulation (Datenschutz-Grundverordnung; DS-GVO) and the applicable local data protection regulations as well as the AMG.
Subjects have to be informed about data protection in the clinical trial and to consent in writing to collect and process their personalized data as well as to transfer their pseudonymized data. The information has to be transparent, precise, easily accessible and understandable and is written in clear and simple language. The written privacy policy must be approved by the responsible ethics committee.

In order to maintain volunteer privacy, all data capture records, study drug accountability records, study reports and communications will identify the volunteer by the assigned volunteer number. The PI determines which persons are authorized to view personal data, the Volunteer Intification Log is only accessible to authorized study team members. Access rights to personal data (including pseudonymised data) are available to prevent unauthorized access to the data (both electronically and physically). Electronic systems and files are access-regulated, possibly password-protected. Documents and files are kept in lockable rooms, if necessary, cupboards with access control.

The volunteer name, initials and the full birth date should never be used in any correspondence with the Sponsor or on the Case Report Forms. The investigator will grant monitor(s) and auditor(s) and/or regulatory authorities direct access to the volunteer's original medical records for verification of data gathered on the data capture records and to audit the data collection process. Direct access includes examining, analyzing, and verifying any recorded data and reports that are important to the evaluation of the monitoring. The investigator is obliged to inform the volunteer that his/her trial-related records will be viewed without violating their confidentiality and that the collected information will only be made publicly available to the extent permitted by the applicable laws and regulations. All data will be stored either paper-based or electronically in a pseudonymous manner and handled strictly confidential. The investigators are obliged to keep all study data and information confidential and to use those data only in context with the persons involved in the trial conduct. Study material or information

developed in this trial must not be available to third parties, except for official representatives of the sponsor or regulatory authorities.

Data will be processed at the study site according to the written safety concept of this institution. Access to the data will be strictly limited to authorized persons. Loss of data is excluded due to extensive back-up procedures. All legal requirements concerning data protection and confidentiality will be respected. All authorized persons are sworn to secrecy.

In the case of withdrawal of consent the stored data collected to this time point will be stored and further used. Data not necessary any longer are deleted immediately.

Collected study data will be stored for at least 25 years after the end of the trial, if there are no other regulatory archiving periods. After archiving has expired, the data will be destructed in a data protection compliant manner.

When processing personal data, the following principles must be observed (pursuant to DS-GVO Article 5 “Principles relating to processing of personal data“):

Personal data shall be:

- processed lawfully, fairly and in a transparent manner in relation to the data subject
- collected for specified, explicit and legitimate purposes and not further processed in a manner that is incompatible with those purposes
- adequate, relevant and limited to what is necessary in relation to the purposes for which they are processed
- accurate and, where necessary, kept up to date
- kept in a form which permits identification of data subjects for no longer than is necessary for the purposes for which the personal data are processed
- processed in a manner that ensures appropriate security of the personal data, including protection against unauthorised or unlawful processing and against accidental loss, destruction or damage, using appropriate technical or organisational measures

10.5. Responsibility of the Investigator

The investigator should ensure that all persons assisting with the trial are adequately informed about the protocol, any amendments to the protocol, the trial treatments, and their trial-related duties and functions.

The investigator should maintain a list of subinvestigators and other appropriately qualified persons to whom he or she has delegated significant trial-related duties.

10.6. Registration of the Trial

Prior to the beginning of the clinical phase (First Patient In) the Sponsor / CI will register the trial in the EudraCT (2020-002502-75) as well as ClinicalTrials.gov Database.

10.7. Continuous Information to Independent Ethics Committee

According to the German Drug Law (AMG) and the GCP Ordinance, the EC and the competent authority (Paul-Ehrlich Institut, PEI) will be informed of all suspected serious unexpected adverse reactions (SUSARs). Both institutions will be informed in case the risk/ benefit assessment did change or any others new and significant hazards for subjects' safety or welfare did occur. In addition, upon activation and prior to discontinuation of a holding rule the sponsor informs the responsible authorities (section 6.5). Furthermore, a report on all observed SAEs will be submitted once a year – Annual Safety Report.
The EC and the regulatory authorities must be informed of the end of the trial. They will be provided with a summary of trial results within one year after the end of clinical phase.

10.8. Approval of Protocol and Subsequent Amendments

Before the start of the trial, the trial protocol, informed consent document, and any other appropriate documents will be submitted to the independent EC as well as to the competent authority (PEI). A written favourable vote of the EC and an (implicit) approval by the competent higher federal authority (PEI) as well as the notification of the local authorities (acc. to §67 AMG) are a prerequisite for initiation of this clinical trial. Before the first subject is enrolled in the trial, all ethical and legal requirements must be met. All planned substantial changes (see §10, (1) of German GCP-Regulation) will be submitted for approval to EC and the competent authority in writing as protocol amendments.

11. Publications

11.1. Reports

Within one year of the completion of the trial, the competent authority and the ethics committee will be supplied with a summary of the final report on the clinical trial containing the principle results.

All reports to the sponsor will be written in English language. All clinical, analytical and statistical results will be presented in a final clinical trial report (CTR). The outline of this report will accord to the ICH Topic E3.

11.2. Publication

The final results of this study will be presented at scientific meetings and published in a peer reviewed journal. All publications on result of this study should be based on the scientific reports (see 11.1) and are the responsibility of the CI. The authorship will reflect the contributions of each collaborating centre. Any publication, abstract or presentation based on patients included in this study must be approved by the CI. First safety data will be published after completion of EOSf of the last patient enrolled in the clinical trial.

No publications on planned or unplanned interim analyses (e.g. safety analysis for DSMB or provisionally results on immunological efficacy before finalization of the scientific reports) are allowed.

12. Financing

This study is financed by the “Sonderfördermaßnahme COVID-19” of the ministry of science, research and art of the state Baden-Wuerttemberg, Germany.

13. Literature

1. Mo P, Xing Y, Xiao Y, et al. Clinical characteristics of refractory COVID-19 pneumonia in Wuhan, China. *Clin Infect Dis* 2020 doi: 10.1093/cid/ciaa270 [published Online First: 2020/03/17]
2. Ng OT, Marimuthu K, Chia PY, et al. SARS-CoV-2 Infection among Travelers Returning from Wuhan, China. *N Engl J Med* 2020 doi: 10.1056/NEJMc2003100 [published Online First: 2020/03/13]
3. Khan S, Siddique R, Shereen MA, et al. The emergence of a novel coronavirus (SARS-CoV-2), their biology and therapeutic options. *J Clin Microbiol* 2020 doi: 10.1128/JCM.00187-20 [published Online First: 2020/03/13]
4. Organization WH. Report of the WHO-China Joint Mission on Coronavirus Disease 2019. 2020
5. Seder RA, Darrah PA, Roederer M. T-cell quality in memory and protection: implications for vaccine design. *Nat Rev Immunol* 2008;8(4):247-58. doi: 10.1038/nri2274 [published Online First: 2008/03/08]
6. Khan N, Best D, Bruton R, et al. T cell recognition patterns of immunodominant cytomegalovirus antigens in primary and persistent infection. *J Immunol* 2007;178(7):4455-65. doi: 10.4049/jimmunol.178.7.4455 [published Online First: 2007/03/21]
7. Hill GR, Tey SK, Beagley L, et al. Successful immunotherapy of HCMV disease using virus-specific T cells expanded from an allogeneic stem cell transplant recipient. *Am J Transplant* 2010;10(1):173-9. doi: 10.1111/j.1600-6143.2009.02872.x [published Online First: 2009/11/19]
8. Feucht J, Joachim L, Lang P, et al. Adoptive T-cell transfer for refractory viral infections with cytomegalovirus, Epstein-Barr virus or adenovirus after allogeneic stem cell transplantation. *Klin Padiatr* 2013;225(3):164-9. doi: 10.1055/s-0033-1333749 [published Online First: 2013/05/24]
9. Hanajiri R, Sani GM, Hanley PJ, et al. Generation of Zika virus-specific T cells from seropositive and virus-naive donors for potential use as an autologous or "off-the-shelf" immunotherapeutic. *Cytotherapy* 2019;21(8):840-55. doi: 10.1016/j.jcyt.2019.06.008 [published Online First: 2019/07/08]
10. Wisskirchen K, Kah J, Malo A, et al. T cell receptor grafting allows virological control of Hepatitis B virus infection. *J Clin Invest* 2019;129(7):2932-45. doi: 10.1172/JCI120228 [published Online First: 2019/05/01]
11. Hanajiri R, Sani GM, Saunders D, et al. Generation of Norovirus-Specific T Cells From Human Donors With Extensive Cross-Reactivity to Variant Sequences: Implications for Immunotherapy. *J Infect Dis* 2020;221(4):578-88. doi: 10.1093/infdis/jiz491 [published Online First: 2019/09/29]
12. Channappanavar R, Fett C, Zhao J, et al. Virus-specific memory CD8 T cells provide substantial protection from lethal severe acute respiratory syndrome coronavirus infection. *J Virol* 2014;88(19):11034-44. doi: 10.1128/JVI.01505-14 [published Online First: 2014/07/25]
13. Channappanavar R, Zhao J, Perlman S. T cell-mediated immune response to respiratory coronaviruses. *Immunol Res* 2014;59(1-3):118-28. doi: 10.1007/s12026-014-8534-z [published Online First: 2014/05/23]
14. Zhao J, Zhao J, Mangalam AK, et al. Airway Memory CD4(+) T Cells Mediate Protective Immunity against Emerging Respiratory Coronaviruses. *Immunity* 2016;44(6):1379-91. doi: 10.1016/j.immuni.2016.05.006 [published Online First: 2016/06/12]
15. Zhao J, Zhao J, Perlman S. T cell responses are required for protection from clinical disease and for virus clearance in severe acute respiratory syndrome coronavirus-infected mice. *J Virol* 2010;84(18):9318-25. doi: 10.1128/JVI.01049-10 [published Online First: 2010/07/09]

16. Liu L, Wei Q, Lin Q, et al. Anti-spike IgG causes severe acute lung injury by skewing macrophage responses during acute SARS-CoV infection. *JCI Insight* 2019;4(4) doi: 10.1172/jci.insight.123158 [published Online First: 2019/03/05]
17. Tang F, Quan Y, Xin ZT, et al. Lack of peripheral memory B cell responses in recovered patients with severe acute respiratory syndrome: a six-year follow-up study. *J Immunol* 2011;186(12):7264-8. doi: 10.4049/jimmunol.0903490 [published Online First: 2011/05/18]
18. Liu WJ, Zhao M, Liu K, et al. T-cell immunity of SARS-CoV: Implications for vaccine development against MERS-CoV. *Antiviral Res* 2017;137:82-92. doi: 10.1016/j.antiviral.2016.11.006 [published Online First: 2016/11/15]
19. Grifoni A, Weiskopf D, Ramirez SI, et al. Targets of T Cell Responses to SARS-CoV-2 Coronavirus in Humans with COVID-19 Disease and Unexposed Individuals. *Cell* 2020 doi: 10.1016/j.cell.2020.05.015 [published Online First: 2020/05/31]
20. Braun J, Loyal L, Frentsch M, et al. Presence of SARS-CoV-2 reactive T cells in COVID-19 patients and healthy donors. *medRxiv* 2020:2020.04.17.20061440. doi: 10.1101/2020.04.17.20061440
21. Vali B, Tohn R, Cohen MJ, et al. Characterization of cross-reactive CD8+ T-cell recognition of HLA-A2-restricted HIV-Gag (SLYNTVATL) and HCV-NS5b (ALYDVVSKL) epitopes in individuals infected with human immunodeficiency and hepatitis C viruses. *J Virol* 2011;85(1):254-63. doi: 10.1128/JVI.01743-10 [published Online First: 2010/10/29]
22. Acierno PM, Newton DA, Brown EA, et al. Cross-reactivity between HLA-A2-restricted FLU-M1:58-66 and HIV p17 GAG:77-85 epitopes in HIV-infected and uninfected individuals. *J Transl Med* 2003;1(1):3. doi: 10.1186/1479-5876-1-3 [published Online First: 2003/10/07]
23. Clute SC, Watkin LB, Cornberg M, et al. Cross-reactive influenza virus-specific CD8+ T cells contribute to lymphoproliferation in Epstein-Barr virus-associated infectious mononucleosis. *J Clin Invest* 2005;115(12):3602-12. doi: 10.1172/JCI25078 [published Online First: 2005/11/26]
24. Nelde A, Bilich T, Heitmann JS, et al. SARS-CoV-2-derived peptides define heterologous and COVID-19-induced T cell recognition. *Nat Immunol* 2021;22(1):74-85. doi: 10.1038/s41590-020-00808-x [published Online First: 2020/10/02]
25. Rodda LB, Netland J, Shehata L, et al. Functional SARS-CoV-2-Specific Immune Memory Persists after Mild COVID-19. *Cell* 2021;184(1):169-83 e17. doi: 10.1016/j.cell.2020.11.029 [published Online First: 2020/12/10]
26. Zuo J, Dowell A, Pearce H, et al. Robust SARS-CoV-2-specific T-cell immunity is maintained at 6 months following primary infection. *bioRxiv* 2020:2020.11.01.362319. doi: 10.1101/2020.11.01.362319
27. Dan JM, Mateus J, Kato Y, et al. Immunological memory to SARS-CoV-2 assessed for up to 8 months after infection. *Science* 2021;371(6529):eabf4063. doi: 10.1126/science.abf4063 [published Online First: 2021/01/08]
28. Le Bert N, Tan AT, Kunasegaran K, et al. SARS-CoV-2-specific T cell immunity in cases of COVID-19 and SARS, and uninfected controls. *Nature* 2020;584(7821):457-62. doi: 10.1038/s41586-020-2550-z [published Online First: 2020/07/16]
29. Sekine T, Perez-Potti A, Rivera-Ballesteros O, et al. Robust T Cell Immunity in Convalescent Individuals with Asymptomatic or Mild COVID-19. *Cell* 2020;183(1):158-68 e14. doi: 10.1016/j.cell.2020.08.017 [published Online First: 2020/09/28]
30. Habel JR, Nguyen THO, van de Sandt CE, et al. Suboptimal SARS-CoV-2-specific CD8(+) T cell response associated with the prominent HLA-A*02:01 phenotype. *Proc Natl Acad Sci U S A* 2020;117(39):24384-91. doi: 10.1073/pnas.2015486117 [published Online First: 2020/09/12]
31. Peng Y, Mentzer AJ, Liu G, et al. Broad and strong memory CD4(+) and CD8(+) T cells induced by SARS-CoV-2 in UK convalescent individuals following COVID-19. *Nat*

Immunol 2020;21(11):1336-45. doi: 10.1038/s41590-020-0782-6 [published Online First: 2020/09/06]
32. Chen Z, John Wherry E. T cell responses in patients with COVID-19. *Nat Rev Immunol* 2020;20(9):529-36. doi: 10.1038/s41577-020-0402-6 [published Online First: 2020/07/31]
33. Braun J, Loyal L, Frentsch M, et al. SARS-CoV-2-reactive T cells in healthy donors and patients with COVID-19. *Nature* 2020;587(7833):270-74. doi: 10.1038/s41586-020-2598-9 [published Online First: 2020/07/30]
34. Grifoni A, Weiskopf D, Ramirez SI, et al. Targets of T Cell Responses to SARS-CoV-2 Coronavirus in Humans with COVID-19 Disease and Unexposed Individuals. *Cell* 2020;181(7):1489-501 e15. doi: 10.1016/j.cell.2020.05.015 [published Online First: 2020/05/31]
35. Mateus J, Grifoni A, Tarke A, et al. Selective and cross-reactive SARS-CoV-2 T cell epitopes in unexposed humans. *Science* 2020;370(6512):89-94. doi: 10.1126/science.abd3871 [published Online First: 2020/08/06]
36. Weiskopf D, Schmitz KS, Raadsen MP, et al. Phenotype and kinetics of SARS-CoV-2-specific T cells in COVID-19 patients with acute respiratory distress syndrome. *Sci Immunol* 2020;5(48) doi: 10.1126/sciimmunol.abd2071 [published Online First: 2020/06/28]
37. Bilich T, Nelde A, Heitmann J, et al. Differential T cell and antibody kinetics delineate SARS-CoV-2 peptides mediating long-term immune response after COVID-19. *Science transl Medicine* 2021(in press)
38. Tan AT, Linster M, Tan CW, et al. Early induction of functional SARS-CoV-2-specific T cells associates with rapid viral clearance and mild disease in COVID-19 patients. *Cell Rep* 2021;34(6):108728. doi: 10.1016/j.celrep.2021.108728 [published Online First: 2021/02/01]
39. Long QX, Tang XJ, Shi QL, et al. Clinical and immunological assessment of asymptomatic SARS-CoV-2 infections. *Nat Med* 2020;26(8):1200-04. doi: 10.1038/s41591-020-0965-6 [published Online First: 2020/06/20]
40. Kreer C, Zehner M, Weber T, et al. Longitudinal Isolation of Potent Near-Germline SARS-CoV-2-Neutralizing Antibodies from COVID-19 Patients. *Cell* 2020;182(4):843-54 e12. doi: 10.1016/j.cell.2020.06.044 [published Online First: 2020/07/17]
41. Ripperger TJ, Uhrlaub JL, Watanabe M, et al. Orthogonal SARS-CoV-2 Serological Assays Enable Surveillance of Low-Prevalence Communities and Reveal Durable Humoral Immunity. *Immunity* 2020;53(5):925-33 e4. doi: 10.1016/j.immuni.2020.10.004 [published Online First: 2020/11/02]
42. Ibarrondo FJ, Fulcher JA, Goodman-Meza D, et al. Rapid Decay of Anti-SARS-CoV-2 Antibodies in Persons with Mild Covid-19. *N Engl J Med* 2020;383(11):1085-87. doi: 10.1056/NEJMc2025179 [published Online First: 2020/07/25]
43. Gaebler C, Wang Z, Lorenzi JCC, et al. Evolution of antibody immunity to SARS-CoV-2. *Nature* 2021 doi: 10.1038/s41586-021-03207-w [published Online First: 2021/01/19]
44. Soresina A, Moratto D, Chiarini M, et al. Two X-linked agammaglobulinemia patients develop pneumonia as COVID-19 manifestation but recover. *Pediatr Allergy Immunol* 2020;31(5):565-69. doi: 10.1111/pai.13263 [published Online First: 2020/04/23]
45. Deres K, Schild H, Wiesmuller KH, et al. In vivo priming of virus-specific cytotoxic T lymphocytes with synthetic lipopeptide vaccine. *Nature* 1989;342(6249):561-4. doi: 10.1038/342561a0 [published Online First: 1989/11/30]
46. Falk K, Rotzschke O, Rammensee HG. Cellular peptide composition governed by major histocompatibility complex class I molecules. *Nature* 1990;348(6298):248-51. doi: 10.1038/348248a0 [published Online First: 1990/11/15]
47. Rammensee HG. Survival of the fitters. *Nature* 2002;419(6906):443-5. doi: 10.1038/419443a [published Online First: 2002/10/09]

48. Rammensee H, Bachmann J, Emmerich NP, et al. SYFPEITHI: database for MHC ligands and peptide motifs. *Immunogenetics* 1999;50(3-4):213-9. doi: 10.1007/s002510050595 [published Online First: 1999/12/22]
49. Bilich T, Nelde A, Bichmann L, et al. The HLA ligandome landscape of chronic myeloid leukemia delineates novel T-cell epitopes for immunotherapy. *Blood* 2019;133(6):550-65. doi: 10.1182/blood-2018-07-866830 [published Online First: 2018/12/12]
50. Lubke M, Spalt S, Kowalewski DJ, et al. Identification of HCMV-derived T cell epitopes in seropositive individuals through viral deletion models. *J Exp Med* 2020;217(3) doi: 10.1084/jem.20191164 [published Online First: 2019/12/24]
51. Berlin C, Kowalewski DJ, Schuster H, et al. Mapping the HLA ligandome landscape of acute myeloid leukemia: a targeted approach toward peptide-based immunotherapy. *Leukemia* 2015;29(3):647-59. doi: 10.1038/leu.2014.233 [published Online First: 2014/08/06]
52. Kowalewski DJ, Schuster H, Backert L, et al. HLA ligandome analysis identifies the underlying specificities of spontaneous antileukemia immune responses in chronic lymphocytic leukemia (CLL). *Proc Natl Acad Sci U S A* 2015;112(2):E166-75. doi: 10.1073/pnas.1416389112
53. Nastke MD, Herrgen L, Walter S, et al. Major contribution of codominant CD8 and CD4 T cell epitopes to the human cytomegalovirus-specific T cell repertoire. *Cell Mol Life Sci* 2005;62(1):77-86. doi: 10.1007/s00018-004-4363-x [published Online First: 2004/12/25]
54. Icheva V, Kayser S, Wolff D, et al. Adoptive transfer of epstein-barr virus (EBV) nuclear antigen 1-specific t cells as treatment for EBV reactivation and lymphoproliferative disorders after allogeneic stem-cell transplantation. *J Clin Oncol* 2013;31(1):39-48. doi: 10.1200/JCO.2011.39.8495 [published Online First: 2012/11/22]
55. Hilf N, Kuttruff-Coqui S, Frenzel K, et al. Actively personalized vaccination trial for newly diagnosed glioblastoma. *Nature* 2019;565(7738):240-45. doi: 10.1038/s41586-018-0810-y [published Online First: 2018/12/21]
56. Baumgaertner P, Jandus C, Rivals JP, et al. Vaccination-induced functional competence of circulating human tumor-specific CD8 T-cells. *Int J Cancer* 2012;130(11):2607-17. doi: 10.1002/ijc.26297 [published Online First: 2011/07/29]
57. Freund J. The effect of paraffin oil and mycobacteria on antibody formation and sensitization; a review. *Am J Clin Pathol* 1951;21(7):645-56. doi: 10.1093/ajcp/21.7.645 [published Online First: 1951/07/01]
58. Rammensee HG, Wiesmuller KH, Chandran PA, et al. A new synthetic toll-like receptor 1/2 ligand is an efficient adjuvant for peptide vaccination in a human volunteer. *J Immunother Cancer* 2019;7(1):307. doi: 10.1186/s40425-019-0796-5 [published Online First: 2019/11/16]
59. Alam I, Goldeck D, Larbi A, et al. Aging affects the proportions of T and B cells in a group of elderly men in a developing country--a pilot study from Pakistan. *Age (Dordr)* 2013;35(5):1521-30. doi: 10.1007/s11357-012-9455-1 [published Online First: 2012/07/20]
60. Fulop T, Larbi A, Pawelec G. Human T cell aging and the impact of persistent viral infections. *Front Immunol* 2013;4:271. doi: 10.3389/fimmu.2013.00271 [published Online First: 2013/09/26]
61. Lambkin R, Novelli P, Oxford J, et al. Human genetics and responses to influenza vaccination: clinical implications. *Am J Pharmacogenomics* 2004;4(5):293-8. doi: 10.2165/00129785-200404050-00002 [published Online First: 2004/10/07]
62. Molano A, Park SH, Chiu YH, et al. Cutting edge: the IgG response to the circumsporozoite protein is MHC class II-dependent and CD1d-independent: exploring the role of GPIs in NK T cell activation and antimalarial responses. *J Immunol* 2000;164(10):5005-9. doi: 10.4049/jimmunol.164.10.5005 [published Online First: 2000/05/09]

63. Oliveira GA, Kumar KA, Calvo-Calle JM, et al. Class II-restricted protective immunity induced by malaria sporozoites. *Infect Immun* 2008;76(3):1200-6. doi: 10.1128/IAI.00566-07 [published Online First: 2007/12/28]
64. Xu R, Johnson AJ, Liggitt D, et al. Cellular and humoral immunity against vaccinia virus infection of mice. *J Immunol* 2004;172(10):6265-71. doi: 10.4049/jimmunol.172.10.6265 [published Online First: 2004/05/07]
65. Sette A, Moutaftsi M, Moyron-Quiroz J, et al. Selective CD4+ T cell help for antibody responses to a large viral pathogen: deterministic linkage of specificities. *Immunity* 2008;28(6):847-58. doi: 10.1016/j.immuni.2008.04.018 [published Online First: 2008/06/14]
66. Shedlock DJ, Shen H. Requirement for CD4 T cell help in generating functional CD8 T cell memory. *Science* 2003;300(5617):337-9. doi: 10.1126/science.1082305 [published Online First: 2003/04/12]
67. Carvalho LH, Sano G, Hafalla JC, et al. IL-4-secreting CD4+ T cells are crucial to the development of CD8+ T-cell responses against malaria liver stages. *Nat Med* 2002;8(2):166-70. doi: 10.1038/nm0202-166 [published Online First: 2002/02/01]
68. Kemball CC, Pack CD, Guay HM, et al. The antiviral CD8+ T cell response is differentially dependent on CD4+ T cell help over the course of persistent infection. *J Immunol* 2007;179(2):1113-21. doi: 10.4049/jimmunol.179.2.1113 [published Online First: 2007/07/10]
69. Marzo AL, Vezys V, Klonowski KD, et al. Fully functional memory CD8 T cells in the absence of CD4 T cells. *J Immunol* 2004;173(2):969-75. doi: 10.4049/jimmunol.173.2.969 [published Online First: 2004/07/09]
70. van de Berg PJ, van Leeuwen EM, ten Berge IJ, et al. Cytotoxic human CD4(+) T cells. *Curr Opin Immunol* 2008;20(3):339-43. doi: 10.1016/j.coi.2008.03.007 [published Online First: 2008/04/29]
71. Johnson AJ, Chu CF, Milligan GN. Effector CD4+ T-cell involvement in clearance of infectious herpes simplex virus type 1 from sensory ganglia and spinal cords. *J Virol* 2008;82(19):9678-88. doi: 10.1128/JVI.01159-08 [published Online First: 2008/08/01]
72. Elyaman W, Kivisakk P, Reddy J, et al. Distinct functions of autoreactive memory and effector CD4+ T cells in experimental autoimmune encephalomyelitis. *Am J Pathol* 2008;173(2):411-22. doi: 10.2353/ajpath.2008.080142 [published Online First: 2008/06/28]
73. Tsuji M, Romero P, Nussenzweig RS, et al. CD4+ cytolytic T cell clone confers protection against murine malaria. *J Exp Med* 1990;172(5):1353-7. doi: 10.1084/jem.172.5.1353 [published Online First: 1990/11/01]
74. Harrington LE, Mangan PR, Weaver CT. Expanding the effector CD4 T-cell repertoire: the Th17 lineage. *Curr Opin Immunol* 2006;18(3):349-56. doi: 10.1016/j.coi.2006.03.017 [published Online First: 2006/04/18]
75. Kreer C, Zehner M, Weber T, et al. Longitudinal Isolation of Potent Near-Germline SARS-CoV-2-Neutralizing Antibodies from COVID-19 Patients. *Cell* 2020 doi: 10.1016/j.cell.2020.06.044 [published Online First: 2020/07/17]
76. Nelde A, Bilich T, Heitmann JS, et al. SARS-CoV-2 T-cell epitopes define heterologous and COVID-19-induced T-cell recognition. In: Preprint, ed. Research Square, 2020.
77. Steere AC, Sikand VK, Meurice F, et al. Vaccination against Lyme disease with recombinant Borrelia burgdorferi outer-surface lipoprotein A with adjuvant. Lyme Disease Vaccine Study Group. *N Engl J Med* 1998;339(4):209-15. doi: 10.1056/NEJM199807233390401 [published Online First: 1998/07/23]
78. Sigal LH, Zahradnik JM, Lavin P, et al. A vaccine consisting of recombinant Borrelia burgdorferi outer-surface protein A to prevent Lyme disease. Recombinant Outer-Surface Protein A Lyme Disease Vaccine Study Consortium. *N Engl J Med* 1998;339(4):216-22. doi: 10.1056/NEJM199807233390402 [published Online First: 1998/07/23]

79. Opie EL, Freund J. An Experimental Study of Protective Inoculation with Heat Killed Tubercle Bacilli. *J Exp Med* 1937;66(6):761-88. doi: 10.1084/jem.66.6.761 [published Online First: 1937/11/30]
80. Jensen FC, Savary JR, Diveley JP, et al. Adjuvant activity of incomplete Freund's adjuvant. *Adv Drug Deliv Rev* 1998;32(3):173-86. doi: 10.1016/s0169-409x(98)00009-x [published Online First: 2000/06/06]
81. Rammensee HG, Stevanovic S, Gouttefangeas C, et al. Designing a therapeutic SARS-CoV-2 T-cell-inducing vaccine for high-risk patient groups. *Research Square [preprint]* 2020 doi:

10.21203/rs.3.rs-27316/v1
82. Kran AM, Sorensen B, Nyhus J, et al. HLA- and dose-dependent immunogenicity of a peptide-based HIV-1 immunotherapy candidate (Vacc-4x). *Aids* 2004;18(14):1875-83.
83. Feyerabend S, Stevanovic S, Gouttefangeas C, et al. Novel multi-peptide vaccination in Hla-A2+ hormone sensitive patients with biochemical relapse of prostate cancer. *Prostate* 2009;69(9):917-27. doi: 10.1002/pros.20941 [published Online First: 2009/03/10]
84. Sato Y, Shomura H, Maeda Y, et al. Immunological evaluation of peptide vaccination for patients with gastric cancer based on pre-existing cellular response to peptide. *Cancer science* 2003;94(9):802-8.
85. Noguchi M, Kobayashi K, Suetsugu N, et al. Induction of cellular and humoral immune responses to tumor cells and peptides in HLA-A24 positive hormone-refractory prostate cancer patients by peptide vaccination. *Prostate* 2003;57(1):80-92. doi: 10.1002/pros.10276
86. Atsmon J, Kate-Ilovitz E, Shaikevich D, et al. Safety and immunogenicity of multimeric-001--a novel universal influenza vaccine. *J Clin Immunol* 2012;32(3):595-603. doi: 10.1007/s10875-011-9632-5 [published Online First: 2012/02/10]
87. Salk JE, Bailey ML, Laurent AM. The use of adjuvants in studies on influenza immunization. II. Increased antibody formation in human subjects inoculated with influenza virus vaccine in a water in-oil emulsion. *Am J Hyg* 1952;55(3):439-56. doi: 10.1093/oxfordjournals.aje.a119534 [published Online First: 1952/05/01]
88. Meiklejohn G. Adjuvant influenza adenovirus vaccine. *JAMA* 1962;179:594-7. doi: 10.1001/jama.1962.03050080006002 [published Online First: 1962/02/24]
89. van Doorn E, Liu H, Huckriede A, et al. Safety and tolerability evaluation of the use of Montanide ISA51 as vaccine adjuvant: A systematic review. *Hum Vaccin Immunother* 2016;12(1):159-69. doi: 10.1080/21645515.2015.1071455
90. Carr A, Rodriguez E, Arango Mdel C, et al. Immunotherapy of advanced breast cancer with a heterophilic ganglioside (NeuGcGM3) cancer vaccine. *J Clin Oncol* 2003;21(6):1015-21. doi: 10.1200/JCO.2003.02.124
91. Yamaue H, Tsunoda T, Tani M, et al. Randomized phase II/III clinical trial of elpamotide for patients with advanced pancreatic cancer: PEGASUS-PC Study. *Cancer science* 2015;106(7):883-90. doi: 10.1111/cas.12674 [published Online First: 2015/04/14]
92. Polack FP, Thomas SJ, Kitchin N, et al. Safety and Efficacy of the BNT162b2 mRNA Covid-19 Vaccine. *N Engl J Med* 2020;383(27):2603-15. doi: 10.1056/NEJMoa2034577 [published Online First: 2020/12/11]
93. Ramasamy MN, Minassian AM, Ewer KJ, et al. Safety and immunogenicity of ChAdOx1 nCoV-19 vaccine administered in a prime-boost regimen in young and old adults (COV002): a single-blind, randomised, controlled, phase 2/3 trial. *Lancet* 2021;396(10267):1979-93. doi: 10.1016/S0140-6736(20)32466-1 [published Online First: 2020/11/23]
94. Baden LR, El Sahly HM, Essink B, et al. Efficacy and Safety of the mRNA-1273 SARS-CoV-2 Vaccine. *N Engl J Med* 2021;384(5):403-16. doi: 10.1056/NEJMoa2035389 [published Online First: 2020/12/31]

95. Institute PE. Verdachtsfälle von Nebenwirkungen und Impfkomplikationen nach Impfung zum Schutz vor COVID-19 2021 [Available from: https://www.pei.de/SharedDocs/Downloads/DE/newsroom/dossiers/sicherheitsberichte/sicherheitsbericht-27-12-bis-26-02-21.pdf?__blob=publicationFile&v=6 accessed 09-03-2021 2021.
96. CDC. Post Vaccine Considerations for Healthcare Personnel 2020 [Available from: https://www.cdc.gov/coronavirus/2019-ncov/hcp/post-vaccine-considerations-healthcare-personnel.html accessed 09-03-2021 2021.
97. Blumenthal KG, Freeman EE, Saff RR, et al. Delayed Large Local Reactions to mRNA-1273 Vaccine against SARS-CoV-2. *N Engl J Med* 2021 doi: 10.1056/NEJMc2102131 [published Online First: 2021/03/04]
98. Stern LJ, Calvo-Calle JM. HLA-DR: molecular insights and vaccine design. *Curr Pharm Des* 2009;15(28):3249-61. doi: 10.2174/138161209789105171 [published Online First: 2009/10/29]
99. Herrington DA, Clyde DF, Losonsky G, et al. Safety and immunogenicity in man of a synthetic peptide malaria vaccine against Plasmodium falciparum sporozoites. *Nature* 1987;328(6127):257-9. doi: 10.1038/328257a0 [published Online First: 1987/07/16]
100. Weihrauch MR, Ansen S, Jurkiewicz E, et al. Phase I/II combined chemoimmunotherapy with carcinoembryonic antigen-derived HLA-A2-restricted CAP-1 peptide and irinotecan, 5-fluorouracil, and leucovorin in patients with primary metastatic colorectal cancer. *Clinical cancer research : an official journal of the American Association for Cancer Research* 2005;11(16):5993-6001. doi: 10.1158/1078-0432.CCR-05-0018
101. Peoples GE, Gurney JM, Hueman MT, et al. Clinical trial results of a HER2/neu (E75) vaccine to prevent recurrence in high-risk breast cancer patients. *J Clin Oncol* 2005;23(30):7536-45. doi: 10.1200/JCO.2005.03.047
102. Walter S, Weinschenk T, Stenzl A, et al. Multipeptide immune response to cancer vaccine IMA901 after single-dose cyclophosphamide associates with longer patient survival. *Nat Med* 2012 doi: 10.1038/nm.2883 [published Online First: 2012/07/31]
103. Mailander V, Scheibenbogen C, Thiel E, et al. Complete remission in a patient with recurrent acute myeloid leukemia induced by vaccination with WT1 peptide in the absence of hematological or renal toxicity. *Leukemia* 2004;18(1):165-6. doi: 10.1038/sj.leu.2403186
104. Oka Y, Tsuboi A, Taguchi T, et al. Induction of WT1 (Wilms' tumor gene)-specific cytotoxic T lymphocytes by WT1 peptide vaccine and the resultant cancer regression. *Proc Natl Acad Sci U S A* 2004;101(38):13885-90. doi: 10.1073/pnas.0405884101
105. Van Tendeloo VF, Van de Velde A, Van Driessche A, et al. Induction of complete and molecular remissions in acute myeloid leukemia by Wilms' tumor 1 antigen-targeted dendritic cell vaccination. *Proc Natl Acad Sci U S A* 2010;107(31):13824-9. doi: 10.1073/pnas.1008051107
106. Schmitt M, Schmitt A, Rojewski MT, et al. RHAMM-R3 peptide vaccination in patients with acute myeloid leukemia, myelodysplastic syndrome, and multiple myeloma elicits immunologic and clinical responses. *Blood* 2008;111(3):1357-65. doi: 10.1182/blood-2007-07-099366 [published Online First: 2007/11/06]
107. Schwartzentruber DJ, Lawson DH, Richards JM, et al. gp100 peptide vaccine and interleukin-2 in patients with advanced melanoma. *N Engl J Med* 2011;364(22):2119-27. doi: 10.1056/NEJMoa1012863
108. Mittendorf EA, Clifton GT, Holmes JP, et al. Clinical trial results of the HER-2/neu (E75) vaccine to prevent breast cancer recurrence in high-risk patients: from US Military Cancer Institute Clinical Trials Group Study I-01 and I-02. *Cancer* 2012;118(10):2594-602. doi: 10.1002/cncr.26574 [published Online First: 2011/10/13]
109. Weinschenk T, Gouttefangeas C, Schirle M, et al. Integrated functional genomics approach for the design of patient-individual antitumor vaccines. *Cancer Res* 2002;62(20):5818-27. [published Online First: 2002/10/18]

110. Honda-Okubo Y, Barnard D, Ong CH, et al. Severe acute respiratory syndrome-associated coronavirus vaccines formulated with delta inulin adjuvants provide enhanced protection while ameliorating lung eosinophilic immunopathology. *J Virol* 2015;89(6):2995-3007. doi: 10.1128/JVI.02980-14 [published Online First: 2014/12/19]
111. Graham BS. Rapid COVID-19 vaccine development. *Science* 2020 doi: 10.1126/science.abb8923 [published Online First: 2020/05/10]
112. Van den Heuvel MM, Burgers SA, van Zandwijk N. Immunotherapy in non-small-cell lung carcinoma: from inflammation to vaccination. *Clinical lung cancer* 2009;10(2):99-105. doi: 10.3816/CLC.2009.n.012
113. Wu Y, Ellis RD, Shaffer D, et al. Phase 1 trial of malaria transmission blocking vaccine candidates Pfs25 and Pvs25 formulated with montanide ISA 51. *PLoS One* 2008;3(7):e2636. doi: 10.1371/journal.pone.0002636
114. Widenmeyer M, Griesemann H, Stevanovic S, et al. Promiscuous survivin peptide induces robust CD4+ T-cell responses in the majority of vaccinated cancer patients. *Int J Cancer* 2012;131(1):140-9. doi: 10.1002/ijc.26365 [published Online First: 2011/08/23]
115. Britten CM, Gouttefangeas C, Welters MJ, et al. The CIMT-monitoring panel: a two-step approach to harmonize the enumeration of antigen-specific CD8+ T lymphocytes by structural and functional assays. *Cancer Immunol Immunother* 2008;57(3):289-302. doi: 10.1007/s00262-007-0378-0 [published Online First: 2007/08/28]
116. Neumann A, Horzer H, Hillen N, et al. Identification of HLA ligands and T-cell epitopes for immunotherapy of lung cancer. *Cancer Immunol Immunother* 2013;62(9):1485-97. doi: 10.1007/s00262-013-1454-2 [published Online First: 2013/07/03]
117. Tavares Da Silva F, De Keyser F, Lambert PH, et al. Optimal approaches to data collection and analysis of potential immune mediated disorders in clinical trials of new vaccines. *Vaccine* 2013;31(14):1870-6. doi: 10.1016/j.vaccine.2013.01.042 [published Online First: 2013/02/09]

Regulatory References

Medicinal Products Act (Arzneimittelgesetz), published on 12 December 2005 (Federal Law Gazette [BGBl.] Part I p. 3394), last amended by Article 3 of the Law of 18 July 2017 (BGBl. I p. 2757)

Ordinance on the implementation of Good Clinical Practice in the conduct of clinical trials on medicinal products for use in humans (GCP Ordinance - GCP-V), published on 09 August 2004 (Federal Law Gazette (BGBl.) I p. 2081), last amended by Article 8 of the Law of 19. Oktober 2012 (BGBl. I S. 2192)

REGULATION (EU) No 536/2014 OF THE EUROPEAN PARLIAMENT AND OF THE COUNCIL of 16 April 2014 on clinical trials on medicinal products for human use, and repealing Directive 2001/20/EC

Medical Devices Act (Medizinproduktegesetz), published on 07.August 2002 (Federal Law Gazette (BGBl.) I p. 3146), last amended by Article 7 of the Law of 18 July 2017 (BGBl. I p. 2757)

ICH Topic E3, Note for Guidance on Structure and Content of Clinical Study Reports (CPMP/ICH/137/95), July 1996

ICH Topic E 6 (R2), Note for Guidance on Good Clinical Practice (CPMP/ICH/135/95), December 2016

ICH Topic E 8, Note for Guidance on General Considerations for Clinical Trials (CPMP/ICH/291/95), March 1998

Clinical Trial Facilitation Group, Recommendations related to contraception and pregnancy testing in clinical trials, 15.09.2014)

EMEA-Guideline On Data Monitoring Committees (EMEA/CHMP/EWP/5872/03 Corr), January 2006

REGULATION (EU) 2016/679 OF THE EUROPEAN PARLIAMENT AND OF THE COUNCIL of 27 April 2016 on the protection of natural persons with regard to the processing of personal data and on the free movement of such data, and repealing Directive 95/46/EC (General Data Protection Regulation)

14. Appendix

14.1. Common Terminology Criteria for Adverse Events (CTCAE) Version

https://ctep.cancer.gov/protocoldevelopment/electronic_applications/docs/CTCAE_v5_Quick_Reference_5x7.pdf

14.2. List of central laboratories

[redacted]

[redacted]

[redacted]

14.3. Volunteer diary

Studie P-pVAC-SARS-CoV-2

Probanden-ID *(vom Arzt auszufüllen)*: [_ _ _ _] - [_ _ _]

Datum der Impfung: [_ _] [_ _] [20_ _]

1. Richtlinien

Füllen Sie Ihr Tagebuch (**täglich**) mit Ankreuzen und gegebenenfalls weiteren Ergänzungen aus. Falls Sie eine Frage nicht beantworten können, streichen Sie diese bitte durch. Falls Sie Fragen mit „Ja" beantworten, füllen Sie bitte weitere Angaben aus. Bei Rückfragen oder starken Beschwerden, melden Sie sich bitte an Ihrem Prüfzentrum.

2. Tag der Impfung (d1) [_ _] [_ _] [20_ _]

		Ja	Nein	Weitere Angaben
1.	Haben Sie Schmerzen an der Impfstelle?	☐	☐	____________________
2.	Ist die Impfstelle gerötet oder geschwollen?	☐	☐	____________________
3.	Haben Sie Fieber, Schüttelfrost oder Gliederschmerzen?	☐	☐	____________________
4.	Haben Sie Kopfschmerzen, Müdigkeit oder Übelkeit?	☐	☐	____________________
5.	Haben Sie andere Beschwerden?	☐	☐	____________________

3. Tag 2 nach der Impfung (d2) [_ _] [_ _] [20_ _]

		Ja	Nein	Weitere Angaben
1.	Haben Sie Schmerzen an der Impfstelle?	☐	☐	____________________
2.	Ist die Impfstelle gerötet oder geschwollen?	☐	☐	____________________
3.	Haben Sie Fieber, Schüttelfrost oder Gliederschmerzen?	☐	☐	____________________
4.	Haben Sie Kopfschmerzen, Müdigkeit oder Übelkeit?	☐	☐	____________________

		Ja	Nein	Weitere Angaben
5.	Haben Sie andere Beschwerden?	☐	☐	____________

4. Tag 3 nach der Impfung (d3) [_ _] [_ _] [20_ _]

		Ja	Nein	Weitere Angaben
1.	Haben Sie Schmerzen an der Impfstelle?	☐	☐	____________
2.	Ist die Impfstelle gerötet oder geschwollen?	☐	☐	____________
3.	Haben Sie Fieber, Schüttelfrost oder Gliederschmerzen?	☐	☐	____________
4.	Haben Sie Kopfschmerzen, Müdigkeit oder Übelkeit?	☐	☐	____________
5.	Haben Sie andere Beschwerden?	☐	☐	____________

5. Tag 4 nach der Impfung (d4) [_ _] [_ _] [20_ _]

		Ja	Nein	Weitere Angaben
1.	Haben Sie Schmerzen an der Impfstelle?	☐	☐	____________
2.	Ist die Impfstelle gerötet oder geschwollen?	☐	☐	____________
3.	Haben Sie Fieber, Schüttelfrost oder Gliederschmerzen?	☐	☐	____________
4.	Haben Sie Kopfschmerzen, Müdigkeit oder Übelkeit?	☐	☐	____________
5.	Haben Sie andere Beschwerden?	☐	☐	____________

6. Tag 5 nach der Impfung (d5) [_ _] [_ _] [20_ _]

		Ja	Nein	Weitere Angaben
1.	Haben Sie Schmerzen an der Impfstelle?	☐	☐	____________
2.	Ist die Impfstelle gerötet oder geschwollen?	☐	☐	____________

3.	Haben Sie Fieber, Schüttelfrost oder Gliederschmerzen?	☐	☐	______________________
4.	Haben Sie Kopfschmerzen, Müdigkeit oder Übelkeit?	☐	☐	______________________
5.	Haben Sie andere Beschwerden?	☐	☐	______________________

7. Tag 6 nach der Impfung (d6) [_ _] [_ _] [20_ _]

		Ja	Nein	Weitere Angaben
1.	Haben Sie Schmerzen an der Impfstelle?	☐	☐	______________________
2.	Ist die Impfstelle gerötet oder geschwollen?	☐	☐	______________________
3.	Haben Sie Fieber, Schüttelfrost oder Gliederschmerzen?	☐	☐	______________________
4.	Haben Sie Kopfschmerzen, Müdigkeit oder Übelkeit?	☐	☐	______________________
5.	Haben Sie andere Beschwerden?	☐	☐	______________________

8. Tag 7 nach der Impfung (d7) [_ _] [_ _] [20_ _]

		Ja	Nein	Weitere Angaben
1.	Haben Sie Schmerzen an der Impfstelle?	☐	☐	______________________
2.	Ist die Impfstelle gerötet oder geschwollen?	☐	☐	______________________
3.	Haben Sie Fieber, Schüttelfrost oder Gliederschmerzen?	☐	☐	______________________
4.	Haben Sie Kopfschmerzen, Müdigkeit oder Übelkeit?	☐	☐	______________________
5.	Haben Sie andere Beschwerden?	☐	☐	______________________

9. Tag 8 nach der Impfung (d8) [_ _] [_ _] [20_ _]

		Ja	Nein	Weitere Angaben
1.	Haben Sie Schmerzen an der Impfstelle?	☐	☐	______________
2.	Ist die Impfstelle gerötet oder geschwollen?	☐	☐	______________
3.	Haben Sie Fieber, Schüttelfrost oder Gliederschmerzen?	☐	☐	______________
4.	Haben Sie Kopfschmerzen, Müdigkeit oder Übelkeit?	☐	☐	______________
5.	Haben Sie andere Beschwerden?	☐	☐	______________

10. Tag 9 nach der Impfung (d9) [_ _] [_ _] [20_ _]

		Ja	Nein	Weitere Angaben
1.	Haben Sie Schmerzen an der Impfstelle?	☐	☐	______________
2.	Ist die Impfstelle gerötet oder geschwollen?	☐	☐	______________
3.	Haben Sie Fieber, Schüttelfrost oder Gliederschmerzen?	☐	☐	______________
4.	Haben Sie Kopfschmerzen, Müdigkeit oder Übelkeit?	☐	☐	______________
5.	Haben Sie andere Beschwerden?	☐	☐	______________

11. Tag 10 nach der Impfung (d10) [_ _] [_ _] [20_ _]

		Ja	Nein	Weitere Angaben
1.	Haben Sie Schmerzen an der Impfstelle?	☐	☐	______________
2.	Ist die Impfstelle gerötet oder geschwollen?	☐	☐	______________
3.	Haben Sie Fieber, Schüttelfrost oder Gliederschmerzen?	☐	☐	______________

		Ja	Nein	Weitere Angaben
4.	Haben Sie Kopfschmerzen, Müdigkeit oder Übelkeit?	☐	☐	______________________
5.	Haben Sie andere Beschwerden?	☐	☐	______________________

12. Tag 11 nach der Impfung (d11) [_ _] [_ _] [20_ _]

		Ja	Nein	Weitere Angaben
1.	Haben Sie Schmerzen an der Impfstelle?	☐	☐	______________________
2.	Ist die Impfstelle gerötet oder geschwollen?	☐	☐	______________________
3.	Haben Sie Fieber, Schüttelfrost oder Gliederschmerzen?	☐	☐	______________________
4.	Haben Sie Kopfschmerzen, Müdigkeit oder Übelkeit?	☐	☐	______________________
5.	Haben Sie andere Beschwerden?	☐	☐	______________________

13. Tag 12 nach der Impfung (d12) [_ _] [_ _] [20_ _]

		Ja	Nein	Weitere Angaben
1.	Haben Sie Schmerzen an der Impfstelle?	☐	☐	______________________
2.	Ist die Impfstelle gerötet oder geschwollen?	☐	☐	______________________
3.	Haben Sie Fieber, Schüttelfrost oder Gliederschmerzen?	☐	☐	______________________
4.	Haben Sie Kopfschmerzen, Müdigkeit oder Übelkeit?	☐	☐	______________________
5.	Haben Sie andere Beschwerden?	☐	☐	______________________

14. Tag 13 nach der Impfung (d13) [_ _] [_ _] [20_ _]

		Ja	Nein	Weitere Angaben
1.	Haben Sie Schmerzen an der Impfstelle?	☐	☐	______________________

		Ja	Nein	Weitere Angaben
2.	Ist die Impfstelle gerötet oder geschwollen?	☐	☐	________________
3.	Haben Sie Fieber, Schüttelfrost oder Gliederschmerzen?	☐	☐	________________
4.	Haben Sie Kopfschmerzen, Müdigkeit oder Übelkeit?	☐	☐	________________
5.	Haben Sie andere Beschwerden?	☐	☐	________________

15. Tag 14 nach der Impfung (d14) [_ _] [_ _] [20_ _]

		Ja	Nein	Weitere Angaben
1.	Haben Sie Schmerzen an der Impfstelle?	☐	☐	________________
2.	Ist die Impfstelle gerötet oder geschwollen?	☐	☐	________________
3.	Haben Sie Fieber, Schüttelfrost oder Gliederschmerzen?	☐	☐	________________
4.	Haben Sie Kopfschmerzen, Müdigkeit oder Übelkeit?	☐	☐	________________
5.	Haben Sie andere Beschwerden?	☐	☐	________________

16. Tag 15 nach der Impfung (d15) [_ _] [_ _] [20_ _]

		Ja	Nein	Weitere Angaben
1.	Haben Sie Schmerzen an der Impfstelle?	☐	☐	________________
2.	Ist die Impfstelle gerötet oder geschwollen?	☐	☐	________________
3.	Haben Sie Fieber, Schüttelfrost oder Gliederschmerzen?	☐	☐	________________
4.	Haben Sie Kopfschmerzen, Müdigkeit oder Übelkeit?	☐	☐	________________

5.	Haben Sie andere Beschwerden?	☐	☐	____________

17. Tag 16 nach der Impfung (d16) [_ _] [_ _] [20_ _]

		Ja	Nein	Weitere Angaben
1.	Haben Sie Schmerzen an der Impfstelle?	☐	☐	____________
2.	Ist die Impfstelle gerötet oder geschwollen?	☐	☐	____________
3.	Haben Sie Fieber, Schüttelfrost oder Gliederschmerzen?	☐	☐	____________
4.	Haben Sie Kopfschmerzen, Müdigkeit oder Übelkeit?	☐	☐	____________
5.	Haben Sie andere Beschwerden?	☐	☐	____________

18. Tag 17 nach der Impfung (d17) [_ _] [_ _] [20_ _]

		Ja	Nein	Weitere Angaben
1.	Haben Sie Schmerzen an der Impfstelle?	☐	☐	____________
2.	Ist die Impfstelle gerötet oder geschwollen?	☐	☐	____________
3.	Haben Sie Fieber, Schüttelfrost oder Gliederschmerzen?	☐	☐	____________
4.	Haben Sie Kopfschmerzen, Müdigkeit oder Übelkeit?	☐	☐	____________
5.	Haben Sie andere Beschwerden?	☐	☐	____________

19. Tag 18 nach der Impfung (d18) [_ _] [_ _] [20_ _]

		Ja	Nein	Weitere Angaben
1.	Haben Sie Schmerzen an der Impfstelle?	☐	☐	____________
2.	Ist die Impfstelle gerötet oder geschwollen?	☐	☐	____________

3.	Haben Sie Fieber, Schüttelfrost oder Gliederschmerzen?	☐	☐	______________________
4.	Haben Sie Kopfschmerzen, Müdigkeit oder Übelkeit?	☐	☐	______________________
5.	Haben Sie andere Beschwerden?	☐	☐	______________________

20. Tag 19 nach der Impfung (d19) [_ _] [_ _] [20_ _]

		Ja	Nein	Weitere Angaben
1.	Haben Sie Schmerzen an der Impfstelle?	☐	☐	______________________
2.	Ist die Impfstelle gerötet oder geschwollen?	☐	☐	______________________
3.	Haben Sie Fieber, Schüttelfrost oder Gliederschmerzen?	☐	☐	______________________
4.	Haben Sie Kopfschmerzen, Müdigkeit oder Übelkeit?	☐	☐	______________________
5.	Haben Sie andere Beschwerden?	☐	☐	______________________

21. Tag 20 nach der Impfung (d20) [_ _] [_ _] [20_ _]

		Ja	Nein	Weitere Angaben
1.	Haben Sie Schmerzen an der Impfstelle?	☐	☐	______________________
2.	Ist die Impfstelle gerötet oder geschwollen?	☐	☐	______________________
3.	Haben Sie Fieber, Schüttelfrost oder Gliederschmerzen?	☐	☐	______________________
4.	Haben Sie Kopfschmerzen, Müdigkeit oder Übelkeit?	☐	☐	______________________
5.	Haben Sie andere Beschwerden?	☐	☐	______________________

22. Tag 21 nach der Impfung (d21) [_ _] [_ _] [20_ _]

		Ja	Nein	Weitere Angaben
1.	Haben Sie Schmerzen an der Impfstelle?	☐	☐	______________________
2.	Ist die Impfstelle gerötet oder geschwollen?	☐	☐	______________________
3.	Haben Sie Fieber, Schüttelfrost oder Gliederschmerzen?	☐	☐	______________________
4.	Haben Sie Kopfschmerzen, Müdigkeit oder Übelkeit?	☐	☐	______________________
5.	Haben Sie andere Beschwerden?	☐	☐	______________________

23. Tag 22 nach der Impfung (d22) [_ _] [_ _] [20_ _]

		Ja	Nein	Weitere Angaben
1.	Haben Sie Schmerzen an der Impfstelle?	☐	☐	______________________
2.	Ist die Impfstelle gerötet oder geschwollen?	☐	☐	______________________
3.	Haben Sie Fieber, Schüttelfrost oder Gliederschmerzen?	☐	☐	______________________
4.	Haben Sie Kopfschmerzen, Müdigkeit oder Übelkeit?	☐	☐	______________________
5.	Haben Sie andere Beschwerden?	☐	☐	______________________

24. Tag 23 nach der Impfung (d23) [_ _] [_ _] [20_ _]

		Ja	Nein	Weitere Angaben
1.	Haben Sie Schmerzen an der Impfstelle?	☐	☐	______________________
2.	Ist die Impfstelle gerötet oder geschwollen?	☐	☐	______________________
3.	Haben Sie Fieber, Schüttelfrost oder Gliederschmerzen?	☐	☐	______________________

4.	Haben Sie Kopfschmerzen, Müdigkeit oder Übelkeit?	☐	☐	________________
5.	Haben Sie andere Beschwerden?	☐	☐	________________

25. Tag 24 nach der Impfung (d24) [_ _] [_ _] [20_ _]

		Ja	Nein	Weitere Angaben
1.	Haben Sie Schmerzen an der Impfstelle?	☐	☐	________________
2.	Ist die Impfstelle gerötet oder geschwollen?	☐	☐	________________
3.	Haben Sie Fieber, Schüttelfrost oder Gliederschmerzen?	☐	☐	________________
4.	Haben Sie Kopfschmerzen, Müdigkeit oder Übelkeit?	☐	☐	________________
5.	Haben Sie andere Beschwerden?	☐	☐	________________

26. Tag 25 nach der Impfung (d25) [_ _] [_ _] [20_ _]

		Ja	Nein	Weitere Angaben
1.	Haben Sie Schmerzen an der Impfstelle?	☐	☐	________________
2.	Ist die Impfstelle gerötet oder geschwollen?	☐	☐	________________
3.	Haben Sie Fieber, Schüttelfrost oder Gliederschmerzen?	☐	☐	________________
4.	Haben Sie Kopfschmerzen, Müdigkeit oder Übelkeit?	☐	☐	________________
5.	Haben Sie andere Beschwerden?	☐	☐	________________

27. Tag 26 nach der Impfung (d26) [_ _] [_ _] [20_ _]

		Ja	Nein	Weitere Angaben
1.	Haben Sie Schmerzen an der Impfstelle?	☐	☐	________________

2.	Ist die Impfstelle gerötet oder geschwollen?	☐	☐	______________________
3.	Haben Sie Fieber, Schüttelfrost oder Gliederschmerzen?	☐	☐	______________________
4.	Haben Sie Kopfschmerzen, Müdigkeit oder Übelkeit?	☐	☐	______________________
5.	Haben Sie andere Beschwerden?	☐	☐	______________________

28. Tag 27 nach der Impfung (d27) [_ _] [_ _] [20_ _]

		Ja	Nein	Weitere Angaben
1.	Haben Sie Schmerzen an der Impfstelle?	☐	☐	______________________
2.	Ist die Impfstelle gerötet oder geschwollen?	☐	☐	______________________
3.	Haben Sie Fieber, Schüttelfrost oder Gliederschmerzen?	☐	☐	______________________
4.	Haben Sie Kopfschmerzen, Müdigkeit oder Übelkeit?	☐	☐	______________________
5.	Haben Sie andere Beschwerden?	☐	☐	______________________

14.4. Volunteer card

[redacted]

Studienkarte

Patientenname ____

Nimmt an der **P-pVac-SARS-CoV-2 Studie** zur Evaluation eines SARS-CoV-2 Impfstoff teil und wurde einmalig mit dem Impfstoff behandelt.

Bitte kontaktieren Sie im Notfall:

____

____

Bitte tragen Sie diese Notfallkarte immer bei sich

14.5. Intensity of solicited and unsolicited local and systemic adverse events

Local solicited AEs	CTCAE Term	Grade 0<br>Normal	Grade 1<br>Mild	Grade 2<br>Moderate	Grade 3<br>Severe	Grade 4<br>Potentially life-threatening
**Erythema**	Injection site reaction	< 25 mm	25-50mm<br>Tenderness with or without associated symptoms (e.g., warmth, erythema, itching)	51-100mm<br>Pain; lipodystrophy; edema; phlebitis	> 100mm<br>Ulceration or necrosis; severe tissue damage; operative intervention indicated	Life-threatening consequences; urgent intervention indicated
**Swelling**		< 25 mm	25-50 mm and does not interfere with activity	> 50 mm or interferes with activity	Prevents daily activity	Necrosis
**Pain**	Injection site reaction	None	Tenderness with or without associated symptoms (e.g., warmth, erythema, itching)<br><br>Does not interfere with activity	Pain; lipodystrophy; edema; phlebitis<br><br>Interferes with activity	Ulceration or necrosis; severe tissue damage; operative intervention indicated<br><br>Prevents daily activity	Life-threatening consequences; urgent intervention indicated<br>Emergency room visit or hospitalization

Systemic solicited AEs	CTCAE Term	Grade 0	Grade 1	Grade 2	Grade 3	Grade 4
**Fever**		None	38.0° - 39.0°C	≥ 39.0° - 40.0°C	≥ 40.0°C for ≤ 24 hours	≥ 40.0°C for ≥ 24 hours
**Chills**		None	Mild sensation of cold; shivering; chattering of teeth	Moderate tremor of the entire body; narcotics indicated	Severe or prolonged, not responsive to narcotics	-
**Myalgia** (described to the subject as generalized muscle ches)		None	Mild pain	Moderate pain; limiting instrumental ADL	Severe pain; limiting self care ADL	-
**Arthralgie** (described to the subject as generalized joint aches)			Mild pain	Moderate pain; limiting instrumental ADL	Severe pain; limiting self care ADL	-
**Fatigue**			Fatigue relieved by rest	Fatigue not relieved by rest; limiting instrumental ADL	Fatigue not relieved by rest, limiting self care ADL	-
**Headache**		None	Mild pain	Moderate pain; limiting instrumental ADL	Severe pain; limiting self care ADL	-
**Gastrointestinal symptoms** (nausea, vomiting, abdominal pain, and/or diarrhea)	nausea	None	Loss of appetite without alteration in eating habits	Oral intake decreased without significant weight loss, dehydration or malnutrition	Inadequate oral caloric or fluid intake; tube feeding, TPN, or hospitalization indicated	-
	vomiting	None	Intervention not indicated	Outpatient IV hydration; medical intervention indicated	Tube feeding, TPN, or hospitalization indicated	Life-threatening consequences
	abdominal pain	None	Mild pain	Moderate pain; limiting instrumental ADL	Severe pain; limiting self care ADL	-
	diarrhea	None	Increase of <4 stools per day over baseline; mild increase in ostomy output compared to baseline	Increase of 4 - 6 stools per day over baseline; moderate increase in ostomy output compared to baseline; limiting instrumental ADL	Increase of ≥7 stools per day over baseline; hospitalization indicated; severe increase in ostomy output compared to baseline; limiting self care ADL	Life-threatening consequences; urgent intervention indicated

14.6. List of specific immune mediated diseases (pIMDs)

Neuroinflammatory disorders	Musculoskeletal disorders	Skin disorders	Liver disorder	Gastrointestinal disorders	Metabolic & endocrine disorders	Vasculitides	Others
Cranial nerve inflammatory disorders, including paralyses/paresis (e.g., Bell's palsy)	Systemic lupus erythematosus	Psoriasis	Autoimmune hepatitis	Crohn's disease	Autoimmune thyroiditis (including Hashimoto thyroiditis)	Large vessels vasculitis including: giant cell arteritis such as Takayasu's arteritis & temporal arteritis	Autoimmune haemolytic anaemia
Acute disseminated encephalomyelitis including site- specific variants: encephalitis, encephalomyelitis, myelitis, myeloradiculoneuritis, cerebellitis	Systemic sclerosis (with limited or diffuse cutaneous involvement)	Vitiligo	Primary biliary cirrhosis	Ulcerative colitis	Grave's or Basedow's disease		Autoimmune thrombocytopenia
Multiple sclerosis	Dermatomyositis	Erythema nodosum	Primary sclerosing cholangitis	Ulcerative proctitis	Diabetes mellitus type I		Antiphospholipid syndrome
Transverse myelitis	Polymyositis		Autoimmune cholangitis.	Celiac disease	Addison's disease		Pernicious anaemia
Optic neuritis	Anti-synthetase syndrome	Cutaneous lupus erythematosus					Raynaud's phenomenon
Narcolepsy	Rheumatoid arthritis	Alopecia areata					Uveitis
	Juvenile chronic arthritis (including Still's disease)	Lichen planus					Autoimmune myocarditis/cardiomyopathy
	Polymyalgia rheumatica	Sweet's syndrome					Sarcoidosis
	Psoriatic arthropathy	Morphoea					Stevens-Johnson syndrome
	Relapsing polychondritis						Sjögren's syndrome
Myasthenia gravis (including Lambert-Eaton myasthenic syndrome)	Mixed connective tissue disorder						Idiopathic pulmonary fibrosis
							Goodpasture syndrome
Immune mediated peripheral neuropathies and plexopathies, (including Guillain-Barré syndrome, Miller Fisher syndrome and other variants, chronic inflammatory demyelinating polyneuropathy, multifocal motor neuropathy and polyneuropathies associated with monoclonal gammopathy)	Spondyloarthritis, including ankylosing spondylitis, reactive arthritis (Reiter's Syndrome) and undifferentiated spondyloarthritis	Autoimmune bullous skin diseases (including pemphigus, pemphigoid & dermatitis herpetiformis)				Medium sized and/or small vessels vasculitis including: polyarteritis nodosa, Kawasaki's disease, microscopic polyangiitis, Wegener's granulomatosis, Churg–Strauss syndrome (allergic granulomatous angiitis), Buerger's disease (thromboangiitis obliterans), necrotising vasculitis & anti-neutrophil cytoplasmic antibody (ANCA) positive vasculitis (type unspecified), Henoch-Schonlein purpura, Behcet's syndrome, leukocytoclastic vasculitis	Autoimmune glomerulonephritis (including IgA nephropathy, glomerulonephritis rapidly progressive, membranous glomerulonephritis, membranoproliferative glomerulonephritis, & mesangioproliferative glomerulonephritis)

Adapted from Tavares Da Silva, F et al., Optimal approaches to data collection and analysis of potential immune mediated disorders in clinical trials of new vaccines, Vaccine, 2013 [117]

[redacted]

[redacted]

[redacted]

[redacted]

[redacted]

[redacted]

[redacted]

[redacted]

[redacted]

[redacted]

[redacted]

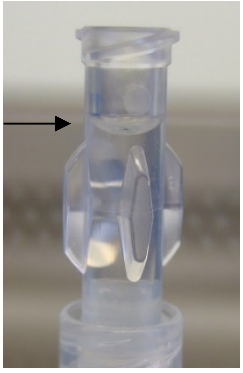

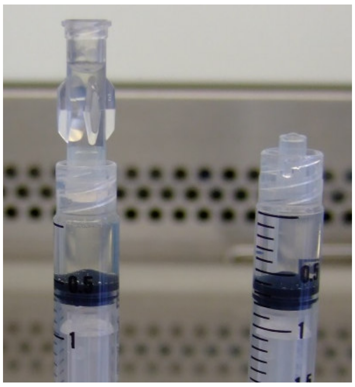

- [redacted]

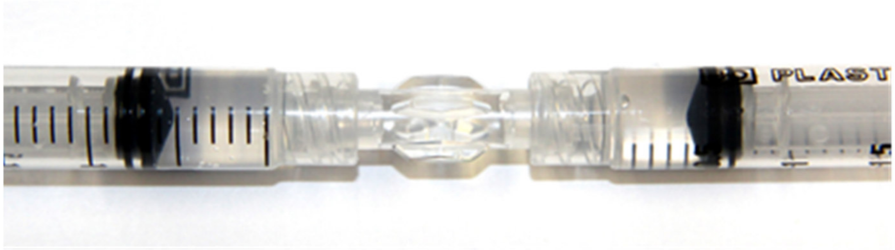

- [redacted]
  1. [redacted]
  2. [redacted]

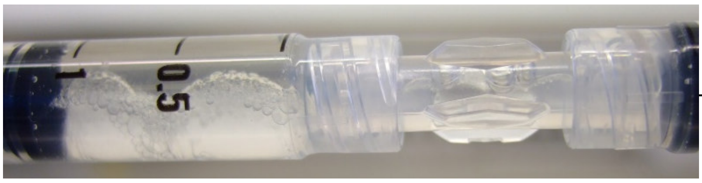

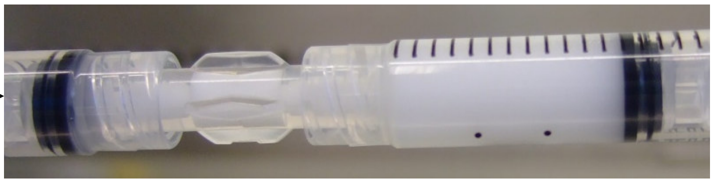

- [redacted]
- [redacted]

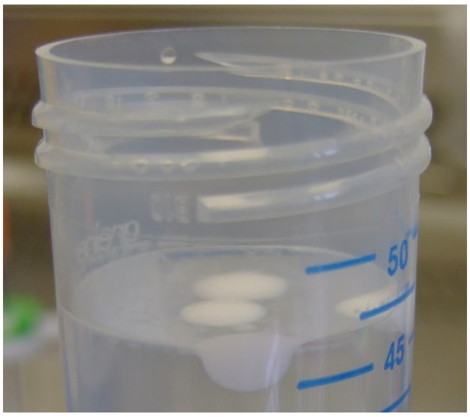

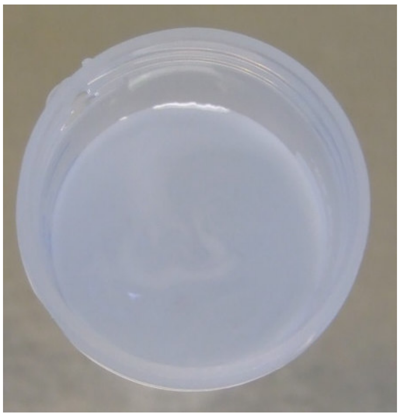

[redacted] [redacted]

[redacted]

[redacted]

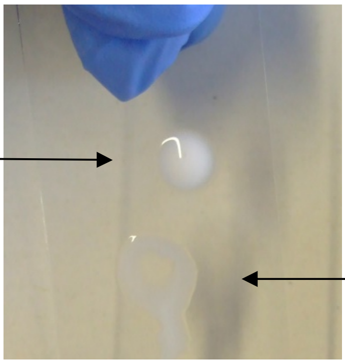

[redacted]

[redacted]

[redacted]

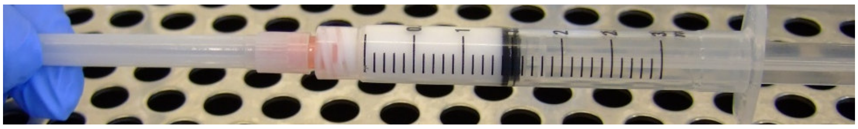

[redacted]

[redacted]

Document	Content
**Protocol**	V1.4, 08.03.2021
V. Synopsis	Indication:<br>Part II: Adults aged 56-80 years<br><br>Number of Volunteers:<br>Total number of volunteers: 36<br>Part I: 12<br>Part II:24<br><br>Inclusion Criteria:<br>Part II: Age 56-80 years at the time of screening<br>2.Part II: With or without pre-existing medical condition, not requiring change in therapy or hospitalization before enrollment<br><br>Description of the Medical Products<br>1.SARS-CoV-2 peptides: Six promiscuous HLA-DR-restricted peptides (250 µg each) derived from different proteins of<br>SARS-CoV-2<br><br>Study Design<br>Part II:<br>24 subjects will receive an open-label 500 µl subcutaneous injection via needle and syringe of the study IMP (CoVac-1).<br><br>Statistics, Safety Variables and Stopping Rules:
Table 1: Table of Events	7. Enrolment: volunteers are enrolled and registered through a screening procedure. Each volunteer
	will be registered under a specific Vol. ID on a subjects log kept at the trial site<br>21.Serological response: 10 ml of serum for analysis of serological response will be analysed by the Immunopathological
	Laboratory, University Hospital Tuebingen (central laboratory). Blood will be taken before peptide vaccination on V1, and during vaccination phase and follow-up at each visit.
1. Introduction	Novel findings on SARS-CoV-2 T cell immunity
	T cells play the central role in SARS-CoV-2 infection and COVID-19 disease. Early detection of SARS-CoV-2 specific CD4+ T cell responses has been correlated with a mild course of COVID-19, whereas high antibody levels were correlated with a more severe course of COVID-19. CD4 T cell levels negatively correlate with virus RNA loads. High diversity of SARS-

	CoV-2 specific T cell responses, i.e. the number of different SARS-CoV-2 T cell epitopes recognized by a subject's T cells, is correlated with a mild course of COVID 19 disease. Moreover, T cells are the central component of the immune system to build long-term immunity to SARS-CoV-2 and thus protection from virus re-exposure. Available reports, up to eight months after COVID-19, point towards a decrease and even loss of SARS-CoV-2-specific antibody responses and thus raise concerns regarding the protection achieved by humoral immunity, in contrast to maintained cellular/ T cell immunity. We could show that long-term T cell immunity is mediated by specific SARS-CoV-2 T cell epitopes, whereas T cell responses to other epitopes decreased or even got lost over time. Notably, T cells can combat COVID-19 even in the complete absence of a humoral i.e. antibody-mediated immune responses: Reportedly, two patients with X-linked agammaglobulinemia, a congenital B cell deficiency syndrome, recovered from moderate COVID-19 lung disease without requirement of SARS-CoV-2 specific treatment
1.1.3.1 Dose rationale for peptides	Preliminary data from a healthy volunteer and cancer patients vaccinated with a personalized peptide vaccine (240-300 µg per peptide) including two of the CoVac-1 peptides (250µg) in combination with XS15 showed potent induction of T-cell responses in 100% of HV and patients and a good safety profile.<br><br>The dose of ~250 µg per peptide per dose for CoVac-1 vaccine was selected based on these findings and on the feasibility in pharmaceutical development of the vaccines.
1.1.4 Rationale for trial design	• Part II: Adults aged 56-80. After proving safety and immunogenicity in a cohort of healthy volunteers aged 18-55 (Part I), an interim safety analysis will be conducted and prior to continuation with Part II approval by DSMB and of an amendment by PEI and Ethics Committee must be obtained.
1.1.5.Preliminary experiences from study Ppart I of the P-pVAC-SARS-CoV-2 study	P-pVAC-SARS-CoV-2 is a phase I single-center safety and immunogenicity trial of multi-peptide vaccination with CoVAC-1 to prevent COVID-19 infection in adults. The study is recruiting since November 2020 and has completed the first part (healthy volunteers (n=12), age 18-55 years) in February 2021. One single subcutaneous vaccination of CoVac-1 was applied. Immunogenicity, in term of induction of T-cell responses to one or more of the six HLA-DR SARS-CoV-2 T cell epitopes included in the CoVac-1 vaccine was assessed pre- vaccination as well as on day 7, 15 and 28 after vaccination (please refer to the IB of CoVac-1 for more details).<br><br>1.1.5.1. Safety and tolerability data after interim analysis (d28) of Part I of the study
	Preliminary safety data were assessed for all volunteers of Part I of the study (n = 12) after an interim safety follow-up visit (d28). Application of CoVac-1 revealed no relevant systemic side effects, in particular no fever or other systemic inflammatory reactions, no allergic reactions and no signs of vaccine-induced autoimmune disease. As expected and intended for Montanide ISA 51 VG-including vaccines, granuloma formation at the vaccination site was observed in all study subjects (max. grade 2 in 33% of subjects). Further local injection site adverse events were mild and included transient erythema, swelling, itching, pain and skin ulceration. The asymptomatic granulomas persisted until day 28 without affecting daily life activities, in particular the working ability, of study subjects.<br>An exemplary local site reaction is depicted in the IB (Appendix 9.5). For a detailed description of all ADRs, reported

please refer to section 6.8.1 of the IB.

1.1.5.2. Immunogenicity data after interim analysis (d28) of Part I of the study

Preliminary immunogenicity data were assessed of all volunteers of Part I of the study (n = 12) after the interim safety follow-up visit (d28). The single dose application of CoVac-1 revealed induction of T cell responses in 100% of vaccinated subjects (n = 12) at day 28 (Fig. 1). Induction of T cell responses was overserved at very early time points with 11/12 (93%) of subjects showing T cell responses already on day 14 after CoVac-1 vaccination. CoVac-1 induced a high diversity of T cell responses with median 5/6 vaccine peptides (range 4-6 peptides) recognized by T cells of the study subjects. CoVac-1-induced T cell responses were multifunctional with positivity for TNF (12/12 subjects), IFNγ (12/12 subjects) and IL-2 (11/12 subjects, Fig. 2). CoVac-1 induced a high frequency of functional SARS-CoV-2 T cells with up to 1.8% IFNγ+, 2.7% TNF+ and 2.5% IL-2+ SARS-CoV-2-specific T cells. In addition to CD4+ T cell responses, CoVac-1 also induced CD8+ T cell responses in 75% of donors. These CD8+ T cells targeting HLA class I T cell epitopes embedded in the CoVac-1 HLA-DR vaccine peptides were shown to be of pathophysiological relevance during natural SARS-CoV-2 infection. For a detailed description of interim immunogenicity data, please refer to section 6.2 of the IB.

1.1.5.3. Comparison of CoVac-1 to approved SARS-COV-2 vaccines (BNT126b2, Biontech SE; mRNA-12738, Moderna, Inc.; ChAdOx1, AstraZeneca)

Safety and tolerability

- In contrast to approved vaccine candidates (chills 32%, fever 14% BNT126b2, 50% chills, 8% fever mRNA-12738, chills 34%, fever 24% ChAdOx1 nCoV-19; AZD1222), no systemic inflammatory reactions were reported for CoVac-1
- No investigator-initiated drug treatment was required for CoVac-1-induced side effects, whereas paracetamol 1g post vaccination every 4-6 hours for 24 hours after vaccination was routinely advised for participants in the phase 2/3 ChAdOx1 nCoV-19 from Astra Zeneca to reduce possible reactogenicity from vaccination.
- None of the side effects reported for CoVac-1 vaccination affected daily life activity or working ability of study subjects. This is in stark contrast to the inflammatory side effects caused by approved vaccine candidates, in particular ChAdOx1 nCoV-19, which cause for example inability to work for up to 72h in a large proportion of vaccinated subjects
- Granuloma formation at the vaccination site was also reported, albeit rarely, in subjects after BNT162b2 vaccination. In contrast to CoVac-1 induced granulomas, these local reactions were indeed reported to affect subject’s daily life and also required specific treatment (e.g. steroids)97.

Vaccine design and immunogenicity

- In contrast to approved vaccine candidates, the peptide-based CoVac-1 vaccine includes validated SARS-CoV-2 T cell epitopes that were proven (i) to be frequently detected in convalescents after natural SARS-CoV-2 infection, (ii) to be of pathophysiological relevance for T cell immunity to combat COVID-19 and (iii) to mediate long-term immunity after infection. Thus, CoVac-1 is expected to induce strong and long-lasting SARS-CoV-2 T cell immunity that is comparable to T cell immunity after natural infection.
- In contrast to approved vaccine candidates that induce immune responses limited to the spike protein of SARS-

	CoV-2, CoVac-1 induces broad T cell immunity targeting multiple viral proteins (e.g. spike, nucleocapsid, membrane, envelope etc.). This is of particular importance in light of emerging mutations that challenge efficacy of current vaccines.<br>• In contrast to approved vaccine candidates that require two vaccinations, CoVac-1 induces strong T cell responses after one single vaccination.<br>• CoVac-1 induces earlier and stronger SARS-CoV-2 T cell responses after one single vaccination compared to the approved vaccine candidates. The detailed comparison of vaccine-induced SARS-CoV-2 T cell responses is provided in the IB section 6.2.
1.2. Benefit/Risk Assessment	1.2.1 Initial benefit and risk assessment<br>• The trial comprises two parts (cohorts of participants) with different age ranges to provide preliminary results on safety in a cohort of young (18-55 years, n=12) and healthy participants, which is then extended to older (Part II) participants. Of note, the risk of vaccine related (S)AEs is hypothesized to be similar in each age group.<br><br>• Confirming safety of the CoVac-1 vaccine in volunteers within the P-pVACSARS-CoV-2 study will further allow the transfer of this approach to induce SARSCoV-2 specific T-cell immunity in a therapeutic setting for patients with SARS-CoV-2 infection.
1.3. Risk and benefit analysis of CoVac-1 after interim safety and immunogenicity analyses of study subjects in Part I of P-pVAC-SARS-CoV-2	Benefits<br>The main goal of this study is to develop a vaccine candidate that induces superior SARS-CoV-2 T cell immunity to better combat COVID-19. It has been shown that T cells play an important role for COVID-19 disease outcome and are the central component of the immune system for maintaining long-term SARS-CoV-2 immunity. Thus, inducing broad and long-lasting SARS-CoV-2 T cell immunity is of utmost importance for COVID-19 vaccine development.<br>The vaccine candidate CoVac-1 was designed with the overarching aim to induce a strong and long-lasting SARS-CoV-2 T cell immunity after one single vaccination, that is comparable to T cell immunity acquired upon natural infection. In contrast to approved vaccine candidates, our peptide-based CoVac-1 vaccine includes validated SARS-CoV-2 T cell epitopes that were proven (i) to be frequently detected and in convalescents after natural SARS-CoV-2 infection, (ii) to be of pathophysiological relevance for T cell immunity to combat COVID-19 and (iii) to mediate long-term immunity after infection. Furthermore, and again in contrast to approved vaccines which only induce immune responses that are limited to the spike protein of SARS-CoV-2, CoVac-1 induces broad T cell immunity targeting multiple viral proteins (e.g. spike, nucleocapsid, membrane, envelope etc.). This is of special importance in the light of emerging mutations that challenge the efficacy of the currently available vaccines inducing immune responses limited to the spike protein.<br><br>Preliminary immunogenicity analyses on d28 in the study subjects included in Part I of our P-pVAC-SARS-CoV-2 study documented superior induction SARS-CoV-2 T cell immunity after one single CoVac-1 vaccination as compared to the approved vaccine candidates (BNT16B1, mRNA-1273 and ChAdOx1 nCoV-19), which all require a second booster vaccination. Of note, superiority of CoVac-1-induced T cell responses was shown in terms of multiple aspects: (i) diversity of T cell responses, (ii) frequency and intensity of functional SARS-CoV-2-specific T cells, and (iii) short time

	until occurrence of documented T cell.<br>These advantages of CoVac-1 are achieved without causing any systemic inflammatory side effects, e.g. fever or chills. Thus, in contrast to the approved vaccines, CoVac-1 does neither affect activities of daily life nor the working ability of study subjects.<br><br>Risks<br>The main (per definition) adverse event identified for CoVac-1 is the induction of a granuloma locally at injection site (max. grade 2 in 33% of subjects). These totally asymptomatic granulomas were still detectable on day 28 (time of interim safety analysis). However, it should be noted that granuloma development represents an expected and, even more, intended local reaction after vaccination that is required to enable the continuous local priming of SARS-CoV-2 specific T cells and thus the induction of long-lasting T cell responses while at the same time preventing systemic inflammation. Granuloma formation was also rarely reported after mRNA-based vaccines, where it required systemic steroid treatment. CoVac-1 induced granulomas, in contrast, did not require any investigator-initiated medication and did not affect the daily life activities, in particular the working ability, of our study subjects.<br><br>Conclusion<br>Together, in our view the available safety and immunogenicity data of CoVac-1 provide a profound rationale for the continued evaluation of CoVac-1 and thus conduct of the second part of the study. This is based, among others, on the comparison to the three vaccine candidates already approved by the EMA, which showed a clear superiority of CoVac-1 to induce SARS-CoV-2 specific T cell immunity, in terms of frequency, intensity and diversity of T cell responses. Thus, especially in the light of emerging mutations and concerns regarding long-term humoral immunity, CoVac-1 represents a highly promising vaccine candidate to combat COVID-19.
1.4 Data Safety Monitoring Board (DSMB)	The DSMB will receive a report listing and summarizing all the relevant safety data at least twice. The first assessment (first interim safety report, section 9.5) will take place after Part I of the trial including DSMB approval and an amendment at the regulatory authorities (Paul-Ehrlich Institute, PEI) and Ethics Committee (EC). If the IMP is considered safe for continuation by DSMB, Part II of the trial will start recruiting. After completion of Part II, the second DSMB report (second interim safety report, section 9.5) will be created and the DSMB has to approve continuation again. report will be made available for EC. In addition, the report will provide data concerning recruiting rates, status of the trial and AESIs (section 9.1.4); also non-occurrence will be mentioned. Based on its review, the DSMB will provide the sponsor with recommendations regarding trial modification and continuation or termination of the trial. An meeting of the DSMB may be called at any time should questions of volunteer safety arise or holding rules apply, and necessary safety reports will be provided. Meetings may be convened as conference calls/e-mail as well as in person.
3. Study Design	Part II and III must not start recruiting prior to approval by authorities. Volunteers of part II are treated simultaneously and 28 days following vaccination of the 12th volunteer, there will be an interim analysis of safety and a safety review by the DSMB whether to proceed to next Part III. Volunteers of part III are treated simultaneously (2 participants per day). Details can be found in figure 3.

4. Study Population	Healthy adult women and men aged 18-55 (Part I), followed by healthy adult women and men aged 56-74 56-80 with age adjusted health condition (Part II) and adult women and men aged ≥ 75 (Part III).
4.1.1. Inclusion Criteria	2. Part II: Age 56-80 years at the time of screening<br>3. 0. Part III: Age ≥ 75 years at the time of screening<br><br>Part I and II: Free of clinically significant health problems, as determined by pertinent medical history and clinical examination at study screening<br>Part II: With or without pre-existing medical condition, not requiring change in therapy or hospitalization before enrollment
4.1.2. Exclusion Criteria	16. Pre-existing auto-immune disease except for Hashimoto thyroiditis and mild (not requiring immunosuppressive treatment) psoriasis
5.1.1. Peptide cocktail	Each volunteer enrolled in the P-pVAC-SARS-CoV-2 trial will receive 6 promiscuous HLA-DR peptides (250 µg each) derived from different proteins of SARS-CoV-2. Details on drug substance can be found in Table 3
5.7 Dose Schedule	The mixing of the peptide vaccine cocktail and Montanide ISA 51 VG will be performed by local pharmacy and the investigator will be provided with a syringe containing the final drug product CoVac-1. A subcutaneous injection of 500 µl (approx. 250 µg per peptide, 50µg XS15) will be applied. A single vaccination per patient will be conducted.
5.7.2.1 Side effects of peptide vaccination	Preliminary safety results of volunteers (n = 12) in part I of the P-pVAC-SARS-CoV-2 study showed as intended and expected developed a local granuloma at injection site in all volunteers (100%). Further local injection site adverse events included transient erythema (100%), swelling (100%), itching (83%), pain (58%) and skin ulceration (8%). Until day 28 no relevant systemic side effects, especially no fever or other inflammatory reactions were reported. No allergic reactions were observed. In some participants fatigue (25%), headache (16%), nausea (16%), myalgia (8%) and arthralgia (8%) were reported.
5.7.2.3 Side effects of Montanide ISA 51 VG	Further side effects rarely reported were erythema nodosum (2/36 patients, 5%) and the development of sterile abscesses at injection site (10%)
6.3.2.Methods and Timing for Assessing, Recording, and Analysing of Efficacy Parameters	count well is at least 3 fold higher than the mean number of spots in the negative control wells<br><br>cancer immunoguiding program (CIP) guidelines).<br><br>Enzyme-linked immunosorbent assay (ELISA)<br><br>To differ between vaccine induced antibody response additional standard Elecsys® Anti-SARS-CoV-2 assay supplied by F. Hoffmann-La Roche AG, Basel, Switzerland or ADVIA Centaur SARS-CoV-2 Total (COV2T) (Siemens Healthcare Diagnostics GmbH) will be performed at central laboratory of the University Hospital Tuebingen.
6.5.Vaccination holding rules	The holding rules are as follow:<br>• Solicited local ADRs: If more than 30% of injections are followed by Grade ≥3 solicited swelling or pain or Grade 4 redness (first occurrence at any time after vaccination)beginning within 3 days after injection

	upon symptom severity and kinetics.
9.1.2. Adverse Drug Reaction	Local solicited ADRs:<br>• Swelling at site of injection<br>• Erythema at site of injection<br>• Pain or itching at site of injection<br>• Formation of granuloma at the injection site<br>• Superficial skin ulceration<br>A grading for severity of ADRs can be found in appendix 14.5 as guidance.

P-pVAC-SARS-CoV-2: Phase I single-center safety and immunogenicity trial of multi-peptide vaccination to prevent COVID-19 infection in adults

Short Title of Clinical Trial	P-pVAC-SARS-CoV-2
Protocol Version	V1.3
Date of Protocol	15.02.2021
EudraCT-Number	2020-002502-75
ClinicalTrials.gov-Number	
Phase	Phase I
Sponsor	University Hospital Tuebingen, [redacted]
Investigational Medicinal Product	Multi-peptide vaccine based on SARS-CoV-2 HLA class II peptides, applied subcutaneously together with the TLR1/2 ligand XS15 emulsified in Montanide ISA 51 VG
Summary of the revision history (amendments)	None

CONFIDENTIAL This protocol contains confidential information and is intended solely for the guidance of clinical investigation. This protocol may not be disclosed to parties not associated with the clinical investigation or used for any purpose without the prior written consent of the coordinating Investigator.

I. Table of Contents

Title Page 1
I. Table of Contents 2
I.a) List of Tables 7
I.b) List of Figures 8
II. Signature Page 9
III. Contacts 11
IV. Abbreviations 14
V. Synopsis 16
1. Introduction 29
1.1. Trial Rationale and Justification 32
1.1.1. Mechanism of action and rationale for a prophylactic SARS-CoV-2 multi-peptide vaccine 32
1.1.2. Rationale for the usage of XS15 as adjuvant in the prophylactic SARS-CoV-2 multi-peptide vaccine 33
1.1.3. Rationale for selected doses 34
1.1.3.1. Dose rationale for peptides 34
1.1.3.2. Dose rationale for XS15 35
1.1.3.3. Dose rationale for Montanide ISA 51 VG 36
1.1.3.4. Rationale for one dose schedule 36
1.1.4. Rationale for trial design 37
1.1.5. Preliminary experiences from study part I 38
1.2. Benefit / Risk Assessment 39
1.3. Data and Safety Monitoring Board (DSMB): 42
2. Study Objectives 44
2.1. Primary Objective and Endpoint 44
2.1.1. Primary Endpoint 44
2.2. Secondary Objectives and Endpoints 44

2.2.1. Secondary Endpoints 44

2.3. Exploratory Objectives and Endpoints 44

2.3.1. Exploratory Endpoints 45

3. Study Design 46

3.1. Study Duration and Schedule 48

3.2. End of Study 48

4. Study Population 49

4.1. General Criteria for Subject Selection 49

4.1.1. Inclusion Criteria 49

4.1.2. Exclusion Criteria 50

5. General Information on the Investigational Medical Product (IMP) 52

5.1. Peptide Vaccine CoVac-1 52

5.1.1. Peptide cocktail 52

5.1.1.1. SARS-CoV-2-specific peptides (drug substance) 52

5.1.1.1. TLR1/2 ligand XS15 (drug substance) 52

5.1.2. Montanide ISA 51 VG 53

5.2. Manufacturing of the Investigational Medicinal Product 55

5.2.1. SARS-CoV-2-specific peptides (drug substance) 55

5.2.2. XS15 (drug substance) 55

5.2.3. Montanide ISA 51 VG 55

5.2.4. Peptide cocktail CoVac-1 (drug product) 55

5.3. Labeling of the Investigational Medicinal Product 56

5.3.1. Peptide cocktail 56

5.3.2. Montanide ISA 51 VG 56

5.4. Storage of the Investigational Medicinal Product 56

5.5. Drug Accountability, Therapy Compliance and Disposal 57

5.6. Method of Treatment Assignment 57

5.7. Dose Schedule 58

5.7.1. Dose modifications for peptide vaccine 58

5.7.2. Side effects 58

5.7.2.1. Side effects of peptide vaccination 58

5.7.2.2. Side effects of XS15 59

5.7.2.3. Side effects of Montanide ISA 51 VG 59

6. Study Procedures and Examination Method 61

6.1. Study Entry 61

6.1.1. Volunteer's Informed Consent 61

6.1.2. Screening 61

6.1.3. Enrolment 62

6.1.4. Randomisation 62

6.1.5. Concomitant Medication and Treatments 62

6.1.6. Permitted Prior and Concomitant Medications and Treatments 62

6.1.7. Prohibited Prior and Concomitant Medications and Treatments 63

6.1.8. Contraception 63

6.2. Vaccination Phase 64

6.2.1. Visit 1 (Vaccination) (Day 1) 64

6.2.2. Visit 2 (Day 7 +/- 1) 65

6.2.3. Visit 3 (Day 14 +/- 1) 65

6.2.4. Visit 4 (Interim safety) (Day 28 +/- 2) 65

6.2.5. Visit 5 (End of Safety follow-up = EOSf) 66

6.2.6. Visit 6-7 (Follow-up) (Month 3 and 6 +/- 7 days) 66

6.2.7. Volunteer's diary/card 66

6.2.8. Unscheduled Visit 66

6.3. Assessment of Efficacy 67

6.3.1. Efficacy Parameters 67

6.3.2. Methods and Timing for Assessing, Recording, and Analysing of Efficacy Parameters 67

6.4. Assessment of Safety 69
6.4.1. Safety parameters 69
6.4.2. Methods and Timing for Assessing, Recording, and Analysing Safety Parameters 70
6.5. Vaccination holding rules 70
6.6. Premature termination of clinical trial for a trial subject 71
6.7. Premature closure of a trial site 72
6.8. Premature termination of the trial 72
6.9. Follow Up 73
6.10. End of Study for Subjects 73
7. Quality control and Quality assurance 74
7.1. Risk-based approach 74
7.2. Monitoring 74
7.3. Audits/ Inspections 75
7.4. Documentation: Collection, Handling, Storage and Archiving of Data 75
7.4.1. Case Report Form 75
7.4.2. Source Data 76
7.4.3. Data Handling 76
7.4.4. Preparation/Handling/Storage/Accountability of biological samples 76
7.4.5. Handling of missing data and drop outs 77
7.4.6. Storage and Archiving of Data 77
8. Statistical Analyses 78
8.1. Study Population Definition 78
8.1.1. Sample Size and Power Consideration 78
8.2. Analysis Primary Variables 78
8.3. Analysis Secondary Variables 78
8.4. Subgroup Analysis 79
8.5. Interim Analysis 79

8.6. Stopping Rules 79
8.7. Biometric Report 80
9. Safety 81
9.1. Definition of Adverse Events and Side Effects 81
9.1.1. Adverse Events 81
9.1.2. Adverse Drug Reaction 81
9.1.3. Expectedness 82
9.1.4. AESI (adverse events of special interest) 83
9.1.5. Serious Adverse Event and Serous Adverse Reaction 83
9.2. Period of Observation 84
9.3. Documentation and Reporting of Adverse Events 84
9.3.1. Documentation and Reporting of Adverse Events by the Investigator 84
9.3.2. Assessment of Severity and Causality 85
9.3.3. Action taken 86
9.3.4. Sponsors Assessment of the SAEs 86
9.3.5. Follow-up of Initial Report 86
9.3.6. Exception of reporting 87
9.3.7. Suspected Unexpected Serious Adverse Reaction (SUSAR) 87
9.3.8. Expedited Reporting to the Regulatory Authorities 87
9.4. Examination and Report of Changes in the Risk to Benefit Ratio 88
9.4.1. Reporting to Data and Safety Monitoring Board 88
9.4.2. Report to the Investigator 88
9.5. Interim Safety analysis 88
9.6. Annual Safety Report 89
9.7. Deviations from the Protocol 89
9.8. Reporting of Pregnancy 89
10. Regulatory Consideration 92
10.1. Ethical Conduct of Clinical Study 92

10.1.1. Good Clinical Practice, Declaration of Helsinki and legal Provision 92

10.2. Subject Information and Informed Consent 92

10.3. Insurance 92

10.4. Confidentiality 93

10.5. Responsibility of the Investigator 94

10.6. Registration of the Trial 95

10.7. Continuous Information to Independent Ethics Committee 95

10.8. Approval of Protocol and Subsequent Amendments 95

11. Publications 96

11.1. Reports 96

11.2. Publication 96

12. Financing 97

13. Literature 98

14. Appendix 106

14.1. Common Terminology Criteria for Adverse Events (CTCAE) Version 106

14.2. List of central laboratories 106

14.3. Volunteer diary 107

14.4. Volunteer card 118

14.5. Intensity of solicited and unsolicited local and systemic adverse events 119

14.6. List of specific immune mediated diseases (pIMDs) 121

14.7. “Mischanleitung“ for the pharmacy of participating centers 122

I.a) List of Tables

Table 1: Table of Events 27

Table 2: Study Timelines 48

Table 3: SARS-CoV-2 specific HLA-DR vaccine peptides 55

I.b) List of Figures

Figure 1: Overall Study Design 47

Figure 2: Individual Study Procedure 47

Figure 3: Treatment sequence 48

II. Signature Page

The present trial protocol was subject to critical review and has been approved in the present version by the persons signed.

Sponsor: The University Hospital Tuebingen is sponsor for the purpose of § 4 (24) German Drug Law with complementary regulations. The internal responsibility to comply with the obligations of the sponsor in terms of these regulations stays with [redacted]

ame: [redacted]

Function: Sponsor’s delegate and person in charge to meet the obligations of the sponsor

[redacted]

ame: [redacted]

Function: Biometrician

Declaration of the Principal Investigator

By my signature, I agree to supervise personally the conduct of this study at my study site and to ensure its conduct is in compliance with the protocol, informed consent, the national laws, the ICH Good Clinical Practices Guidelines and the Declaration of Helsinki. I will train the involved personal accordingly.

[redacted]

Name: [redacted]

Function: [redacted]

(AMG)

[redacted]

Name: [redacted]

Function: Deputy Principal Investigator

Adress of the Study Center: [redacted]

III. Contacts

Sponsor

Universitätsklinikum Tuebingen
Geissweg 3
72076 Tuebingen

Sponsor's Delegate

[redacted]
Phone: [redacted]
Fax: [redacted]
e-mail: [redacted]

Coordinating Investigator (CI)

Leiterin der klinischen Prüfung, according to § 4 German Drug Law (AMG)

[redacted]
Phone: [redacted]
Fax: [redacted]
e-mail: [redacted]

Co-Coordinating Investigator

[redacted]
Phone: [redacted]
Fax: [redacted]
e-mail: [redacted]

Scientific Coordinators

[redacted]
Phone: [redacted]
Fax: [redacted]

e-mail: [redacted]

[redacted]
Phone: [redacted]
Fax: [redacted]
e-mail: [redacted]

[redacted]

[redacted]
Phone: [redacted]
Fax: [redacted]
e-mail: [redacted]

Data management

[redacted]
Phone: [redacted]
Fax: [redacted]

Project management

[redacted]
Phone: [redacted]
Fax: [redacted]
e-mail: [redacted]

Monitoring

[redacted]
Phone: [redacted]
Fax: [redacted]
e-mail: [redacted]

SAE-Management

[redacted]
Phone: [redacted]
Fax: [redacted]
e-mail: [redacted]

IV. Abbreviations

ADR	Adverse Drug Reaction
ADE	Antibody-dependent Enhancement
ADL	Activities of Daily Living
ADV	Adenovirus
AE	Adverse Event
AESI	Adverse Event of Special Interest
AMG	German Drug Law (Deutsches Arzneimittelgesetz)
CCR	Cellular Conversion Rate
CI	Coordinating Investigator
CNS	Central Nervous System
COPD	Chronic Obstructive Pulmonary Disease
COVID-19	Coronavirus Disease 2019
COV	Coronavirus
CMV	Cytomegalovirus
CRF	Case Report Form
CTC(AE)	Common Toxicity Criteria (for Adverse Events)
CTR	Clinical trial report
DBL	Data Base Lock
DSMO	Dimethyl sulfoxide
DSMB	Data and Safety Monitoring Board
EBV	Epstein-Barr Virus
EC	Ethics Committee
EORTC	European Organisation for Research and Treatment of Cancer
EOSf	End of Safety follow-up
FCBP	Female of Child Bearing Potential
FSI	First Subject In
GCP	Good Clinical Practice
GCP-V	Good Clinical Practice Ordinance (GCP-Verordnung)
GMP	Good Manufacturing Practice
GMT	Geometric mean titer

HLA	Human Leukocyte Antigen System
HRT	Hormone Replacement Therapy
IB	Investigator’s Brochure
IC	Informed Consent
ICH	International Conference on Harmonization of Technical Requirements for Registration of Pharmaceuticals for Human Use
ICU	Intensive Care Unit
IMP	Investigational Medicinal Product
ISF	Investigator Site File
LSI	Last Subject In
LSO	Last Subject Out
MERS-CoV	Middle East Respiratory Syndrome Coronavirus
PCR	Polymerase Chain Reaction
PBMC	Peripheral Blood Mononuclear Cell
PEI	Paul-Ehrlich-Institut
pIMD	Potential Immune Mediated Disease
RNA	Ribonucleic acid
SARS-CoV-2	Severe Acute Respiratory Syndrome - Coronavirus 2
SAE	Serious Adverse Event
SmPC	Summary of Product Characteristics (deutsch: Fachinformation)
SDV	Source Data Verification
SOP	Standard Operating Procedure
SPC	Summary of Product Characteristics
SUSAR	Suspected Unexpected Serious Adverse Reaction
TLR	Toll-like receptor
TMF	Trial Master File

V. Synopsis

Sponsor	University Hospital of Tuebingen represented by<br>Medical Director: Prof. Dr. med. M. Bamberg<br>Director of Administration: G. Sonntag
Title	P-pVAC-SARS-CoV-2: Phase I single center safety and immungenicity trial of multi-peptide vaccination to prevent COVID-19 infection in adults
Short Title	P-pVAC-SARS-CoV-2
Coordinating Investigator<br>(Leiter der klinischen Prüfung, According to § 4 German Drug Law (AMG))<br><br>Co-Coordinating Investigator<br><br>Sponsor's Delegate<br><br>Scientific Coordinator	[redacted]<br><br><br>[redacted]<br><br>[redacted]<br><br>[redacted]
Indication	Part I: Adults aged 18-55 years<br>Part II: Adults aged > 55 years
Number of Volunteers	Total number of volunteers: 36<br>Part I: 12<br>Part II: 24

Inclusion Criteria	1. Adult male or non-pregnant, non-lactating female<br>1. Part I: Age 18-55 at the time of screening<br>2. Part II: Age > 55 years at the time of screening<br>2. Pre-existing medical condition<br>1. Part I: Free of clinically significant health problems, as determined by pertinent medical history and clinical examination at study screening<br>2. Part II: With or without pre-existing medical condition, not requiring change in therapy or hospitalization before enrollment<br>3. Ability to understand and voluntarily sign an informed consent form<br>4. Ability to adhere to the study visit schedule and other protocol requirements<br>5. Female volunteers of child bearing potential (FCBP) and male volunteers with partners of child bearing potential, who are sexually active, must agree to the use of two effective forms (at least one highly effective method) of contraception. This should be started from the signing of the informed consent and continue until three months after vaccination

Inclusion criteria	6. Postmenopausal or evidence of non-child-bearing status. For women of childbearing potential: negative urine or serum pregnancy test within 7 days prior to study treatment. Postmenopausal or evidence of non-childbearing status is defined as:<br>1. Amenorrhoea for 1 year or more following cessation of exogenous hormonal treatments<br>2. Luteinizing hormone (LH) and Follicle stimulating hormone (FSH) levels in the postmenopausal range for women under 50<br>7. Be willing to minimize blood and body fluid exposure from others for 7 days after vaccination<br>1. Use of effective barrier prophylaxis, such as latex condoms, during sexual intercourse<br>2. Avoiding the sharing of needles, razors, or toothbrushes<br>3. Avoiding open-mouth kissing<br>8. Refrain from blood donation during the course of the study

Exclusion Criteria	1. Pregnant or lactating females<br>2. Participation in any clinical study with intake of any investigational drug interfering with the study primary endpoint including:<br>o Active infection<br>o Psychatric disorders<br>o Known systemic anaphylaxis<br>3. Any concomitant disease affecting the effect of the therapeutic vaccine or interfering with the study primary endpoint<br>4. Any immunosuppressive treatment except low dose corticosteroids (equivalent to ≤10mg prednisolone/day)<br>5. Prior or current infection with SARS-CoV-2 tested serologically or by throat/nose swab (PCR)<br>6. History of Guillain-Barré syndrome<br>7. Positive serological HIV, hepatitis B or C test. In case of positive HBsAg, volunteer must provide prove of hepatitis B vaccination, otherwise volunteer must be excluded.<br>8. History of relevant CNS pathology or current relevant CNS pathology (e.g. seizure, paresis, aphasia, cerebrovascular ischemia/haemorrhage, severe brain injuries, dementia, Parkinson’s disease, cerebellar disease, organic brain syndrome, psychosis, coordination or movement disorder, excluding febrile seizures as child)<br>9. Baseline laboratory with lymphocyte count ≤ 1000/µl<br>10. <u>Only Part I</u><br>o Acute or chronic, clinically significant psychiatric, hematologic, pulmonary, cardiovascular, or hepatic or renal functional abnormality as determined by the Investigator based on medical history, physical exam, and/or laboratory screening test

11. All parts of the clinical trial
    - o Diabetes mellitus Typ II requiring drug treatment
    - o Chronic lung disease requiring drug treatment
    - o Any chronic liver disease or unknown liver abnormalities defined as:
        - ALT and AST ≤ 2.5 x ULN
        - γ-GT ≤ 2.5 x ULN
    - o Chronic renal failure defined as GFR < 60 ml/min/1,73m^2
    - o Serious pre-existing cardiovascular disease such as NYHA ≥ I, coronary heart disease requiring coronary surgery or known pAVK ≥ grade 2
    - o Sickle cell anemia
    - o Obesity (as defined by age adjusted body mass index)
12. Hospitalization at study inclusion
13. Administration of immunoglobulins and/or any blood products within the 120 days preceding study entry or planned administration during the study period
14. History of blood donation within 30 days of enrolment or planned donations within the study period
15. Known hypersensitivity to any of the components included in the CoVac-1 vaccine
16. Pre-existing auto-immune disease except for Hashimoto thyroiditis and mild (not requiring immunosuppressive treatment) psoriasis

Description of the Medical Products	IMP/Drug product/Peptide vaccine: CoVac-1 applied as one multipeptide cocktails consisting of:<br>1. SARS-CoV-2 peptides: Six promiscuous HLA-DR-restricted peptides (250 µg each) derived from different proteins of SARS-CoV-2<br>2. XS15: The lipopeptide XS15 is a water-soluble synthetic Pam_3Cys-derivative. As TLR1/2 ligand it will be included as an adjuvant in the peptide vaccine.<br>Peptides are synthesized in the GMP-certified Wirkstoffpeptidlabor at the University of Tuebingen (Prof. Stefan Stevanović) and will be formulated at the GMP-Center of the University Hospital Tuebingen. The GMP-certified Wirkstoffpeptidlabor specializes in multipeptide cocktails with variable composition and holds a production permit (Herstellungserlaubnis) for different multipeptide cocktails including the TLR 1/2 ligand XS15.<br>3. Montanide ISA 51 VG: Prior to application, the peptide cocktail (consisting of 6 SARS-CoV-2-derived peptides and XS15) will be emulsified in a water-oil emulsion 1:1 with Montanide ISA 51 VG to a final volume of 500 µl.
	Treatment schedule:<br>A single vaccination with the IMP CoVac-1 (SARS-CoV-2 HLA-DR peptides, XS15 emulsified in Montanide ISA 51 VG) (500 µl) will be applied subcutaneously (s.c.) to the abdominal skin.

Study Design:	Single center Phase I clinical trial<br>Part I:<br>12 subjects will receive an open-label 500 µl subcutaneous injection via needle and syringe of the study IMP (CoVac-1). No more than one subject per day will be enrolled. 28 days following vaccination of the 12th volunteer, there will be an interim analysis of safety and a safety review by the data safety monitoring board (DSMB) as well as an amendment to the regulatory authorities (Paul-Ehrlich Institute and Ethics Committee) before proceeding to Part II.<br>Part II:<br>24 subjects will receive an open-label 500 µl subcutaneous injection via needle and syringe of the study IMP (CoVac-1).
Aim of the Study	To evaluate the safety and immunogenicity of a single use of a SARS-CoV-2-derived multi-peptide vaccine in combination with the TLR1/2 ligand XS15 in adults

Objectives/Endpoints	Primary endpoint:<br>• The nature, frequency, and severity of AEs and/or SAEs associated with administration of CoVac-1:<br>• Solicited: ADRs/AEs occurring from the time of each injection throughout 28 days following the procedure, facilitated by use of a volunteer diary<br>• Unsolicited: AEs from the time of injection throughout 56 days following injection<br>• SAEs from the time of injection until the final study visit for each subject<br>• Incidence of AESIs until the final study visit for each subject<br>Secondary endpoints:<br>• Development of a CoVac-1 specific T-cell response to at least one of the single SARS-CoV-2 T-cell epitopes included in the CoVac-1 vaccine on Visits 2, 3, 4, 5 measured by IFN-γ ELISpot ex vivo and after in vitro T-cell amplification (compared to Visit 1), this includes:<br>• Cellular conversion rate (CCR) at Visits 2, 3, 4, 5 after immunization

	<u>Explorative endpoints:</u><br>• Characteristics of T-cell response on Visits 2, 3, 4, 5 measured by ELISpot/ICS. This includes:<br>- Phenotyping of SARS-CoV-2 specific T-cells (CD4, CD8 etc.) by flow cytometry<br>- Characterization of cytokine profiles of SARS-CoV-2 specific T cells (TNF, IFN, IL-2, CD107a etc.) by intracellular cytokine staining<br>- Recognition rate defined as percentage of peptides inducing a T cell response in one individual<br>- Intensity of T cell response to a single SARS-CoV-2 T cell epitope included in the CoVac-1 vaccine<br>• Induction of long-term SARS-CoV-2 specific T-cell responses 3 and 6 months after peptide vaccination.<br>• Induction of antibodies specific to the SARS-CoV-2 T-cell epitopes included in the CoVac-1 vaccine<br>In case of unexpected detection of CoVac-1 specific antibodies the following assays will be performed:<br>- Individual neutralization antibody titers<br>- Seroconversion rates<br>- Calculation of geometric mean titers (GMT) for neutralizing and binding antibodies<br>• Biomarkers and clinical characteristics influencing immunogenicity.

Statistics, Safety Variables and Stopping Rules	**Safety**:<br>In this phase I study the safety/toxicity of one vaccination will be investigated. For this purpose, it will be investigated whether the incidence of severe adverse events (SAE) associated with administration of CoVac-1 exceeds a predetermined rate of 5% (= P1 = alternative hypothesis) in the whole study population. Safety of the CoVac-1 vaccine is shown if no SAE (= P0 = null hypothesis) occurs in the study population. An evaluable sample size of 33 achieves 81.6% power to detect a difference (P1-P0) of 0.0499 using a one-sided exact test based on the binomial distribution with a target significance level of 0.05. The actual significance level achieved by this test is 0.003. These results assume that the population proportion under the null hypotheses (P0) is 0.0001. Assuming a dropout rate of 7.5% (percentage of subjects that are expected to be lost at random during the course of the study and for whom no response data concerning existence of SAE will be collected, i.e. will be treated as "missing") the total number of 36 subjects should be enrolled in the study in order to end up with 33 evaluable subjects. Sample size computed using PASS 2020 (NCSS, LLC, Kaysville, Utah, USA).<br><br>**Sample size:** 36<br>Part I:<br>n=12<br>Interim Safety Analysis after Part I and a substantial amendment to authorities<br>Part II:<br>n=24
Database	A validated GCP conform clinical trial database hosted by the IKEAB Tuebingen (SecuTrial) will be used for data capture and validation in this trial
Participating Centers and Investigators	CCU Translational Immunology, Department of Internal Medicine, University Hospital Tuebingen, [redacted])
Study Type	• AMG
Competent Regulatory Authorities	• PEI and EC

Monitoring according GCP	Monitoring of the clinical trial will be performed by the ZKS Tuebingen.
Study duration	Total study duration for individual volunteer: 6 months<br>Safety duration for individual volunteer: 8 weeks<br>Follow up (exploratory end points) for individual volunteer:<br>4 months
Length of Study/ Time Lines	Total trial duration: 1 years<br>Duration for individual patient: Safety follow-up: 8 weeks<br>Follow-up: 4 months<br>Number of visits: 8<br>FSI (First Subject In): Q4/2020<br>LSI (Last Subject In): Q1/2021<br>LSO (Last Subject Out): Q3/2021<br>DBL (Data Base Lock): Q3/2021<br>Statistical Analyses Completed: Q4/2021<br>*Trial Report Completed: Q4/2021*

Table 1: Table of Events

Protocol activities and forms to be completed	Screening	Vaccination phase[1]					Follow-up period[2]
					Interim Safety	EOSf	
	≤ - 7 days	Day 1	Day 7 +/- 1 days	Day 14 +/- 1 days	Day 28 +/- 2 days	Day 56 +/- 2 days	3 and 6 months after peptide vaccination
Visit		V1	V2	V3	V4	V5	V6-7
Informed consent[3]	X						
Demographics[4]	X						
Medical history[5]	X						X
Signs/symptoms[6]		X	X	X	X	X	
Enrolment[7]	X						
	**Clinical assessments**						
Vital signs[8]	X	X	X	X	X		
Physical examination[9]	X	X	X	X	X		
Assessment of concomitant medications[10]	X	X	X	X	X	X	
AE assessments[11]		X	X	X	X	X	X
	**Laboratory assessments**						
Hematology (*local lab)*[12]	X	X	X	X	X	X	
Blood chemistry and coagulation (*local lab)*[13]	X	X	X	X	X	X	
Immunoglobulins/Immunophenotype[14]	X						
Urine analysis (*local lab)*[15]	X						
HBV, HCV, HIV-1, (*local lab)*[16]	X						
Pregnancy test[17]	X						
SARS-CoV-2 testing	X[18]						
	**Treatment**						
Vaccine CoVac-1[19]		X					
	**Efficacy assessment**						
T-cell response[20]		X	X	X	X	X	X
Serological response[21]		X	X	X	X	X	X

Detailed information on schedule and activities are described in the footnotes.

1. The peptide vaccination should be applied as early as possible after screening (max. 7 days) and approved eligibility of the volunteer. Vaccination phase will be 2 months and ends with the end of safety follow-up (EOSf).
2. Follow-up: After vaccination phase, volunteers will enter follow-up, which ends with the last visit 6 months after vaccination (V7, EOS).
3. Informed consent and volunteer registration: every volunteer must date and sign informed consent form to participate in this trial before starting any trial-related procedures.
4. Demographics: gender, year of birth, ethnicity
5. Medical history: The investigator has to collect information on the volunteers' medical history including prior illnesses, hospitalisations, and symptoms of a SARS-CoV-2 infection.
6. Signs/symptoms: vaccine-related and -unrelated signs and symptoms

7. Enrolment: volunteers are enrolled and registered through a screening procedure. Each volunteer will be registered under a specific Vol. ID on a subjects log kept at the trial site.
8. Vital signs: At all visits: ECOG, temperature (in grade centigrade), blood pressure/pulse. At baseline additionally: height (in cm) and weight (in kg). At V4 and V5 additionally: weight (in kg). For detailed surveillance after vaccination, please refer to section 6.2 of the study protocol
9. Physical examination: inspection, abdominal, cardiac and lung auscultation, palpation of the abdomen and lymph node sites, neurological examination, inspection of vaccination site.
10. Concomitant medications should be reported in the respective CRF pages, including drugs used for treating AEs or, if applicable, chronic diseases.
11. AE assessments: events should be documented and recorded continuously. Volunteers have to be followed for AEs from application up to 56 days or until all drug-related toxicities have been resolved, whichever is later, or until the investigator assesses AEs as “chronic” or “stable”. Each AE must be reported indicating the CTC (Version 5.0) grade. If an event stops and later restarts or CTC grading changes, all occurrences must be reported. A specific procedure for definition and reporting of SAEs is described in the protocol.
12. Hematology (local lab): hemoglobin (Hb), red blood cells (RBC), platelet count (PLT) white blood cells (WBC). Differential cell counts should be performed at baseline, at each visit during vaccination phase and thereafter at investigators discretion. Clinical status and laboratory parameters are to be followed using individual institutional guidelines and the best clinical judgment of the responsible physician, which can involve more frequent testing.
13. Blood chemistry and coagulation (local lab): Alkaline phosphatase (AP), total bilirubin, aspartate transaminase (AST/ SGOT), alanine transaminase (ALT/ SGPT), lactate dehydrogenase (LDH), and uric acid, C-reactive protein (CRP), sodium, potassium, calcium, blood urea nitrogen, creatinine, glucose: at baseline and during vaccination phase, thereafter at each visit using individual institutional guidelines and the best clinical judgment of the responsible physician, which can involve more frequent testing. Prothrombin time, aPTT, and fibrinogen will be measured at baseline and at investigator’s discretion during treatment.
14. Immunoglobulin/immunophenotype: Assessment of IgA, IgG and IgM; lymphocyte subsets: T ($CD4^+$ and $CD8^+$) as well as B and NK cells.
15. Urine analysis (local lab): pH, glucose, proteins (qualitative, dipstick accepted): at baseline and at investigator’s discretion during treatment
16. HBV, HCV and HIV-1: at baseline and thereafter at investigator’s discretion
17. Pregnancy testing: For all FCBP, pregnancy testing has to be performed at the screening visit. Negative results must be available prior to vaccination.
18. SARS-CoV-2 testing: Volunteer must be tested for prior or current SARS-CoV-2 infection. Patients should be tested by serological test and throat/nose swab. If screening takes more than 48 hours, throat/nose swab for SARS-CoV-2 infection must be repeated. The vaccine can only be applied if a negative SARS-CoV-2 PCR test is available on the day of vaccination not older than 48 hours.If patients develop SARS-CoV-2 typical symptoms until vaccination, testing should be repeated.
19. Vaccine CoVac-1: Peptide vaccination should be started as soon as possible after the screening visit. Peptide vaccination will be performed once.
20. T-cell response: 60 ml of heparin blood for immunomonitoring and analysis of peptide specific T-cell response will be analyzed by the Walz lab, KKE Translational Immunologie at the Department of Immunology, Tuebingen (central laboratory). Blood will be taken before peptide vaccination on V1, and during vaccination phase and follow-up at each visit.
21. Serological response: 10 ml of serum for analysis of serological response will be analysed by the Immunopathological Laboratory, University Hospital Tuebingen (central laboratory). Blood will be taken before peptide vaccination on V1, and during vaccination phase and follow-up at each visit.

1. Introduction

The novel coronavirus SARS-CoV-2 causes the COVID-19 disease, which especially in elderly, weakened and immunocompromised patients, shows severe and fatal courses.[1-3] In the meantime, SARS-CoV-2 has spread to a worldwide pandemic with yet incalculable medical, economic and socio-political consequences. So far, there are no established therapies and a vaccine is not yet available.

Deaths and serious illness are more common in the older population over 60 years of age.[4] Outbreaks in long-term care facilities have been observed in several countries, which pose particular challenges in terms of containment and isolation within the facility, affecting and threatening those most at risk. For patients over 65 years of age with SARS-CoV-2 infection, a high hospitalization rate of between 28.6% and 43.5% in the age group 65-74 years and between 30.5% and 58.7% in the age group 75-84 years has been described, with an associated high mortality rate of up to 30%.[4]

There are two promising options for reducing the number of severe COVID-19 disease cases in elderly and comorbid people in the future:

- The development of preemptive measures (vaccination) that prevent the disease or reduce its progression.
- A therapeutic intervention in early stages of the disease, especially in the group of ≥ 65-year-olds with the highest risk of a severe course of the disease.

Both approaches can prevent deterioration in disease course, reduce the frequency of hospital admissions and intensive care treatment and thus take the pressure off the health care system.

T-cell based immunity

T-cell immunity plays an essential role in the control of viral infections. $CD4^+$ T-helper cells (Th1) are essential for the regulation and maintenance of the immune response and for the production of antiviral cytokines, while cytotoxic $CD8^+$ T-cells (CTL) are responsible for the elimination of virus-infected cells. The recognition of viral antigens, which are presented as short peptides via the human leukocyte antigen system (HLA), is essential for the activation and function of T cells. To identify and analyze protective T-cell immune responses against viral infections in the human population, a comprehensive identification and characterization of such viral T-cell epitopes is necessary.[5] [6] This knowledge is not only essential for understanding the host's immune response and the mechanisms of long-term protection in case of virus recurrence, but also a prerequisite for the development of new and more efficient therapeutic and preventive immunotherapy approaches. Besides the generation of virus-specific T-cells *ex vivo* with subsequent transfer into the patient,[7-11] the possibility of

direct vaccination with T-cell epitopes for the induction of a T-cell response directly *in vivo* is of particular importance. Such vaccines can be used to generate immune responses against the SARS-CoV-2 without enduring COVID-19 disease. Furthermore, they can also be used therapeutically to prevent severe courses of disease in acute SARS-CoV-2 infected patients by accelerating/generating a virus-specific T-cell response and activating *in vivo* virus-specific B-cells supporting antibody production.

The findings and experience with two other zoonotic coronaviruses - SARS-CoV-1 and MERS-CoV - based on the detection of CoV-specific CD8$^+$ and long-lasting CD4$^+$ memory T-cell responses in convalescents provide evidence that T-cell immunity also plays an important role in the control of coronavirus infections.[12-15] This is even more important since studies on humoral immunity to SARS-CoV-1 provided evidence that antibody responses are short-lived and can even cause or aggravate virus-associated lung pathology.[16 17] For CD8$^+$ and Th1 CD4$^+$ T cells in contrast a crucial role in viral clearance and protection against the deadly SARS-CoV-1 infection was reported especially in terms of reported lung pathology.[12 14 15] Numerous CD4$^+$ and CD8$^+$ T-cell epitopes have been described for SARS-CoV-1 and MERS-CoV, which, due to the sequence homology of the two coronaviruses, suggest potential cross-reactivity and could also be potential T-cell epitopes for the new SARS-CoV-2 virus.[18] With regard to SARS-CoV-2, two very recent studies[19 20] described CD4$^+$ and CD8$^+$ T-cell responses against viral peptide pools in donors that had recovered from COVID-19 as well as individuals not exposed to SARS-CoV-2, indicative of potential T-cell cross-reactivity.[21-23] In own preliminary work , we define SARS-CoV-2-specific and cross-reactive CD4$^+$ and CD8$^+$ T-cell epitopes in a large collection of SARS-CoV-2 convalescents as well as non-exposed individuals and confirmed their relevance for immunity and the course of COVID-19 disease.[24] These SARS-CoV-2 T-cell epitopes show high recognition frequencies in convalescents from SARS-CoV-2 infection, suggesting their important role in the natural course and immune control of COVID-19. These T-cell epitopes represent the basis for the vaccine peptides included in the CoVac-1 vaccine.

SARS-CoV-2 peptide vaccine

The aim of this study is to investigate the safety and immunogenicity of a peptide vaccine consisting of SARS-CoV-2 specific HLA class II peptides in volunteers without prior or current SARS-CoV-2 infection.

The identification and characterization of T-cell epitopes is a long-standing and unparalleled expertise of the Department of Immunology.[25-27] This unique approach is based on i) the prediction of HLA binding sequences for HLA class I and class II alleles using the world's first prediction tool (www.syfpeithi.de[28]) and newer, more refined methods, all based on SYFPEITHI, ii) the identification of naturally presented HLA class I and class II ligands

(immunopeptidomics), iii) the synthesis of synthetic peptides, and iv) the characterization of T-cell epitopes and peptide-specific $CD4^+$ and $CD8^+$ T cell responses. This strategy has been successfully applied in recent years to define and characterize T-cell epitopes derived from various viruses such as CMV, EBV, ADV and influenza as well as tumor-associated antigens of various solid and hematological malignancies [29-33].

Based on this work, the results were translated into therapeutic vaccination and T-cell transfer studies in cancer patients (e.g. NCT02802943) and viral infections[34 35]. This direct translation is made possible by the Wirkstoffpeptidlabor ([redacted]) of the Department of Immunology and the GMP facility for individualized drugs at the University Hospital Tuebingen as well as our immune monitoring platform equipped with state-of-the-art, validated T-cell assays and methods.

The existing experience and logistics can be directly used for the treatment and prevention of COVID-19 disease. In preliminary work for this study, $CD4^+$ T cell epitopes have already been characterized in a large cohort of SARS-CoV-2 infected donors validating their high relevance in the natural course of COVID-19. The vaccination cocktail in the study will consist of seven promiscuous HLA class II peptides from the different proteins of the SARS-CoV-2 virus, predicted to bind to several HLA class II allotypes. Furthermore, especially those peptides were selected that contain embedded HLA class I sequences in order to induce $CD4^+$ T cell responses and $CD8^+$ T cell responses simultaneously. Furthermore, especially for peptides derived from virus surface proteins, only sequences were selected that do not represent antibody epitopes (not accessible to antibodies due to the predicted 3D structure of the protein; for more detail see IB section 4.2.6). This should prevent the formation of antibodies against the vaccinated peptides, which could possibly have a deteriorative effect on COVID-19. Immunogenicity was proven for all HLA class II peptides included in the peptide cocktail in a large cohort of SARS-CoV-2 convalescent donors as well as for single peptides in a first vaccination of a healthy volunteer (for more detail see IB section 4.2.3).

<u>Adjuvants</u>

A further prerequisite for successful peptide vaccination, besides selection of optimal antigen targets, is the use of a suitable adjuvant, which is able to induce potent and long-lasting immune responses. Among the most effective approaches tested in humans is the subcutaneous injection of peptides emulsified in Montanide ISA 51 VG, a water-in-oil-emulsion, combined with the TLR9 ligand CpG.[36] However, CpG is not available for clinical trials, and a peptide/antigen vaccine emulsified in Montanide without any additional adjuvant induces no or only weak immune responses[37]. In the P-pVac-SARS-CoV-2 trial,

the novel TLR1/2 ligand XS15 emulsified in Montanide ISA 51 VG will be employed as adjuvant, applied subcutaneously together with the peptide vaccine. XS15 is a water-soluble derivative of the TLR1/2 ligand Pam_3Cys and induced a strong $CD8^+$ and $Th1CD4^+$ T-cell response against free short peptides in Montanide ISA 51 VG after a single s.c. injection in a healthy volunteer as well as in cancer patients.[38] Immune responses could be induced against viral peptides (including SARS-CoV-2 derived peptides), neoepitopes derived from cancer-specific mutations as well as tumor-associated self-peptides. XS15 results in granuloma formation on the vaccination site, where the vaccinated peptides persist for at least 7 weeks. Peptide-specific T cells were detected at the granuloma site, however, with a lower frequency than in peripheral blood, which rules out the risk of T-cell sequestration, dysfunction or deletion at the vaccination site due to the use of XS15 in Montanide ISA 51 VG. Strikingly, the induced immune responses were found to persist for more than 1.5 years.

With regard to the planned study we could also show that this vaccination method is able to induce potent SARS-CoV-2 specific T-cell responses in a human volunteer (for more detail see IB of XS15 (1.0. 27 May 2020)).

1.1. Trial Rationale and Justification

1.1.1. Mechanism of action and rationale for a prophylactic SARS-CoV-2 multi-peptide vaccine

The CoVac-1 vaccine evaluated in the P-pVAC-SARS-CoV-2 study is based on multiple HLA-DR SARS-CoV-2 T-cell epitopes and aims to induce SARS-CoV-2 specific T-cells in the vaccinated donors. Antibodies other than IgM are only produced if T cell help is provided to the B cells. Therefore the rationale of the T-cell inducing CoVac-1 vaccine described here is to induce T-helper cells first, before infection and thus before B cells have first contact to the viral antigen. If the B cells then see antigen after infection, they will present the antigens(s) recognized on their HLA class II molecules, and immediately will receive help from the preactivated and expanded vaccine induced T cells. During natural infection, it would take several days for the T cells to get activated and sufficiently expanded. Thus, the production of antibodies, in particular of IgG and IgA classes, should occur much faster in the vaccinated individuals, so that the virus can be cleared faster. Of special note is here that older individuals have lower numbers of T cells, in particular $CD4^+$ T cells [39 40]. Thus, virus antigen specific $CD4^+$ T cells already preactivated and expanded at the time of infection should be especially benefitting for older individuals. Multiple studies in animal models have

clearly demonstrated the requirement of $CD4^+$ T cell help for the generation of protective antibody responses (for example, influenza[41], malaria[42 43], vaccinia[44 45]). Recent studies have also demonstrated that the role of $CD4^+$ T cells in the immune response to viral infections is not limited to help for antibody production; $CD4^+$ T cells are also required to generate optimal $CD8^+$ T cell responses[46-49]. Moreover, $CD4^+$ T cells additionally can act as effector cells by the secretion of cytokines and direct killing of infected cells[50-54]. HLA class II antigens specifically activate $CD4^+$ helper T cells, therefore the CoVac-1 vaccine based on SARS-CoV-2-derived HLA class II peptides will enable a potent cellular and humoral immune response to SARS-CoV-2 preventing severe courses of COVID-19.

The development of a multi-peptide vaccine focusing on the induction of SARS-CoV-2 specific T-cell responses is further supported by several recent publications describing a decrease in neutralizing SARS-CoV-2 antibodies in COVID-19 convalescents after two to four month[55 56]. In contrast a recent study still detected SARS-CoV-1 specific T-cell 17 years after infection suggesting that in contrast to antibodies T cells might enable a long lasting immunity to SARS-CoV-2. In own preclinical data we could further detect SARS-CoV-2 specific T-cell against the T-cell epitopes in the CoVac-1 vaccine in donors after COVID-19 infection even if no antibody responses could be detected. Furthermore, we could show that donors with a high diversity of T-cell responses to SARS-CoV-2 T-cell epitopes in terms of numbers of epitopes detected by a donors was associated with milder symptoms of COVID-19[24].

1.1.2. Rationale for the usage of XS15 as adjuvant in the prophylactic SARS-CoV-2 multi-peptide vaccine

Beside the selection of optimal antigen targets, a further important prerequisite is the use of suitable adjuvant drugs able to induce potent and long-lasting immune responses. In this clinical study, we will use for the first time the novel TLR1/2 ligand XS15 (emulsified in Montanide ISA 51 VG) which 1) is water-soluble and 2) GMP-amenable, 3) non-toxic and 4) effective in inducing T cell responses *in vivo.* The active molecular component in XS15 is Pam3Cys. This is a natural substance component found in bacteria and as such has already been used in a borreliosis vaccine (Limerix) approved in the USA in over 20,000 healthy people[57 58]. Pam3Cys was covalent with a protein compound (Surface protein A (OspA) from B. burgdorferi). In experimental peptide vaccines, Pam3Cys-peptide conjugates proved to be very efficient, but such molecules are unsuitable for pharmaceutical development, especially for personalized multi-peptide vaccines, as validation of a drug produced from them would be very costly or impossible. For this reason, the water-soluble Pam3Cys derivative XS15 was

developed. This derivative has a comparable effect to the above mentioned conjugates in vitro, but is more suitable for pharmaceutical development, because it is water soluble, easily purified by HPLC and detectable by mass spectrometry. Combined with Montanide ISA 51 VG and peptides, XS15 induces efficient T-cell responses after a single injection. This is especially important for its use in prophylactic viral vaccines, as immunization of large cohorts requires highly efficient immunity induction with the lowest number of vaccinations possible. Thus, Montanide/XS15 can be considered as a GMP-amenable version of the well known Complete Freund's Adjuvans [59 60] and therefore represents the optimal adjuvant for the P-pVAC-SARS-CoV-2 study.

Based on animal toxicity data and preliminary evidence (self-administration of vaccines and information gained through administration of XS15 adjuvanted vaccines as an unproven intervention, according to physicians judgement and with informed consent, in keeping with principle 37 of the Declaration of Helsinki), we assume that a dosage of 50 µg XS15 (total dosage) administered as a vaccine together with Montanide ISA 51 VG and synthetic peptides can be considered as a safe and potentially effective strategy (for more detail see IB of XS15 (1.0. 27 May 2020)).[38 61]

1.1.3. Rationale for selected doses

1.1.3.1. Dose rationale for peptides

Previous vaccination trials were performed at peptide doses ranging from 10 to 5,000 µg per vaccination: Even though only a few of these trials included a dose finding element, there is a tendency that doses below 100 µg are not effective to induce T-cell responses whilst doses above 500 µg do not seem to generate an increasing immunogenicity. Dose-finding studies performed with viral protein-derived epitopes showed significantly stronger immune responses in the 300-500 µg range versus the 100 µg dose, without significantly higher immune responses in the 1,000 vs. 500 µg group[62]. This is supported by own data of the investigator and the Immatics Biotechnologies GmbH[63] (for more details refer to the IB of CoVac-1). Preliminary data from a healthy volunteer and cancer patients vaccinated with a personalized peptide vaccine (240-300 µg per peptide) including two of the CoVac-1 peptides (250µg) in combination with XS15 showed potent induction of T-cell responses in 100% of HV and patients and a good safety profile. Concerning safety of peptide vaccines in different doses no severe side effects were observed even with very high doses of peptides up to 30mg[64,65].

Furthermore, a similar multi-peptide vaccination study for influenza evaluated safety and immunogenicity with two doses of peptides (250µg and 500µg). No difference in the safety profile was detected for the two different doses and significant induction of functional T-cell responses were observed for both peptide doses, suggesting the dose of 250µg sufficient and safe for a prophylactic viral peptide vaccine[66].

The dose of ~250 µg per peptide per dose for CoVac-1 vaccine was selected based on these findings and on the feasibility in pharmaceutical development of the vaccines.

1.1.3.2. Dose rationale for XS15

The molecular mode of action of both the Pam3Cys conjugates and XS15 is an activation of immune cells via the toll-like receptor TLR1/2. These immune cells are mainly found in the blood and lymphoid tissues. Desired as well as toxic effects are therefore to be expected above all and presumably exclusively due to the XS15-TLR1/2 interaction with these cells, in particular through an over activation of these cells, which could then lead to a so-called cytokine release syndrome. The dose of XS15 is based on an in vitro assay that investigated both potential toxicity as well as efficiency. In these assay 10 µg/ml XS15 was shown to be the most efficient dose for the stimulation of immune cells (for more details please refer to the IB of XS15). The following considerations regarding the concentration of XS15 after a subcutaneous administration are the basis of dose finding: When used with Montanide ISA 51 VG in a total volume of 500 µl suspension, a granuloma forms rapidly at the injection site, which has a volume of estimated 2 ml. This granuloma further increases up to 8ml on day 17 after vaccination[38]. Thus, the initial local concentration of XS15 is maximally 50 µg/ml which is reduced soon thereafter to 25 µg/ml (50µg in 2 ml) and soon thereafter is diluted even more, since the granuloma increases more, so that a concentration of 10 microgram/ml will soon be reached. Further dilution will follow with the granuloma increase to 6,25mg/ml (50 µg in 8ml). Based on this in vitro experiments and considerations the dose of 50 µg was selected for further in vitro and in vivo toxicity evaluation as well as for first in vivo vaccination experiments.

In the toxicity study of mice, a dose of 50 µg XS15 in Montanide, applied locally s.c., did not reveal any toxicity beyond the long known and expected toxicity of Montanide alone. Therefore, this study proves that XS15 has no local and above all no systemic toxicity under this application method up to the above mentioned dose (for more details please refer to the IB of XS15). Furthermore, considering systemic toxicity of XS15 50µg after s.c. injection the following considerations were made: If this dose (in the absence of Montanide ISA 51 VG) is immediately distributed in the blood (6l), a maximum blood concentration of 0.008 µg/ml

would be expected. At a concentration of 0.008 µg/ml no measurable reaction (stimulation of immune cells) is detected in the above described in vitro test.

When used with Montanide, the formation of a granuloma at the injection site, which has a depot effect for peptides, means that a gradual release of these peptides or XS15 into the blood can be expected. Therefore, the actual blood concentration of XS15 after administration of 50 µg in a Montanide/water emulsion is likely to be much lower than the maximum concentration of 0.008 µg/ml described above. Therefore, a systemic toxic effect of XS15 is not expected at a dose of 50 µg s.c. with or without Montanide.

1.1.3.3. Dose rationale for Montanide ISA 51 VG

Montanide™ ISA 51 VG has been used in about 300 clinical trials from phase I to phase III which represents more than 19 000 vaccines. In addition, Montanide™ ISA 51 VG has been approved in a commercial vaccine against non-small cell lung cancer (NSCLC).

Dosing of 0,25ml after 50/50 mixture with peptides is based on two published clinical studies evaluating influenza vaccines in more than 2500 donors showing high immunogenicity and a good safety profile[67] [68]. Detailed information on preclinical and clinical safety data for Montanide ISA 51 VG could be found in the respective IB as well as in the attached “Human application form for Montanide ISA 51 VG”.

1.1.3.4. Rationale for one dose schedule

The combination of multi-peptide vaccine with the TLR1/2 ligand XS15 emulsified in Montanide ISA 51 VG with the above described dosing was already evaluated in a healthy volunteer as well as in cancer patients (n=12). Multi-peptide vaccines included beside tumor-associated neoepitopes and self-peptides also viral T-cell epitopes derived from CMV and SARS-CoV-2. In all vaccinated individuals peptide-specific T-cell responses could be detected after one single vaccination. For viral T-cell epitopes including SARS-CoV-2 derived peptides strong T-cell responses could even be detected ex vivo without in vitro amplification of T-cells after one single vaccination. Immune responses after vaccination were shown to last for more than 1,5 years so far. Furthermore, the safety profile of these vaccines with similar composition and dosing as for the CoVac-1 vaccine was very good after a single vaccination, showing only grade 1 local reaction at vaccination side after single injection. Therefore, the first-in-man evaluation of CoVac-1 with a single vaccination seems reasonable to enable efficient induction of immune response with the lowest possible number of vaccination and side effects. Please find below a detailed description of the data from in vivo

administration of peptide vaccines in similar composition in a healthy volunteer and cancer patients (for more details please refer to the IB of CoVac-1).

1.1.4. Rationale for trial design

This is a phase I multi-peptide vaccination study using SARS-CoV-2 HLA-DR peptides in combination with the novel TLR1/2 ligand XS15 in healthy volunteers to prove safety and immunogenicity. The primary objective is incidence and severity of AEs (≥ Grade 4) after vaccination in the observational time (until day 28). Furthermore, the trial aims to expand experience on overall safety and immunogenicity in the study cohort.
This is based on the following rationale:

The SARS-CoV 2 pandemic is currently one of the major threats to the world population and requires the rapid development of effective preventive and therapeutic tools. $CD4^+$ and $CD8^+$ T-cells, as comparts of the adaptive immune system, are an important cornerstone in the control of viral infections. As state above, T-cell immunity seems to play a significant role in corona virus infections including SARS-CoV-2 and has a major impact on the course of disease including severe lung pathology as observed in COVID-19. The induction of SARS-CoV-2 specific T-cell responses therefore might represent a valuable preventive and therapeutic tool especially in the group of elderly and comorbid patients to prevent severe courses of SARS-CoV-2 infection. SARS-CoV-2 specific T-cell immunity can be achieved by peptide vaccination applying SARS-CoV-2 specific promiscuous HLA class II T-cell epitopes. The HLA class II epitopes were selected based on the immunogenicity in a cohort of SARS-CoV-2 convalescent donors, proving their pathophysiological relevance in COVID-19.[24]

In view of the pandemic spread of COVID-19, health care systems face major challenges, as a large number of patients require hospital treatment and intensive care. As soon as the capacities of individual health care systems are exceeded, optimized care for all can no longer be guaranteed.
Containment strategies in Germany include the quarantine of infected persons and the 14-day quarantine of contact persons (incubation period). At the population level, most affected countries have reduced contacts through various measures such as closing schools, shops, restaurants and, in extreme cases, a total curfew. Without effective treatment options for COVID-19 and a vaccine available for the broad population, these measures can not be terminated, which results in immense economic and socio-political damage. This

underscores the high need for the development of novel treatment approaches to prevent a severe disease course of SARS-CoV-2 infection.

Therefore this trial has been conceptualized to prove safety and immunogenicity of a peptide vaccine against SARS-CoV-2. The focus in the study population is set to older participants. This is of special interest as these people are considered to be at high risk for severe disease and society has to protect the elderly. Vaccination will be conducted in three different healthy volunteer cohorts (Part I-III), each followed by an interim safety analysis before proceeding:

- Part I: Healthy adult aged 18-55 years
- Part II: Adults aged > 55. After proving safety and immunogenicity in a cohort of healthy volunteers aged 18-55 (Part I), an interim safety analysis will be conducted and prior to continuation with Part II approval by DSMB and of an amendment by PEI and Ethics Committee must be obtained.

1.1.5. Preliminary experiences from study part I

P-pVAC-SARS-CoV-2 is a phase I single-center safety and immunogenicity trial of multi-peptide vaccination with CoVAC-1 to prevent COVID-19 infection in adults. The study is recruiting since November 2020 and has completed the first part (healthy volunteers (n=12), age 18-55 years) in February 2021. One single subcutaneous vaccination of CoVAC-1 was applied. Immunogenicity, in term of induction of T-cell responses to one or more of the six HLA-DR SARS-CoV-2 T cell epitopes included in the CoVAC-1 vaccine was assessed pre vaccination as well as on day 7, 15 and 28 after vaccination (please refer to the IB of CoVAC-1 for more details). Induction of SARS-CoV-2 T cells was shown in 100 % (12/12) of volunteers in part I of the study. Earliest T cell responses were observed at day 14 (V3) for 11/12 volunteers. Immune responses were induced to multiple of the vaccine peptides (median 5/volunteer, range 4-6).

First safety data of CoVAC-1 are available until d28 (V4) after vaccination. As intended and expected all volunteers (n=12) developed a granuloma local at injection site. Further local injection site adverse events included transient erythema, itching, pain and skin ulceration. Until day 28 no relevant systemic side effects, especially no fever or other inflammatory reactions were reported. No allergic reactions were observed. For a detailed description of all ADRs reported please refer to the IB of CoVAC-1.

Thus, these preliminary data suggest a high immunogenicity of CoVAC-1 to induce early and multi-peptide T cell responses as well as a good tolerability and safety profile.

1.2. Benefit / Risk Assessment

The assumed clinical benefit and risk of P-pVAC-SARS-CoV-2 vaccination are based on the following aspects:

- Peptide vaccination using HLA-presented peptides represents an established immunotherapy approach utilized for preventive vaccine development in infectious disease[69] [70] as well as for therapeutic approaches in malignant disease. Several peptide vaccination studies in patients with malignant disease including solid tumors[63] [71-73] and hematological malignancies[74-77] have proven safety and tolerability of this approach.
- Multi-peptide vaccination represents a low side-effect immunotherapy approach relying on specific immune recognition of HLA-presented peptides[78-80].
- The Wirkstoffpeptidlabor holds certificates for the production of GMP grade synthetic peptides and for the formulation of multi-peptide vaccine cocktails including the TLR1/2 ligand XS15, which allows for a rapid GMP production of the CoVac-1 vaccine. This is of great importance due to the serious threat the SARS-CoV-2 pandemic currently poses to the world population.
- All peptides included in the CoVac-1 vaccine are proven SARS-CoV-2 T-cell epitopes with pathophysiological relevance in the natural course of COVID-19 disease
- CoVac-1 peptide vaccination can induce potent CD8$^+$ and CD4$^+$Th1 T-cell responses against SARS-CoV-2 providing immunity against infection as:
  - CD4$^+$Th1 cells will directly contribute to virus clearance and deliver strong T helper signals to CD8$^+$ T cells primed during natural infection. Furthermore, these SARS-CoV-2 specific CD4$^+$Th1 cells can activate virus antigen-experienced B cells. The resulting enhanced activity could lead to more rapid virus clearance and prevention of a severe course of COVID-19 disease.
  - Vaccine peptides contain embedded CD8 T-cell epitopes predicted to bind to many HLA class I allotypes. Such CD8$^+$ T cells should also contribute to faster virus clearance.
- Since we found IFNγ-producing SARS-Cov-2 specific T-cells in a healthy volunteer vaccinated with SARS-CoV-2 T-cell epitopes, it is very likely that significantly CD4$^+$Th1 T cells are induced by the vaccine. There should be thus no disease enhancing-effect due induction of Th2-bias as described for other corona viruses[81].
- As development of antibody-dependent enhancement (ADE) has been identified as potential risk[82] for infected patients after vaccination approaches, the following considerations and risk mitigation strategies have been undertaken:
- In contrast to other classical vaccines aiming to induce an antibody response to

prevent viral infections, the CoVac-1 vaccine is designed to induce SARS-CoV-2 specific T-cells. According to experience from comparable peptide vaccines in cancer patients it is very unlikely, that such antibodies will be induced after a single vaccination. Induction of antibodies against vaccine peptides were observed in cancer patients with delay, and only after several vaccinations. So far, no antibody induction against the T-cell epitopes included in the CoVac-1 vaccine was observed.

- Furthermore and most importantly, even in the unlikely event of antibody induction against CoVac-1 vaccine peptides, which will be monitored during the study as outlined in the protocol (section 6.3.2), these antibodies cannot recognize viral particles, because none of the vaccine peptides is exposed on the virus particle surface. Thus, neither neutralizing nor ADE-inducing antibodies can be induced by the vaccine. In contrast to ADE mediated by vaccine induced antibodies, which as describe above is extremely unlikely with the CoVac-1 vaccine, there might be a risk of ADE in cases of SARS-CoV-2 infection in which the patient’s B cells have already been primed against epitopes of common cold seasonal human coronavirus strains and produce low amounts of antibodies, antibodies with low affinity or antibodies with the wrong affinity. In theory, vaccine-induced $CD4^+$ T-cells might cause or exacerbate immune pathological effects indirectly. As such *in vivo* effects can not be preliminary assessed in an in vitro setting, symptoms attributable to SARS-CoV-2 infection will results in subsequent PCR testing and proven SARS-CoV-2 infection will be reported as AEs of special interest (AESI). These AESIs will be monitored particularly carefully including early hospital admission of patients with COVID-19 after CoVac-1 vaccination. This was outlined in more detail in the study protocol.
- Preliminary safety and immunogenicity analyses of the volunteers vaccinated with CoVAC-1 in part I of the study (n = 12) have proven high immunogenicity with the induction of early, multi-peptide-directed functional T cell responses in 100% of volunteers as well as a good safety profile with no systemic adverse drug reactions or allergic reactions.

- Participant selection is based on medical care and safety considerations:
  - The trial comprises two parts (cohorts of participants) with different age ranges to provide preliminary results on safety in a cohort of young (18-55 years, n=12) and healthy participants, which is then extended to older (Part II) participants. Of note, the risk of vaccine related (S)AEs is hypothesized to be similar in each age group.
  - The design addresses the urgent medical need for protection of people at risk

for serve SARS-CoV-2 infection by providing safety and immunogenicity data as well as first efficacy data in terms of SARS-CoV-2 infection in this population.

  - After Part I of the clinical trial (last patient has completed V4) a substantial amendment is send to the regulatory authorities besides seeking advice from the DSMB.
  - Safety is continuously monitored by an independent DSMB, which will be provided with reports on a regular basis (see DSMB Charter).

- Successful development of a peptide vaccine will help to put an end to quarantine and fear of SARS-CoV-2.
- Confirming safety of the CoVac-1 vaccine in volunteers within the P-pVAC-SARS-CoV-2 study will further allow the transfer of this approach to induce SARS-CoV-2 specific T-cell immunity in a therapeutic setting for patients with SARS-CoV-2 infection.

The assumed clinical benefit and risks of peptide vaccination in combination with the TLR1/2 ligand XS15 in Montanide ISA 51 VG are based on the following aspects:

- Peptide vaccination alone is rarely able to induce clinically effective T-cell responses; thus the peptide vaccine has to be combined with an adjuvant drug to enhance immune responses.
- Several TLR ligands have been shown to potently induce $CD8^+$/Th1$CD4^+$ responses in humans, including CPG (TLR9 ligand), imiquimod (TLR7 ligand) and poly-IC (TLR3 ligand). However, no GMP compliant substance based on these TLR ligands is available that can be applied with a peptide vaccine.
- XS15 is a water-soluble derivative of the TLR1/2 ligand Pam3Cys and induces a strong $CD8^+$ and Th1$CD4^+$ T-cell response against free short peptides emulsified in Montanide ISA 51 VG after a single s.c. injection in healthy volunteers as well as cancer patients.
- Using XS15, immune responses could be induced for viral peptides (including SARS-CoV-2 derived peptides), neoepitopes from cancer-specific mutations as well as for tumor-associated self-peptides.
- XS15 results in granuloma formation on the vaccination site, where the vaccinated peptides persist for at least 7 weeks, which supports the induction of a strong immune response.
- The induced immune responses observed so far persisted for more than 1.5 years.

- Beside formation of granuloma locally on injection side, no relevant side effects of peptide vaccination in combination with XS15 in Montanide ISA 51 VG were observed in a healthy volunteer and cancer patients. In particular, no allergic or anaphylactic reactions or cytokine release syndrome have been observed (detailed information can be found in the IB V1.0 and the IB of XS15 (1.0. 27 May 2020)).
- Montanide ISA 51 VG is an oil adjuvant suitable for human injection that allows the manufacturing of water in oil emulsions. Montanide ISA 51 VG has been used in more than 200 clinical trials including more than 6000 patients. Most common side effects are injection site reactions (68%) including granuloma development, fatigue (54%), fever (41%), gastrointestinal disorders (32%) and injection site or local erythema (28%)[83]. In general, the observed adverse from controlled trials with non-healthy as well as healthy individuals were mild to moderate in intensity.

Conclusion

Taking into account the lack of effective treatment options and the dismal prognosis in SARS-CoV-2 infected high risk patient populations, especially in comorbid patients aged > 65 years, the expected benefits of a SARS-CoV-2 specific HLA class II peptide vaccination in combination with XS15 emulsified Montanide ISA 51 VG are considered to outweigh the potential risks for the participants, especially since multiple risk mitigation (e.g. interim safety analysis) measures have been incorporated.

1.3. Data and Safety Monitoring Board (DSMB):

An independent Data and Safety Monitoring Board (DSMB) will be assembled. The DSMB will be composed of independent experts in the field of immunology and infectiology assessing the progress, safety data and critical efficacy endpoints. The mission of the DSMB is to ensure the ethical conduct of the trial and to protect the safety interests of participants in this trial.

The DSMB will receive a report listing and summarizing all the relevant safety data at least twice. The first assessment (first interim safety report, section 9.5) will take place after Part I of the trial including DSMB approval and an amendment at the regulatory authorities (Paul-Ehrlich Institute, PEI) and Ethics Committee (EC). If the IMP is considered safe for continuation by DSMB, Part II of the trial will start recruiting. In addition, the report will provide data concerning recruiting rates, status of the trial and AESIs (section 9.1.4); also non-occurrence will be mentioned. An emergency meeting of the DSMB may be called at any time should questions of volunteer safety arise or holding rules apply, and necessary safety

reports will be provided. Meetings may be convened as conference calls/e-mail as well as in person.

2. Study Objectives

2.1. Primary Objective and Endpoint

The primary objective of this trial is to evaluate the safety and tolerability of the CoVac-1 vaccine, a single dose SARS-CoV-2 specific multi-peptide vaccine combined with the TLR1/2 ligand XS15 emulsified in Montanide ISA 51 VG in adults.

2.1.1. Primary Endpoint

The nature, frequency, and severity of AEs and/or SAEs associated with administration of CoVac-1:

- Solicited: ADRs/AEs occurring from the time of each injection throughout 28 days following the procedure, facilitated by use of a volunteer diary
- Unsolicited: AEs from the time of injection throughout 56 days following injection
- SAEs from the time of injection until the final study visit for each subject
- Incidence of AESIs until the final study visit for each subject

2.2. Secondary Objectives and Endpoints

Secondary objectives of this trial are to evaluate the efficacy of the CoVac-1 vaccine in terms of induction of SARS-CoV-2 specific T-cells.

2.2.1. Secondary Endpoints

- Development of a CoVac-1 specific T-cell response to at least one of the single SARS-CoV-2 T-cell epitopes included in the CoVac-1 vaccine on Visits 2, 3, 4, 5 measured by IFN-γ ELISpot ex vivo and after in vitro T-cell amplification (compared to Visit 1), this includes:
    - Cellular conversion rate (CCR) at Visits 2, 3, 4, 5 after immunization

2.3. Exploratory Objectives and Endpoints

Explorative objectives are the duration and characteristics of T-cell responses and the analysis of induction of antibody responses to single SARS-CoV-2 T-cell epitopes included in the CoVac-1 vaccine.

2.3.1. Exploratory Endpoints

- Characteristics of T-cell response on Visits 2, 3, 4, 5 measured by ELISpot/ICS. This includes:
  - Phenotyping of SARS-CoV-2 specific T-cells (CD4, CD8 etc.) by flow cytometry
  - Characterization of cytokine profiles of SARS-CoV-2 specific T cells (TNF, IFN, IL-2, CD107a etc.) by intracellular cytokine staining
  - Recognition rate defined as percentage of peptides inducing a T cell response in one individual
  - Intensity of T cell response to a single SARS-CoV-2 T cell epitope included in the CoVac-1 vaccine
- Induction of long-term SARS-CoV-2 specific T-cell responses 3 and 6 months after peptide vaccination.
- Induction of antibodies specific to the SARS-CoV-2 T-cell epitopes included in the CoVac-1 vaccine measured by ELISA. In case of unexpected detection of CoVac-1 specific antibodies the following assays will be performed:
  - Individual neutralization antibody titers
  - Seroconversion rates
  - Calculation of geometric mean titers (GMT) for neutralizing and binding antibodies
- Biomarkers and clinical characteristics influencing immunogenicity.

3. Study Design

This is an interventional, open-label, phase I trial evaluating the CoVac-1 vaccine, a single dose SARS-CoV-2 specific multi-peptide vaccine combined with the TLR1/2 ligand XS15 emulsified in Montanide ISA 51 VG in adults. The study is divided into two parts, which will recruit consecutively. Prior to initiation of the next part, the previous part must have completed recruiting, and day 28 of the last patient enrolled must have passed. After interim safety analysis and approval from the authorities (section 9.5), the next study part starts recruiting (Figure 1 and 2).

The first volunteer included in the trial will be hospitalized after vaccination and closely monitored. This patient is observed until day 28 and possibly arising safety issues are reported to and decided on by the Sponsor. Thereafter, no more than one subject per day will be treated/vaccinated. 28 days following vaccination of the 12th volunteer, there will be an interim analysis of safety and a safety review by the data safety monitoring board (DSMB) as well as a substantial amendment to the regulatory authorities (PEI and EC) before proceeding to Part II. Part II must not start recruiting prior to approval by authorities. Volunteers of part II are treated simultaneously. Details can be found in figure 3.

To avoid bias in treatment, a manualized process protocol as well as monitoring and treatment reports are implemented. The volunteer selection will be documented. Reasons for refusal will be assessed. To avoid bias in data analysis, monitoring and analysis by intention-to-treat are planned. Data analysis will be conducted by an independent statistician.

Figure 1: Overall Study Design

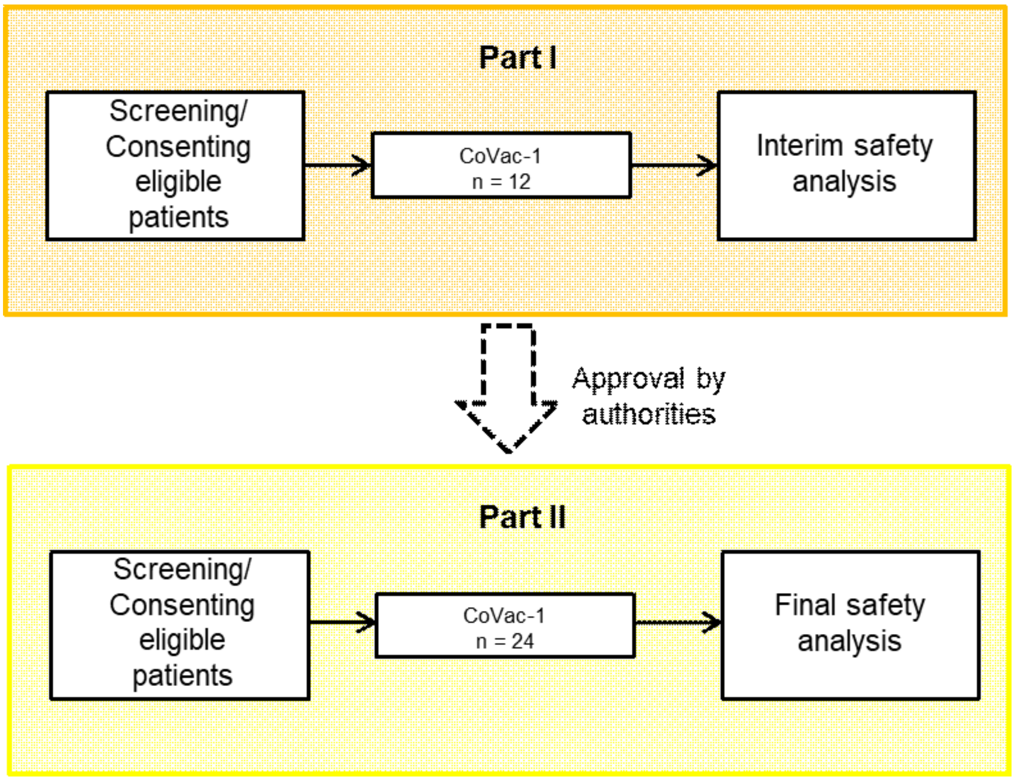

Figure 2: Individual Study Procedure

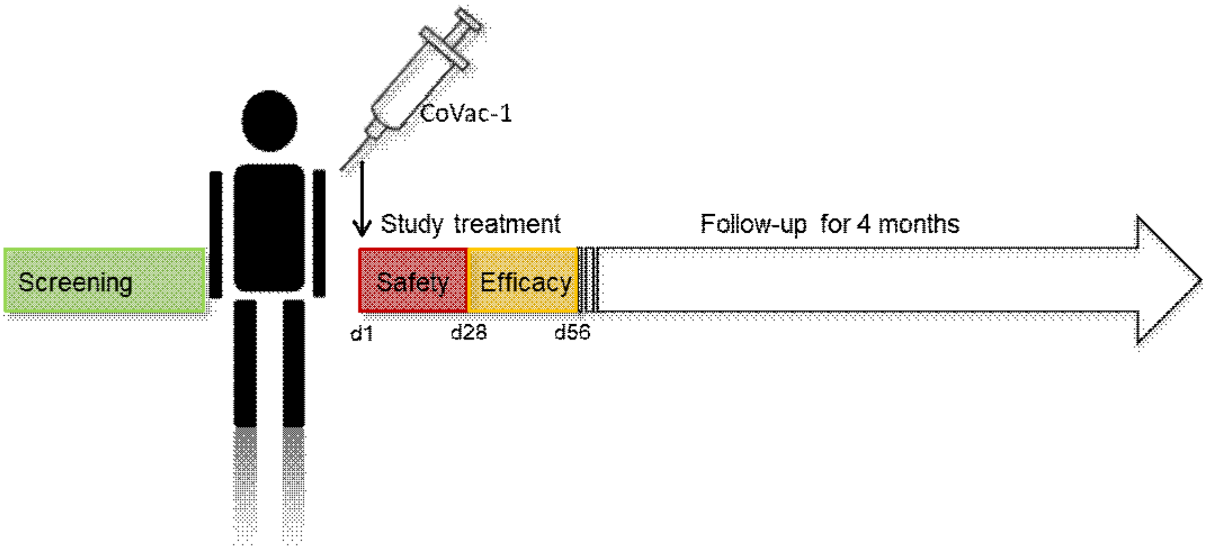

Figure 3: Treatment sequence

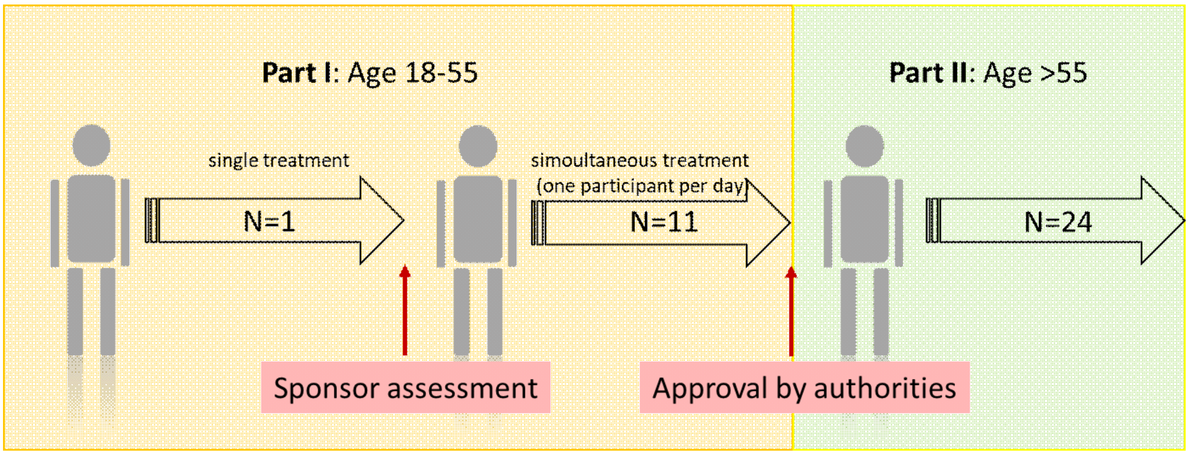

3.1. Study Duration and Schedule

The duration of the trial for each subject is expected to be 6 months, including 2 months of safety follow-up after vaccination and 4 months of follow-up.

The overall duration of the trial is expected to be approximately 12 months including the preparatory phase. Recruitment of subjects will start in Q3 2020. The actual overall duration or duration of recruitment may vary. The study timeline is described in Table 2.

Table 2: Study Timelines

Total trial duration	12 months
Duration for individual volunteer	Study treatment: 2 months<br>Follow-up: 4 months
FSI (First Subject In)	Q4/2020
LSI (Last Subject In)	Q1/2021
LSO (Last Subject Out)	Q3/2021
DBL (Data Base Lock)	Q3/2021
Statistical Analyses Completed	Q4/2021
Trial Report Completed	Q4/2021

3.2. End of Study

The end of the study is defined as the last visit of the last volunteer.

4. Study Population

Healthy subjects (designated as volunteers):

Healthy adult women and men aged 18-55 (Part I), followed by adult women and men aged > 55 with age adjusted health condition (Part II).Volunteers will be recruited by means of paper- and online-based calls as considered appropriate by the EC of the University Hospital of Tuebingen.

4.1. General Criteria for Subject Selection

Adult male and female volunteers fulfilling the inclusion criteria outlined below will be enrolled.
The trial population will consist of both genders. Gender distribution in the trial is supposed to reflect the distribution in the population; there will be no prior defined quantitative ratio between females and males.

4.1.1. Inclusion Criteria

Subjects meeting all of the following criteria will be considered for admission to the trial:

1. Adult male or non-pregnant, non-lactating female
    1. Part I: Age 18-55 at the time of screening
    2. Part II: Age >55 years at the time of screening
2. Pre-existing medical condition
    1. Part I: Free of clinically significant health problems, as determined by pertinent medical history and clinical examination at study screening
    2. Part II: With or without pre-existing medical condition, not requiring change in therapy or hospitalization before enrollment
3. Ability to understand and voluntarily sign the informed consent form.
4. Ability to adhere to the study visit schedule and other protocol requirements.
5. FCBP and male volunteers with partners of childbearing potential, who are sexually active must agree to the use of two effective forms (at least one highly effective method) of contraception. This should be started from the signing of the informed consent and continue until three months after vaccination
6. Postmenopausal or evidence of non-childbearing status. For women of childbearing potential: negative urine or serum pregnancy test within 7 days prior to study treatment. Postmenopausal or evidence of non-childbearing status is defined as:

    1. Amenorrhoea for 1 year or more following cessation of exogenous hormonal treatments
    2. Luteinizing hormone (LH) and Follicle stimulating hormone (FSH) levels in the post-menopausal range for women under 50

7. Be willing to minimize blood and body fluid exposure of others for 7 days after vaccination
    1. Use of effective barrier prophylaxis, such as latex condoms, during sexual intercourse
    2. Avoiding the sharing of needles, razors, or toothbrushes
    3. Avoiding open-mouth kissing
    4. Refrain from blood donation during the course of the study

4.1.2. Exclusion Criteria

Subjects presenting with any of the following criteria will not be included in the trial:

1. Pregnant or lactating females.
2. Participation in any clinical study with intake of any investigational drug interfering with the study primary endpoint
3. Any concomitant disease affecting the effect of the therapeutic vaccine or interfering with the study primary endpoint
4. Any immunosuppressive treatment except low dose corticosteroids (≤10mg prednisolone/day)
5. Prior or current infection with SARS-CoV-2 tested serologically or by throat/nose swab (PCR)
6. History of Guillain-Barré Syndrome
7. Positive serological HIV, hepatitis B or C test. In case of positive HBsAg, volunteer must provide prove of hepatitis B vaccination, otherwise volunteer must be excluded.
8. History of relevant CNS pathology or current relevant CNS pathology (e.g. seizure, paresis, aphasia, cerebrovascular ischemia/hemorrhage, severe brain injuries, dementia, Parkinson's disease, cerebellar disease, organic brain syndrome, psychosis, coordination or movement disorder, excluding febrile seizures as child)
9. Baseline laboratory with lymphocyte count ≤ 1000/µl
10. Only Part I:
    - Acute or chronic, clinically significant psychiatric, hematologic, pulmonary, cardiovascular, or hepatic or renal functional abnormality as determined by the

Investigator based on medical history, physical exam, and/or laboratory screening test

11. All parts of the clinical trial
    - Diabetes mellitus Typ II requiring drug treatment
    - Chronic lung disease requiring drug treatment
    - Any chronic liver disease or unknown liver abnormalities defined as:
        - ALT and AST ≤ 2.5 x ULN
        - γ-GT ≤ 2.5 x ULN
    - Chronic renal failure defined as GFR < 60 ml/min/1,73m^2
    - Serious pre-existing cardiovascular disease such as NYHA ≥ I, coronary heart disease requiring coronary surgery or known pAVK ≥ grade 2
    - Sickle cell anemia
    - Obesity (as defined by age adjusted body mass index)
12. Hospitalization at study inclusion
13. Administration of immunoglobulins and/or any blood products within 120 days preceding study entry or planned administration during the study period
14. History of blood donation within 30 days of enrolment or planned donations within the study period
15. Known hypersensitivity to any of the components included in the CoVac-1 vaccine
16. Pre-existing auto-immune disease except for Hashimoto thyroiditis and mild (not requiring immunosuppressive treatment) psoriasis

5. General Information on the Investigational Medical Product (IMP)

Definition of terms

**Drug substances:**	Six SARS-CoV-2-derived HLA class II peptides derived and the TLR1/2 ligand XS15
**Peptide cocktail:**	Peptide cocktail for each study volunteer including 6 immunogenic SARS-CoV-2 peptides and the TLR1/2 ligand XS15
**IMP/Drug product/ peptide vaccine:**	CoVac-1: Peptide cocktail emulsified in Montanide ISA 51 VG
**IMP administration:**	subcutaneous injection with 2ml syringe (e.g. BD Emerald) and needle (e.g. BD Eclipse Needle 27Gx1/2)

5.1. Peptide Vaccine CoVac-1

The IMP/drug product in this study is CoVac-1. The final peptide vaccine is a water-in-oil emulsion of the peptide cocktail as described in detail below and Montanide ISA 51 VG. All components will be provided by the Wirkstoffpeptidlabor of the Department of Immunology in Tübingen together with a “mixing kit” allowing for the mixture of the components (peptide cocktail, Montanide ISA 51 VG) by the pharmacy of the participating centers.

5.1.1. Peptide cocktail

5.1.1.1. SARS-CoV-2-specific peptides (drug substance)

Each volunteer enrolled in the P-pVAC-SARS-CoV-2 trial will receive 6 promiscuous HLA-DR peptides (250 µg each) derived from different proteins of SARS-CoV-2. Details on drug substance can be found in Table 3.

5.1.1.1. TLR1/2 ligand XS15 (drug substance)

The lipopeptide XS15 (50 µg), chemical name N-Palmitoyl-S-[2,3-bis(palmitoyloxy)-(2R)-propyl]-(R)-cysteinyl-GDPKHPKSF, a water-soluble synthetic Pam_3Cys-derivative is a TLR1/2 ligand that will be included as an adjuvant in the peptide cocktail.

5.1.2. Montanide ISA 51 VG

Prior to application, the peptide cocktail (consisting of 6 SARS-CoV-2-specific HLA-DR peptides and the TLR1/2 ligand XS15) will be emulsified in a water-oil emulsion 1:1 with Montanide ISA 51 VG. Montanide ISA 51 VG is based on a blend of mannide monooleate surfactant and mineral oil and has been used as an adjuvant in more than 200 human vaccine trials. Montanide ISA 51 VG is rendering stable water-in-oil emulsions when mixed with water-based antigenic media.

Table 3: SARS-CoV-2 specific HLA-DR vaccine peptides

sequence	HLA restriction	peptide length	position	protein	protein name	protein class
ASWFTALTQHGKEDL	DR	15	50-64	ORF9	nucleocapsid protein	structural
LLLLDRLNQLESKMS	DR	15	221-235	ORF9	nucleocapsid protein	structural
ITRFQTLLALHRSYL	DR	15	235-249	ORF9	spike protein	structural
LSYYKLGASQRVAGD	DR	15	176-190	ORF5	membrane protein	structural
FYVYSRVKNLNSSRV	DR	15	56-70	ORF4	membrane protein	structural
SKWYIRVGARKSAPL	DR	15	43-57	ORF8	n.a.	non-structural

5.2. Manufacturing of the Investigational Medicinal Product

5.2.1. SARS-CoV-2-specific peptides (drug substance)

All SARS-CoV-2 vaccine peptides are manufactured by the Wirkstoffpeptidlabor, University of Tübingen, Auf der Morgenstelle 15, 72076 Tübingen, Germany. The Wirkstoffpeptidlabor holds certificates for the production of GMP grade synthetic peptides and for the formulation of multi-peptide vaccine cocktails including the TLR1/2 ligand XS15. All peptides are synthetic peptides manufactured by well-established solid phase peptide synthesis (SPPS) procedures using Fmoc chemistry.

5.2.2. XS15 (drug substance)

XS15 is delivered as bulkware in GMP-quality from the external manufacturer Bachem AG, Hauptstrasse 144, CH-4416 Bubendorf in active ingredient quality.

Bachem's manufacturing process is described in a separate "Documentation on XS15 Hydrochloride" of 31.05.2018 by the company. The Wirkstoffpeptidlabor performs a second lyophilization as additional manufacturing step. This manufacturing step is divided into four sub-steps: Reconstitution, combining, aliquoting and lyophilization.

5.2.3. Montanide ISA 51 VG

Montanide is manufactured by Seppic and by the rewarding manufacturer Elaiapharm, respectively.

5.2.4. Peptide cocktail CoVac-1 (drug product)

The peptide cocktail is manufactured by the Wirkstoffpeptidlabor by aseptic filling at the GMP-Center of the University Hospital Tuebingen. Each peptide is solubilized in DMSO and sterile filtered, the obtained peptide solutions are pooled. Water is added and the obtained solution is sterile filtered and filled into single dose vials.

5.3. Labeling of the Investigational Medicinal Product

5.3.1. Peptide cocktail

Peptide cocktails (including the TLR1/2 ligand XS15) will be packaged into sterile containers labeled with an identification code definitely assignable to the P-pVAC-SARS-CoV-2 study and a vial number that will be assigned to the individual study volunteer. The trial medication will be labeled according to § 5 of GCP-V. Samples of the labels are filed in the trial master file (TMF).

The peptide vaccine cocktail will be packaged together with Montanide ISA 51 VG and the mixing equipment into the “mixing kit” and shipped from the *Wirkstoffpeptidlabor* of the Department of Immunology, Tübingen to the pharmacy of the participating center. Shipment will be documented according to standard operation procedures (SOP). The “mixing kit” will be shipped using isolated packaging with an automated temperature control system, whose logging data have to be returned to the Wirkstoffpeptidlabor of the Department of Immunology together with the acknowledgement of receipt after delivery of the consignment. The device will be read out to document the correct storage temperatures during shipment. Data will be documented according to SOP. The shipment will be performed by an associate of the Wirkstoffpeptidlabor of the Department of Immunology, Tübingen.

5.3.2. Montanide ISA 51 VG

Montanide ISA 51 VG is packed by Seppic and Elaiapharm. Montanide will be packaged together with the peptide cocktail and the mixing equipment into the “mixing kit” and shipped from the Wirkstoffpeptidlabor of the Department of Immunology, Tübingen to the pharmacy of the participating center, as described above.

5.4. Storage of the Investigational Medicinal Product

Trial medication will be stored at the pharmacy of the participating center and must be kept in a locked area with access restricted to designated trial staff. The “mixing kit” including the peptide cocktail and Montanide ISA 51 VG must be stored in accordance with manufacturer’s instructions at -20°C and dry. The investigator must ensure that the investigational products are stored according to the sponsor’s instructions (temperature, light and humidity) and should control the integrity of the packaging upon receipt. If concerns about the quality or appearance of the investigational products arise, the products may not be dispensed. In this case, the principal investigator must be contacted immediately.

5.5. Drug Accountability, Therapy Compliance and Disposal

The investigator or the site personnel will keep an account of the trial medication and acknowledge the receipt of all shipments of the trial medication. Trial medication will be ordered by the investigator and delivered by the Wirkstoffpeptidlabor to the pharmacy of the participating center. The investigator will document the date of dispensary, subject identification, batch/serial numbers or other identification of trial medication. Upon completion or termination of the study, all unused “mixing kits” have to be returned to the Wirkstoffpeptidlabor of the Department of Immunology. The returned products must be accompanied by adequate documentation and identified clearly with trial site and patient number. The return of any unused study medication must be coordinated by the responsible study monitor/study nurse/pharmacy. Empty packaging does not have to be returned. The disposal is in the responsibility of the study center according to the German laws and local and institutional guidelines and procedures for litter disposal.
In case of SAEs related to the vaccination peptides or adjuvant, the study medication will be returned to the Wirkstoffpeptidlabor of the Department of Immunology, Tübingen for further analysis. The returns will be documented according to SOP.

The returned charges will be locked and deleted according to SOP. A declassification of a drug for clinical use for an application in *in vitro* research experiments is not touched by the declaration. This declassification will be documented. Unused charges of vaccination peptides will be returned to the Wirkstoffpeptidlabor of the Department of Immunology, Tübingen and will be stored.

All waste will be discharged according to German waste laws (date of issue 27.09.1994).

The IMP CoVac-1 may only be applied to subjects included in the P-pVAC-SARS-CoV-2 trial. Other individuals must not receive peptides produced for the P-pVAC-SARS-CoV-2 trial.

Investigational products must be dispensed only by trained and authorized personnel according to legal regulations. Physicians outside the study facility may not apply the study drugs.

5.6. Method of Treatment Assignment

After screening and enrolment, volunteers will be assigned to treatment with CoVac-1.

5.7. Dose Schedule

The CoVac-1 vaccine (500 µl) will be administered subcutaneously. Emulsification will be performed by the pharmacy of the participating center according to the “Anmischanleitung Montanide-Emulsion” provided with the “Mixing Kit” by the Wirkstoffpeptidlabor of the Department of Immunology Tübingen. Final vaccine drug product has to be stored at room temperature and to be administered within 24 h after mixing of the components. For qualification of the pharmacy and study center staff regarding ordering and mixing of the peptide vaccine cocktail with Montanide ISA 51 VG, a controlled dry run process will be performed.

The mixing of the peptide vaccine cocktail and Montanide ISA 51 VG will be performed by local pharmacy and the investigator will be provided with a syringe containing the final drug product CoVac-1. A subcutaneous injection of 500 µl (approx. 250 µg per peptide, 50µg XS15) will be applied. A single vaccination per patient will be conducted.

Vaccination instruction

Peptide vaccines should be injected into the skin at the lower part of the abdomen of the volunteers. The site of vaccination (right or left) will be determined by the investigator. At investigators discretion antihistamins such as 4 mg dimetindene can be applied as i.v. injection or infusion about 30 minutes prior to application of the vaccine.

5.7.1. Dose modifications for peptide vaccine

No dose modification is planned in this trial.

5.7.2. Side effects

5.7.2.1. Side effects of peptide vaccination

Peptide vaccination is generally well tolerated. Mild reactions at local vaccination sites are the most common side effects, followed by fatigue[73] [84]. Peptide vaccination can lead to immediate anaphylactic reactions with elevation of heart rate, hyperhidrosis and subjective feeling of dizziness, in rare cases with concomitant drop in blood pressure[63] [63] [73]. Cutaneous erythema at the vaccination site was observed more frequently and may persist for up to five weeks. Also, there is a risk of granuloma formation. Some of the patients reported one episode of fever not lasting more than two days. No grade III or IV toxicities were observed in former peptide vaccination studies, including an early trial with a peptide based malaria

vaccine, which only reported mild local reactions in approximately 50% of volunteers[63, 70, 73]. Furthermore, no signs for the development of antibody-dependent enhancement (ADE) was reported. Of note, side effects in the reported studies are most likely attributable to the applied adjuvants.

In our ongoing iVAC-CLL01 study using peptide cocktails, most of the patients experienced mild local skin reactions at the vaccination site. No anaphylactic or allergic reaction, or other AE related to the peptide vaccine was observed.

Preliminary safety results of volunteers (n = 12) in part I of the P-pVAC-SARS-CoV-2 study showed as intended and expected developed a local granuloma at injection site in all volunteers (100%). Further local injection site adverse events included transient erythema (100%), swelling (100%), itching (83%), pain (58%) and skin ulceration (8%). Until day 28 no relevant systemic side effects, especially no fever or other inflammatory reactions were reported. No allergic reactions were observed. In some participants fatigue (25%), headache (16%), nausea (16%), myalgia (8%) and arthralgia (8%) were reported.

In the P-pVAC-SARS-CoV-2 study, patients will be monitored for heart rate, blood pressure, temperature and subjective well-being after vaccination for at least 2 hours. The volunteers will be discharged after documentation of these parameters. More detailed information on CoVac-1 vaccine peptides is provided with the current IB (Version 1.0).

5.7.2.2. Side effects of XS15

The TLR 1/2 ligand XS15 will be administered subcutaneously together with the SARS-CoV-2 specific peptides emulsified in Montanide ISA 51 VG. XS15 was never used in a clinical trial before. Common side effects of other TLR ligands used for peptide vaccination are reported to be usually mild, comprising local skin reactions, fatigue, flu-like symptoms like fever, muscular pain and ague. TLR ligands can worsen pre-existing autoinflammatory skin disorders.

Previous application of XS15 in a healthy volunteer and cancer patients (within the scope of individual healing attempts) did, besides local reactions at the vaccination site including formation of granuloma, not cause relevant systemic side effects, in particular no allergic or anaphylactic reactions. More detailed information on XS15 is provided with the current IB (1.0. 27 May 2020).

5.7.2.3. Side effects of Montanide ISA 51 VG

Montanide ISA51 is an oil adjuvant suitable for human injection that will be administered together with the SARS-CoV-2 specific peptides and XS15 subcutaneously. Montanide ISA 51 VG was used as an adjuvant in more than 100 peptide vaccination. Most common side

effects are injection site reaction (68%) including granuloma development, fatigue (54%), fever (41%), gastrointestinal disorders (32%) and injection site or local erythema (28%)[83]. In general, the observed AEs from controlled trials involving non-healthy as well as healthy individuals were mild to moderate in intensity. Further side effects rarely reported were erythema nodosum (2/36 patients, 5%)[85] and the development of sterile abscesses at injection site (10%)[83 86 87].

More detailed information on Montanide ISA 51 VG is provided with the current IB (Version 3291/GB/03/June 2019).

6. Study Procedures and Examination Method

This study will consist of the following consecutive phases: Study entry, vaccination/treatment and follow-up. Time-points and trial procedures are listed in Table 1.

6.1. Study Entry

6.1.1. Volunteer's Informed Consent

Subjects are informed both in writing and verbally by the investigator before any study-specific procedure is performed. Each volunteer will be informed about the modalities of the clinical study in accordance with the provided volunteer information. The volunteer is given sufficient time (≥ 24 h) to consider participation in the clinical trial and to ask for additional advise if needed. Informed consent from the volunteer will be obtained using a form approved by the responsible EC. The volunteer and informing investigator must each personally date and sign the informed consent form containing an integrated declaration on data privacy protection. The original signed document will be part of the investigator's site file and retained with it, a copy including the insurance policy of the trial will be handed to the volunteer. The informed consent process is documented in the volunteer records.

6.1.2. Screening

Screening will be performed within *one* week (7 days) prior to the administration of the CoVac-1 vaccine. After having signed the informed consent form, volunteers will undergo all assessments listed below:

- Demographics
- Medical history
- Enrolment
- Vital signs
- Physical examination
- Concomitant medications
- Hematology (local lab)
- Blood chemistry and coagulation (local lab)
- Urine analysis (local lab)
- Immunoglobulins/Immunophenotype (local lab), approximately 10 ml blood
- Testing for previous or current SARS-CoV-2 infection: 5ml serum blood will be drawn for antibody testing and a nose/throat swab* will be performed.
- HBV, HCV, HIV-1, (local lab)

- Pregnancy test

* If screening takes more than 48 hours, throat/nose swab for SARS-CoV-2 infection must be repeated. The vaccine can only be applied if a negative SARS-CoV-2 PCR test is available on the day of vaccination not older than 48 hours.

The investigator will review all information obtained from the screening procedures via an eligibility form. The investigator will confirm, in writing, whether the subject fulfils all criteria for eligibility. Volunteers who fulfil all the inclusion criteria and none of the exclusion criteria will be eligible to participate in the trial. Screening failures, i.e. screened volunteers not in compliance with all criteria, are to be excluded and the reason will be recorded in the volunteer records.
Information of volunteer's trial participation can be provided to the volunteer's general practitioner if the volunteer agrees.

6.1.3. Enrolment

A volunteer is considered for screening when he or she has signed the Informed Consent form.

In case of confirmation of volunteer's eligibility (volunteers must meet all inclusion criteria and must not meet any exclusion criteria), volunteer will be registered under a specific Vol. ID on a subjects log kept at the trial site. Only these volunteers are enrolled in the study, all others are assessed as screening failures.

The study is open-label.

6.1.4. Randomisation

No randomisation will be done in this clinical trial.

6.1.5. Concomitant Medication and Treatments

Relevant additional medications and treatments administered to the subjects on entry to the trial or at any time during the trial are regarded as concomitant medications and treatments and must be documented on the appropriate pages of the CRF.

6.1.6. Permitted Prior and Concomitant Medications and Treatments

The following concomitant medications and treatments are permitted during the trial.

Part I: No concomitant medication, apart from contraception for FCBP.

Part II : Any concomitant medication (already applied at screening) for e.g. other diseases are allowed except for medications stated in section 6.1.7.

6.1.7. Prohibited Prior and Concomitant Medications and Treatments

The following concomitant medications and treatments are prohibited during the trial:

- Immunosuppressive agents apart from (≤ 10 mg prednisolone or equivalent)
- During the trial, other vaccinations or non-urgent medical interventions are prohibited. Initiation of new medications, regardless of indication must be discussed with the investigator and must be noted on the participant's record.

6.1.8. Contraception

Within this study, all FCBP must have a negative pregnancy test ≤ 7 days prior initiation of study treatment. A FCBP is defined as any female who does not meet the criteria of non-childbearing potential. These are as follows:

- documented hysterectomy, bilateral oophorectomy (ovarectomy), or bilateral tubal ligation
- post-menopausal (a practical definition accepts menopause ≥ 1 year without menses with an appropriate clinical profile, e.g. age > 45 years in the absence of hormone replacement therapy (HRT). In questionable cases, the subject must have a follicle stimulating hormone (FSH) value > 40 mIU/ml and an estradiol value < 40pg/ml.

Sexually active men and women of child-bearing potential must use two methods of reliable contraception including one highly effective (Pearl Index < 1) and one additional effective (barrier) method as described below maintained for up to 3 months after the last dose of study therapy.

The following contraceptive methods with a Pearl Index < 1 are regarded as highly-effective:

- oral hormonal contraception ('pill')

Please note: in case that its efficacy is impaired during the trial, e.g. due to vomiting and diarrhoea, additional/other methods as listed below are required to assure adequate safety

- dermal hormonal contraception/contraceptive plaster
- vaginal hormonal contraception (NuvaRing®)
- long-acting injectable contraceptives/implants that release progesterone (Implanon®)
- tubal ligation (female sterilization)
- intrauterine devices that release hormones (hormone spiral)
- double barrier methods

- partner's vasectomy

Additional effective (barrier) methods are:

- male condom
- diaphragm/cervical cap

The following contraceptive methods are not regarded as safe: condom plus spermicide, simple barrier methods (vaginal pessaries, condom, female condoms), copper spirals, rhythm/basal temperature method and withdrawal method (coitus interruptus).

6.2. Vaccination Phase

Vaccination phase begins as soon as possible (within 7 days) after screening and confirmation of patient's eligibility. If screening takes more than 48 hours, throat/nose swab for SARS-CoV-2 infection must be repeated. The vaccine can only be applied if a negative SARS-CoV-2 PCR test is available on the day of vaccination not older than 48 hours.

Peptide vaccines should be injected into the skin at the lower part of the abdomen of the patients. The site of vaccination (right or left) will be determined by the investigator and documented.

To minimize the risk for severe and unexpected side effects for subjects included in the study, all participants will be monitored for at least two hours after vaccination, including close monitoring of heart rate, blood pressure, temperature, oxygen saturation and subjective well-being. Each monitoring unit must be equipped with a crash cart and an intensive care team should be on standby.

Treatment and monitoring of the first volunteer are performed in an in-patient setting with access to intensive care for 24h. Close monitoring (every 30 minutes vital parameters) will be performed for the first four hours after vaccination. Thereafter, monitoring is performed at hourly intervals until 6 hours after vaccination. Thereafter every 3 hours until 24 hours after application of the vaccine.

6.2.1. Visit 1 (Vaccination) (Day 1)

- Signs/symptoms, baseline
- Vital signs, close monitoring after vaccination (blood pressure, temperature, heart rate and oxygen saturation every 30 minutes for at least 2 hours)
- Physical examination, baseline
- Assessment of concomitant medications

- AE assessment
- Hematology and blood chemistry (local lab), approximately 10 ml blood
- Vaccination (section 5.7)
- T-cell response, baseline obtained before vaccination, approximately 60 ml blood
- Serological response, baseline obtained before vaccination, approximately 15 ml blood

6.2.2. Visit 2 (Day 7 +/- 1)

- Signs/symptoms, as assessed on volunteer’s diary and visit
- Vital signs,
- Physical examination, including investigation of vaccination side
- Assessment of concomitant medications
- AE assessment
- Hematology and blood chemistry (local lab), approximately 10 ml blood
- T-cell response, approximately 60 ml blood
- Serological response, approximately 15 ml blood

6.2.3. Visit 3 (Day 14 +/- 1)

- Signs/symptoms, as assessed on volunteer’s diary and visit
- Vital signs,
- Physical examination, including investigation of vaccination side
- Assessment of concomitant medications
- AE assessment
- Hematology and blood chemistry (local lab), approximately 10 ml blood
- T-cell response, approximately 60 ml blood
- Serological response, approximately 15 ml blood

6.2.4. Visit 4 (Interim safety) (Day 28 +/- 2)

- Signs/symptoms, as assessed on volunteer’s diary and visit
- Vital signs,

- Physical examination, including investigation of vaccination side
- Assessment of concomitant medications
- AE assessment
- Hematology and blood chemistry (local lab), approximately 10 ml blood
- T-cell response, approximately 60 ml blood
- Serological response, approximately 15 ml blood

6.2.5. Visit 5 (End of Safety follow-up = EOSf)

- Signs/symptoms, as assessed on volunteer’s diary and visit
- Vital signs,
- Physical examination, including investigation of vaccination side
- Assessment of concomitant medications
- AE assessments
- Hematology and blood chemistry (local lab), approximately 10 ml blood
- T- cell response, approximately 60 ml blood
- Serological response, approximately 15 ml blood

6.2.6. Visit 6-7 (Follow-up) (Month 3 and 6 +/- 7 days)

- Medical history, anamnestic evaluation of SARS-CoV-2 specific symptoms
- T-cell response, approximately 60 ml blood
- Serological response, approximately 15 ml blood

6.2.7. Volunteer’s diary/card

Each patient included in the P-pVac-SARS-CoV-2 study will receive a volunteer’s card, which states that he/she is participating in the study (Appendix 13.4). This will also include a 24h emergency contact number. Furthermore, each patient will be provided with a volunteer’s diary to note their symptoms daily (Appendix 13.3)

6.2.8. Unscheduled Visit

Subjects may contact the investigator at any time for an unscheduled phone or on-site visit should they experience clinical symptoms or signs following injection. At all unscheduled visits, the following minimum assessment will be performed: Questions concerning the history of the present illness as well as the subject’s general health and lifestyle. Findings

resulting in (S)AEs will be documented and reported as indicated. All other symptoms/signs will be reported on the next scheduled visit on eCRF.

Upon occurrence of symptoms characteristic of SARS-CoV-2 (i. e. cough, fever (cut-off >39°C), loss of taste and smell, limb pain) at any time until day 56, subjects are supposed to get in touch with the investigator. Investigator will initiate SARS-CoV-2 testing for the volunteer (nose or mouth swab followed by PCR per institutional guidelines). If the test is positive, patients should be treated per investigators discretion. Positive results must be recorded as an AESI (section 9.1.4). Negative results will be followed by a second testing ≥ 24h later. Only upon the second negative test, patients are considered negative, all others must be reported as positive.

If participants are positively tested for SARS-CoV-2, all accompanying symptoms and treatments (e.g. hospitalisation, ICU) are recorded

Medically attended AEs and all SAEs will be recorded, and concomitant medication or vaccination will be noted. After identifying the history of the present illness and performing corresponding exams or laboratory tests, the investigator will decide on the best course of treatment according to standard medical practice.

6.3. Assessment of Efficacy

6.3.1. Efficacy Parameters

Immunological Efficacy:

Induction of SARS-CoV-2-specific $CD8^+$ and $CD4^+$ T cells is evaluated using:

- IFN-γ ELISPOT
- Intracellular cytokine staining for TNF and IFN-γ

Induction of SARS-CoV-2 specific antibodies:

- ELISA

6.3.2. Methods and Timing for Assessing, Recording, and Analysing of Efficacy Parameters

Immunological Efficacy:

Serial measurements of immunological efficacy will be performed prior to peptide vaccination (V1), and V2, V3, V4, at the end of study visit and the follow up visits as outlined in table 1. All scheduled visits have a ± 1 day window unless otherwise stated. 75ml peripheral blood (60 ml Na^+-heparin and 15 ml serum) for immunological assays will be obtained prior to vaccination as indicated in table 1. Immunological assays will be performed in the Department of Immunology or the Immunopathological Laboratory, Department of Internal Medicine, University Hospital Tuebingen based on standard SOPs.

Amplification of SARS-COV-2-specific T cells:

PBMCs from volunteers are pulsed the respective peptide and cultured for 12 days adding IL-2 on days 3, 5, and 7. Peptide stimulated PBMCs are analyzed by enzyme-linked immunospot (ELISPOT) assay on day 12 or by flow cytometry-based tetramer and intracellular cytokine staining as described below.

IFN-γ ELISPOT assay

IFN-γ ELISPOT assays are carried out as described previously.[88] In brief, 96-well nitrocellulose plates are coated with anti-IFN-γ. Plates are blocked and PBMCs (*ex vivo* or after T-cell amplification as described above) are distributed to the wells and re-stimulated with HLA class II peptides. Cytokine staining is performed after incubation period. Analysis is performed according to manufacturer’s instructions. Spots are counted using an Immunospot analyzer (according to the cancer immunoguiding program (CIP) guidelines).[89]

To differ between vaccine induced and natural T-cell induction by SARS-CoV-2 infection we will included, beside the T-cell epitopes included in the CoVac-1 vaccine, additional SARS-CoV-2 T-cell epitopes defined in our preclincial work in the peptide readout [24].

Cellular conversion rate (CCR) is calculated by dividing the number of volunteers with an immune response by the number of tested participants to a time point (Visit 2, 3, 4 and 5). A volunteer is considered as having developed an immune response due to immunization if *ex vivo* IFN-γ ELISPOT assay is positive (as described above) and the spot count is at least 2-fold higher than the baseline assay (Visit 1).

Intracellular IFN-γ and TNF staining

The frequency and functionality of peptide-specific $CD8^+$ T cells is analyzed by intracellular IFN-γ or TNF staining as described previously.[88 90] PBMCs are pulsed with individual peptide and incubated in the presence of Brefeldin A and GolgiStop. Cells are labeled using

Cytofix/Cytoperm, CD8, CD4, TNF and IFN-γ coupled to fluorochromes. Samples are evaluated on a FACS analyzer.

Enzyme-linked immunosorbent assay (ELISA)

To identify SARS-CoV-2 antibody responses induced by the vaccine, ELISA assays will be performed using serum samples (15 ml serum tube) obtained at the time points described in Table 1. Specific antibodies against the seven SARS-CoV-2 T-cell epitopes will be assessed by ELISA assay at the Department of Immunology, Tübingen. To differ between vaccine induced antibody response additional standard Elecsys® Anti-SARS-CoV-2 assay supplied by F. Hoffmann-La Roche AG, Basel, Switzerland or ADVIA Centaur SARS-CoV-2 Total (COV2T) (Siemens Healthcare Diagnostics GmbH) will be performed at central laboratory of the University Hospital Tuebingen.

Occurrence or relevant (≥2-fold) increase of SARS-CoV-2 specific IgG antibodies compared to baseline are considered as positive.

In the unlikely event of antibody induction by the CoVaC-1 vaccine, neutralization capacity of antibodies will be assessed by SARS-CoV-2 Pseudovirus Neutralization Assay (CD, Creative Diagnostics®)

6.4. Assessment of Safety

6.4.1. Safety parameters

(Serious) Adverse Events (see section 9)

- Vital signs: pulse, blood pressure, temperature, and weight
- Physical examination including inspection of the vaccination side
- Clinical laboratory evaluations:
  Hematology: white blood cell (WBC), hemoglobin (Hb), platelet count, absolute neutrophil count (ANC), absolute lymphocyte count (ALC)
  Chemistry: AP, total bilirubin, AST/ SGOT, ALT/ SGPT, LDH, and uric acid, CRP, sodium, potassium, calcium, blood urea nitrogen, creatinine, glucose, C-reactive protein
- Concomitant medications
- (S)AEs by NCI CTCAE Version 5.0 and as in appendix 14.5

6.4.2. Methods and Timing for Assessing, Recording, and Analysing Safety Parameters

Serial measurements of safety will be performed at screening and at scheduled intervals throughout the duration of the study as outlined in table 1. All scheduled visits have a ± 1 day window unless otherwise stated. Abnormalities will be captured as protocol deviations. Lab abnormalities grade 1-2 are only considered AE if they fulfill one of the following criteria:

- Accompanied by clinical symptoms.
- Requiring a change in concomitant therapy (e.g. addition or change in a concomitant medication, therapy or treatment).

All Grade 3-4 laboratory abnormalities fulfilling the criteria for an SAE will be reported as SAEs and will be recorded on the AE pages of the CRF; however, those that are not deemed by the investigator to be part of a diagnosis or syndrome will not be reported to the Health Authorities in an expedited manner. Cause of death is to be recorded in the CRF and the subject's medical record.

6.5. Vaccination holding rules

Safety holding rules for each subject will apply throughout the study period until interim safety analysis (V4). Vaccination of further study subjects in the consecutive study phase will not occur until a safety review has been conducted by the DSMB and only by approval a holding rule can be resolved. If a holding rule is activated, the PI will inform the Sponsor within 48 hours. The Sponsor will inform the responsible authorities (PEI and EC).

If the DSMB permits the resumption of injections, a formal request with pertinent data must be submitted to ECs and PEI. The discontinuation of a holding rule should be communicated to all entities in the same manner and timeframe as described above.

The DSMB safety review will consider:

- The relationship of the AE or SAE to the vaccine or other possible causes of the event.
- If appropriate, additional screening or laboratory testing for other volunteers to identify those who may develop similar symptoms and alterations to the current informed consent form will be discussed.

All injected volunteers will be followed for safety until resolution or stabilization (if determined to be chronic sequelae) of their AE.

<u>The holding rules are as follow:</u>

- Solicited local ADRs: If more than 30% of injections are followed by Grade ≥3 solicited swelling or pain or Grade 4 redness (first occurrence at any time after vaccination)and persisting at Grade 3 (swelling or pain)/4 (redness) for > 48 h to maximum 72 hours depending upon symptom severity and kinetics.
- Solicited systemic AEs: If more than 25% of injections are followed by Grade 3 solicited systemic AE beginning within 3 days after study injection (day of injection and 2 subsequent days) and persisting at Grade ≥ 3 for > 48 h to maximum 72 hours depending upon symptom severity and kinetics.
- Unsolicited AEs: If more than 25% of volunteers develop a Grade ≥ 3 unsolicited AE (including laboratory AE and physical observations) that is considered probably or definitely related to injection and persists at Grade 3 for > 48 to maximum 72 hours depending upon symptom severity and kinetics.
- A suspected unexpected serious adverse drug reaction (SUSAR) occurs that is life-threatening or results in death.

6.6. Premature termination of clinical trial for a trial subject

Reasons for premature termination of trial for an individual trial subject are:

1. Death
2. Withdrawal of consent
3. Volunteer lost to follow-up
4. For women, in case of pregnancy

The PI decides about withdrawal of subjects from trial treatment in case of occurrence of criteria mentioned above. In all cases, the reason for withdrawal must be recorded in the CRF and in the subject’s medical records. In case of withdrawal of a subject at his/ her own request, the reason should be determined and documented.

All examinations scheduled for the last trial day will be performed and documented as far as possible, subject to consent of the volunteer. Subjects will enter the regular follow-up of the trial, unless the subject has withdrawn his/her consent to any further study-related procedure. If a subject is withdrawn from all trial-related procedures (including follow-up visits) e.g. at his/her own request, this will not result in any disadvantages for the volunteer.

All ongoing Adverse Events (AEs)/ Serious Adverse Events (SAEs) of withdrawn subjects have to be followed-up until no more signs and symptoms are verifiable or the subject is on stable condition.

Premature termination should be avoided. In case of a premature termination of study, reasons/circumstances and if applicable the final status have to be documented. If volunteers do not withdraw the consent for further follow-up, they should be followed-up as planned.

6.7. Premature closure of a trial site

Premature closure of a trial site has to be considered if:

- The recruitment rate is not sufficient
- The conduct of the study is not compliant with the protocol or the legal regulations, or
- The data quality is not sufficient

The premature closure of a site will be decided by the sponsor.

Site principal investigators may terminate his/her participation in the study. If this occurs they should provide a written statement of the reasons for terminating participation and must provide the sponsor with all available and up-to-date study data.

The sponsor may also decide to terminate participation of an investigator or study centre for the following reasons:

- Breach of agreement
- Serious non-compliance to protocol or the legal regulations
- Insufficient volunteer recruitment

If a participating center closes, or is closed, prior to termination of the whole trial, the sponsor expects that data from volunteers already entered into the trial will be reported as per protocol. Details on further treatment and follow-up of volunteers on study have to be discussed with the site principal investigator.

6.8. Premature termination of the trial

The trial may be prematurely terminated, if in the opinion of the sponsor and coordinating investigator, there is sufficient reasonable cause. Written notification documenting the reason for study termination will be provided to the investigators.

In case of the following situations a premature termination of the trial has to be considered:

- Observation of one SAE associated with administration of CoVac-1 (Statistical Stopping rule of the study)
- Serious adverse drug reactions / not justifiable toxicity
- Substantial changes in risk-benefit considerations

- New insights from other trials
- Insufficient efficacy
- Insufficient recruitment rate

The DSMB will monitor the study conduct and the safety aspects of the trial on a regular basis, and will give recommendations to the coordinating investigator/ the sponsor whether to stop the trial or to change the trial protocol. The sponsor will then decide on the actions to be taken. According to the German drug law (§42a), the trial may be suspended or prematurely terminated by decision of the competent authority (PEI).

6.9. Follow Up

Volunteers will be followed for up to 4 months after EOSf. Thereafter patients may be contacted by phone call/e-mail to assess infection with SARS-CoV-2.

6.10. End of Study for Subjects

The end of Study for a subject enrolled in this trial is defined as the last study visit.

7. Quality control and Quality assurance

7.1. Risk-based approach

During protocol development, processes and data that are critical to ensure human subject protection and the reliability of trial results were identified.

The identified risks were evaluated against existing risk controls by considering:

- The likelihood of errors occurring
- The extent to which such errors would be detectable
- The impact of such errors on human subject protection and reliability of trial results.

In case of unacceptable risks, risk reduction activities were defined and incorporated e.g. in the protocol, monitoring plan and agreements.

Results will be communicated to those who are involved in or affected by such activities.

The sponsor periodically reviews risk control measures to ascertain whether the implemented activities remain effective and relevant, taking into account emerging knowledge and experience.

7.2. Monitoring

Monitoring for this study is provided by the *Zentrum für Klinische Studien Tuebingen (ZKS Tuebingen).* The monitoring will be conducted according to *ZKS Tuebingen Internal Standard Operating Procedures (SOPs)* and a dedicated monitoring manual for the study. The monitoring timelines include, for all centres, initiation visit, regular monitor visits during the course of the trial as well as a close out visit. Usually, monitoring will end with the last visit after full documentation of the last volunteer enrolled (close out visit). All investigators agree that the monitors regularly visit the trial site, assure that the monitors will receive appropriate support in their activities and will have access to all trial-related documents.
The aims of the monitoring visits are as follows:

- Check informed consent documents
- Monitor trial subject safety (occurrence and documentation/reporting of Serious Adverse Events (SAEs) and Adverse Events (AEs)).
- Check completeness and accuracy of entries on the CRFs.
- Validate entries on the CRFs against those in the source documents (source data verification (SDV)).

- Check the Drug Account
- Check the storage conditions of the IMP
- Evaluate the progress of the trial
- Evaluate compliance with the trial protocol
- Assess whether the trial is being performed according to GCP at the trial site
- Discuss with the investigator aspects of trial conduct and any deficiencies found
- A monitoring visit report is prepared for each visit describing the progress of the clinical trial and any problems

7.3. Audits/ Inspections

In addition to the monitoring activities, audits can be conducted by the sponsor or assigned auditors. These audits may include checking the whole course of the study, documentation, trial centre, investigators and the monitor.

The competent regulatory authorities may also conduct inspections.

With his/her participation in the study, the investigator agrees to support the activities of the auditor/inspector, provide her/him with direct access to the source documents, study documentation and give her/him the opportunity to audit/inspect the study site, laboratory facilities, storage of the investigational product, etc.

7.4. Documentation: Collection, Handling, Storage and Archiving of Data

7.4.1. Case Report Form

The trial Case Report Form (CRF) is the primary data collection instrument for the trial. All data requested on the CRF must be recorded. All missing data must be explained.

For this project, electronic Case Report Forms (eCRFs) will be used. The Clinical Data Management System [secuTrial “SecuTrial”] will be used for data capture, processing and storage of study data. Data entry is performed at the investigational site by clinical staff after having received training and a user manual for the electronic CRF. Training and the user manual will detail procedures to be followed in case of technical problems. Queries resulting from edit checks and/or data verification procedures will be posted electronically in the eCRF.
The Clinical Trial Data Management System (CDMS) is validated and changes are tracked via an audit trail.

The correctness of entries in eCRFs will be confirmed by dated signature of an authorized investigator. The Principal investigator is responsible for ensuring that all sections of the eCRF are completed correctly and that entries can be verified against source data. The Principal investigator has to verify the eCRFs via dated electronic signature after completion of the eCRF.

7.4.2. Source Data

Source data is all information, original records of clinical findings, observations or other activities in a clinical trial necessary for the reconstruction and evaluation of the trial. Source data are contained in source documents. Examples of these original documents and data records include: hospital records, clinical and office charts, laboratory notes, memoranda, volunteers' diaries or evaluation checklists, pharmacy dispensing records, recorded data from automated instruments, copies or transcriptions certified after verification as being accurate and complete, x-rays, CTs, MRIs, ultrasound reports, volunteer files, and records kept at the pharmacy, at the laboratories, and at medico-technical departments involved in the clinical trial.

7.4.3. Data Handling

Authorized clinical staff at the investigational site will enter the data into the eCRF using an access controlled, audit-trailed, ICH/GCP compliant, validated system. Entered data will be subjected to plausibility checks directly implemented in the eCRF, monitoring and medical review. Implausible or missing data will be queried. Database lock will be performed after completion of data entry, data cleaning and a final data review.

7.4.4. Preparation/Handling/Storage/Accountability of biological samples

Biological samples collected under this protocol may be used in accordance with the study informed consent form to conduct protocol related safety and immunogenicity evaluations, exploratory laboratory evaluations related to the SARS-CoV-2 infection the vaccine was designed to prevent, exploratory laboratory evaluations related to vaccine research in general and for research assay validation. All biological samples obtained within the study will be identified solely by means of the individual identification code (Patient ID). Samples will be either processed directly or for PBMC and serum samples for immunogenicity analysis stored until further analyses. Storage of biological samples on a computer will be done in accordance with local data protection law and will be handled in strictest confidence.

For protection of these data, organizational procedures are implemented to prevent distribution of data to unauthorized persons. The appropriate regulations of local data legislation will be fulfilled in its entirety. Samples are stored at the Department of Immunology, Tuebingen. Only investigators or their designees will have access to the samples and corresponding data. Sample tracking and preparation will be performed according to established standard operating procedures. The biological samples will be destroyed at the latest 30 years after the end of the study. If a study subject withdraws consent to participate in the study all samples taken and identifiable are destroyed without prior analysis if requested.

7.4.5. Handling of missing data and drop outs

Missing values will be predicted based on plausible assumptions that account for the uncertainty due to missing data. For patients with unknown status for the primary endpoint, i.e. a volunteer without complete follow-up and without any SAE until the last known study site contact, a detailed report on the course should be presented by the investigator and discussed concerning probable unknown SAEs and the reasons for drop-out. If substantial reason will be found that the person could have experienced a SAE, this will be interpreted as failure and the recruitment should be stopped accordingly. Otherwise the safety of the person will be interpreted as success, i.e. the subject will be interpreted to have not experienced a SAE. If this decision cannot be precisely concluded, patient will be considered as drop-out. All missing data or inconsistencies will be resolved by the responsible investigator.

7.4.6. Storage and Archiving of Data

According to the EU Clinical Trial Regulation 536/2014 all essential trial documents (e.g. CRF) will be archived for at least 25 years after the trial termination. The investigator(s) will archive all trial data (source data and Investigator Site File (ISF) including subject identification list and relevant correspondence) according to the Guideline ICH GCP (E6) and to local law or regulations.

8. Statistical Analyses

8.1. Study Population Definition

8.1.1. Sample Size and Power Consideration

In this phase I study the safety/toxicity of one vaccination will be investigated. For this purpose, it will be investigated whether the incidence of severe adverse events (SAE) associated with administration of CoVac-1 exceeds a predetermined rate of 5% (= P1 = alternative hypothesis) in the whole study population. Safety of the CoVac-1 vaccine is shown if no SAE (= P0 = null hypothesis) occurs in the study population. An evaluable sample size of 33 achieves 81.6% power to detect a difference (P1-P0) of 0.0499 using a one-sided exact test based on the binomial distribution with a target significance level of 0.05. The actual significance level achieved by this test is 0.003. These results assume that the population proportion under the null hypotheses (P0) is 0.0001. Assuming a dropout rate of 7.5% (percentage of subjects that are expected to be lost at random during the course of the study and for whom no response data concerning existence of SAE will be collected, i.e. will be treated as "missing") the total number of 36 subjects should be enrolled in the study in order to end up with 33 evaluable subjects. Sample size computed using PASS 2020 (NCSS, LLC, Kaysville, Utah, USA).

8.2. Analysis Primary Variables

The occurrence of critical events (SAE) associated with administration of CoVac-1 should be reported to the Sponsor (section 9.3.1) and documented immediately in the eCRF (within 48h). The statistical center will evaluate the occurrence of critical events using automatized alerts of the e(CRF) on a daily basis and distribute this information to the Sponsor/DSMB. If one critical event will be observed, the formal statistical stopping rule of the study is reached and no further recruitment is adequate. Otherwise the safety of the procedure will be accepted, if no out of 33 volunteers will experience a critical event.

No further statistical tests with confirmatory aim are planned.

8.3. Analysis Secondary Variables

<u>Safety</u>

The statistical analysis of the secondary endpoint will be done in a descriptive manner. No statistical tests with confirmatory aim are planned. The toxicity and safety will be described by absolute and relative frequencies using CTCAE V5.0-scoring.

Efficacy

The rate of patients with induction of peptide-specific T-cell responses within a maximum of 56 days after vaccination will be the secondary endpoint for efficacy. T-cell responses will be assessed as described in section 6.3.1

The rate of patients with induction of antibody responses within a maximum of 56 days after vaccination will be the secondary endpoint for efficacy. The antibody response will be assessed as described in section 6.3.1

8.4. Subgroup Analysis

Exploratory subgroup analyses are planned for each part (I and II) regarding primary and secondary endpoints.

8.5. Interim Analysis

The primary endpoint will be evaluated in a sequential manner after every consecutive included volunteer has reached day 28. No further formal interim efficacy analysis will be performed during the conduct of the study.

8.6. Stopping Rules

The pre-defined stopping rule for the study is reached if one critical event (SAE as defined in section 9.1.5) associated with administration of CoVac-1 will be observed in the study population resp. if the first critical event will be observed.

The sponsor has the right to terminate the trial prematurely if there are any relevant medical or ethical concerns, or for reasonable administrative reasons. If such action is taken, the reasons for terminating the trial have to be documented in detail. All volunteers who are not considered end of study must undergo a final examination, which must be documented.

Criteria for termination of the study as a whole are:

- An unacceptable profile or incidence rate of adverse events/ adverse events of special interest revealed in this or any other study in which at least one of the investigational products of this trial is administered.
- Significant number of cases of death associated with the study treatment.

- Any other factor that in the view of the sponsor constitutes an adequate reason for terminating the study as a whole.

The Sponsor has to be informed without delay if any investigator has ethical concerns.

8.7. Biometric Report

The biometric report lies within the responsibility of the biostatistician of the clinical trial. The sponsor has to make every effort to acquire a complete data set for statistical analysis. The trial report has to be completed within a reasonable time.

9. Safety

9.1. Definition of Adverse Events and Side Effects

9.1.1. Adverse Events

Any untoward clinical relevant medical occurrence in a volunteer or clinical investigation subject to whom a pharmaceutical product had been administered and which does not necessarily have a causal relationship with this treatment. An AE can therefore be any clinical relevant unfavorable and unintended sign (including an abnormal laboratory finding), clinical relevant symptom, or disease temporally associated with the use of a medicinal (investigational) product, whether or not related to the medicinal (investigational) product.

An AE may be:

- New clinical relevant symptoms/ medical conditions
- New clinical relevant diagnosis
- Clinical relevant changes of laboratory parameters
- Diseases and medical consequences of an accident
- Worsening of medical conditions/ diseases existing before clinical trial start
- Recurrence of disease
- Clinical relevant increase of frequency or intensity of episodical diseases

A pre-existing disease or symptom will not be considered an AE unless there will be an untoward change in its intensity, frequency or quality. This change will be documented by the investigator.

In general, abnormal laboratory findings or clinical events without clinical significance (based on the investigator´s judgement) should not be recorded as AEs.

Surgical procedures themselves are not AEs; they are therapeutic measures for conditions that require surgery. The condition for which the surgery is required may be an AE. Planned surgical measures permitted by the clinical trial protocol and the condition(s) leading to these measures are not AEs, if the condition leading to the measure was present prior to inclusion into the trial.

AEs are classified as "non-serious" or "serious".

9.1.2. Adverse Drug Reaction

An Adverse Drug Reaction (adverse reaction: undesirable effect) is a response to a medicinal product which is noxious and unintended. Adverse reactions may arise from use of the product within or outside terms of the marketing authorisation or from occupational

exposure. Use outside the marketing authorisation includes off-label use, overdose, misuse, abuse and medication errors.

An unexpected Adverse Drug Reaction (ADR) is a reaction which nature or severity is not consistent with the applicable product information available for the IMP.

Expected ADRs arelisted in the appropriate reference documents, e.g. Investigator's Brochures; and below:

A solicited AE/ADR is a predetermined event, which may reflect safety concerns related to the investigational product and is, at least for the local solicited AEs, expected. The solicited ADR/AEs (local and systemic) for this study include:

Local solicited ADRs:

- Swelling at site of injection
- Erythema at site of injection
- Pain or itching at site of injection
- Formation of granuloma at the injection site
- Superficial skin ulceration

Systemic solicited AEs:

- Fever
- Chills
- Myalgia (described to the subject as generalized muscle aches)
- Arthralgia (described to the subject as generalized joint aches)
- Fatigue
- Headache
- Gastrointestinal symptoms (loss of appetite, nausea, vomiting, abdominal pain, and/or diarrhoea)

A grading for severity of ADRs can be found in appendix 14.5 as guidance.

9.1.3. Expectedness

An 'unexpected' adverse event is one the nature or severity of which is not consistent with the applicable product information, e.g. Investigator's Brochure (IB). Furthermore, reports

which add significant information on specificity or severity of a known adverse reaction are counted as ‘unexpected’ events.

9.1.4. AESI (adverse events of special interest)

An adverse event of special interest (AESI), serious or non-serious, is one of scientific and medical concern specific to the sponsor’s product, for which ongoing monitoring and rapid communication (≤ 48 hours) by the investigator to the sponsor can be appropriate. Such an event might warrant further investigation in order to characterize and understand it. Depending on the nature of the event, rapid communication by the trial sponsor to other parties (e.g. regulators) might also be warranted (adapted from CIOMS 2005).

In case of the CoVac-1 vaccine in this study, AESIs include proven SARS-CoV-2 infection and potential immune mediated diseases (pIMDs, see Appendix 14.6)[91]. Instructions for management are provided in section 6.3.

With regard to trial schedule and AESI occurrence, AESIs constitute:

- Novel proven (PCR-based) SARS-CoV-2 infection accompanied by symptoms
- Novel proven (PCR-based) SARS-CoV-2 positivity without symptoms
- Novel potential immune mediated diseases (pIMD) according the listed diseases in Appendix 14.6
- Formation of granuloma at the injection site

AESIs are always to be addressed as part of the patient safety report to the DSMB (section 1.3), also non-occurrence will be mentioned. Depending on the decision of DSMB, the vaccination of further volunteers will be permanently stopped.

9.1.5. Serious Adverse Event and Serous Adverse Reaction

AEs are classified as "non-serious" or "serious".

A serious adverse event (SAE) is one that at any dose:

- Results in death.
- Is life-threatening (the term life-threatening refers to an event in which the subject was at risk of death at the time of event and not to an event which hypothetically might have caused death if it was more severe).
- Requires subject hospitalization or prolongation of existing hospitalization.
- Results in persistent or significant disability/ incapacity.
- Causes a congenital anomaly / birth defect.
- Is medically significant (e.g. suspected transmission of an infectious agent via medicinal product). Moreover, there are other situations - such as important medical events that may not be immediately life threatening or

result in death or hospitalization, but may jeopardize the patient or may require intervention to prevent one of the other outcomes listed above.Important medical event [ICH E2A; EMA/155528/2018]: Examples of such events are intensive treatment in an emergency room or at home for allergic bronchospasm; blood dyscrasias or convulsions that do not result in hospitalisation; development of drug dependency or drug abuse (Important medical event terms list (MedDRA ≥ version 23.0).

9.2. Period of Observation

For the purpose of this trial, the period of observation for collection of AEs extends from the time of administration of the IMP until Visit 5.
All AEs that occur in the course of a clinical trial regardless of the causal relationship must be monitored and followed up until the outcome is known or no more information is achievable.

9.3. Documentation and Reporting of Adverse Events

9.3.1. Documentation and Reporting of Adverse Events by the Investigator

The investigator must document all AEs that occur during the observation period set in this protocol on the pages provided in the case report form. Additional instructions may be provided in the investigator file and in the case report form itself. The following approach will be taken for documentation:
All AEs (whether serious or non-serious) must be documented on the “adverse event” page of the eCRF.
If the AE is serious, the investigator must complete, in addition to the “adverse event” page in the case report form, a “serious adverse event report form” at the time when the SAE is detected. The investigator will document the date when he/she or any employee was first aware of the report. The initial report must be as concise as possible, including reported terms according to “Common Terminology Criteria for Adverse Events (CTCAE)-List” (one term per event), details of the current illness and (S) AE, severity, serious criteria as well as an assessment of the causal relationship between the event and the trial medication.
SAE reports (initial and follow-up reports), even if they are incomplete, should be send within 24 hours upon receipt to representative of the Sponsor:

[redacted]

9.3.2. Assessment of Severity and Causality

The investigator will also provide an assessment of the severity of the event according to CTCAE criteria (Version 5.0) and causal relationship between the event and each of the investigational products or trial procedures.

AEs and SAEs should be evaluated for severity according to the following scale:

- Grade 1 Mild; asymptomatic or mild symptoms; clinical or diagnostic observations only; intervention not indicated.
- Grade 2 Moderate; minimal, local or noninvasive intervention indicated; limiting age-appropriate instrumental Activities of Daily Living (ADL).
- Grade 3 Severe or medically significant but not immediately life-threatening; hospitalization or prolongation of hospitalization indicated; disabling; limiting self care ADL.
- Grade 4 Life-threatening consequences; urgent intervention indicated.
- Grade 5 Death related to AE.

The investigator must determine the causal relationship between the administration of IMP and the occurrence of an AE/SAE as defined below:

Related: There is a reasonable possibility that the SAE may be related to the IMP (e.g. favorable temporal relationship, positive dechallenge: symptoms are receding when IMP is withdrawn or the dose reduced, positive rechallenge: symptoms are reappearing when the IMP is reintroduced or the full dose is re-administered)

Not Related: There is no reasonable possibility that the SAE is related to the IMP (e.g. there is a plausible alternative cause for the SAE that better explains the occurrence of the SAE)

Outcome of AEs

The outcome of an AE at the time of the last observation will be classified as:

**Recovered/ resolved**	All signs and symptoms of an AE disappeared without any sequels at the time of the last interrogation.
**Recovering/ resolving**	The intensity of signs and symptoms has been diminishing and/ or their clinical pattern has been changing up to the time of the last interrogation in a way typical for its resolution.
**Not recovered/ not resolved**	Signs and symptoms of an AE are mostly unchanged at the time of the last interrogation.
**Recovered/**	Actual signs and symptoms of an AE disappeared but there are sequels

**resolved with sequel**	related to the AE.
**Fatal**	Resulting in death. If there are more than one AE, only the AE leading to death (possibly related) will be characterized as ‘fatal‘.
**Unknown**	The outcome is unknown or implausible and the information cannot be supplemented or verified.

9.3.3. Action taken

No action will be taken with regards to the IMP as the vaccine is applied only once.

9.3.4. Sponsors Assessment of the SAEs

All SAE will be subject to a second assessment by the trial Sponsor or authorized second assessors, e.g. CI.

The second assessor will fill out a ‘Second Assessment Form’ for each SAE containing.

- Event serious yes/no
- Relationship between SAE and IMP/study procedure
- Expectedness of SAE according to the reference document: IB CoVac-1 peptide vaccine V1.0 dated 22.5.2020.
- Benefit / risk assessment for the trial regarding change as a result of SAE.

9.3.5. Follow-up of Initial Report

Information not available at the time of the initial report (e.g. end date for the AE or laboratory values received after the report) must be documented on a “Serious Adverse Event” form with the box “Follow-up” checked under “Report type”.

All volunteers who have AEs, whether considered associated with the use of the investigational products or not, must be monitored to determine the outcome as far as possible. The clinical course of the AE will be followed up according to accepted standards of medical practice even after the end of the period of observation, until a satisfactory explanation is found or the investigator considers it medically justifiable to terminate follow-up. Should the AE result in death, a full pathologist’s report should be supplied, if possible.

The sponsor will identify missing information for each SAE report and will require follow up information in regular intervals from the investigators until all queries are resolved or no further information can be reasonably expected. All responses to queries and supply of

additional information by the investigator should follow the same reporting route and timelines as the initial report.

9.3.6. Exception of reporting

As this is a prophylactic vaccination trial with application of CoVac-1 in healthy adults, no exception of reporting for AEs are made.

9.3.7. Suspected Unexpected Serious Adverse Reaction (SUSAR)

SAEs that are both suspected, i.e. possibly related to IMP, and 'unexpected', i.e. the nature and/ or severity of which is not consistent with the applicable product information are to be classified as Suspected Unexpected Serious Adverse Reactions (SUSARs).

In case that either the investigator who primarily reported the SAE, or the second assessor classify the SAE as 'suspected' (*i.e. not as "definitely not related to IMP")* and the SAE is also unexpected, it will be categorized as a SUSAR.

All SUSARs are subject to an expedited reporting to the responsible ethics committee(s), the competent higher federal authority (PEI) and to all participating investigators.

9.3.8. Expedited Reporting to the Regulatory Authorities

Fatal and life-threatening SUSARs

The competent authority (PEI) and the EC responsible must be informed by the Sponsor of all fatal or life-threatening SUSARs. This must be done immediately, at the latest seven calendar days after becoming aware of the minimum criteria for reporting. In all cases, attempts must be made to obtain further relevant information, which must be supplied to the competent authority and the EC in overall charge within a further eight days. Furthermore, if a trial subject dies, this information must be additionally passed on to the EC responsible for the region in which the death occurred.

SUSARs that are not fatal or life-threatening

The authority (PEI) and the EC responsible will be informed without delay by the sponsor or CI of all SUSARs, at the latest within 15 calendar days of becoming aware of the minimum criteria for reporting. Further relevant details will be passed on as soon as possible.

If the information at the time of reporting is incomplete, further information to enable adequate assessment of the case will be requested from the reporter or other available sources.

9.4. Examination and Report of Changes in the Risk to Benefit Ratio

Without delay, and at the latest within 15 days of the decision for the need to do so, the Sponsor / CI will inform the competent authority (PEI), the EC responsible of any events or factors that could result in a review of the risk-benefit ratio of the IMP. These consist especially of:

- Individual reports of expected serious ADRs with an unexpected outcome.
- A clinically relevant increase in the rate of occurrence of expected ADRs.
- SUSARs in trial subjects who have already completed the follow-up period of the clinical trial (”end-of-trial visit”).
- Factors emerging in connection with trial conduct or the development of the IMP that may affect the safety of persons concerned.

9.4.1. Reporting to Data and Safety Monitoring Board

The DSMB will be informed of all safety-relevant events by the Sponsor / CI. An interim safety analysis will be sent to the DSMB after completion of Part I and Part II. The DSMB will decide on trial continuation. Additionally, the DSMB will be informed as soon as a IMP-related SAE/SUSAR occurs or a holding rule is reached. Meetings may be convened as conference calls/Emails as well as in person.

9.4.2. Report to the Investigator

The Sponsor / CI will inform investigators of all SUSARs including all relevant further information within the periods set by the authority.

If new information becomes known that is different from the scientific information given to the investigator, all investigators will be informed of this by the sponsor.

9.5. Interim Safety analysis

Two or more interim safety analyses will be undertaken to guide decision and whether to start recruitment in the consecutive trial parts. Upon completion of a study part, screening will be interrupted until safety approval of DSMB is available. The data to be evaluated by the DSMB will include (report):

- Solicited and unsolicited AEs/ADRs, AESIs and SAEs
- Review and, if necessary, assessment of (S)AE relatedness to IMP

The DSMB decision will be documented in a TMF. The information will be distributed to the study sponsor, the drug manufacturer, all investigators/trial site and the ZKS Department Pharmakovigilanz for information.

The interim safety analysis together with the DSMB decision and first data on immunogenicity of CaVac-1 will be send to the authorities (PEI and ethic committee) as a substantial amendment to gain approval for recruiting in Part II and III of the planned study. After responsible authorities approve the submitted documents, the study will continue enrolment as planned.

9.6. Annual Safety Report

Once a year, the Sponsor / CI will supply a report on the safety of trial subjects with all available relevant information concerning volunteer safety during the reference period to the competent authorities. Information required for this purpose will be made available to the ZKS by the Sponsor/ CI at the reporting date. This report will also be supplied to the responsible ethics committee.
The annual safety report will be compiled according to the corresponding ICH guideline E2F „Development Safety Update Report – DSUR“. The safety report will cover all IMPs used in this study.

9.7. Deviations from the Protocol

Any significant deviation from the protocol will be noted.

The PI or a nominated person will evaluate this deviation from the protocol and will decide on the further course of the trial for the respective subject.

9.8. Reporting of Pregnancy

<u>Maternal exposure</u>

If a volunteers becomes pregnant during the course of the study related procedures have to be discontinued immediately.

The outcome of any conception occurring from the date of the vaccination until 1 month after the application should be followed up and documented.

Pregnancy itself is not regarded as an AE unless there is a suspicion that the investigational product under study may have interfered with the effectiveness of a contraceptive

medication. Congenital abnormalities/birth defects and spontaneous miscarriages should be reported and handled as SAEs. Elective abortions without complications should not be handled as AEs. The outcome of all pregnancies (spontaneous miscarriage, elective termination, ectopic pregnancy, normal birth or congenital abnormality) should be followed up and documented even if the patient was withdrawn from the study.

If any pregnancy or suspected pregnancy occurs in the course of the study, it must be reported to ZKS Tuebingen, department pharmacovigilance (on behalf of sponsor) immediately by fax (fax-number: + 49 (0)7071 29 25205) or mail (zks-pv@med.uni-tuebingen.de) on the Pregnancy Report Form.

All pregnancies should be followed up and documented, even if the patient was withdrawn from the study, until outcome (spontaneous miscarriage, elective termination, ectopic pregnancy, normal birth or congenital abnormality). The outcome must be notified immediately by the investigator to the ZKS Tuebingen, department pharmacovigilance (on behalf of sponsor) within 24 hours of first knowledge as a follow-up to the initial report.

For any event during the pregnancy which meets a seriousness criterion, the Investigator will also follow the procedures for reporting SAEs (complete and send the SAE form to the Sponsor by fax within 24 hours of the Investigator's knowledge of the event).
All neonatal deaths that occur within 30 days of birth should be reported, without regard to causality, as SAEs. In addition, any infant death at any time thereafter that the Investigator suspects is related to the exposure to the study drug/IMPs should also be reported to the Sponsor by facsimile within 24 hours of the Investigators' knowledge of the event.

The same timelines apply when outcome information is available.

If the female is found not to be pregnant, continuation of the volunteer within the study will be determined by the investigator(s).

Paternal exposure

Male patients should refrain from fathering a child or donating sperm during the study and for 3 months following the vaccination.

Pregnancy of the patient’s partners is not considered to be an AE. However, the outcome of all pregnancies (spontaneous miscarriage, elective termination, ectopic pregnancy, normal birth or congenital abnormality) should if possible be followed up and documented.

The outcome of any conception occurring from the date of the first dose until 3 months after the last dose should be followed up and documented.

Information on pregnancy must be collected on the “Pregnancy Reporting Form”. In order for Sponsor or designee to collect any pregnancy surveillance information from the female

partner, the female partner must sign an informed consent form for disclosure of this information.

10. Regulatory Consideration

10.1. Ethical Conduct of Clinical Study

10.1.1. Good Clinical Practice, Declaration of Helsinki and legal Provision

The procedures set out in this trial protocol, pertaining to the conduct, evaluation, and documentation of this trial, are designed to ensure that all persons involved in the trial act according to Good Clinical Practice (GCP) and the ethical principles described in the applicable version of the Declaration of Helsinki.

10.2. Subject Information and Informed Consent

Each volunteer will be informed about the modalities of the clinical study in accordance with the provided volunteer informed consent (IC). The volunteer is to be informed both in writing and verbally by the investigator before any study-specific procedure is performed. The volunteer must be given sufficient time to decide whether to participate in this comparative study and to ask questions concerning this trial. It must also be made clear to the volunteer that he / she can withdraw from the study at any time without giving reasons and that he / she will not be in any way disadvantaged for this. The subject must give consent in writing. The volunteer and informing physician must each personally date and sign the informed consent form with an integrated declaration on data privacy protection, whereby the physician must not sign before the volunteer. Original signed documents will be part of the investigator's file and retained with it. A copy of the signed informed consent document and study insurance policy must be given to the subject. The documents must be in a language understandable to the subject and must specify who informed the subject. The subjects will be informed as soon as possible if new information may influence his/her decision to participate in the trial. The communication of this information should be documented in the volunteer chart.

10.3. Insurance

Each volunteer is insured against any health impairment occurring as a result of participation in the study in accordance with the laws and regulations of the "German Arzneimittelgesetz". The insurance is covered by *HDI Global SE, Am Schönenkamp 45, 40599 Düsseldorf, Policy* number 57 010311 03013/03052 and valid throughout the conduct of the study including follow-up for each individual volunteer. A copy of the insurance policy and conditions are distributed to the volunteer upon enrolment into the study and the volunteer is advised to adhere to the conditions of the insurance policy to safeguard a valid volunteer insurance.

Travel insurance will be included for all volunteers enrolled in the clinical trial.

10.4. Confidentiality

The data obtained in the course of the trial will be treated according to the European General Data Protection Regulation (Datenschutz-Grundverordnung; DS-GVO) and the applicable local data protection regulations as well as the AMG.

Subjects have to be informed about data protection in the clinical trial and to consent in writing to collect and process their personalized data as well as to transfer their pseudonymized data. The information has to be transparent, precise, easily accessible and understandable and is written in clear and simple language. The written privacy policy must be approved by the responsible ethics committee.

In order to maintain volunteer privacy, all data capture records, study drug accountability records, study reports and communications will identify the volunteer by the assigned volunteer number. The PI determines which persons are authorized to view personal data, the Volunteer Intification Log is only accessible to authorized study team members. Access rights to personal data (including pseudonymised data) are available to prevent unauthorized access to the data (both electronically and physically). Electronic systems and files are access-regulated, possibly password-protected. Documents and files are kept in lockable rooms, if necessary, cupboards with access control.

The volunteer name, initials and the full birth date should never be used in any correspondence with the Sponsor or on the Case Report Forms. The investigator will grant monitor(s) and auditor(s) and/or regulatory authorities direct access to the volunteer's original medical records for verification of data gathered on the data capture records and to audit the data collection process. Direct access includes examining, analyzing, and verifying any recorded data and reports that are important to the evaluation of the monitoring. The investigator is obliged to inform the volunteer that his/her trial-related records will be viewed without violating their confidentiality and that the collected information will only be made publicly available to the extent permitted by the applicable laws and regulations. All data will be stored either paper-based or electronically in a pseudonymous manner and handled strictly confidential. The investigators are obliged to keep all study data and information confidential and to use those data only in context with the persons involved in the trial conduct. Study material or information developed in this trial must not be available to third parties, except for official representatives of the sponsor or regulatory authorities.

Data will be processed at the study site according to the written safety concept of this institution. Access to the data will be strictly limited to authorized persons. Loss of data is excluded due to extensive back-up procedures. All legal requirements concerning data protection and confidentiality will be respected. All authorized persons are sworn to secrecy.
In the case of withdrawal of consent the stored data collected to this time point will be stored and further used. Data not necessary any longer are deleted immediately.
Collected study data will be stored for at least 25 years after the end of the trial, if there are no other regulatory archiving periods. After archiving has expired, the data will be destructed in a data protection compliant manner.

When processing personal data, the following principles must be observed (pursuant to DS-GVO Article 5 “Principles relating to processing of personal data“):

Personal data shall be:

- processed lawfully, fairly and in a transparent manner in relation to the data subject
- collected for specified, explicit and legitimate purposes and not further processed in a manner that is incompatible with those purposes
- adequate, relevant and limited to what is necessary in relation to the purposes for which they are processed
- accurate and, where necessary, kept up to date
- kept in a form which permits identification of data subjects for no longer than is necessary for the purposes for which the personal data are processed
- processed in a manner that ensures appropriate security of the personal data, including protection against unauthorised or unlawful processing and against accidental loss, destruction or damage, using appropriate technical or organisational measures

10.5. Responsibility of the Investigator

The investigator should ensure that all persons assisting with the trial are adequately informed about the protocol, any amendments to the protocol, the trial treatments, and their trial-related duties and functions.
The investigator should maintain a list of subinvestigators and other appropriately qualified persons to whom he or she has delegated significant trial-related duties.

10.6. Registration of the Trial

Prior to the beginning of the clinical phase (First Patient In) the Sponsor / CI will register the trial in the EudraCT (2020-002502-75) as well as ClinicalTrials.gov Database.

10.7. Continuous Information to Independent Ethics Committee

According to the German Drug Law (AMG) and the GCP Ordinance, the EC and the competent authority (Paul-Ehrlich Institut, PEI) will be informed of all suspected serious unexpected adverse reactions (SUSARs). Both institutions will be informed in case the risk/ benefit assessment did change or any others new and significant hazards for subjects' safety or welfare did occur. In addition, upon activation and prior to discontinuation of a holding rule the sponsor informs the responsible authorities (section 6.5). Furthermore, a report on all observed SAEs will be submitted once a year – Annual Safety Report.
The EC and the regulatory authorities must be informed of the end of the trial. They will be provided with a summary of trial results within one year after the end of clinical phase.

10.8. Approval of Protocol and Subsequent Amendments

Before the start of the trial, the trial protocol, informed consent document, and any other appropriate documents will be submitted to the independent EC as well as to the competent authority (PEI). A written favourable vote of the EC and an (implicit) approval by the competent higher federal authority (PEI) as well as the notification of the local authorities (acc. to §67 AMG) are a prerequisite for initiation of this clinical trial. Before the first subject is enrolled in the trial, all ethical and legal requirements must be met. All planned substantial changes (see §10, (1) of German GCP-Regulation) will be submitted for approval to EC and the competent authority in writing as protocol amendments.

11. Publications

11.1. Reports

Within one year of the completion of the trial, the competent authority and the ethics committee will be supplied with a summary of the final report on the clinical trial containing the principle results.

All reports to the sponsor will be written in English language. All clinical, analytical and statistical results will be presented in a final clinical trial report (CTR). The outline of this report will accord to the ICH Topic E3.

11.2. Publication

The final results of this study will be presented at scientific meetings and published in a peer reviewed journal. All publications on result of this study should be based on the scientific reports (see 11.1) and are the responsibility of the CI. The authorship will reflect the contributions of each collaborating centre. Any publication, abstract or presentation based on patients included in this study must be approved by the CI. First safety data will be published after completion of EOSf of the last patient enrolled in the clinical trial.

No publications on planned or unplanned interim analyses (e.g. safety analysis for DSMB or provisionally results on immunological efficacy before finalization of the scientific reports) are allowed.

12. Financing

This study is financed by the “Sonderfördermaßnahme COVID-19” of the ministry of science, research and art of the state Baden-Wuerttemberg, Germany.

13. Literature

1. Mo P, Xing Y, Xiao Y, et al. Clinical characteristics of refractory COVID-19 pneumonia in Wuhan, China. *Clin Infect Dis* 2020 doi: 10.1093/cid/ciaa270 [published Online First: 2020/03/17]
2. Ng OT, Marimuthu K, Chia PY, et al. SARS-CoV-2 Infection among Travelers Returning from Wuhan, China. *N Engl J Med* 2020 doi: 10.1056/NEJMc2003100 [published Online First: 2020/03/13]
3. Khan S, Siddique R, Shereen MA, et al. The emergence of a novel coronavirus (SARS-CoV-2), their biology and therapeutic options. *J Clin Microbiol* 2020 doi: 10.1128/JCM.00187-20 [published Online First: 2020/03/13]
4. Organization WH. Report of the WHO-China Joint Mission on Coronavirus Disease 2019. 2020
5. Seder RA, Darrah PA, Roederer M. T-cell quality in memory and protection: implications for vaccine design. *Nat Rev Immunol* 2008;8(4):247-58. doi: 10.1038/nri2274 [published Online First: 2008/03/08]
6. Khan N, Best D, Bruton R, et al. T cell recognition patterns of immunodominant cytomegalovirus antigens in primary and persistent infection. *J Immunol* 2007;178(7):4455-65. doi: 10.4049/jimmunol.178.7.4455 [published Online First: 2007/03/21]
7. Hill GR, Tey SK, Beagley L, et al. Successful immunotherapy of HCMV disease using virus-specific T cells expanded from an allogeneic stem cell transplant recipient. *Am J Transplant* 2010;10(1):173-9. doi: 10.1111/j.1600-6143.2009.02872.x [published Online First: 2009/11/19]
8. Feucht J, Joachim L, Lang P, et al. Adoptive T-cell transfer for refractory viral infections with cytomegalovirus, Epstein-Barr virus or adenovirus after allogeneic stem cell transplantation. *Klin Padiatr* 2013;225(3):164-9. doi: 10.1055/s-0033-1333749 [published Online First: 2013/05/24]
9. Hanajiri R, Sani GM, Hanley PJ, et al. Generation of Zika virus-specific T cells from seropositive and virus-naive donors for potential use as an autologous or "off-the-shelf" immunotherapeutic. *Cytotherapy* 2019;21(8):840-55. doi: 10.1016/j.jcyt.2019.06.008 [published Online First: 2019/07/08]
10. Wisskirchen K, Kah J, Malo A, et al. T cell receptor grafting allows virological control of Hepatitis B virus infection. *J Clin Invest* 2019;129(7):2932-45. doi: 10.1172/JCI120228 [published Online First: 2019/05/01]
11. Hanajiri R, Sani GM, Saunders D, et al. Generation of Norovirus-Specific T Cells From Human Donors With Extensive Cross-Reactivity to Variant Sequences: Implications for Immunotherapy. *J Infect Dis* 2020;221(4):578-88. doi: 10.1093/infdis/jiz491 [published Online First: 2019/09/29]
12. Channappanavar R, Fett C, Zhao J, et al. Virus-specific memory CD8 T cells provide substantial protection from lethal severe acute respiratory syndrome coronavirus infection. *J Virol* 2014;88(19):11034-44. doi: 10.1128/JVI.01505-14 [published Online First: 2014/07/25]
13. Channappanavar R, Zhao J, Perlman S. T cell-mediated immune response to respiratory coronaviruses. *Immunol Res* 2014;59(1-3):118-28. doi: 10.1007/s12026-014-8534-z [published Online First: 2014/05/23]
14. Zhao J, Zhao J, Mangalam AK, et al. Airway Memory CD4(+) T Cells Mediate Protective Immunity against Emerging Respiratory Coronaviruses. *Immunity* 2016;44(6):1379-91. doi: 10.1016/j.immuni.2016.05.006 [published Online First: 2016/06/12]
15. Zhao J, Zhao J, Perlman S. T cell responses are required for protection from clinical disease and for virus clearance in severe acute respiratory syndrome coronavirus-infected mice. *J Virol* 2010;84(18):9318-25. doi: 10.1128/JVI.01049-10 [published Online First: 2010/07/09]

16. Liu L, Wei Q, Lin Q, et al. Anti-spike IgG causes severe acute lung injury by skewing macrophage responses during acute SARS-CoV infection. *JCI Insight* 2019;4(4) doi: 10.1172/jci.insight.123158 [published Online First: 2019/03/05]
17. Tang F, Quan Y, Xin ZT, et al. Lack of peripheral memory B cell responses in recovered patients with severe acute respiratory syndrome: a six-year follow-up study. *J Immunol* 2011;186(12):7264-8. doi: 10.4049/jimmunol.0903490 [published Online First: 2011/05/18]
18. Liu WJ, Zhao M, Liu K, et al. T-cell immunity of SARS-CoV: Implications for vaccine development against MERS-CoV. *Antiviral Res* 2017;137:82-92. doi: 10.1016/j.antiviral.2016.11.006 [published Online First: 2016/11/15]
19. Grifoni A, Weiskopf D, Ramirez SI, et al. Targets of T Cell Responses to SARS-CoV-2 Coronavirus in Humans with COVID-19 Disease and Unexposed Individuals. *Cell* 2020 doi: 10.1016/j.cell.2020.05.015 [published Online First: 2020/05/31]
20. Braun J, Loyal L, Frentsch M, et al. Presence of SARS-CoV-2 reactive T cells in COVID-19 patients and healthy donors. *medRxiv* 2020:2020.04.17.20061440. doi: 10.1101/2020.04.17.20061440
21. Vali B, Tohn R, Cohen MJ, et al. Characterization of cross-reactive CD8+ T-cell recognition of HLA-A2-restricted HIV-Gag (SLYNTVATL) and HCV-NS5b (ALYDVVSKL) epitopes in individuals infected with human immunodeficiency and hepatitis C viruses. *J Virol* 2011;85(1):254-63. doi: 10.1128/JVI.01743-10 [published Online First: 2010/10/29]
22. Acierno PM, Newton DA, Brown EA, et al. Cross-reactivity between HLA-A2-restricted FLU-M1:58-66 and HIV p17 GAG:77-85 epitopes in HIV-infected and uninfected individuals. *J Transl Med* 2003;1(1):3. doi: 10.1186/1479-5876-1-3 [published Online First: 2003/10/07]
23. Clute SC, Watkin LB, Cornberg M, et al. Cross-reactive influenza virus-specific CD8+ T cells contribute to lymphoproliferation in Epstein-Barr virus-associated infectious mononucleosis. *J Clin Invest* 2005;115(12):3602-12. doi: 10.1172/JCI25078 [published Online First: 2005/11/26]
24. Nelde A, Bilich T, Heitmann JS, et al. SARS-CoV-2 T-cell epitopes define heterologous and COVID-19-induced T-cell recognition. In: Preprint, ed. Research Square, 2020.
25. Deres K, Schild H, Wiesmuller KH, et al. In vivo priming of virus-specific cytotoxic T lymphocytes with synthetic lipopeptide vaccine. *Nature* 1989;342(6249):561-4. doi: 10.1038/342561a0 [published Online First: 1989/11/30]
26. Falk K, Rotzschke O, Rammensee HG. Cellular peptide composition governed by major histocompatibility complex class I molecules. *Nature* 1990;348(6298):248-51. doi: 10.1038/348248a0 [published Online First: 1990/11/15]
27. Rammensee HG. Survival of the fitters. *Nature* 2002;419(6906):443-5. doi: 10.1038/419443a [published Online First: 2002/10/09]
28. Rammensee H, Bachmann J, Emmerich NP, et al. SYFPEITHI: database for MHC ligands and peptide motifs. *Immunogenetics* 1999;50(3-4):213-9. doi: 10.1007/s002510050595 [published Online First: 1999/12/22]
29. Bilich T, Nelde A, Bichmann L, et al. The HLA ligandome landscape of chronic myeloid leukemia delineates novel T-cell epitopes for immunotherapy. *Blood* 2019;133(6):550-65. doi: 10.1182/blood-2018-07-866830 [published Online First: 2018/12/12]
30. Lubke M, Spalt S, Kowalewski DJ, et al. Identification of HCMV-derived T cell epitopes in seropositive individuals through viral deletion models. *J Exp Med* 2020;217(3) doi: 10.1084/jem.20191164 [published Online First: 2019/12/24]
31. Berlin C, Kowalewski DJ, Schuster H, et al. Mapping the HLA ligandome landscape of acute myeloid leukemia: a targeted approach toward peptide-based immunotherapy. *Leukemia* 2015;29(3):647-59. doi: 10.1038/leu.2014.233 [published Online First: 2014/08/06]
32. Kowalewski DJ, Schuster H, Backert L, et al. HLA ligandome analysis identifies the underlying specificities of spontaneous antileukemia immune responses in chronic

lymphocytic leukemia (CLL). *Proc Natl Acad Sci U S A* 2015;112(2):E166-75. doi: 10.1073/pnas.1416389112

33. Nastke MD, Herrgen L, Walter S, et al. Major contribution of codominant CD8 and CD4 T cell epitopes to the human cytomegalovirus-specific T cell repertoire. *Cell Mol Life Sci* 2005;62(1):77-86. doi: 10.1007/s00018-004-4363-x [published Online First: 2004/12/25]
34. Icheva V, Kayser S, Wolff D, et al. Adoptive transfer of epstein-barr virus (EBV) nuclear antigen 1-specific t cells as treatment for EBV reactivation and lymphoproliferative disorders after allogeneic stem-cell transplantation. *J Clin Oncol* 2013;31(1):39-48. doi: 10.1200/JCO.2011.39.8495 [published Online First: 2012/11/22]
35. Hilf N, Kuttruff-Coqui S, Frenzel K, et al. Actively personalized vaccination trial for newly diagnosed glioblastoma. *Nature* 2019;565(7738):240-45. doi: 10.1038/s41586-018-0810-y [published Online First: 2018/12/21]
36. Baumgaertner P, Jandus C, Rivals JP, et al. Vaccination-induced functional competence of circulating human tumor-specific CD8 T-cells. *Int J Cancer* 2012;130(11):2607-17. doi: 10.1002/ijc.26297 [published Online First: 2011/07/29]
37. Freund J. The effect of paraffin oil and mycobacteria on antibody formation and sensitization; a review. *Am J Clin Pathol* 1951;21(7):645-56. doi: 10.1093/ajcp/21.7.645 [published Online First: 1951/07/01]
38. Rammensee HG, Wiesmuller KH, Chandran PA, et al. A new synthetic toll-like receptor 1/2 ligand is an efficient adjuvant for peptide vaccination in a human volunteer. *J Immunother Cancer* 2019;7(1):307. doi: 10.1186/s40425-019-0796-5 [published Online First: 2019/11/16]
39. Alam I, Goldeck D, Larbi A, et al. Aging affects the proportions of T and B cells in a group of elderly men in a developing country--a pilot study from Pakistan. *Age (Dordr)* 2013;35(5):1521-30. doi: 10.1007/s11357-012-9455-1 [published Online First: 2012/07/20]
40. Fulop T, Larbi A, Pawelec G. Human T cell aging and the impact of persistent viral infections. *Front Immunol* 2013;4:271. doi: 10.3389/fimmu.2013.00271 [published Online First: 2013/09/26]
41. Lambkin R, Novelli P, Oxford J, et al. Human genetics and responses to influenza vaccination: clinical implications. *Am J Pharmacogenomics* 2004;4(5):293-8. doi: 10.2165/00129785-200404050-00002 [published Online First: 2004/10/07]
42. Molano A, Park SH, Chiu YH, et al. Cutting edge: the IgG response to the circumsporozoite protein is MHC class II-dependent and CD1d-independent: exploring the role of GPIs in NK T cell activation and antimalarial responses. *J Immunol* 2000;164(10):5005-9. doi: 10.4049/jimmunol.164.10.5005 [published Online First: 2000/05/09]
43. Oliveira GA, Kumar KA, Calvo-Calle JM, et al. Class II-restricted protective immunity induced by malaria sporozoites. *Infect Immun* 2008;76(3):1200-6. doi: 10.1128/IAI.00566-07 [published Online First: 2007/12/28]
44. Xu R, Johnson AJ, Liggitt D, et al. Cellular and humoral immunity against vaccinia virus infection of mice. *J Immunol* 2004;172(10):6265-71. doi: 10.4049/jimmunol.172.10.6265 [published Online First: 2004/05/07]
45. Sette A, Moutaftsi M, Moyron-Quiroz J, et al. Selective CD4+ T cell help for antibody responses to a large viral pathogen: deterministic linkage of specificities. *Immunity* 2008;28(6):847-58. doi: 10.1016/j.immuni.2008.04.018 [published Online First: 2008/06/14]
46. Shedlock DJ, Shen H. Requirement for CD4 T cell help in generating functional CD8 T cell memory. *Science* 2003;300(5617):337-9. doi: 10.1126/science.1082305 [published Online First: 2003/04/12]
47. Carvalho LH, Sano G, Hafalla JC, et al. IL-4-secreting CD4+ T cells are crucial to the development of CD8+ T-cell responses against malaria liver stages. *Nature medicine* 2002;8(2):166-70. doi: 10.1038/nm0202-166 [published Online First: 2002/02/01]

48. Kemball CC, Pack CD, Guay HM, et al. The antiviral CD8+ T cell response is differentially dependent on CD4+ T cell help over the course of persistent infection. *J Immunol* 2007;179(2):1113-21. doi: 10.4049/jimmunol.179.2.1113 [published Online First: 2007/07/10]
49. Marzo AL, Vezys V, Klonowski KD, et al. Fully functional memory CD8 T cells in the absence of CD4 T cells. *J Immunol* 2004;173(2):969-75. doi: 10.4049/jimmunol.173.2.969 [published Online First: 2004/07/09]
50. van de Berg PJ, van Leeuwen EM, ten Berge IJ, et al. Cytotoxic human CD4(+) T cells. *Curr Opin Immunol* 2008;20(3):339-43. doi: 10.1016/j.coi.2008.03.007 [published Online First: 2008/04/29]
51. Johnson AJ, Chu CF, Milligan GN. Effector CD4+ T-cell involvement in clearance of infectious herpes simplex virus type 1 from sensory ganglia and spinal cords. *J Virol* 2008;82(19):9678-88. doi: 10.1128/JVI.01159-08 [published Online First: 2008/08/01]
52. Elyaman W, Kivisakk P, Reddy J, et al. Distinct functions of autoreactive memory and effector CD4+ T cells in experimental autoimmune encephalomyelitis. *Am J Pathol* 2008;173(2):411-22. doi: 10.2353/ajpath.2008.080142 [published Online First: 2008/06/28]
53. Tsuji M, Romero P, Nussenzweig RS, et al. CD4+ cytolytic T cell clone confers protection against murine malaria. *J Exp Med* 1990;172(5):1353-7. doi: 10.1084/jem.172.5.1353 [published Online First: 1990/11/01]
54. Harrington LE, Mangan PR, Weaver CT. Expanding the effector CD4 T-cell repertoire: the Th17 lineage. *Curr Opin Immunol* 2006;18(3):349-56. doi: 10.1016/j.coi.2006.03.017 [published Online First: 2006/04/18]
55. Long QX, Tang XJ, Shi QL, et al. Clinical and immunological assessment of asymptomatic SARS-CoV-2 infections. *Nature medicine* 2020;26(8):1200-04. doi: 10.1038/s41591-020-0965-6 [published Online First: 2020/06/20]
56. Kreer C, Zehner M, Weber T, et al. Longitudinal Isolation of Potent Near-Germline SARS-CoV-2-Neutralizing Antibodies from COVID-19 Patients. *Cell* 2020 doi: 10.1016/j.cell.2020.06.044 [published Online First: 2020/07/17]
57. Steere AC, Sikand VK, Meurice F, et al. Vaccination against Lyme disease with recombinant Borrelia burgdorferi outer-surface lipoprotein A with adjuvant. Lyme Disease Vaccine Study Group. *N Engl J Med* 1998;339(4):209-15. doi: 10.1056/NEJM199807233390401 [published Online First: 1998/07/23]
58. Sigal LH, Zahradnik JM, Lavin P, et al. A vaccine consisting of recombinant Borrelia burgdorferi outer-surface protein A to prevent Lyme disease. Recombinant Outer-Surface Protein A Lyme Disease Vaccine Study Consortium. *N Engl J Med* 1998;339(4):216-22. doi: 10.1056/NEJM199807233390402 [published Online First: 1998/07/23]
59. Opie EL, Freund J. An Experimental Study of Protective Inoculation with Heat Killed Tubercle Bacilli. *J Exp Med* 1937;66(6):761-88. doi: 10.1084/jem.66.6.761 [published Online First: 1937/11/30]
60. Jensen FC, Savary JR, Diveley JP, et al. Adjuvant activity of incomplete Freund's adjuvant. *Adv Drug Deliv Rev* 1998;32(3):173-86. doi: 10.1016/s0169-409x(98)00009-x [published Online First: 2000/06/06]
61. Rammensee HG, Stevanovic S, Gouttefangeas C, et al. Designing a therapeutic SARS-CoV-2 T-cell-inducing vaccine for high-risk patient groups. *Research Square [preprint]* 2020 doi:

10.21203/rs.3.rs-27316/v1
62. Kran AM, Sorensen B, Nyhus J, et al. HLA- and dose-dependent immunogenicity of a peptide-based HIV-1 immunotherapy candidate (Vacc-4x). *AIDS* 2004;18(14):1875-83.
63. Feyerabend S, Stevanovic S, Gouttefangeas C, et al. Novel multi-peptide vaccination in Hla-A2+ hormone sensitive patients with biochemical relapse of prostate cancer.

Prostate 2009;69(9):917-27. doi: 10.1002/pros.20941 [published Online First: 2009/03/10]

64. Sato Y, Shomura H, Maeda Y, et al. Immunological evaluation of peptide vaccination for patients with gastric cancer based on pre-existing cellular response to peptide. *Cancer Sci* 2003;94(9):802-8.
65. Noguchi M, Kobayashi K, Suetsugu N, et al. Induction of cellular and humoral immune responses to tumor cells and peptides in HLA-A24 positive hormone-refractory prostate cancer patients by peptide vaccination. *Prostate* 2003;57(1):80-92. doi: 10.1002/pros.10276
66. Atsmon J, Kate-Ilovitz E, Shaikevich D, et al. Safety and immunogenicity of multimeric-001--a novel universal influenza vaccine. *J Clin Immunol* 2012;32(3):595-603. doi: 10.1007/s10875-011-9632-5 [published Online First: 2012/02/10]
67. Salk JE, Bailey ML, Laurent AM. The use of adjuvants in studies on influenza immunization. II. Increased antibody formation in human subjects inoculated with influenza virus vaccine in a water in-oil emulsion. *Am J Hyg* 1952;55(3):439-56. doi: 10.1093/oxfordjournals.aje.a119534 [published Online First: 1952/05/01]
68. Meiklejohn G. Adjuvant influenza adenovirus vaccine. *JAMA* 1962;179:594-7. doi: 10.1001/jama.1962.03050080006002 [published Online First: 1962/02/24]
69. Stern LJ, Calvo-Calle JM. HLA-DR: molecular insights and vaccine design. *Curr Pharm Des* 2009;15(28):3249-61. doi: 10.2174/138161209789105171 [published Online First: 2009/10/29]
70. Herrington DA, Clyde DF, Losonsky G, et al. Safety and immunogenicity in man of a synthetic peptide malaria vaccine against Plasmodium falciparum sporozoites. *Nature* 1987;328(6127):257-9. doi: 10.1038/328257a0 [published Online First: 1987/07/16]
71. Weihrauch MR, Ansen S, Jurkiewicz E, et al. Phase I/II combined chemoimmunotherapy with carcinoembryonic antigen-derived HLA-A2-restricted CAP-1 peptide and irinotecan, 5-fluorouracil, and leucovorin in patients with primary metastatic colorectal cancer. *Clinical cancer research : an official journal of the American Association for Cancer Research* 2005;11(16):5993-6001. doi: 10.1158/1078-0432.CCR-05-0018
72. Peoples GE, Gurney JM, Hueman MT, et al. Clinical trial results of a HER2/neu (E75) vaccine to prevent recurrence in high-risk breast cancer patients. *J Clin Oncol* 2005;23(30):7536-45. doi: 10.1200/JCO.2005.03.047
73. Walter S, Weinschenk T, Stenzl A, et al. Multipeptide immune response to cancer vaccine IMA901 after single-dose cyclophosphamide associates with longer patient survival. *Nature medicine* 2012 doi: 10.1038/nm.2883 [published Online First: 2012/07/31]
74. Mailander V, Scheibenbogen C, Thiel E, et al. Complete remission in a patient with recurrent acute myeloid leukemia induced by vaccination with WT1 peptide in the absence of hematological or renal toxicity. *Leukemia* 2004;18(1):165-6. doi: 10.1038/sj.leu.2403186
75. Oka Y, Tsuboi A, Taguchi T, et al. Induction of WT1 (Wilms' tumor gene)-specific cytotoxic T lymphocytes by WT1 peptide vaccine and the resultant cancer regression. *Proc Natl Acad Sci U S A* 2004;101(38):13885-90. doi: 10.1073/pnas.0405884101
76. Van Tendeloo VF, Van de Velde A, Van Driessche A, et al. Induction of complete and molecular remissions in acute myeloid leukemia by Wilms' tumor 1 antigen-targeted dendritic cell vaccination. *Proc Natl Acad Sci U S A* 2010;107(31):13824-9. doi: 10.1073/pnas.1008051107
77. Schmitt M, Schmitt A, Rojewski MT, et al. RHAMM-R3 peptide vaccination in patients with acute myeloid leukemia, myelodysplastic syndrome, and multiple myeloma elicits immunologic and clinical responses. *Blood* 2008;111(3):1357-65. doi: 10.1182/blood-2007-07-099366 [published Online First: 2007/11/06]
78. Schwartzentruber DJ, Lawson DH, Richards JM, et al. gp100 peptide vaccine and interleukin-2 in patients with advanced melanoma. *N Engl J Med* 2011;364(22):2119-27. doi: 10.1056/NEJMoa1012863

79. Mittendorf EA, Clifton GT, Holmes JP, et al. Clinical trial results of the HER-2/neu (E75) vaccine to prevent breast cancer recurrence in high-risk patients: from US Military Cancer Institute Clinical Trials Group Study I-01 and I-02. *Cancer* 2012;118(10):2594-602. doi: 10.1002/cncr.26574 [published Online First: 2011/10/13]
80. Weinschenk T, Gouttefangeas C, Schirle M, et al. Integrated functional genomics approach for the design of patient-individual antitumor vaccines. *Cancer Res* 2002;62(20):5818-27. [published Online First: 2002/10/18]
81. Honda-Okubo Y, Barnard D, Ong CH, et al. Severe acute respiratory syndrome-associated coronavirus vaccines formulated with delta inulin adjuvants provide enhanced protection while ameliorating lung eosinophilic immunopathology. *J Virol* 2015;89(6):2995-3007. doi: 10.1128/JVI.02980-14 [published Online First: 2014/12/19]
82. Graham BS. Rapid COVID-19 vaccine development. *Science* 2020 doi: 10.1126/science.abb8923 [published Online First: 2020/05/10]
83. van Doorn E, Liu H, Huckriede A, et al. Safety and tolerability evaluation of the use of Montanide ISA51 as vaccine adjuvant: A systematic review. *Hum Vaccin Immunother* 2016;12(1):159-69. doi: 10.1080/21645515.2015.1071455
84. Van den Heuvel MM, Burgers SA, van Zandwijk N. Immunotherapy in non-small-cell lung carcinoma: from inflammation to vaccination. *Clinical lung cancer* 2009;10(2):99-105. doi: 10.3816/CLC.2009.n.012
85. Wu Y, Ellis RD, Shaffer D, et al. Phase 1 trial of malaria transmission blocking vaccine candidates Pfs25 and Pvs25 formulated with montanide ISA 51. *PLoS One* 2008;3(7):e2636. doi: 10.1371/journal.pone.0002636
86. Carr A, Rodriguez E, Arango Mdel C, et al. Immunotherapy of advanced breast cancer with a heterophilic ganglioside (NeuGcGM3) cancer vaccine. *J Clin Oncol* 2003;21(6):1015-21. doi: 10.1200/JCO.2003.02.124
87. Yamaue H, Tsunoda T, Tani M, et al. Randomized phase II/III clinical trial of elpamotide for patients with advanced pancreatic cancer: PEGASUS-PC Study. *Cancer Sci* 2015;106(7):883-90. doi: 10.1111/cas.12674 [published Online First: 2015/04/14]
88. Widenmeyer M, Griesemann H, Stevanovic S, et al. Promiscuous survivin peptide induces robust CD4+ T-cell responses in the majority of vaccinated cancer patients. *Int J Cancer* 2012;131(1):140-9. doi: 10.1002/ijc.26365 [published Online First: 2011/08/23]
89. Britten CM, Gouttefangeas C, Welters MJ, et al. The CIMT-monitoring panel: a two-step approach to harmonize the enumeration of antigen-specific CD8+ T lymphocytes by structural and functional assays. *Cancer Immunol Immunother* 2008;57(3):289-302. doi: 10.1007/s00262-007-0378-0 [published Online First: 2007/08/28]
90. Neumann A, Horzer H, Hillen N, et al. Identification of HLA ligands and T-cell epitopes for immunotherapy of lung cancer. *Cancer Immunol Immunother* 2013;62(9):1485-97. doi: 10.1007/s00262-013-1454-2 [published Online First: 2013/07/03]
91. Tavares Da Silva F, De Keyser F, Lambert PH, et al. Optimal approaches to data collection and analysis of potential immune mediated disorders in clinical trials of new vaccines. *Vaccine* 2013;31(14):1870-6. doi: 10.1016/j.vaccine.2013.01.042 [published Online First: 2013/02/09]

Regulatory References

Medicinal Products Act (Arzneimittelgesetz), published on 12 December 2005 (Federal Law Gazette [BGBl.] Part I p. 3394), last amended by Article 3 of the Law of 18 July 2017 (BGBl. I p. 2757)

Ordinance on the implementation of Good Clinical Practice in the conduct of clinical trials on medicinal products for use in humans (GCP Ordinance - GCP-V), published on 09 August 2004 (Federal Law Gazette (BGBl.) I p. 2081), last amended by Article 8 of the Law of 19. Oktober 2012 (BGBl. I S. 2192)

REGULATION (EU) No 536/2014 OF THE EUROPEAN PARLIAMENT AND OF THE COUNCIL of 16 April 2014 on clinical trials on medicinal products for human use, and repealing Directive 2001/20/EC

Medical Devices Act (Medizinproduktegesetz), published on 07.August 2002 (Federal Law Gazette (BGBl.) I p. 3146), last amended by Article 7 of the Law of 18 July 2017 (BGBl. I p. 2757)

ICH Topic E3, Note for Guidance on Structure and Content of Clinical Study Reports (CPMP/ICH/137/95), July 1996

ICH Topic E 6 (R2), Note for Guidance on Good Clinical Practice (CPMP/ICH/135/95), December 2016

ICH Topic E 8, Note for Guidance on General Considerations for Clinical Trials (CPMP/ICH/291/95), March 1998

Clinical Trial Facilitation Group, Recommendations related to contraception and pregnancy testing in clinical trials, 15.09.2014)

EMEA-Guideline On Data Monitoring Committees (EMEA/CHMP/EWP/5872/03 Corr), January 2006

REGULATION (EU) 2016/679 OF THE EUROPEAN PARLIAMENT AND OF THE COUNCIL of 27 April 2016 on the protection of natural persons with regard to the processing of personal data and on the free movement of such data, and repealing Directive 95/46/EC (General Data Protection Regulation)

14. Appendix

14.1. Common Terminology Criteria for Adverse Events (CTCAE) Version

https://ctep.cancer.gov/protocoldevelopment/electronic_applications/docs/CTCAE_v5_Quick_Reference_5x7.pdf

14.2. List of central laboratories

[redacted]

[redacted]

[redacted]

14.3. Volunteer diary

Studie	P-pVAC-SARS-CoV-2
Probanden-ID *(vom Arzt auszufüllen)*:	[_ _ _ _] - [_ _ _]
Datum der Impfung:	[_ _] [_ _] [20_ _]

1. Richtlinien

Füllen Sie Ihr Tagebuch (**täglich**) mit Ankreuzen und gegebenenfalls weiteren Ergänzungen aus. Falls Sie eine Frage nicht beantworten können, streichen Sie diese bitte durch. Falls Sie Fragen mit „Ja“ beantworten, füllen Sie bitte weitere Angaben aus. Bei Rückfragen oder starken Beschwerden, melden Sie sich bitte an Ihrem Prüfzentrum.

2. Tag der Impfung (d1) [_ _] [_ _] [20_ _]

		Ja	Nein	Weitere Angaben
1.	Haben Sie Schmerzen an der Impfstelle?	☐	☐	______________________
2.	Ist die Impfstelle gerötet oder geschwollen?	☐	☐	______________________
3.	Haben Sie Fieber, Schüttelfrost oder Gliederschmerzen?	☐	☐	______________________
4.	Haben Sie Kopfschmerzen, Müdigkeit oder Übelkeit?	☐	☐	______________________
5.	Haben Sie andere Beschwerden?	☐	☐	______________________

3. Tag 2 nach der Impfung (d2) [_ _] [_ _] [20_ _]

		Ja	Nein	Weitere Angaben
1.	Haben Sie Schmerzen an der Impfstelle?	☐	☐	______________________
2.	Ist die Impfstelle gerötet oder geschwollen?	☐	☐	______________________
3.	Haben Sie Fieber, Schüttelfrost oder Gliederschmerzen?	☐	☐	______________________
4.	Haben Sie Kopfschmerzen, Müdigkeit oder Übelkeit?	☐	☐	______________________

		Ja	Nein	Weitere Angaben
5.	Haben Sie andere Beschwerden?	☐	☐	______________

4. Tag 3 nach der Impfung (d3) [_ _] [_ _] [20_ _]

		Ja	Nein	Weitere Angaben
1.	Haben Sie Schmerzen an der Impfstelle?	☐	☐	______________
2.	Ist die Impfstelle gerötet oder geschwollen?	☐	☐	______________
3.	Haben Sie Fieber, Schüttelfrost oder Gliederschmerzen?	☐	☐	______________
4.	Haben Sie Kopfschmerzen, Müdigkeit oder Übelkeit?	☐	☐	______________
5.	Haben Sie andere Beschwerden?	☐	☐	______________

5. Tag 4 nach der Impfung (d4) [_ _] [_ _] [20_ _]

		Ja	Nein	Weitere Angaben
1.	Haben Sie Schmerzen an der Impfstelle?	☐	☐	______________
2.	Ist die Impfstelle gerötet oder geschwollen?	☐	☐	______________
3.	Haben Sie Fieber, Schüttelfrost oder Gliederschmerzen?	☐	☐	______________
4.	Haben Sie Kopfschmerzen, Müdigkeit oder Übelkeit?	☐	☐	______________
5.	Haben Sie andere Beschwerden?	☐	☐	______________

6. Tag 5 nach der Impfung (d5) [_ _] [_ _] [20_ _]

		Ja	Nein	Weitere Angaben
1.	Haben Sie Schmerzen an der Impfstelle?	☐	☐	______________
2.	Ist die Impfstelle gerötet oder geschwollen?	☐	☐	______________

3.	Haben Sie Fieber, Schüttelfrost oder Gliederschmerzen?	☐	☐	______________________
4.	Haben Sie Kopfschmerzen, Müdigkeit oder Übelkeit?	☐	☐	______________________
5.	Haben Sie andere Beschwerden?	☐	☐	______________________

7. Tag 6 nach der Impfung (d6) [_ _] [_ _] [20_ _]

		Ja	Nein	Weitere Angaben
1.	Haben Sie Schmerzen an der Impfstelle?	☐	☐	______________________
2.	Ist die Impfstelle gerötet oder geschwollen?	☐	☐	______________________
3.	Haben Sie Fieber, Schüttelfrost oder Gliederschmerzen?	☐	☐	______________________
4.	Haben Sie Kopfschmerzen, Müdigkeit oder Übelkeit?	☐	☐	______________________
5.	Haben Sie andere Beschwerden?	☐	☐	______________________

8. Tag 7 nach der Impfung (d7) [_ _] [_ _] [20_ _]

		Ja	Nein	Weitere Angaben
1.	Haben Sie Schmerzen an der Impfstelle?	☐	☐	______________________
2.	Ist die Impfstelle gerötet oder geschwollen?	☐	☐	______________________
3.	Haben Sie Fieber, Schüttelfrost oder Gliederschmerzen?	☐	☐	______________________
4.	Haben Sie Kopfschmerzen, Müdigkeit oder Übelkeit?	☐	☐	______________________
5.	Haben Sie andere Beschwerden?	☐	☐	______________________

9. Tag 8 nach der Impfung (d8) [_ _] [_ _] [20_ _]

		Ja	Nein	Weitere Angaben

1.	Haben Sie Schmerzen an der Impfstelle?	☐	☐	______________
2.	Ist die Impfstelle gerötet oder geschwollen?	☐	☐	______________
3.	Haben Sie Fieber, Schüttelfrost oder Gliederschmerzen?	☐	☐	______________
4.	Haben Sie Kopfschmerzen, Müdigkeit oder Übelkeit?	☐	☐	______________
5.	Haben Sie andere Beschwerden?	☐	☐	______________

10. Tag 9 nach der Impfung (d9) [_ _] [_ _] [20_ _]

		Ja	Nein	Weitere Angaben
1.	Haben Sie Schmerzen an der Impfstelle?	☐	☐	______________
2.	Ist die Impfstelle gerötet oder geschwollen?	☐	☐	______________
3.	Haben Sie Fieber, Schüttelfrost oder Gliederschmerzen?	☐	☐	______________
4.	Haben Sie Kopfschmerzen, Müdigkeit oder Übelkeit?	☐	☐	______________
5.	Haben Sie andere Beschwerden?	☐	☐	______________

11. Tag 10 nach der Impfung (d10) [_ _] [_ _] [20_ _]

		Ja	Nein	Weitere Angaben
1.	Haben Sie Schmerzen an der Impfstelle?	☐	☐	______________
2.	Ist die Impfstelle gerötet oder geschwollen?	☐	☐	______________
3.	Haben Sie Fieber, Schüttelfrost oder Gliederschmerzen?	☐	☐	______________
4.	Haben Sie Kopfschmerzen, Müdigkeit oder Übelkeit?	☐	☐	______________

5.	Haben Sie andere Beschwerden?	☐	☐	________________

12. Tag 11 nach der Impfung (d11) [_ _] [_ _] [20_ _]

		Ja	Nein	Weitere Angaben
1.	Haben Sie Schmerzen an der Impfstelle?	☐	☐	________________
2.	Ist die Impfstelle gerötet oder geschwollen?	☐	☐	________________
3.	Haben Sie Fieber, Schüttelfrost oder Gliederschmerzen?	☐	☐	________________
4.	Haben Sie Kopfschmerzen, Müdigkeit oder Übelkeit?	☐	☐	________________
5.	Haben Sie andere Beschwerden?	☐	☐	________________

13. Tag 12 nach der Impfung (d12) [_ _] [_ _] [20_ _]

		Ja	Nein	Weitere Angaben
1.	Haben Sie Schmerzen an der Impfstelle?	☐	☐	________________
2.	Ist die Impfstelle gerötet oder geschwollen?	☐	☐	________________
3.	Haben Sie Fieber, Schüttelfrost oder Gliederschmerzen?	☐	☐	________________
4.	Haben Sie Kopfschmerzen, Müdigkeit oder Übelkeit?	☐	☐	________________
5.	Haben Sie andere Beschwerden?	☐	☐	________________

14. Tag 13 nach der Impfung (d13) [_ _] [_ _] [20_ _]

		Ja	Nein	Weitere Angaben
1.	Haben Sie Schmerzen an der Impfstelle?	☐	☐	________________
2.	Ist die Impfstelle gerötet oder geschwollen?	☐	☐	________________

3.	Haben Sie Fieber, Schüttelfrost oder Gliederschmerzen?	☐	☐	______
4.	Haben Sie Kopfschmerzen, Müdigkeit oder Übelkeit?	☐	☐	______
5.	Haben Sie andere Beschwerden?	☐	☐	______

15. Tag 14 nach der Impfung (d14) [_ _] [_ _] [20_ _]

		Ja	Nein	Weitere Angaben
1.	Haben Sie Schmerzen an der Impfstelle?	☐	☐	______
2.	Ist die Impfstelle gerötet oder geschwollen?	☐	☐	______
3.	Haben Sie Fieber, Schüttelfrost oder Gliederschmerzen?	☐	☐	______
4.	Haben Sie Kopfschmerzen, Müdigkeit oder Übelkeit?	☐	☐	______
5.	Haben Sie andere Beschwerden?	☐	☐	______

16. Tag 15 nach der Impfung (d15) [_ _] [_ _] [20_ _]

		Ja	Nein	Weitere Angaben
1.	Haben Sie Schmerzen an der Impfstelle?	☐	☐	______
2.	Ist die Impfstelle gerötet oder geschwollen?	☐	☐	______
3.	Haben Sie Fieber, Schüttelfrost oder Gliederschmerzen?	☐	☐	______
4.	Haben Sie Kopfschmerzen, Müdigkeit oder Übelkeit?	☐	☐	______
5.	Haben Sie andere Beschwerden?	☐	☐	______

17. Tag 16 nach der Impfung (d16) [_ _] [_ _] [20_ _]

		Ja	Nein	Weitere Angaben

		Ja	Nein	Weitere Angaben
1.	Haben Sie Schmerzen an der Impfstelle?	☐	☐	______________
2.	Ist die Impfstelle gerötet oder geschwollen?	☐	☐	______________
3.	Haben Sie Fieber, Schüttelfrost oder Gliederschmerzen?	☐	☐	______________
4.	Haben Sie Kopfschmerzen, Müdigkeit oder Übelkeit?	☐	☐	______________
5.	Haben Sie andere Beschwerden?	☐	☐	______________

18. Tag 17 nach der Impfung (d17) [_ _] [_ _] [20_ _]

		Ja	Nein	Weitere Angaben
1.	Haben Sie Schmerzen an der Impfstelle?	☐	☐	______________
2.	Ist die Impfstelle gerötet oder geschwollen?	☐	☐	______________
3.	Haben Sie Fieber, Schüttelfrost oder Gliederschmerzen?	☐	☐	______________
4.	Haben Sie Kopfschmerzen, Müdigkeit oder Übelkeit?	☐	☐	______________
5.	Haben Sie andere Beschwerden?	☐	☐	______________

19. Tag 18 nach der Impfung (d18) [_ _] [_ _] [20_ _]

		Ja	Nein	Weitere Angaben
1.	Haben Sie Schmerzen an der Impfstelle?	☐	☐	______________
2.	Ist die Impfstelle gerötet oder geschwollen?	☐	☐	______________
3.	Haben Sie Fieber, Schüttelfrost oder Gliederschmerzen?	☐	☐	______________
4.	Haben Sie Kopfschmerzen, Müdigkeit oder Übelkeit?	☐	☐	______________

		Ja	Nein	Weitere Angaben
5.	Haben Sie andere Beschwerden?	☐	☐	______

20. Tag 19 nach der Impfung (d19) [_ _] [_ _] [20_ _]

		Ja	Nein	Weitere Angaben
1.	Haben Sie Schmerzen an der Impfstelle?	☐	☐	______
2.	Ist die Impfstelle gerötet oder geschwollen?	☐	☐	______
3.	Haben Sie Fieber, Schüttelfrost oder Gliederschmerzen?	☐	☐	______
4.	Haben Sie Kopfschmerzen, Müdigkeit oder Übelkeit?	☐	☐	______
5.	Haben Sie andere Beschwerden?	☐	☐	______

21. Tag 20 nach der Impfung (d20) [_ _] [_ _] [20_ _]

		Ja	Nein	Weitere Angaben
1.	Haben Sie Schmerzen an der Impfstelle?	☐	☐	______
2.	Ist die Impfstelle gerötet oder geschwollen?	☐	☐	______
3.	Haben Sie Fieber, Schüttelfrost oder Gliederschmerzen?	☐	☐	______
4.	Haben Sie Kopfschmerzen, Müdigkeit oder Übelkeit?	☐	☐	______
5.	Haben Sie andere Beschwerden?	☐	☐	______

22. Tag 21 nach der Impfung (d21) [_ _] [_ _] [20_ _]

		Ja	Nein	Weitere Angaben
1.	Haben Sie Schmerzen an der Impfstelle?	☐	☐	______
2.	Ist die Impfstelle gerötet oder geschwollen?	☐	☐	______

		Ja	Nein	Weitere Angaben
3.	Haben Sie Fieber, Schüttelfrost oder Gliederschmerzen?	☐	☐	______
4.	Haben Sie Kopfschmerzen, Müdigkeit oder Übelkeit?	☐	☐	______
5.	Haben Sie andere Beschwerden?	☐	☐	______

23. Tag 22 nach der Impfung (d22) [_ _] [_ _] [20_ _]

		Ja	Nein	Weitere Angaben
1.	Haben Sie Schmerzen an der Impfstelle?	☐	☐	______
2.	Ist die Impfstelle gerötet oder geschwollen?	☐	☐	______
3.	Haben Sie Fieber, Schüttelfrost oder Gliederschmerzen?	☐	☐	______
4.	Haben Sie Kopfschmerzen, Müdigkeit oder Übelkeit?	☐	☐	______
5.	Haben Sie andere Beschwerden?	☐	☐	______

24. Tag 23 nach der Impfung (d23) [_ _] [_ _] [20_ _]

		Ja	Nein	Weitere Angaben
1.	Haben Sie Schmerzen an der Impfstelle?	☐	☐	______
2.	Ist die Impfstelle gerötet oder geschwollen?	☐	☐	______
3.	Haben Sie Fieber, Schüttelfrost oder Gliederschmerzen?	☐	☐	______
4.	Haben Sie Kopfschmerzen, Müdigkeit oder Übelkeit?	☐	☐	______
5.	Haben Sie andere Beschwerden?	☐	☐	______

25. Tag 24 nach der Impfung (d24) [_ _] [_ _] [20_ _]

Ja Nein Weitere Angaben

1.	Haben Sie Schmerzen an der Impfstelle?	☐	☐	______
2.	Ist die Impfstelle gerötet oder geschwollen?	☐	☐	______
3.	Haben Sie Fieber, Schüttelfrost oder Gliederschmerzen?	☐	☐	______
4.	Haben Sie Kopfschmerzen, Müdigkeit oder Übelkeit?	☐	☐	______
5.	Haben Sie andere Beschwerden?	☐	☐	______

26. Tag 25 nach der Impfung (d25) [_ _] [_ _] [20_ _]

		Ja	Nein	Weitere Angaben
1.	Haben Sie Schmerzen an der Impfstelle?	☐	☐	______
2.	Ist die Impfstelle gerötet oder geschwollen?	☐	☐	______
3.	Haben Sie Fieber, Schüttelfrost oder Gliederschmerzen?	☐	☐	______
4.	Haben Sie Kopfschmerzen, Müdigkeit oder Übelkeit?	☐	☐	______
5.	Haben Sie andere Beschwerden?	☐	☐	______

27. Tag 26 nach der Impfung (d26) [_ _] [_ _] [20_ _]

		Ja	Nein	Weitere Angaben
1.	Haben Sie Schmerzen an der Impfstelle?	☐	☐	______
2.	Ist die Impfstelle gerötet oder geschwollen?	☐	☐	______
3.	Haben Sie Fieber, Schüttelfrost oder Gliederschmerzen?	☐	☐	______
4.	Haben Sie Kopfschmerzen, Müdigkeit oder Übelkeit?	☐	☐	______

		Ja	Nein	Weitere Angaben
5.	Haben Sie andere Beschwerden?	☐	☐	______________________

28. Tag 27 nach der Impfung (d27) [_ _] [_ _] [20_ _]

		Ja	Nein	Weitere Angaben
1.	Haben Sie Schmerzen an der Impfstelle?	☐	☐	______________________
2.	Ist die Impfstelle gerötet oder geschwollen?	☐	☐	______________________
3.	Haben Sie Fieber, Schüttelfrost oder Gliederschmerzen?	☐	☐	______________________
4.	Haben Sie Kopfschmerzen, Müdigkeit oder Übelkeit?	☐	☐	______________________
5.	Haben Sie andere Beschwerden?	☐	☐	______________________

14.4. Volunteer card

[redacted]

Studienkarte

Patientenname ____

Nimmt an der **P-pVac-SARS-CoV-2 Studie** zur Evaluation eines SARS-CoV-2 Impfstoff teil und wurde einmalig mit dem Impfstoff behandelt.

Bitte kontaktieren Sie im Notfall:

____

____

Bitte tragen Sie diese Notfallkarte immer bei sich

14.5. Intensity of solicited and unsolicited local and systemic adverse events

Local solicited AEs	CTCAE Term	Grade 0<br>Normal	Grade 1<br>Mild	Grade 2<br>Moderate	Grade 3<br>Severe	Grade 4<br>Potentially life-threatening
**Erythema**	Injection site reaction	< 25 mm	25-50mm<br>Tenderness with or without associated symptoms (e.g., warmth, erythema, itching)	51-100mm<br>Pain; lipodystrophy; edema; phlebitis	> 100mm<br>Ulceration or necrosis; severe tissue damage; operative intervention indicated	Life-threatening consequences; urgent intervention indicated
**Swelling**		< 25 mm	25-50 mm and does not interfere with activity	> 50 mm or interferes with activity	Prevents daily activity	Necrosis
**Pain**	Injection site reaction	None	Tenderness with or without associated symptoms (e.g., warmth, erythema, itching)<br><br>Does not interfere with activity	Pain; lipodystrophy; edema; phlebitis<br><br>Interferes with activity	Ulceration or necrosis; severe tissue damage; operative intervention indicated<br><br>Prevents daily activity	Life-threatening consequences; urgent intervention indicated<br>Emergency room visit or hospitalization

[redacted]

Systemic solicited AEs	CTCAE Term	Grade 0	Grade 1	Grade 2	Grade 3	Grade 4
**Fever**		None	38.0° - 39.0°C	≥ 39.0° - 40.0°C	≥ 40.0°C for ≤ 24 hours	≥ 40.0°C for ≥ 24 hours
**Chills**		None	Mild sensation of cold; shivering; chattering of teeth	Moderate tremor of the entire body; narcotics indicated	Severe or prolonged, not responsive to narcotics	-
**Myalgia** (described to the subject as generalized muscle ches)		None	Mild pain	Moderate pain; limiting instrumental ADL	Severe pain; limiting self care ADL	-
**Arthralgie** (described to the subject as generalized joint aches)			Mild pain	Moderate pain; limiting instrumental ADL	Severe pain; limiting self care ADL	-
**Fatigue**			Fatigue relieved by rest	Fatigue not relieved by rest; limiting instrumental ADL	Fatigue not relieved by rest, limiting self care ADL	-
**Headache**		None	Mild pain	Moderate pain; limiting instrumental ADL	Severe pain; limiting self care ADL	-
**Gastrointestinal symptoms** (nausea, vomiting, abdominal pain, and/or diarrhea)	nausea	None	Loss of appetite without alteration in eating habits	Oral intake decreased without significant weight loss, dehydration or malnutrition	Inadequate oral caloric or fluid intake; tube feeding, TPN, or hospitalization indicated	-
	vomiting	None	Intervention not indicated	Outpatient IV hydration; medical intervention indicated	Tube feeding, TPN, or hospitalization indicated	Life-threatening consequences
	abdominal pain	None	Mild pain	Moderate pain; limiting instrumental ADL	Severe pain; limiting self care ADL	-
	diarrhea	None	Increase of <4 stools per day over baseline; mild increase in ostomy output compared to baseline	Increase of 4 - 6 stools per day over baseline; moderate increase in ostomy output compared to baseline; limiting instrumental ADL	Increase of ≥7 stools per day over baseline; hospitalization indicated; severe increase in ostomy output compared to baseline; limiting self care ADL	Life-threatening consequences; urgent intervention indicated

14.6. List of specific immune mediated diseases (pIMDs)

Neuroinflammatory disorders	Musculoskeletal disorders	Skin disorders	Liver disorder	Gastrointestinal disorders	Metabolic & endocrine disorders	Vasculitides	Others
Cranial nerve inflammatory disorders, including paralyses/paresis (e.g., Bell's palsy)	Systemic lupus erythematosus	Psoriasis	Autoimmune hepatitis	Crohn's disease	Autoimmune thyroiditis (including Hashimoto thyroiditis)	Large vessels vasculitis including: giant cell arteritis such as Takayasu's arteritis & temporal arteritis	Autoimmune haemolytic anaemia
Acute disseminated encephalomyelitis including site- specific variants: encephalitis, encephalomyelitis, myelitis, myeloradiculoneuritis, cerebellitis	Systemic sclerosis (with limited or diffuse cutaneous involvement)	Vitiligo	Primary biliary cirrhosis	Ulcerative colitis	Grave's or Basedow's disease		Autoimmune thrombocytopenia
Multiple sclerosis	Dermatomyositis	Erythema nodosum	Primary sclerosing cholangitis	Ulcerative proctitis	Diabetes mellitus type I		Antiphospholipid syndrome
Transverse myelitis	Polymyositis		Autoimmune cholangitis.	Celiac disease	Addison's disease		Pernicious anaemia
Optic neuritis	Anti-synthetase syndrome	Cutaneous lupus erythematosus					Raynaud's phenomenon
Narcolepsy	Rheumatoid arthritis	Alopecia areata					Uveitis
	Juvenile chronic arthritis (including Still's disease)	Lichen planus					Autoimmune myocarditis/cardiomyopathy
	Polymyalgia rheumatica	Sweet's syndrome					Sarcoidosis
	Psoriatic arthropathy	Morphoea					Stevens-Johnson syndrome
	Relapsing polychondritis						Sjögren's syndrome
Myasthenia gravis (including Lambert-Eaton myasthenic syndrome)	Mixed connective tissue disorder						Idiopathic pulmonary fibrosis
							Goodpasture syndrome
Immune mediated peripheral neuropathies and plexopathies, (including Guillain-Barré syndrome, Miller Fisher syndrome and other variants, chronic inflammatory demyelinating polyneuropathy, multifocal motor neuropathy and polyneuropathies associated with monoclonal gammopathy)	Spondyloarthritis, including ankylosing spondylitis, reactive arthritis (Reiter's Syndrome) and undifferentiated spondyloarthritis	Autoimmune bullous skin diseases (including pemphigus, pemphigoid & dermatitis herpetiformis)				Medium sized and/or small vessels vasculitis including: polyarteritis nodosa, Kawasaki's disease, microscopic polyangiitis, Wegener's granulomatosis, Churg–Strauss syndrome (allergic granulomatous angiitis), Buerger's disease (thromboangiitis obliterans), necrotising vasculitis & anti-neutrophil cytoplasmic antibody (ANCA) positive vasculitis (type unspecified), Henoch-Schonlein purpura, Behcet's syndrome, leukocytoclastic vasculitis	Autoimmune glomerulonephritis (including IgA nephropathy, glomerulonephritis rapidly progressive, membranous glomerulonephritis, membranoproliferative glomerulonephritis, & mesangioproliferative glomerulonephritis)

Adapted from Tavares Da Silva, F et al., Optimal approaches to data collection and analysis of potential immune mediated disorders in clinical trials of new vaccines, Vaccine, 2013 [91]

[redacted]

[redacted]

[redacted]

[redacted]

[redacted]

[redacted]

[redacted]

[redacted]

[redacted]

[redacted]

-

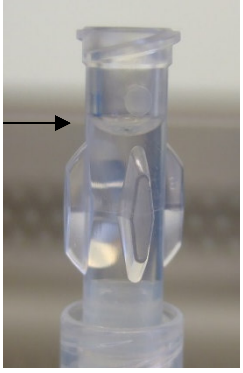

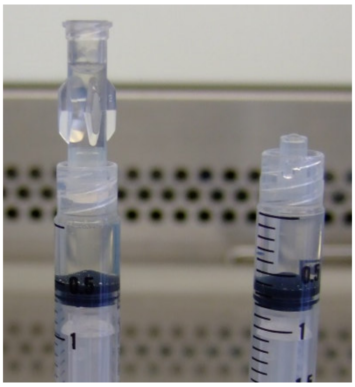

- [redacted]

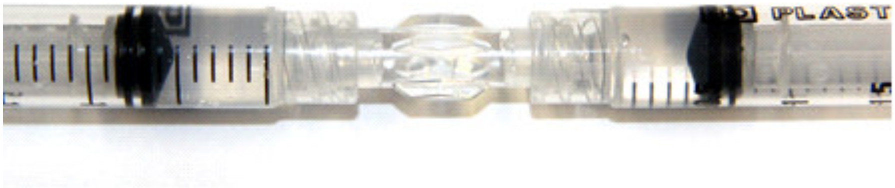

- [redacted]

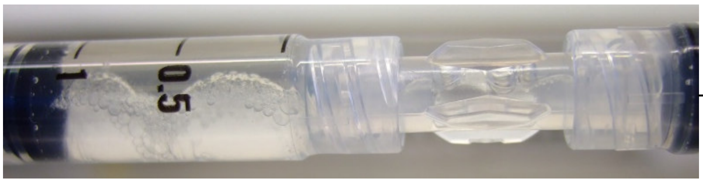

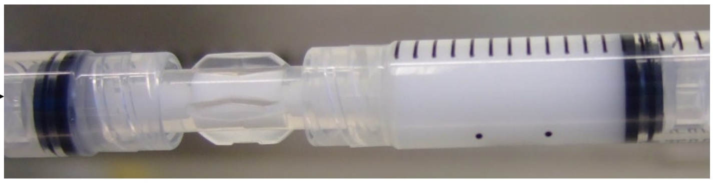

[redacted]

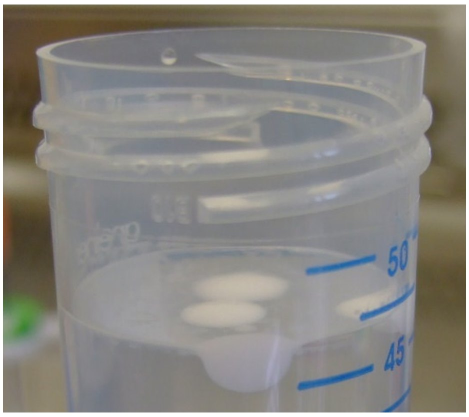

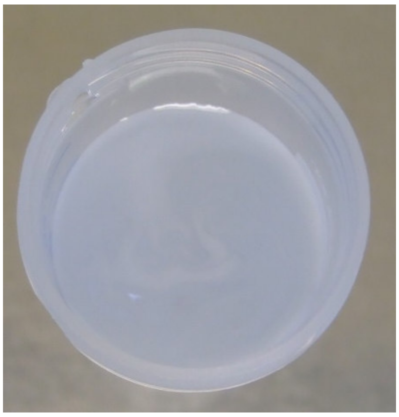

[redacted]

[redacted]

[redacted]

[redacted]

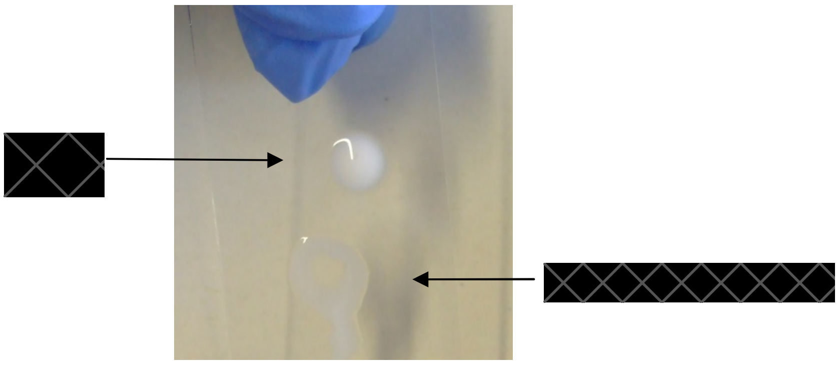

[redacted]

[redacted]

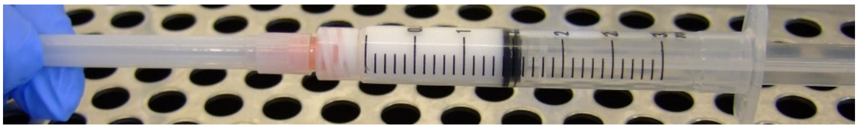

[redacted]

[redacted]

Document	Content
**Protocol**	V1.3, 15.02.2021
V. Synopsis	Indication:<br>Part II: Adults aged > 55 years<br><br>Number of Volunteers:<br>Total number of volunteers: 36<br>Part I: 12<br>Part II:24<br><br>Inclusion Criteria:<br>Part II: Age > 55 years at the time of screening<br>2.Part II: With or without pre-existing medical condition, not requiring change in therapy or hospitalization before enrollment<br><br>Description of the Medical Products<br>1.SARS-CoV-2 peptides: Six promiscuous HLA-DR-restricted peptides (250 µg each) derived from different proteins of SARS-CoV-2<br><br>Study Design<br>Part II:<br>24 subjects will receive an open-label 500 µl subcutaneous injection via needle and syringe of the study IMP (CoVac-1).<br><br>Statistics, Safety Variables and Stopping Rules:<br>Part II: n=24
Table 1: Table of Events	7. Enrolment: volunteers are enrolled and registered through a screening procedure. Each volunteer will be registered under a specific Vol. ID on a subjects log kept at the trial site<br>21.Serological response: 10 ml of serum for analysis of serological response will be analysed by the Immunopathological Laboratory, University Hospital Tuebingen (central laboratory). Blood will be taken before peptide vaccination on V1, and during vaccination phase and follow-up at each visit.
1.1.3.1 Dose rationale for peptides	Preliminary data from a healthy volunteer and cancer patients vaccinated with a personalized peptide vaccine (240-300 µg per peptide) including two of the CoVac-1 peptides (250µg) in combination with XS15 showed potent induction of T-cell responses in 100% of HV and patients and a good safety profile.

	The dose of ~250 µg per peptide per dose for CoVac-1 vaccine was selected based on these findings and on the feasibility in pharmaceutical development of the vaccines.
1.1.4 Rationale for trial design	• Part II: Adults aged > 55. After proving safety and immunogenicity in a cohort of healthy volunteers aged 18-55 (Part I), an interim safety analysis will be conducted and prior to continuation with Part II approval by DSMB and of an amendment by PEI and Ethics Committee must be obtained.
1.1.5 Preliminary experiences from study part I	P-pVAC-SARS-CoV-2 is a phase I single-center safety and immunogenicity trial of multi-peptide vaccination with CoVAC-1 to prevent COVID-19 infection in adults. The study is recruiting since November 2020 and has completed the first part (healthy volunteers (n=12), age 18-55 years) in February 2021. One single subcutaneous vaccination of CoVAC-1 was applied. Immunogenicity, in term of induction of T-cell responses to one or more of the six HLA-DR SARS-CoV-2 T cell epitopes included in the CoVAC-1 vaccine was assessed pre vaccination as well as on day 7, 15 and 28 after vaccination (please refer to the IB of CoVAC-1 for more details). Induction of SARS-CoV-2 T cells was shown in 100 % (12/12) of volunteers in part I of the study. Earliest T cell responses were observed at day 14 (V3) for 11/12 volunteers. Immune responses were induced to multiple of the vaccine peptides (median 5/volunteer, range 4-6).<br>First safety data of CoVAC-1 are available until d28 (V4) after vaccination. As intended and expected all volunteers (n=12) developed a granuloma local at injection site. Further local injection site adverse events included transient erythema, itching, pain and skin ulceration.<br>Until day 28 no relevant systemic side effects, especially no fever or other inflammatory reactions were reported. No allergic reactions were observed. For a detailed description of all ADRs reported please refer to the IB of CoVAC-1.<br>Thus, these preliminary data suggest a high immunogenicity of CoVAC-1 to induce early and multi-peptide T cell responses as well as a good tolerability and safety profile.
1.2. Benefit/Risk Assessment	•Preliminary safety and immunogenicity analyses of the volunteers vaccinated with CoVAC-1 in part I of the study (n = 12) have proven high immunogenicity with the induction of early, multi-peptide-directed functional T cell responses in 100% of volunteers as well as a good safety profile with no systemic adverse drug reactions or allergic reactions.<br><br>•The trial comprises two parts (cohorts of participants) with different age ranges to provide preliminary results on safety in a cohort of young (18-55 years, n=12) and healthy participants, which is then extended to older (Part II) participants. Of note, the risk of vaccine related (S)AEs is hypothesized to be similar in each age group.<br>•Confirming safety of the CoVac-1 vaccine in volunteers within the P-pVACSARS-CoV-2 study will further allow the transfer of this approach to induce SARSCoV-2 specific T-cell immunity in a therapeutic setting for patients with SARS-CoV-2 infection.

1.3 Data Safety Monitoring Board (DSMB)	The DSMB will receive a report listing and summarizing all the relevant safety data at least twice. The first assessment (first interim safety report, section 9.5) will take place after Part I of the trial including DSMB approval and an amendment at the regulatory authorities (Paul-Ehrlich Institute, PEI) and Ethics Committee (EC). If the IMP is considered safe for continuation by DSMB, Part II of the trial will start recruiting. ~~After completion of Part II, the second DSMB report (second interim safety report, section 9.5) will be created and the DSMB has to approve continuation again. This report will be made available for EC.~~ In addition, the report will provide data concerning recruiting rates, status of the trial and AESIs (section 9.1.4); also non-occurrence will be mentioned. ~~Based on its review, the DSMB will provide the sponsor with recommendations regarding trial modification and continuation or termination of the trial.~~ An emergency meeting of the DSMB may be called at any time should questions of volunteer safety arise or holding rules apply, and necessary safety reports will be provided. Meetings may be convened as conference calls/e-mail as well as in person.
3. Study Design	Part II ~~and III~~ must not start recruiting prior to approval by authorities. Volunteers of part II are treated simultaneously ~~and 28 days following vaccination of the 12th volunteer, there will be an interim analysis of safety and a safety review by the DSMB whether to proceed to next Part III. Volunteers of part III are treated simultaneously (2 participants per day).~~ Details can be found in figure 3.
4. Study Population	Healthy adult women and men aged 18-55 (Part I), followed by ~~healthy~~ adult women and men aged ~~56-74~~> 55 with age adjusted health condition (Part II) ~~and adult women and men aged ≥ 75 (Part III)~~.
4.1.1. Inclusion Criteria	2. Part II: Age >56-74~~5~~ years at the time of screening<br>3. ~~Part III: Age ≥ 75 years at the time of screening~~<br><br>Part I ~~and II~~: Free of clinically significant health problems, as determined by pertinent medical history and clinical examination at study screening<br>Part II: With or without pre-existing medical condition, not requiring change in therapy or hospitalization before enrollment
4.1.2. Exclusion Criteria	16. Pre-existing auto-immune disease except for Hashimoto thyroiditis and mild (not requiring immunosuppressive treatment) psoriasis
5.1.1. Peptide cocktail	Each volunteer enrolled in the P-pVAC-SARS-CoV-2 trial will receive 6 promiscuous HLA-DR peptides (250 µg each) derived from different proteins of SARS-CoV-2. Details on drug substance can be found in Table 3
5.7 Dose Schedule	The mixing of the peptide vaccine cocktail and Montanide ISA 51 VG will be performed by local pharmacy and the investigator will be provided with a syringe containing the final drug product CoVac-1. A subcutaneous injection of 500 µl (approx. 250 µg per peptide, 50µg XS15) will be applied. A single vaccination per patient will be conducted.
5.7.2.1 Side effects of peptide vaccination	Preliminary safety results of volunteers (n = 12) in part I of the P-pVAC-SARS-CoV-2 study showed as intended and expected developed a local granuloma at injection site in all volunteers (100%). Further local injection site adverse events included transient erythema (100%), swelling (100%), itching (83%), pain (58%) and skin ulceration (8%). Until day 28 no relevant systemic side effects, especially no fever or other inflammatory reactions were reported. No allergic reactions were observed. In some participants fatigue (25%), headache (16%), nausea (16%), myalgia (8%) and arthralgia (8%) were reported.

5.7.2.3 Side effects of Montanide ISA 51 VG	Further side effects rarely reported were erythema nodosum (2/36 patients, 5%) and the development of sterile abscesses at injection site (10%)
6.3.2.Methods and Timing for Assessing, Recording, and Analysing of Efficacy Parameters	Spots are counted using an Immunospot analyzer. ~~T cell responses are considered to be positive when the mean spot count per well is at least 3-fold higher than the mean number of spots in the negative control wells~~ (according to the cancer immunoguiding program (CIP) guidelines).<br><br>To differ between vaccine induced antibody response additional standard Elecsys® Anti-SARS-CoV-2 assay supplied by F. Hoffmann-La Roche AG, Basel, Switzerland or ADVIA Centaur SARS-CoV-2 Total (COV2T) (Siemens Healthcare Diagnostics GmbH) will be performed at central laboratory of the University Hospital Tuebingen.
6.5.Vaccination holding rules	The holding rules are as follow:<br>• Solicited local ADRs: If more than 30% of injections are followed by Grade ≥3 solicited swelling or pain or Grade 4 redness (first occurrence at any time after vaccination)~~beginning within 3 days after injection (day of injection and 2 subsequent days)~~ and persisting at Grade 3 (swelling or pain)/4 (redness) for > 48 h to maximum 72 hours depending upon symptom severity and kinetics.
9.1.2. Adverse Drug Reaction	Local solicited ADRs:<br>•Swelling at site of injection<br>•Erythema at site of injection<br>•Pain or itching at site of injection<br>•Formation of granuloma at the injection site<br>•Superficial skin ulceration A grading for severity of ADRs can be found in appendix 14.5 as guidance.
**Patienteninformation**	**Version 1.3, 15.02.2021**
Warum wird diese Prüfung durchgeführt?	Bislang existieren zwar mehrere zugelassene Impfstoffe, jedoch ist die Verfügbarkeit für die breite Bevölkerung limitiert, d.h. es kann nicht jedem der geimpft werden möchte, ein Impfangebot gemacht werden. Langzeiterfahrungen mit den zugelassenen SARS-CoV-2 Impfstoffen fehlen bisher.<br><br>Die Studie behandelt Menschen ohne ein erhöhtes Risiko für eine schwere COVID¬19 Erkrankung, gliedert sich aber in ~~drei~~ zwei Abschnitte, da Probanden aus unterschiedlichen Altersgruppen behandelt werden:<br>• Abschnitt I: 12 gesunde Erwachsene im Alter von 18-55 Jahren<br>• Abschnitt II: 12 Erwachsene im Alter ~~von~~ über 55~~6~~-74 Jahren<br>• ~~Abschnitt III: 12 Erwachsene älter als 75 Jahre~~
Werden alle Probanden auf einmal behandelt ?	Wie bereits weiter oben aufgeführt, werden in dieser Studie Menschen aus verschiedenen Altersgruppen in zwei Studienabschnitten behandelt. Da im Rahmen dieser Studie erstmals der Impfstoff CoVac-1 genutzt wird, wird nach der Impfung des ersten Probanden für einen Monat kein weiterer Proband in die Studie eingeschlossen. Danach werden die Nebenwirkungen des ersten Probanden bewertet, bevor die Impfung weiterer Probanden fortgesetzt wird. Wenn die Bewertung positiv ausfällt, wird danach im ersten Studienabschnitt immer ein Proband pro Tag geimpft. Nach Erreichen der maximalen Anzahl der Probanden im ersten Studienabschnitt erfolgt eine Zusammenfassung der Daten und eine

	Bewertung der Sicherheit durch unabhängige Experten auf dem Feld der Infektionserkrankungen und Impfungen. Diese Bewertung zusammen mit der Zusammenfassung der Daten wird an die zuständigen regulatorischen Behörden (Paul Ehrlich Institut und Ethik Kommission) zur Zweitbewertung geschickt. Nur wenn diese Bewertung positiv ausfällt und die Fortsetzung der Studie genehmigt wird, kann mit dem nachfolgenden Studienabschnitt begonnen werden
Voruntersuchungs-Visite / Screening Visite	(…)teilt wird diese Ihnen dies Ihr Prüfarzt mitteilen.
Welche Risiken und Nebenwirkungen können mit der Teilnahme an der Studie verbunden sein?	Erste vorläufige Sicherheitsdaten liegen bereits von den Probanden des ersten Studienabschnittes vor. Zudem kommen Hinweise auf mögliche Nebenwirkungen kommen daher aus früheren Studien mit ähnlichen Peptidimpfstoffen, die insbesondere bei Patienten mit Krebserkrankungen angewendet wurden<br><br>Wahrscheinliche Nebenwirkungen (basierend auf der Auswertung der Probanden im ersten Teil der P-pVAC-SARS-CoV-2 Studie)<br>Die häufigsten beobachten Nebenwirkungen von CoVAC-1 sind Lokalreaktionen an der Einstichstelle wie die Bildung eines Granuloms (Knoten an der Impfstelle 100%), Rötung der Haut (Erytheme, 100%), Schwellungen (Ödeme, 100%), Juckreiz (83%), Hautirritationen (nicht näher beschriebene Veränderungen an der Haut, keine exakten Häufigkeitsangaben, gelegentlich), Schmerz (58%), Überempfindlichkeit und eine minimale Eröffnung der oberflächlichen Haut (Ulzeration, 8%). Schwere systemische Nebenwirkungen wie beispielsweise Fieber sind bislang nicht aufgetreten. Ebenso wurden keine allergischen Reaktionen beobachtet. Ein Teil der Probanden hatte Müdigkeit (25%), Kopfschmerzen (16%), Übelkeit (16%), Muskel- und Gelenkbeschwerden (8%).<br><br>Weitere mögliche Nebenwirkungen (basierend auf Erfahrungen aus anderen Peptid-Impfstudien)<br>Schwere mögliche Nebenwirkungen (basierend auf Erfahrungen aus anderen Peptid-Impfstudien)<br><br>(…)<br>Die Anwendung von XS15 zusammen mit einer Peptidimpfung in Montanide ISA 51 VG bei einem gesunden Probanden und mehreren Tumorpatienten im Vorfeld dieser Studie zeigte, neben der Bildung einer lokalen Verhärtung, sogenanntes Granulom, an der Impfstelle, keine relevante Nebenwirkung, insbesondere keine allergische oder anaphylaktische Reaktion.

P-pVAC-SARS-CoV-2: Phase I single-center safety and immungenicity trial of multi-peptide vaccination to prevent COVID-19 infection in adults

Short Title of Clinical Trial	P-pVAC-SARS-CoV-2
Protocol Version	V1.2
Date of Protocol	07.10.2020
EudraCT-Number	2020-002502-75
ClinicalTrials.gov-Number	
Phase	Phase I
Sponsor	University Hospital Tuebingen, [redacted] 72076 Tuebingen Germany
Investigational Medicinal Product	Multi-peptide vaccine based on SARS-CoV-2 HLA class II peptides, applied subcutaneously together with the TLR1/2 ligand XS15 emulsified in Montanide ISA 51 VG
Summary of the revision history (amendments)	None

CONFIDENTIAL This protocol contains confidential information and is intended solely for the guidance of clinical investigation. This protocol may not be disclosed to parties not associated with the clinical investigation or used for any purpose without the prior written consent of the coordinating Investigator.

I. Table of Contents

Title Page 1

I. Table of Contents 2

I.a) List of Tables 7

I.b) List of Figures 7

II. Signature Page 9

III. Contacts 11

IV. Abbreviations 14

V. Synopsis 16

1. Introduction 29

1.1. Trial Rationale and Justification 32

1.1.1. Mechanism of action and rational for a prophylactic SARS-CoV-2 multi-peptide vaccine 32

1.1.2. Rational for the usage of XS15 as adjuvant in the prophylactic SARS-CoV-2 multi-peptide vaccine 33

1.1.3. Rational for selected doses 34

1.1.3.1. Dose rational for peptides 34

1.1.3.2. Dose rational for XS15 35

1.1.3.3. Dose rationale for Montanide ISA 51 VG 36

1.1.3.4. Rationale for one dose schedule 36

1.1.4. Rational for trial design 37

1.2. Benefit / Risk Assessment 38

1.3. Data and Safety Monitoring Board (DSMB): 41

2. Study Objectives 43

2.1. Primary Objective and Endpoint 43

2.1.1. Primary Endpoint 43

2.2. Secondary Objectives and Endpoints 43

2.2.1. Secondary Endpoints 43

2.3. Exploratory Objectives and Endpoints 43
2.3.1. Exploratory Endpoints 44
3. Study Design 45
3.1. Study Duration and Schedule 47
3.2. End of Study 48
4. Study Population 49
4.1. General Criteria for Subject Selection 49
4.1.1. Inclusion Criteria 49
4.1.2. Exclusion Criteria 50
5. General Information on the Investigational Medical Product (IMP) 52
5.1. Peptide Vaccine CoVac-1 52
5.1.1. Peptide cocktail 52
5.1.1.1. SARS-CoV-2-specific peptides (drug substance) 52
5.1.1.1. TLR1/2 ligand XS15 (drug substance) 52
5.1.2. Montanide ISA 51 VG 53
5.2. Manufacturing of the Investigational Medicinal Product 55
5.2.1. SARS-CoV-2-specific peptides (drug substance) 55
5.2.2. XS15 (drug substance) 55
5.2.3. Montanide ISA 51 VG 55
5.2.4. Peptide cocktail CoVac-1 (drug product) 55
5.3. Labeling of the Investigational Medicinal Product 56
5.3.1. Peptide cocktail 56
5.3.2. Montanide ISA 51 VG 56
5.4. Storage of the Investigational Medicinal Product 56
5.5. Drug Accountability, Therapy Compliance and Disposal 57
5.6. Method of Treatment Assignment 57
5.7. Dose Schedule 58
5.7.1. Dose modifications for peptide vaccine 58

5.7.2. Side effects 58
5.7.2.1. Side effects of peptide vaccination 58
5.7.2.2. Side effects of XS15 59
5.7.2.3. Side effects of Montanide ISA 51 VG 59
6. Study Procedures and Examination Method 61
6.1. Study Entry 61
6.1.1. Volunteer’s Informed Consent 61
6.1.2. Screening 61
6.1.3. Enrolment 62
6.1.4. Randomisation 62
6.1.5. Concomitant Medication and Treatments 62
6.1.6. Permitted Prior and Concomitant Medications and Treatments 62
6.1.7. Prohibited Prior and Concomitant Medications and Treatments 63
6.1.8. Contraception 63
6.2. Vaccination Phase 64
6.2.1. Visit 1 (Vaccination) (Day 1) 64
6.2.2. Visit 2 (Day 7 +/- 1) 65
6.2.3. Visit 3 (Day 14 +/- 1) 65
6.2.4. Visit 4 (Interim safety) (Day 28 +/- 2) 65
6.2.5. Visit 5 (End of Safety follow-up = EOSf) 66
6.2.6. Visit 6-7 (Follow-up) (Month 3 and 6 +/- 7 days) 66
6.2.7. Volunteer’s diary/card 66
6.2.8. Unscheduled Visit 66
6.3. Assessment of Efficacy 67
6.3.1. Efficacy Parameters 67
6.3.2. Methods and Timing for Assessing, Recording, and Analysing of Efficacy Parameters 67
6.4. Assessment of Safety 69

6.4.1. Safety parameters 69

6.4.2. Methods and Timing for Assessing, Recording, and Analysing Safety Parameters 70

6.5. Vaccination holding rules 70

6.6. Premature termination of clinical trial for a trial subject 71

6.7. Premature closure of a trial site 72

6.8. Premature termination of the trial 72

6.9. Follow Up 73

6.10. End of Study for Subjects 73

7. Quality control and Quality assurance 74

7.1. Risk-based approach 74

7.2. Monitoring 74

7.3. Audits/ Inspections 75

7.4. Documentation: Collection, Handling, Storage and Archiving of Data 75

7.4.1. Case Report Form 75

7.4.2. Source Data 76

7.4.3. Data Handling 76

7.4.4. Preparation/Handling/Storage/Accountability of biological samples 76

7.4.5. Handling of missing data and drop outs 77

7.4.6. Storage and Archiving of Data 77

8. Statistical Analyses 78

8.1. Study Population Definition 78

8.1.1. Sample Size and Power Consideration 78

8.2. Analysis Primary Variables 78

8.3. Analysis Secondary Variables 78

8.4. Subgroup Analysis 79

8.5. Interim Analysis 79

8.6. Stopping Rules 79

8.7. Biometric Report 80

9. Safety 81

9.1. Definition of Adverse Events and Side Effects 81

9.1.1. Adverse Events 81

9.1.2. Adverse Drug Reaction 81

9.1.3. Expectedness 82

9.1.4. AESI (adverse events of special interest) 83

9.1.5. Serious Adverse Event and Serous Adverse Reaction 83

9.2. Period of Observation 84

9.3. Documentation and Reporting of Adverse Events 84

9.3.1. Documentation and Reporting of Adverse Events by the Investigator 84

9.3.2. Assessment of Severity and Causality 85

9.3.3. Action taken 86

9.3.4. Sponsors Assessment of the SAEs 86

9.3.5. Follow-up of Initial Report 86

9.3.6. Exception of reporting 87

9.3.7. Suspected Unexpected Serious Adverse Reaction (SUSAR) 87

9.3.8. Expedited Reporting to the Regulatory Authorities 87

9.4. Examination and Report of Changes in the Risk to Benefit Ratio 88

9.4.1. Reporting to Data and Safety Monitoring Board 88

9.4.2. Report to the Investigator 88

9.5. Interim Safety analysis 88

9.6. Annual Safety Report 89

9.7. Deviations from the Protocol 89

9.8. Reporting of Pregnancy 89

10. Regulatory Consideration 92

10.1. Ethical Conduct of Clinical Study 92

10.1.1. Good Clinical Practice, Declaration of Helsinki and legal Provision 92

10.2. Subject Information and Informed Consent 92
10.3. Insurance 92
10.4. Confidentiality 93
10.5. Responsibility of the the Investigator 94
10.6. Registration of the Trial 95
10.7. Continuous Information to Independent Ethics Committee 95
10.8. Approval of Protocol and Subsequent Amendments 95
11. Publications 96
11.1. Reports 96
11.2. Publication 96
12. Financing 97
13. Literature 98
14. Appendix 106
14.1. Common Terminology Criteria for Adverse Events (CTCAE) Version 106
14.2. List of central laboratories 106
14.3. Volunteer diary 107
14.4. Volunteer card 118
14.5. Intensity of solicited and unsolicited local and systemic adverse events 119
14.6. List of specific immune mediated diseases (pIMDs) 121
14.7. “Mischanleitung“ for the pharmacy of participating centers 122

I.a) List of Tables

Table 1: Table of Events 27
Table 2**:** Study Timelines 48
Table 3: SARS-CoV-2 specific HLA-DR vaccine peptides 54

I.b) List of Figures

Figure 1: Overall Study Design 45

Figure 2: Individual Study Procedure 47

Figure 3: Treatment sequence 47

II. Signature Page

The present trial protocol was subject to critical review and has been approved in the present version by the persons signed.

Sponsor: The University Hospital Tuebingen is sponsor for the purpose of § 4 (24) German Drug Law with complementary regulations. The internal responsibility to comply with the obli ations of the s onsor in terms of these re ulations sta s with [redacted]

Name: [redacted]

Function: Sponsor's delegate and person in charge to meet the obligations of the sponsor

[redacted]

ame: [redacted]

Function: Biometrician

Declaration of the Principal Investigator

By my signature, I agree to supervise personally the conduct of this study at my study site and to ensure its conduct is in compliance with the protocol, informed consent, the national laws, the ICH Good Clinical Practices Guidelines and the Declaration of Helsinki. I will train the involved ersonal accordin l . [redacted]

Function: Principal Investigator, Leiterin der klinischen Prüfung according to § 4 German Drug Law (AMG)

[redacted]

Name: [redacted]

Function: Deputy Principal Investigator

Adress of the Study Center: [redacted]

III. Contacts

Sponsor

Universitätsklinikum Tuebingen
Geissweg 3
72076 Tuebingen

Sponsor's Delegate

[redacted]
Phone: [redacted]
Fax: [redacted]
e-mail: [redacted]

Coordinating Investigator (CI)

Leiterin der klinischen Prüfung, according to § 4 German Drug Law (AMG)

[redacted]
Phone: [redacted]
Fax: [redacted]
e-mail: [redacted]

Co-Coordinating Investigator

[redacted]
Phone: [redacted]
Fax: [redacted]
e-mail: [redacted]

Scientific Coordinators

[redacted]
Phone: [redacted]
Fax: [redacted]

e-mail: [redacted]

[redacted]
Phone: [redacted]
Fax: [redacted]
e-mail: [redacted]

Biometrician

[redacted]
Phone: [redacted]
Fax: [redacted]
e-mail: [redacted]

Data management

[redacted]
Phone: [redacted]
Fax: [redacted]

Project management

[redacted]
Phone: [redacted]
Fax: [redacted]
e-mail: [redacted]

Monitoring

[redacted]
Phone: [redacted]
Fax: [redacted]
e-mail: [redacted]

SAE-Management

[redacted]
Phone: [redacted]
Fax: [redacted]
e-mail: [redacted]

IV. Abbreviations

ADR	Adverse Drug Reaction
ADE	Antibody-dependent Enhancement
ADL	Activities of Daily Living
ADV	Adenovirus
AE	Adverse Event
AESI	Adverse Event of Special Interest
AMG	German Drug Law (Deutsches Arzneimittelgesetz)
CCR	Cellular Conversion Rate
CI	Coordinating Investigator
CNS	Central Nervous System
COPD	Chronic Obstructive Pulmonary Disease
COVID-19	Coronavirus Disease 2019
COV	Coronavirus
CMV	Cytomegalovirus
CRF	Case Report Form
CTC(AE)	Common Toxicity Criteria (for Adverse Events)
CTR	Clinical trial report
DBL	Data Base Lock
DSMO	Dimethyl sulfoxide
DSMB	Data and Safety Monitoring Board
EBV	Epstein-Barr Virus
EC	Ethics Committee
EORTC	European Organisation for Research and Treatment of Cancer
EOSf	End of Safety follow-up
FCBP	Female of Child Bearing Potential
FSI	First Subject In
GCP	Good Clinical Practice
GCP-V	Good Clinical Practice Ordinance (GCP-Verordnung)
GMP	Good Manufacturing Practice
GMT	Geometric mean titer

HLA	Human Leukocyte Antigen System
HRT	Hormone Replacement Therapy
IB	Investigator’s Brochure
IC	Informed Consent
ICH	International Conference on Harmonization of Technical Requirements for Registration of Pharmaceuticals for Human Use
ICU	Intensive Care Unit
IMP	Investigational Medicinal Product
ISF	Investigator Site File
LSI	Last Subject In
LSO	Last Subject Out
MERS-CoV	Middle East Respiratory Syndrome Coronavirus
PCR	Polymerase Chain Reaction
PBMC	Peripheral Blood Mononuclear Cell
PEI	Paul-Ehrlich-Institut
pIMD	Potential Immune Mediated Disease
RNA	Ribonucleic acid
SARS-CoV-2	Severe Acute Respiratory Syndrome - Coronavirus 2
SAE	Serious Adverse Event
SmPC	Summary of Product Characteristics (deutsch: Fachinformation)
SDV	Source Data Verification
SOP	Standard Operating Procedure
SPC	Summary of Product Characteristics
SUSAR	Suspected Unexpected Serious Adverse Reaction
TLR	Toll-like receptor
TMF	Trial Master File

V. Synopsis

Sponsor	University Hospital of Tuebingen represented by<br>Medical Director: Prof. Dr. med. M. Bamberg<br>Director of Administration: G. Sonntag
Title	P-pVAC-SARS-CoV-2: Phase I single center safety and immungenicity trial of multi-peptide vaccination to prevent COVID-19 infection in adults
Short Title	P-pVAC-SARS-CoV-2
Coordinating Investigator<br>(Leiter der klinischen Prüfung, According to § 4 German Drug Law (AMG))<br><br>Co-Coordinating Investigator<br><br>Sponsor's Delegate<br><br>Scientific Coordinator	[redacted]<br><br>[redacted]<br><br>[redacted]<br><br>[redacted]
Indication	Part I: Adults aged 18-55 years<br>Part II: Adults aged 56-74<br>Part III: Adults aged ≥ 75
Number of Volunteers	Total number of volunteers: 36<br>Part I: 12<br>Part II: 12<br>Part III: 12

Inclusion Criteria	1. Adult male or non-pregnant, non-lactating female<br>1. Part I: Age 18-55 at the time of screening<br>2. Part II: Age 56-74 years at the time of screening<br>3. Part III: Age ≥ 75 years at the time of screening<br>2. Pre-existing medical condition<br>1. Part I and II: Free of clinically significant health problems, as determined by pertinent medical history and clinical examination at study screening<br>2.<br>3. Ability to understand and voluntarily sign an informed consent form<br>4. Ability to adhere to the study visit schedule and other protocol requirements<br>5. Female volunteers of child bearing potential (FCBP) and male volunteers with partners of child bearing potential, who are sexually active, must agree to the use of two effective forms (at least one highly effective method) of contraception. This should be started from the signing of the informed consent and continue until three months after vaccination

Inclusion criteria	6. Postmenopausal or evidence of non-child-bearing status. For women of childbearing potential: negative urine or serum pregnancy test within 7 days prior to study treatment. Postmenopausal or evidence of non-childbearing status is defined as:<br>1. Amenorrhoea for 1 year or more following cessation of exogenous hormonal treatments<br>2. Luteinizing hormone (LH) and Follicle stimulating hormone (FSH) levels in the postmenopausal range for women under 50<br>7. Be willing to minimize blood and body fluid exposure from others for 7 days after vaccination<br>1. Use of effective barrier prophylaxis, such as latex condoms, during sexual intercourse<br>2. Avoiding the sharing of needles, razors, or toothbrushes<br>3. Avoiding open-mouth kissing<br>8. Refrain from blood donation during the course of the study

Exclusion Criteria	1. Pregnant or lactating females<br>2. Participation in any clinical study with intake of any investigational drug interfering with the study primary endpoint including:<br>o Active infection<br>o Psychatric disorders<br>o Known systemic anaphylaxis<br>3. Any concomitant disease affecting the effect of the therapeutic vaccine or interfering with the study primary endpoint<br>4. Any immunosuppressive treatment except low dose corticosteroids (equivalent to ≤10mg prednisolone/day)<br>5. Prior or current infection with SARS-CoV-2 tested serologically or by throat/nose swab (PCR)<br>6. History of Guillain-Barré syndrome<br>7. Positive serological HIV, hepatitis B or C test. In case of positive HBsAg, volunteer must provide prove of hepatitis B vaccination, otherwise volunteer must be excluded.<br>8. History of relevant CNS pathology or current relevant CNS pathology (e.g. seizure, paresis, aphasia, cerebrovascular ischemia/haemorrhage, severe brain injuries, dementia, Parkinson's disease, cerebellar disease, organic brain syndrome, psychosis, coordination or movement disorder, excluding febrile seizures as child)<br>9. Baseline laboratory with lymphocyte count ≤ 1000/µl<br>10. <u>Only Part I</u><br>o Acute or chronic, clinically significant psychiatric, hematologic, pulmonary, cardiovascular, or hepatic or renal functional abnormality as determined by the Investigator based on medical history, physical exam, and/or laboratory screening test

	11. All parts of the clinical trial<br>o Diabetes mellitus Typ II requiring drug treatment<br>o Chronic lung disease requiring drug treatment<br>o Any chronic liver disease or unknown liver abnormalities defined as:<br>• ALT and AST ≤ 2.5 x ULN<br>• γ-GT ≤ 2.5 x ULN<br>o Chronic renal failure defined as GFR < 60 ml/min/1,73m^2<br>o Serious pre-existing cardiovascular disease such as NYHA ≥ I, coronary heart disease requiring coronary surgery or known pAVK ≥ grade 2<br>o Sickle cell anemia<br>o Obesity (as defined by age adjusted body mass index)<br>12. Hospitalization at study inclusion<br>13. Administration of immunoglobulins and/or any blood products within the 120 days preceding study entry or planned administration during the study period<br>14. History of blood donation within 30 days of enrolment or planned donations within the study period<br>15. Known hypersensitivity to any of the components included in the CoVac-1 vaccine<br>16. Pre-existing auto-immune disease except for Hashimoto thyroiditis and mild (not requiring immunosuppressive treatment) psoriasis

<table>
<tr>
<td>Description of the Medical Products</td>
<td>
<u>IMP/Drug product/Peptide vaccine: CoVac-1</u> applied as one multipeptide cocktails consisting of:

<u>SARS-CoV-2 peptides:</u> Six promiscuous HLA-DR-restricted peptides (240 µg each) derived from different proteins of SARS-CoV-2
<u>XS15:</u> The lipopeptide XS15 is a water-soluble synthetic Pam_3Cys-derivative. As TLR1/2 ligand it will be included as an adjuvant in the peptide vaccine.

Peptides are synthesized in the GMP-certified Wirkstoffpeptidlabor at the University of Tuebingen (Prof. Stefan Stevanović) and will be formulated at the GMP-Center of the University Hospital Tuebingen. The GMP-certified Wirkstoffpeptidlabor specializes in multipeptide cocktails with variable composition and holds a production permit (Herstellungserlaubnis) for different multipeptide cocktails including the TLR 1/2 ligand XS15.
<ol start="3">
<u>Montanide ISA 51 VG:</u> Prior to application, the peptide cocktail (consisting of 6 SARS-CoV-2-derived peptides and XS15) will be emulsified in a water-oil emulsion 1:1 with Montanide ISA 51 VG to a final volume of 500 µl.

</td>
</tr>
<tr>
<td></td>
<td>
<u>Treatment schedule:</u>
A single vaccination with the IMP CoVac-1 (SARS-CoV-2 HLA-DR peptides, XS15 emulsified in Montanide ISA 51 VG) (500 µl) will be applied subcutaneously (s.c.) to the abdominal skin.
</td>
</tr>
</table>

Study Design:	Single center Phase I clinical trial<br>Part I:<br>12 subjects will receive an open-label 500 µl subcutaneous injection via needle and syringe of the study IMP (CoVac-1). No more than one subject per day will be enrolled. 28 days following vaccination of the 12th volunteer, there will be an interim analysis of safety and a safety review by the data safety monitoring board (DSMB) as well as an amendment to the regulatory authorities (Paul-Ehrlich Institute and Ethics Committee) before proceeding to Part II.<br>Part II:<br>12 subjects will receive an open-label 500 µl subcutaneous injection via needle and syringe of the study IMP (CoVac-1). 28 days following vaccination of the 12th volunteer, there will be an interim analysis of safety and a safety review by the DSMB as well as a substantial amendment to the regulatory authorities (Paul-Ehrlich Institute and Ethics Committee) whether to proceed to next Part III.<br>Part III:<br>12 subjects will receive an open-label 500 µl subcutaneous injection via needle and syringe of the study IMP (CoVac-1).
Aim of the Study	To evaluate the safety and immunogenicity of a single use of a SARS-CoV-2-derived multi-peptide vaccine in combination with the TLR1/2 ligand XS15 in adults

Objectives/Endpoints	Primary endpoint:<br>• The nature, frequency, and severity of AEs and/or SAEs associated with administration of CoVac-1:<br>• Solicited: ADRs/AEs occurring from the time of each injection throughout 28 days following the procedure, facilitated by use of a volunteer diary<br>• Unsolicited: AEs from the time of injection throughout 56 days following injection<br>• SAEs from the time of injection until the final study visit for each subject<br>• Incidence of AESIs until the final study visit for each subject<br>Secondary endpoints:<br>• Development of a CoVac-1 specific T-cell response to at least one of the single SARS-CoV-2 T-cell epitopes included in the CoVac-1 vaccine on Visits 2, 3, 4, 5 measured by IFN-γ ELISpot ex vivo and after in vitro T-cell amplification (compared to Visit 1), this includes:<br>• Cellular conversion rate (CCR) at Visits 2, 3, 4, 5 after immunization

Explorative endpoints:

- Characteristics of T-cell response on Visits 2, 3, 4, 5 measured by ELISpot/ICS. This includes:

  - Phenotyping of SARS-CoV-2 specific T-cells (CD4, CD8 etc.) by flow cytometry

  - Characterization of cytokine profiles of SARS-CoV-2 specific T cells (TNF, IFN, IL-2, CD107a etc.) by intracellular cytokine staining

  - Recognition rate defined as percentage of peptides inducing a T cell response in one individual

  - Intensity of T cell response to a single SARS-CoV-2 T cell epitope included in the CoVac-1 vaccine

- Induction of long-term SARS-CoV-2 specific T-cell responses 3 and 6 months after peptide vaccination.
- Induction of antibodies specific to the SARS-CoV-2 T-cell epitopes included in the CoVac-1 vaccine

In case of unexpected detection of CoVac-1 specific antibodies the following assays will be performed:

- Individual neutralization antibody titers
- Seroconversion rates
- Calculation of geometric mean titers (GMT) for neutralizing and binding antibodies

- Biomarkers and clinical characteristics influencing immunogenicity.

Statistics, Safety Variables and Stopping Rules	**Safety**:<br>In this phase I study the safety/toxicity of one vaccination will be investigated. For this purpose, it will be investigated whether the incidence of severe adverse events (SAE) associated with administration of CoVac-1 exceeds a predetermined rate of 5% (= P1 = alternative hypothesis) in the whole study population. Safety of the CoVac-1 vaccine is shown if no SAE (= P0 = null hypothesis) occurs in the study population. An evaluable sample size of 33 achieves 81.6% power to detect a difference (P1-P0) of 0.0499 using a one-sided exact test based on the binomial distribution with a target significance level of 0.05. The actual significance level achieved by this test is 0.003. These results assume that the population proportion under the null hypotheses (P0) is 0.0001. Assuming a dropout rate of 7.5% (percentage of subjects that are expected to be lost at random during the course of the study and for whom no response data concerning existence of SAE will be collected, i.e. will be treated as “missing”) the total number of 36 subjects should be enrolled in the study in order to end up with 33 evaluable subjects. Sample size computed using PASS 2020 (NCSS, LLC, Kaysville, Utah, USA).<br><br>**Sample size:** 36<br>Part I:<br>n=12<br>Interim Safety Analysis after Part I and a substantial amendment to authorities<br>Part II:<br>n=12<br>Interim Safety Analysis after Part II<br>Part III:<br>n=12
Database	A validated GCP conform clinical trial database hosted by the IKEAB Tuebingen (SecuTrial) will be used for data capture and validation in this trial
Participating Centers and Investigators	CCU Translational Immunology, Department of Internal Medicine, University Hospital Tuebingen, ([redacted])
Study Type	• AMG

Competent Regulatory Authorities	• PEI and EC
Monitoring according GCP	Monitoring of the clinical trial will be performed by the ZKS Tuebingen.
Study duration	Total study duration for individual volunteer: 6 months<br>Safety duration for individual volunteer: 8 weeks<br>Follow up (exploratory end points) for individual volunteer:<br>4 months
Length of Study/ Time Lines	Total trial duration: 1 years<br>Duration for individual patient: Safety follow-up: 8 weeks<br>Follow-up: 4 months<br>Number of visits: 8<br>FSI (First Subject In): Q3/2020<br>LSI (Last Subject In): Q1/2021<br>LSO (Last Subject Out): Q3/2021<br>DBL (Data Base Lock): Q3/2021<br>Statistical Analyses Completed: Q4/2021<br>*Trial Report Completed: Q4/2021*

Table 1: Table of Events

**Protocol activities and forms to be completed**	**Screening**	**Vaccination phase[1]**					**Follow-up period[2]**
					**Interim Safety**	**EOSf**	
	**≤ - 7 days**	**Day 1**	**Day 7 +/- 1 days**	**Day 14 +/- 1 days**	**Day 28 +/- 2 days**	**Day 56 +/- 2 days**	**3 and 6 months after peptide vaccination**
Visit		**V1**	**V2**	**V3**	**V4**	**V5**	**V6-7**
Informed consent[3]	X						
Demographics[4]	X						
Medical history[5]	X						X
Signs/symptoms[6]		X	X	X	X	X	
Enrolment[7]	X						
	**Clinical assessments**						
Vital signs[8]	X	X	X	X	X		
Physical examination[9]	X	X	X	X	X		
Assessment of concomitant medications[10]	X	X	X	X	X	X	
AE assessments[11]		X	X	X	X	X	X
	**Laboratory assessments**						
Hematology (*local lab)*[12]	X	X	X	X	X	X	
Blood chemistry and coagulation (*local lab)*[13]	X	X	X	X	X	X	
Immunoglobulins/Immunophenotype[14]	X						
Urine analysis (*local lab)*[15]	X						
HBV, HCV, HIV-1, (*local lab)*[16]	X						
Pregnancy test[17]	X						
SARS-CoV-2 testing	X[18]						
	**Treatment**						
Vaccine CoVac-1[19]		X					
	**Efficacy assessment**						
T-cell response[20]		X	X	X	X	X	X
Serological response[21]		X	X	X	X	X	X

Detailed information on schedule and activities are described in the footnotes.

1. The peptide vaccination should be applied as early as possible after screening (max. 7 days) and approved eligibility of the volunteer. Vaccination phase will be 2 months and ends with the end of safety follow-up (EOSf).
2. Follow-up: After vaccination phase, volunteers will enter follow-up, which ends with the last visit 6 months after vaccination (V7, EOS).
3. Informed consent and volunteer registration: every volunteer must date and sign informed consent form to participate in this trial before starting any trial-related procedures.
4. Demographics: gender, year of birth, ethnicity
5. Medical history: The investigator has to collect information on the volunteers' medical history including prior illnesses, hospitalisations, and symptoms of a SARS-CoV-2 infection.
6. Signs/symptoms: vaccine-related and -unrelated signs and symptoms

7. Enrolment: volunteers are enrolled and registered through a screening procedure via ZKS Tuebingen.
8. Vital signs: At all visits: ECOG, temperature (in grade centigrade), blood pressure/pulse. At baseline additionally: height (in cm) and weight (in kg). At V4 and V5 additionally: weight (in kg). For detailed surveillance after vaccination, please refer to section 6.2 of the study protocol
9. Physical examination: inspection, abdominal, cardiac and lung auscultation, palpation of the abdomen and lymph node sites, neurological examination, inspection of vaccination site.
10. Concomitant medications should be reported in the respective CRF pages, including drugs used for treating AEs or, if applicable, chronic diseases.
11. AE assessments: events should be documented and recorded continuously. Volunteers have to be followed for AEs from application up to 56 days or until all drug-related toxicities have been resolved, whichever is later, or until the investigator assesses AEs as “chronic” or “stable”. Each AE must be reported indicating the CTC (Version 5.0) grade. If an event stops and later restarts or CTC grading changes, all occurrences must be reported. A specific procedure for definition and reporting of SAEs is described in the protocol.
12. Hematology (local lab): hemoglobin (Hb), red blood cells (RBC), platelet count (PLT) white blood cells (WBC). Differential cell counts should be performed at baseline, at each visit during vaccination phase and thereafter at investigators discretion. Clinical status and laboratory parameters are to be followed using individual institutional guidelines and the best clinical judgment of the responsible physician, which can involve more frequent testing.
13. Blood chemistry and coagulation (local lab): Alkaline phosphatase (AP), total bilirubin, aspartate transaminase (AST/ SGOT), alanine transaminase (ALT/ SGPT), lactate dehydrogenase (LDH), and uric acid, C-reactive protein (CRP), sodium, potassium, calcium, blood urea nitrogen, creatinine, glucose: at baseline and during vaccination phase, thereafter at each visit using individual institutional guidelines and the best clinical judgment of the responsible physician, which can involve more frequent testing. Prothrombin time, aPTT, and fibrinogen will be measured at baseline and at investigator’s discretion during treatment.
14. Immunoglobulin/immunophenotype: Assessment of IgA, IgG and IgM; lymphocyte subsets: T ($CD4^+$ and $CD8^+$) as well as B and NK cells.
15. Urine analysis (local lab): pH, glucose, proteins (qualitative, dipstick accepted): at baseline and at investigator’s discretion during treatment
16. HBV, HCV and HIV-1: at baseline and thereafter at investigator’s discretion
17. Pregnancy testing: For all FCBP, pregnancy testing has to be performed at the screening visit. Negative results must be available prior to vaccination.
18. SARS-CoV-2 testing: Volunteer must be tested for prior or current SARS-CoV-2 infection. Patients should be tested by serological test and throat/nose swab. If screening takes more than 48 hours, throat/nose swab for SARS-CoV-2 infection must be repeated. The vaccine can only be applied if a negative SARS-CoV-2 PCR test is available on the day of vaccination not older than 48 hours.If patients develop SARS-CoV-2 typical symptoms until vaccination, testing should be repeated.
19. Vaccine CoVac-1: Peptide vaccination should be started as soon as possible after the screening visit. Peptide vaccination will be performed once.
20. T-cell response: 60 ml of heparin blood for immunomonitoring and analysis of peptide specific T-cell response will be analyzed by the Walz lab, KKE Translational Immunologie at the Department of Immunology, Tuebingen (central laboratory). Blood will be taken before peptide vaccination on V1, and during vaccination phase and follow-up at each visit.
21. Serological response: 10 ml of serum for analysis of serological response will be analysed by the Department of Immunology, Tuebingen (central laboratory). Blood will be taken before peptide vaccination on V1, and during vaccination phase and follow-up at each visit.

1. Introduction

The novel coronavirus SARS-CoV-2 causes the COVID-19 disease, which especially in elderly, weakened and immunocompromised patients, shows severe and fatal courses.[1-3] In the meantime, SARS-CoV-2 has spread to a worldwide pandemic with yet incalculable medical, economic and socio-political consequences. So far, there are no established therapies and a vaccine is not yet available.

Deaths and serious illness are more common in the older population over 60 years of age.[4] Outbreaks in long-term care facilities have been observed in several countries, which pose particular challenges in terms of containment and isolation within the facility, affecting and threatening those most at risk. For patients over 65 years of age with SARS-CoV-2 infection, a high hospitalization rate of between 28.6% and 43.5% in the age group 65-74 years and between 30.5% and 58.7% in the age group 75-84 years has been described, with an associated high mortality rate of up to 30%.[4]

There are two promising options for reducing the number of severe COVID-19 disease cases in elderly and comorbid people in the future:

- The development of preemptive measures (vaccination) that prevent the disease or reduce its progression.
- A therapeutic intervention in early stages of the disease, especially in the group of ≥ 65-year-olds with the highest risk of a severe course of the disease.

Both approaches can prevent deterioration in disease course, reduce the frequency of hospital admissions and intensive care treatment and thus take the pressure off the health care system.

T-cell based immunity

T-cell immunity plays an essential role in the control of viral infections. $CD4^+$ T-helper cells (Th1) are essential for the regulation and maintenance of the immune response and for the production of antiviral cytokines, while cytotoxic $CD8^+$ T-cells (CTL) are responsible for the elimination of virus-infected cells. The recognition of viral antigens, which are presented as short peptides via the human leukocyte antigen system (HLA), is essential for the activation and function of T cells. To identify and analyze protective T-cell immune responses against viral infections in the human population, a comprehensive identification and characterization of such viral T-cell epitopes is necessary.[5] [6] This knowledge is not only essential for understanding the host's immune response and the mechanisms of long-term protection in case of virus recurrence, but also a prerequisite for the development of new and more efficient therapeutic and preventive immunotherapy approaches. Besides the generation of virus-specific T-cells *ex vivo* with subsequent transfer into the patient,[7-11] the possibility of

direct vaccination with T-cell epitopes for the induction of a T-cell response directly *in vivo* is of particular importance. Such vaccines can be used to generate immune responses against the SARS-CoV-2 without enduring COVID-19 disease. Furthermore, they can also be used therapeutically to prevent severe courses of disease in acute SARS-CoV-2 infected patients by accelerating/generating a virus-specific T-cell response and activating *in vivo* virus-specific B-cells supporting antibody production.

The findings and experience with two other zoonotic coronaviruses - SARS-CoV-1 and MERS-CoV - based on the detection of CoV-specific $CD8^+$ and long-lasting $CD4^+$ memory T-cell responses in convalescents provide evidence that T-cell immunity also plays an important role in the control of coronavirus infections.[12-15] This is even more important since studies on humoral immunity to SARS-CoV-1 provided evidence that antibody responses are short-lived and can even cause or aggravate virus-associated lung pathology.[16 17] For $CD8^+$ and Th1 $CD4^+$ T cells in contrast a crucial role in viral clearance and protection against the deadly SARS-CoV-1 infection was reported especially in terms of reported lung pathology.[12 14 15] Numerous $CD4^+$ and $CD8^+$ T-cell epitopes have been described for SARS-CoV-1 and MERS-CoV, which, due to the sequence homology of the two coronaviruses, suggest potential cross-reactivity and could also be potential T-cell epitopes for the new SARS-CoV-2 virus.[18] With regard to SARS-CoV-2, two very recent studies[19 20] described $CD4^+$ and $CD8^+$ T-cell responses against viral peptide pools in donors that had recovered from COVID-19 as well as individuals not exposed to SARS-CoV-2, indicative of potential T-cell cross-reactivity.[21-23] In own preliminary work , we define SARS-CoV-2-specific and cross-reactive $CD4^+$ and $CD8^+$ T-cell epitopes in a large collection of SARS-CoV-2 convalescents as well as non-exposed individuals and confirmed their relevance for immunity and the course of COVID-19 disease.[24] These SARS-CoV-2 T-cell epitopes show high recognition frequencies in convalescents from SARS-CoV-2 infection, suggesting their important role in the natural course and immune control of COVID-19. These T-cell epitopes represent the basis for the vaccine peptides included in the CoVac-1 vaccine.

SARS-CoV-2 peptide vaccine

The aim of this study is to investigate the safety and immunogenicity of a peptide vaccine consisting of SARS-CoV-2 specific HLA class II peptides in volunteers without prior or current SARS-CoV-2 infection.

The identification and characterization of T-cell epitopes is a long-standing and unparalleled expertise of the Department of Immunology.[25-27] This unique approach is based on i) the prediction of HLA binding sequences for HLA class I and class II alleles using the world's first prediction tool (www.syfpeithi.de[28]) and newer, more refined methods, all based on SYFPEITHI, ii) the identification of naturally presented HLA class I and class II ligands

(immunopeptidomics), iii) the synthesis of synthetic peptides, and iv) the characterization of T-cell epitopes and peptide-specific $CD4^+$ and $CD8^+$ T cell responses. This strategy has been successfully applied in recent years to define and characterize T-cell epitopes derived from various viruses such as CMV, EBV, ADV and influenza as well as tumor-associated antigens of various solid and hematological malignancies [29-33].

Based on this work, the results were translated into therapeutic vaccination and T-cell transfer studies in cancer patients (e.g. NCT02802943) and viral infections[34 35]. This direct translation is made possible by the Wirkstoffpeptidlabor ([redacted]) of the Department of Immunology and the GMP facility for individualized drugs at the University Hospital Tuebingen as well as our immune monitoring platform equipped with state-of-the-art, validated T-cell assays and methods.

The existing experience and logistics can be directly used for the treatment and prevention of COVID-19 disease. In preliminary work for this study, $CD4^+$ T cell epitopes have already been characterized in a large cohort of SARS-CoV-2 infected donors validating their high relevance in the natural course of COVID-19. The vaccination cocktail in the study will consist of seven promiscuous HLA class II peptides from the different proteins of the SARS-CoV-2 virus, predicted to bind to several HLA class II allotypes. Furthermore, especially those peptides were selected that contain embedded HLA class I sequences in order to induce $CD4^+$ T cell responses and $CD8^+$ T cell responses simultaneously. Furthermore, especially for peptides derived from virus surface proteins, only sequences were selected that do not represent antibody epitopes (not accessible to antibodies due to the predicted 3D structure of the protein; for more detail see IB section 4.2.6). This should prevent the formation of antibodies against the vaccinated peptides, which could possibly have a deteriorative effect on COVID-19. Immunogenicity was proven for all HLA class II peptides included in the peptide cocktail in a large cohort of SARS-CoV-2 convalescent donors as well as for single peptides in a first vaccination of a healthy volunteer (for more detail see IB section 4.2.3).

<u>Adjuvants</u>

A further prerequisite for successful peptide vaccination, besides selection of optimal antigen targets, is the use of a suitable adjuvant, which is able to induce potent and long-lasting immune responses. Among the most effective approaches tested in humans is the subcutaneous injection of peptides emulsified in Montanide ISA 51 VG, a water-in-oil-emulsion, combined with the TLR9 ligand CpG.[36] However, CpG is not available for clinical trials, and a peptide/antigen vaccine emulsified in Montanide without any additional adjuvant induces no or only weak immune responses[37]. In the P-pVac-SARS-CoV-2 trial,

the novel TLR1/2 ligand XS15 emulsified in Montanide ISA 51 VG will be employed as adjuvant, applied subcutaneously together with the peptide vaccine. XS15 is a water-soluble derivative of the TLR1/2 ligand Pam_3Cys and induced a strong $CD8^+$ and Th1$CD4^+$ T-cell response against free short peptides in Montanide ISA 51 VG after a single s.c. injection in a healthy volunteer as well as in cancer patients.[38] Immune responses could be induced against viral peptides (including SARS-CoV-2 derived peptides), neoepitopes derived from cancer-specific mutations as well as tumor-associated self-peptides. XS15 results in granuloma formation on the vaccination site, where the vaccinated peptides persist for at least 7 weeks. Peptide-specific T cells were detected at the granuloma site, however, with a lower frequency than in peripheral blood, which rules out the risk of T-cell sequestration, dysfunction or deletion at the vaccination site due to the use of XS15 in Montanide ISA 51 VG. Strikingly, the induced immune responses were found to persist for more than 1.5 years.

With regard to the planned study we could also show that this vaccination method is able to induce potent SARS-CoV-2 specific T-cell responses in a human volunteer (for more detail see IB of XS15 (1.0. 27 May 2020)).

1.1. Trial Rationale and Justification

1.1.1. Mechanism of action and rational for a prophylactic SARS-CoV-2 multi-peptide vaccine

The CoVac-1 vaccine evaluated in the P-pVAC-SARS-CoV-2 study is based on multiple HLA-DR SARS-CoV-2 T-cell epitopes and aims to induce SARS-CoV-2 specific T-cells in the vaccinated donors. Antibodies other than IgM are only produced if T cell help is provided to the B cells. Therefore the rationale of the T-cell inducing CoVac-1 vaccine described here is to induce T-helper cells first, before infection and thus before B cells have first contact to the viral antigen. If the B cells then see antigen after infection, they will present the antigens(s) recognized on their HLA class II molecules, and immediately will receive help from the preactivated and expanded vaccine induced T cells. During natural infection, it would take several days for the T cells to get activated and sufficiently expanded. Thus, the production of antibodies, in particular of IgG and IgA classes, should occur much faster in the vaccinated individuals, so that the virus can be cleared faster. Of special note is here that older individuals have lower numbers of T cells, in particular $CD4^+$ T cells [39 40]. Thus, virus antigen specific $CD4^+$ T cells already preactivated and expanded at the time of infection should be especially benefitting for older individuals. Multiple studies in animal models have

clearly demonstrated the requirement of CD4$^+$ T cell help for the generation of protective antibody responses (for example, influenza[41], malaria[42 43], vaccinia[44 45]). Recent studies have also demonstrated that the role of CD4$^+$ T cells in the immune response to viral infections is not limited to help for antibody production; CD4$^+$ T cells are also required to generate optimal CD8$^+$ T cell responses[46-49]. Moreover, CD4$^+$ T cells additionally can act as effector cells by the secretion of cytokines and direct killing of infected cells[50-54]. HLA class II antigens specifically activate CD4$^+$ helper T cells, therefore the CoVac-1 vaccine based on SARS-CoV-2-derived HLA class II peptides will enable a potent cellular and humoral immune response to SARS-CoV-2 preventing severe courses of COVID-19.

The development of a multi-peptide vaccine focusing on the induction of SARS-CoV-2 specific T-cell responses is further supported by several recent publications describing a decrease in neutralizing SARS-CoV-2 antibodies in COVID-19 convalescents after two to four month[55 56]. In contrast a recent study still detected SARS-CoV-1 specific T-cell 17 years after infection suggesting that in contrast to antibodies T cells might enable a long lasting immunity to SARS-CoV-2. In own preclinical data we could further detect SARS-CoV-2 specific T-cell against the T-cell epitopes in the CoVac-1 vaccine in donors after COVID-19 infection even if no antibody responses could be detected. Furthermore, we could show that donors with a high diversity of T-cell responses to SARS-CoV-2 T-cell epitopes in terms of numbers of epitopes detected by a donors was associated with milder symptoms of COVID-19[24].

1.1.2. Rational for the usage of XS15 as adjuvant in the prophylactic SARS-CoV-2 multi-peptide vaccine

Beside the selection of optimal antigen targets, a further important prerequisite is the use of suitable adjuvant drugs able to induce potent and long-lasting immune responses. In this clinical study, we will use for the first time the novel TLR1/2 ligand XS15 (emulsified in Montanide ISA 51 VG) which 1) is water-soluble and 2) GMP-amenable, 3) non-toxic and 4) effective in inducing T cell responses *in vivo.* The active molecular component in XS15 is Pam3Cys. This is a natural substance component found in bacteria and as such has already been used in a borreliosis vaccine (Limerix) approved in the USA in over 20,000 healthy people[57 58]. Pam3Cys was covalent with a protein compound (Surface protein A (OspA) from B. burgdorferi). In experimental peptide vaccines, Pam3Cys-peptide conjugates proved to be very efficient, but such molecules are unsuitable for pharmaceutical development, especially for personalized multi-peptide vaccines, as validation of a drug produced from them would be very costly or impossible. For this reason, the water-soluble Pam3Cys derivative XS15 was

developed. This derivative has a comparable effect to the above mentioned conjugates in vitro, but is more suitable for pharmaceutical development, because it is water soluble, easily purified by HPLC and detectable by mass spectrometry. Combined with Montanide ISA 51 VG and peptides, XS15 induces efficient T-cell responses after a single injection. This is especially important for its use in prophylactic viral vaccines, as immunization of large cohorts requires highly efficient immunity induction with the lowest number of vaccinations possible. Thus, Montanide/XS15 can be considered as a GMP-amenable version of the well known Complete Freund's Adjuvans [59 60] and therefore represents the optimal adjuvant for the P-pVAC-SARS-CoV-2 study.
Based on animal toxicity data and preliminary evidence (self-administration of vaccines and information gained through administration of XS15 adjuvanted vaccines as an unproven intervention, according to physicians judgement and with informed consent, in keeping with principle 37 of the Declaration of Helsinki), we assume that a dosage of 50 µg XS15 (total dosage) administered as a vaccine together with Montanide ISA 51 VG and synthetic peptides can be considered as a safe and potentially effective strategy (for more detail see IB of XS15 (1.0. 27 May 2020)).[38 61]

1.1.3. Rational for selected doses

1.1.3.1. Dose rational for peptides

Previous vaccination trials were performed at peptide doses ranging from 10 to 5,000 µg per vaccination: Even though only a few of these trials included a dose finding element, there is a tendency that doses below 100 µg are not effective to induce T-cell responses whilst doses above 500 µg do not seem to generate an increasing immunogenicity. Dose-finding studies performed with viral protein-derived epitopes showed significantly stronger immune responses in the 300-500 µg range versus the 100 µg dose, without significantly higher immune responses in the 1,000 vs. 500 µg group[62]. This is supported by own data of the investigator and the Immatics Biotechnologies GmbH[63] (for more details refer to the IB of CoVac-1).Preliminary data from a healthy volunteer and cancer patients vaccinated with a personalized peptide vaccine (240-300 µg per peptide) including two of the CoVac-1 peptides (240µg) in combination with XS15 showed potent induction of T-cell responses in 100% of HV and patients and a good safety profile. Concerning safety of peptide vaccines in different doses no severe side effects were observed even with very high doses of peptides up to 30mg[64,65].

Furthermore, a similar multi-peptide vaccination study for influenza evaluated safety and immunogenicity with two doses of peptides (250µg and 500µg). No difference in the safety profile was detected for the two different doses and significant induction of functional T-cell responses were observed for both peptide doses, suggesting the dose of 250µg sufficient and safe for a prophylactic viral peptide vaccine[66].

The dose of ~240 µg per peptide per dose for CoVac-1 vaccine was selected based on these findings and on the feasibility in pharmaceutical development of the vaccines.

1.1.3.2. Dose rational for XS15

The molecular mode of action of both the Pam3Cys conjugates and XS15 is an activation of immune cells via the toll-like receptor TLR1/2. These immune cells are mainly found in the blood and lymphoid tissues. Desired as well as toxic effects are therefore to be expected above all and presumably exclusively due to the XS15-TLR1/2 interaction with these cells, in particular through an over activation of these cells, which could then lead to a so-called cytokine release syndrome. The dose of XS15 is based on an in vitro assay that investigated both potential toxicity as well as efficiency. In these assay 10 µg/ml XS15 was shown to be the most efficient dose for the stimulation of immune cells (for more details please refer to the IB of XS15). The following considerations regarding the concentration of XS15 after a subcutaneous administration are the basis of dose finding: When used with Montanide ISA 51 VG in a total volume of 500 µl suspension, a granuloma forms rapidly at the injection site, which has a volume of estimated 2 ml. This granuloma further increases up to 8ml on day 17 after vaccination[38]. Thus, the initial local concentration of XS15 is maximally 50 µg/ml which is reduced soon thereafter to 25 µg/ml (50µg in 2 ml) and soon thereafter is diluted even more, since the granuloma increases more, so that a concentration of 10 microgram/ml will soon be reached. Further dilution will follow with the granuloma increase to 6,25mg/ml (50 µg in 8ml). Based on this in vitro experiments and considerations the dose of 50 µg was selected for further in vitro and in vivo toxicity evaluation as well as for first in vivo vaccination experiments.

In the toxicity study of mice, a dose of 50 µg XS15 in Montanide, applied locally s.c., did not reveal any toxicity beyond the long known and expected toxicity of Montanide alone. Therefore, this study proves that XS15 has no local and above all no systemic toxicity under this application method up to the above mentioned dose (for more details please refer to the IB of XS15). Furthermore, considering systemic toxicity of XS15 50µg after s.c. injection the following considerations were made: If this dose (in the absence of Montanide ISA 51 VG) is immediately distributed in the blood (6l), a maximum blood concentration of 0.008 µg/ml

would be expected. At a concentration of 0.008 µg/ml no measurable reaction (stimulation of immune cells) is detected in the above described in vitro test.

When used with Montanide, the formation of a granuloma at the injection site, which has a depot effect for peptides, means that a gradual release of these peptides or XS15 into the blood can be expected. Therefore, the actual blood concentration of XS15 after administration of 50 µg in a Montanide/water emulsion is likely to be much lower than the maximum concentration of 0.008 µg/ml described above. Therefore, a systemic toxic effect of XS15 is not expected at a dose of 50 µg s.c. with or without Montanide.

1.1.3.3. Dose rationale for Montanide ISA 51 VG

Montanide™ ISA 51 VG has been used in about 300 clinical trials from phase I to phase III which represents more than 19 000 vaccines. In addition, Montanide™ ISA 51 VG has been approved in a commercial vaccine against non-small cell lung cancer (NSCLC).

Dosing of 0,25ml after 50/50 mixture with peptides is based on two published clinical studies evaluating influenza vaccines in more than 2500 donors showing high immunogenicity and a good safety profile[67] [68]. Detailed information on preclinical and clinical safety data for Montanide ISA 51 VG could be found in the respective IB as well as in the attached “Human application form for Montanide ISA 51 VG”.

1.1.3.4. Rationale for one dose schedule

The combination of multi-peptide vaccine with the TLR1/2 ligand XS15 emulsified in Montanide ISA 51 VG with the above described dosing was already evaluated in a healthy volunteer as well as in cancer patients (n=12). Multi-peptide vaccines included beside tumor-associated neoepitopes and self peptides also viral T-cell epitopes derived from CMV and SARS-CoV-2. In all vaccinated individuals peptide-specific T-cell responses could be detected after one single vaccination. For viral T-cell epitopes including SARS-CoV-2 derived peptides strong T-cell responses could even be detected ex vivo without in vitro amplification of T-cells after one single vaccination. Immune responses after vaccination were shown to last for more than 1,5 years so far. Furthermore, the safety profile of these vaccines with similar composition and dosing as for the CoVac-1 vaccine was very good after a single vaccination, showing only grade 1 local reaction at vaccination side after single injection. Therefore, the first-in-man evaluation of CoVac-1 with a single vaccination seems reasonable to enable efficient induction of immune response with the lowest possible number of vaccination and side effects. Please find below a detailed description of the data from in vivo

administration of peptide vaccines in similar composition in a healthy volunteer and cancer patients (for more details please refer to the IB of CoVac-1).

1.1.4. Rational for trial design

This is a phase I multi-peptide vaccination study using SARS-CoV-2 HLA-DR peptides in combination with the novel TLR1/2 ligand XS15 in healthy volunteers to prove safety and immunogenicity. The primary objective is incidence and severity of AEs (≥ Grade 4) after vaccination in the observational time (until day 28). Furthermore, the trial aims to expand experience on overall safety and immunogenicity in the study cohort.
This is based on the following rationale:

The SARS-CoV 2 pandemic is currently one of the major threats to the world population and requires the rapid development of effective preventive and therapeutic tools. $CD4^+$ and $CD8^+$ T-cells, as comparts of the adaptive immune system, are an important cornerstone in the control of viral infections. As state above, T-cell immunity seems to play a significant role in corona virus infections including SARS-CoV-2 and has a major impact on the course of disease including severe lung pathology as observed in COVID-19. The induction of SARS-CoV-2 specific T-cell responses therefore might represent a valuable preventive and therapeutic tool especially in the group of elderly and comorbid patients to prevent severe courses of SARS-CoV-2 infection. SARS-CoV-2 specific T-cell immunity can be achieved by peptide vaccination applying SARS-CoV-2 specific promiscuous HLA class II T-cell epitopes. The HLA class II epitopes were selected based on the immunogenicity in a cohort of SARS-CoV-2 convalescent donors, proving their pathophysiological relevance in COVID-19.[24]

In view of the pandemic spread of COVID-19, health care systems face major challenges, as a large number of patients require hospital treatment and intensive care. As soon as the capacities of individual health care systems are exceeded, optimized care for all can no longer be guaranteed.
Containment strategies in Germany include the quarantine of infected persons and the 14-day quarantine of contact persons (incubation period). At the population level, most affected countries have reduced contacts through various measures such as closing schools, shops, restaurants and, in extreme cases, a total curfew. Without effective treatment options for COVID-19 and a vaccine available for the broad population, these measures can not be terminated, which results in immense economic and socio-political damage. This

underscores the high need for the development of novel treatment approaches to prevent a severe disease course of SARS-CoV-2 infection.

Therefore this trial has been conceptualized to prove safety and immunogenicity of a peptide vaccine against SARS-CoV-2. The focus in the study population is set to older participants. This is of special interest as these people are considered to be at high risk for severe disease and society has to protect the elderly. Vaccination will be conducted in three different healthy volunteer cohorts (Part I-III), each followed by an interim safety analysis before proceeding:

- Part I: Healthy adult aged 18-55 years
- Part II: Adults aged 56-74 Part III: Adults aged ≥ 75 After proving safety and immunogenicity in a cohort of healthy volunteers aged 18-55 (Part I), an interim safety analysis will be conducted and prior to continuation with Part II approval by DSMB and of an amendment by PEI and Ethics Committee must be obtained. Again, after completion of the Part II with volunteers aged 56-74, approval from DSMB will be obtained, before recruiting volunteers aged ≥75 at high risk for serve course of SARS-CoV-2 infection in the vaccination trial.

1.2. Benefit / Risk Assessment

The assumed clinical benefit and risk of P-pVAC-SARS-CoV-2 vaccination are based on the following aspects:

- Peptide vaccination using HLA-presented peptides represents an established immunotherapy approach utilized for preventive vaccine development in infectious disease[69 70] as well as for therapeutic approaches in malignant disease. Several peptide vaccination studies in patients with malignant disease including solid tumors[63 71-73] and hematological malignancies[74-77] have proven safety and tolerability of this approach.
- Multi-peptide vaccination represents a low side-effect immunotherapy approach relying on specific immune recognition of HLA-presented peptides[78-80].
- The Wirkstoffpeptidlabor holds certificates for the production of GMP grade synthetic peptides and for the formulation of multi-peptide vaccine cocktails including the TLR1/2 ligand XS15, which allows for a rapid GMP production of the CoVac-1 vaccine. This is of great importance due to the serious threat the SARS-CoV-2 pandemic currently poses to the world population.
- All peptides included in the CoVac-1 vaccine are proven SARS-CoV-2 T-cell epitopes with pathophysiological relevance in the natural course of COVID-19 disease
- CoVac-1 peptide vaccination can induce potent $CD8^+$ and $CD4^+$Th1 T-cell responses

against SARS-CoV-2 providing immunity against infection as:

- $CD4^{+}$Th1 cells will directly contribute to virus clearance and deliver strong T helper signals to $CD8^{+}$ T cells primed during natural infection. Furthermore, these SARS-CoV-2 specific $CD4^{+}$Th1 cells can activate virus antigen-experienced B cells. The resulting enhanced activity could lead to more rapid virus clearance and prevention of a severe course of COVID-19 disease.
- Vaccine peptides contain embedded CD8 T-cell epitopes predicted to bind to many HLA class I allotypes. Such $CD8^{+}$ T cells should also contribute to faster virus clearance.

* Since we found IFNγ-producing SARS-Cov-2 specific T-cells in a healthy volunteer vaccinated with SARS-CoV-2 T-cell epitopes, it is very likely that significantly $CD4^{+}$Th1 T cells are induced by the vaccine. There should be thus no disease enhancing-effect due induction of Th2-bias as described for other corona viruses[81].
* As development of antibody-dependent enhancement (ADE) has been identified as potential risk[82] for infected patients after vaccination approaches, the following considerations and risk mitigation strategies have been undertaken:
* In contrast to other classical vaccines aiming to induce an antibody response to prevent viral infections, the CoVac-1 vaccine is designed to induce SARS-CoV-2 specific T-cells. According to experience from comparable peptide vaccines in cancer patients it is very unlikely, that such antibodies will be induced after a single vaccination. Induction of antibodies against vaccine peptides were observed in cancer patients with delay, and only after several vaccinations. So far, no antibody induction against the T-cell epitopes included in the CoVac-1 vaccine was observed.
* Furthermore and most importantly, even in the unlikely event of antibody induction against CoVac-1 vaccine peptides, which will be monitored during the study as outlined in the protocol (section 6.3.2), these antibodies cannot recognize viral particles, because none of the vaccine peptides is exposed on the virus particle surface. Thus, neither neutralizing nor ADE-inducing antibodies can be induced by the vaccine. In contrast to ADE mediated by vaccine induced antibodies, which as describe above is extremely unlikely with the CoVac-1 vaccine, there might be a risk of ADE in cases of SARS-CoV-2 infection in which the patient's B cells have already been primed against epitopes of common cold seasonal human coronavirus strains and produce low amounts of antibodies, antibodies with low affinity or antibodies with the wrong affinity. In theory, vaccine-induced $CD4^{+}$ T-cells might cause or exacerbate immune pathological effects indirectly. As such *in vivo* effects can not be preliminary assessed in an in vitro setting, symptoms attributable to SARS-CoV-2 infection will

results in subsequent PCR testing and proven SARS-CoV-2 infection will be reported as AEs of special interest (AESI). These AESIs will be monitored particularly carefully including early hospital admission of patients with COVID-19 after CoVac-1 vaccination. This was outlined in more detail in the study protocol.

- Participant selection is based on medical care and safety considerations:
    - The trial comprises three parts (cohorts of participants) with different age ranges to provide preliminary results on safety in a cohort of young (18-55 years, n=12) and healthy participants, which is then extended to older (Part II and Part III) participants. Of note, the risk of vaccine related (S)AEs is hypothesized to be similar in each age group.
    - The design addresses the urgent medical need for protection of people at risk for serve SARS-CoV-2 infection by providing safety and immunogenicity data as well as first efficacy data in terms of SARS-CoV-2 infection in this population.
    - After Part I of the clinical trial (last patient has completed V4) a substantial amendment is send to the regulatory authorities besides seeking advice from the DSMB.
    - Safety is continuously monitored by an independent DSMB, which will be provided with reports on a regular basis (see DSMB Charter).
- Successful development of a peptide vaccine will help to put an end to quarantine and fear of SARS-CoV-2.
- Confirming safety of the CoVac-1 vaccine in healthy volunteers within the P-pVAC-SARS-CoV-2 study will further allow the transfer of this approach to induce SARS-CoV-2 specific T-cell immunity in a therapeutic setting for patients with SARS-CoV-2 infection.

The assumed clinical benefit and risks of peptide vaccination in combination with the TLR1/2 ligand XS15 in Montanide ISA 51 VG are based on the following aspects:

- Peptide vaccination alone is rarely able to induce clinically effective T-cell responses; thus the peptide vaccine has to be combined with an adjuvant drug to enhance immune responses.
- Several TLR ligands have been shown to potently induce $CD8^+$/$Th1CD4^+$ responses in humans, including CPG (TLR9 ligand), imiquimod (TLR7 ligand) and poly-IC (TLR3 ligand). However, no GMP compliant substance based on these TLR ligands is available that can be applied with a peptide vaccine.

- XS15 is a water-soluble derivative of the TLR1/2 ligand Pam3Cys and induces a strong $CD8^+$ and $Th1CD4^+$ T-cell response against free short peptides emulsified in Montanide ISA 51 VG after a single s.c. injection in healthy volunteers as well as cancer patients.
- Using XS15, immune responses could be induced for viral peptides (including SARS-CoV-2 derived peptides), neoepitopes from cancer-specific mutations as well as for tumor-associated self-peptides.
- XS15 results in granuloma formation on the vaccination site, where the vaccinated peptides persist for at least 7 weeks, which supports the induction of a strong immune response.
- The induced immune responses observed so far persisted for more than 1.5 years.
- Beside formation of granuloma locally on injection side, no relevant side effects of peptide vaccination in combination with XS15 in Montanide ISA 51 VG were observed in a healthy volunteer and cancer patients. In particular, no allergic or anaphylactic reactions or cytokine release syndrome have been observed (detailed information can be found in the IB V1.0 and the IB of XS15 (1.0. 27 May 2020)).
- Montanide ISA 51 VG is an oil adjuvant suitable for human injection that allows the manufacturing of water in oil emulsions. Montanide ISA 51 VG has been used in more than 200 clinical trials including more than 6000 patients. Most common side effects are injection site reactions (68%) including granuloma development, fatigue (54%), fever (41%), gastrointestinal disorders (32%) and injection site or local erythema (28%)[83]. In general, the observed adverse from controlled trials with non-healthy as well as healthy individuals were mild to moderate in intensity.

Conclusion

Taking into account the lack of effective treatment options and the dismal prognosis in SARS-CoV-2 infected high risk patient populations, especially in comorbid patients aged > 65 years, the expected benefits of a SARS-CoV-2 specific HLA class II peptide vaccination in combination with XS15 emulsified Montanide ISA 51 VG are considered to outweigh the potential risks for the participants, especially since multiple risk mitigation (e.g. interim safety analysis) measures have been incorporated.

1.3. Data and Safety Monitoring Board (DSMB):

An independent Data and Safety Monitoring Board (DSMB) will be assembled. The DSMB will be composed of independent experts in the field of immunology and infectiology assessing the progress, safety data and critical efficacy endpoints. The mission of the DSMB

is to ensure the ethical conduct of the trial and to protect the safety interests of participants in this trial.

The DSMB will receive a report listing and summarizing all the relevant safety data at least twice. The first assessment (first interim safety report, section 9.5) will take place after Part I of the trial including DSMB approval and an amendment at the regulatory authorities (Paul-Ehrlich Institute, PEI) and Ethics Committee (EC). If the IMP is considered safe for continuation by DSMB, Part II of the trial will start recruiting. After completion of Part II, the second DSMB report (second interim safety report, section 9.5) will be created and the DSMB has to approve continuation again. This report will be made available for EC. In addition, the report will provide data concerning recruiting rates, status of the trial and AESIs (section 9.1.4); also non-occurrence will be mentioned. Based on its review, the DSMB will provide the sponsor with recommendations regarding trial modification and continuation or termination of the trial. An emergency meeting of the DSMB may be called at any time should questions of volunteer safety arise or holding rules apply, and necessary safety reports will be provided. Meetings may be convened as conference calls/e-mail as well as in person.

2. Study Objectives

2.1. Primary Objective and Endpoint

The primary objective of this trial is to evaluate the safety and tolerability of the CoVac-1 vaccine, a single dose SARS-CoV-2 specific multi-peptide vaccine combined with the TLR1/2 ligand XS15 emulsified in Montanide ISA 51 VG in adults.

2.1.1. Primary Endpoint

The nature, frequency, and severity of AEs and/or SAEs associated with administration of CoVac-1:

- Solicited: ADRs/AEs occurring from the time of each injection throughout 28 days following the procedure, facilitated by use of a volunteer diary
- Unsolicited: AEs from the time of injection throughout 56 days following injection
- SAEs from the time of injection until the final study visit for each subject
- Incidence of AESIs until the final study visit for each subject

2.2. Secondary Objectives and Endpoints

Secondary objectives of this trial are to evaluate the efficacy of the CoVac-1 vaccine in terms of induction of SARS-CoV-2 specific T-cells.

2.2.1. Secondary Endpoints

- Development of a CoVac-1 specific T-cell response to at least one of the single SARS-CoV-2 T-cell epitopes included in the CoVac-1 vaccine on Visits 2, 3, 4, 5 measured by IFN-γ ELISpot ex vivo and after in vitro T-cell amplification (compared to Visit 1), this includes:
    - Cellular conversion rate (CCR) at Visits 2, 3, 4, 5 after immunization

2.3. Exploratory Objectives and Endpoints

Explorative objectives are the duration and characteristics of T-cell responses and the analysis of induction of antibody responses to single SARS-CoV-2 T-cell epitopes included in the CoVac-1 vaccine.

2.3.1. Exploratory Endpoints

- Characteristics of T-cell response on Visits 2, 3, 4, 5 measured by ELISpot/ICS. This includes:
  - Phenotyping of SARS-CoV-2 specific T-cells (CD4, CD8 etc.) by flow cytometry
  - Characterization of cytokine profiles of SARS-CoV-2 specific T cells (TNF, IFN, IL-2, CD107a etc.) by intracellular cytokine staining
  - Recognition rate defined as percentage of peptides inducing a T cell response in one individual
  - Intensity of T cell response to a single SARS-CoV-2 T cell epitope included in the CoVac-1 vaccine
- Induction of long-term SARS-CoV-2 specific T-cell responses 3 and 6 months after peptide vaccination.
- Induction of antibodies specific to the SARS-CoV-2 T-cell epitopes included in the CoVac-1 vaccine measured by ELISA. In case of unexpected detection of CoVac-1 specific antibodies the following assays will be performed:
  - Individual neutralization antibody titers
  - Seroconversion rates
  - Calculation of geometric mean titers (GMT) for neutralizing and binding antibodies
- Biomarkers and clinical characteristics influencing immunogenicity.

3. Study Design

This is an interventional, open-label, phase I trial evaluating the CoVac-1 vaccine, a single dose SARS-CoV-2 specific multi-peptide vaccine combined with the TLR1/2 ligand XS15 emulsified in Montanide ISA 51 VG in adults. The study is divided into three parts, which will recruit consecutively. Prior to initiation of the next part, the previous part must have completed recruiting, and day 28 of the last patient enrolled must have passed. After interim safety analysis and approval from the authorities (section 9.5), the next study part starts recruiting (Figure 1 and 2).

The first volunteer included in the trial will be hospitalized after vaccination and closely monitored. This patient is observed until day 28 and possibly arising safety issues are reported to and decided on by the Sponsor. Thereafter, no more than one subject per day will be treated/vaccinated. 28 days following vaccination of the 12th volunteer, there will be an interim analysis of safety and a safety review by the data safety monitoring board (DSMB) as well as a substantial amendment to the regulatory authorities (PEI and EC) before proceeding to Part II. Part II and III must not start recruiting prior to approval by authorities. Volunteers of part II are treated simultaneously and 28 days following vaccination of the 12th volunteer, there will be an interim analysis of safety and a safety review by the DSMB whether to proceed to next Part III. Volunteers of part III are treated simultaneously (2 participants per day). Details can be found in figure 3.

To avoid bias in treatment, a manualized process protocol as well as monitoring and treatment reports are implemented. The volunteer selection will be documented. Reasons for refusal will be assessed. To avoid bias in data analysis, monitoring and analysis by intention-to-treat are planned. Data analysis will be conducted by an independent statistician.

Figure 1: Overall Study Design

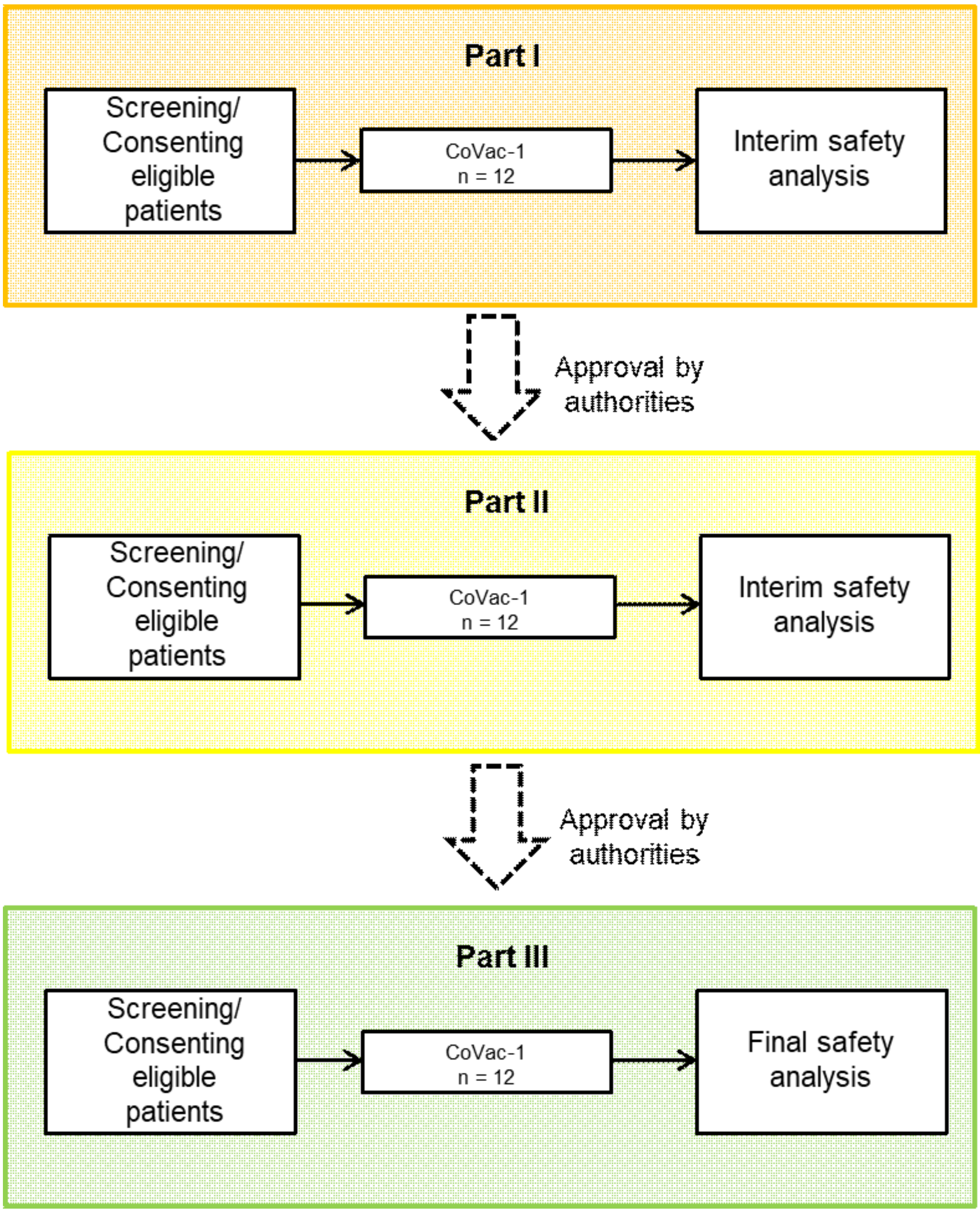
Part I
Screening/ Consenting eligible patients
CoVac-1
n = 12
Interim safety analysis
Approval by authorities
Part II
Screening/ Consenting eligible patients
CoVac-1
n = 12
Interim safety analysis
Approval by authorities
Part III
Screening/ Consenting eligible patients
CoVac-1
n = 12
Final safety analysis

Figure 2: Individual Study Procedure

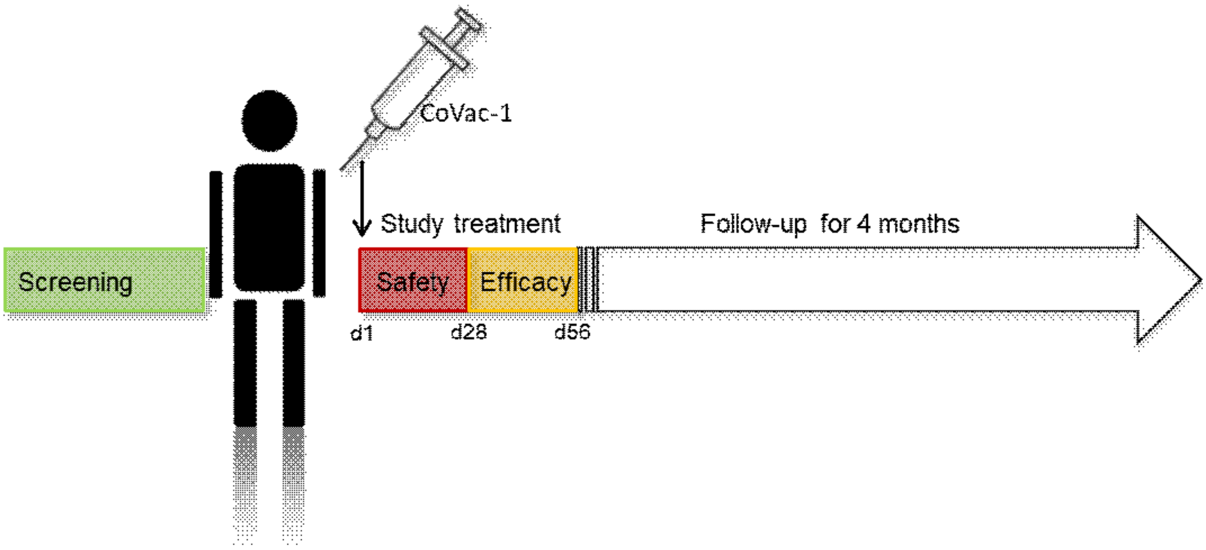

Figure 3: Treatment sequence

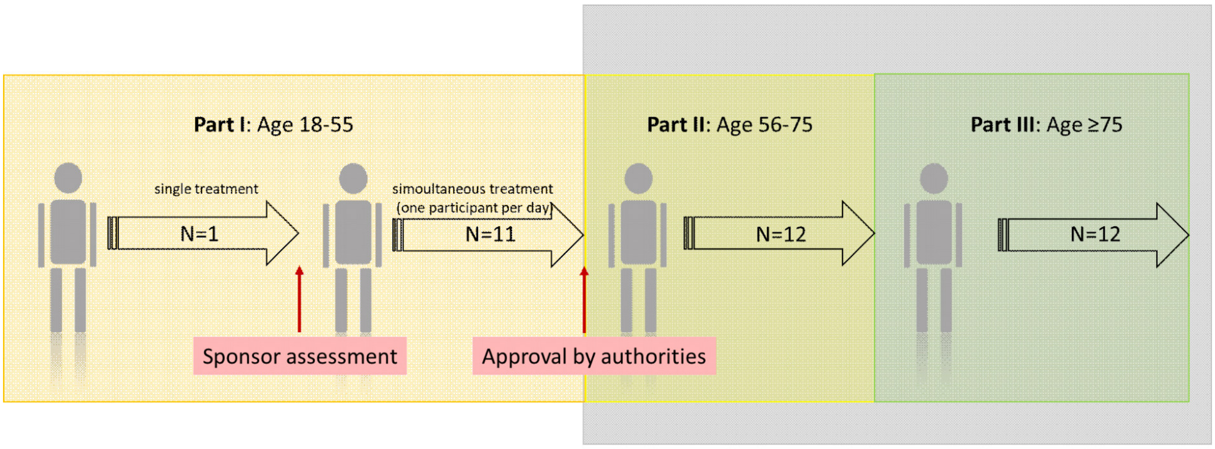

3.1. Study Duration and Schedule

The duration of the trial for each subject is expected to be 6 months, including 2 months of safety follow-up after vaccination and 4 months of follow-up.

The overall duration of the trial is expected to be approximately 12 months including the preparatory phase. Recruitment of subjects will start in Q3 2020. The actual overall duration or duration of recruitment may vary. The study timeline is described in Table 2.

Table 2: Study Timelines

Total trial duration	12 months
Duration for individual volunteer	Study treatment: 2 months<br><br>Follow-up: 4 months
FSI (First Subject In)	Q3/2020
LSI (Last Subject In)	Q1/2021
LSO (Last Subject Out)	Q3/2021
DBL (Data Base Lock)	Q3/2021
Statistical Analyses Completed	Q4/2021
Trial Report Completed	Q4/2021

3.2. End of Study

The end of the study is defined as the last visit of the last volunteer.

4. Study Population

Healthy subjects (designated as volunteers):

Healthy adult women and men aged 18-55 (Part I), followed by healthy adult women and men aged 56-74 (Part II) and adult women and men aged ≥ 75 (Part III).

Volunteers will be recruited by means of paper- and online-based calls as considered appropriate by the EC of the University Hospital of Tuebingen.

4.1. General Criteria for Subject Selection

Adult male and female volunteers fulfilling the inclusion criteria outlined below will be enrolled.
The trial population will consist of both genders. Gender distribution in the trial is supposed to reflect the distribution in the population; there will be no prior defined quantitative ratio between females and males.

4.1.1. Inclusion Criteria

Subjects meeting all of the following criteria will be considered for admission to the trial:

1. Adult male or non-pregnant, non-lactating female
    1. Part I: Age 18-55 at the time of screening
    2. Part II: Age 56-74 years at the time of screening
    3. Part III: Age ≥ 75 years at the time of screening
2. Pre-existing medical condition
    1. Part I and II: Free of clinically significant health problems, as determined by pertinent medical history and clinical examination at study screening
3. Ability to understand and voluntarily sign the informed consent form.
4. Ability to adhere to the study visit schedule and other protocol requirements.
5. FCBP and male volunteers with partners of childbearing potential, who are sexually active must agree to the use of two effective forms (at least one highly effective method) of contraception. This should be started from the signing of the informed consent and continue until three months after vaccination
6. Postmenopausal or evidence of non-childbearing status. For women of childbearing potential: negative urine or serum pregnancy test within 7 days prior to study treatment. Postmenopausal or evidence of non-childbearing status is defined as:

   1. Amenorrhoea for 1 year or more following cessation of exogenous hormonal treatments
   2. Luteinizing hormone (LH) and Follicle stimulating hormone (FSH) levels in the post menopausal range for women under 50

7. Be willing to minimize blood and body fluid exposure of others for 7 days after vaccination
   1. Use of effective barrier prophylaxis, such as latex condoms, during sexual intercourse
   2. Avoiding the sharing of needles, razors, or toothbrushes
   3. Avoiding open-mouth kissing
   4. Refrain from blood donation during the course of the study

4.1.2. Exclusion Criteria

Subjects presenting with any of the following criteria will not be included in the trial:

1. Pregnant or lactating females.
2. Participation in any clinical study with intake of any investigational drug interfering with the study primary endpoint
3. Any concomitant disease affecting the effect of the therapeutic vaccine or interfering with the study primary endpoint
4. Any immunosuppressive treatment except low dose corticosteroids (≤10mg prednisolone/day)
5. Prior or current infection with SARS-CoV-2 tested serologically or by throat/nose swab (PCR)
6. History of Guillain-Barré Syndrome
7. Positive serological HIV, hepatitis B or C test. In case of positive HBsAg, volunteer must provide prove of hepatitis B vaccination, otherwise volunteer must be excluded.
8. History of relevant CNS pathology or current relevant CNS pathology (e.g. seizure, paresis, aphasia, cerebrovascular ischemia/hemorrhage, severe brain injuries, dementia, Parkinson's disease, cerebellar disease, organic brain syndrome, psychosis, coordination or movement disorder, excluding febrile seizures as child)
9. Baseline laboratory with lymphocyte count ≤ 1000/µl
10. Only Part I:
    - Acute or chronic, clinically significant psychiatric, hematologic, pulmonary, cardiovascular, or hepatic or renal functional abnormality as determined by the

Investigator based on medical history, physical exam, and/or laboratory screening test

11. All parts of the clinical trial
    - Diabetes mellitus Typ II requiring drug treatment
    - Chronic lung disease requiring drug treatment
    - Any chronic liver disease or unknown liver abnormalities defined as:
        - ALT and AST ≤ 2.5 x ULN
        - γ-GT ≤ 2.5 x ULN
    - Chronic renal failure defined as GFR < 60 ml/min/1,73m^2
    - Serious pre-existing cardiovascular disease such as NYHA ≥ I, coronary heart disease requiring coronary surgery or known pAVK ≥ grade 2
    - Sickle cell anemia
    - Obesity (as defined by age adjusted body mass index)
12. Hospitalization at study inclusion
13. Administration of immunoglobulins and/or any blood products within 120 days preceding study entry or planned administration during the study period
14. History of blood donation within 30 days of enrolment or planned donations within the study period
15. Known hypersensitivity to any of the components included in the CoVac-1 vaccine

5. General Information on the Investigational Medical Product (IMP)

Definition of terms

**Drug substances:**	Six SARS-CoV-2-derived HLA class II peptides derived and the TLR1/2 ligand XS15
**Peptide cocktail:**	Peptide cocktail for each study volunteer including 6 immunogenic SARS-CoV-2 peptides and the TLR1/2 ligand XS15
**IMP/Drug product/ peptide vaccine:**	CoVac-1: Peptide cocktail emulsified in Montanide ISA 51 VG
**IMP administration:**	subcutaneous injection with 2ml syringe (e.g. BD Emerald) and needle (e.g. BD Eclipse Needle 27Gx1/2)

5.1. Peptide Vaccine CoVac-1

The IMP/drug product in this study is CoVac-1. The final peptide vaccine is a water-in-oil emulsion of the peptide cocktail as described in detail below and Montanide ISA 51 VG. All components will be provided by the Wirkstoffpeptidlabor of the Department of Immunology in Tübingen together with a "mixing kit" allowing for the mixture of the components (peptide cocktail, Montanide ISA 51 VG) by the pharmacy of the participating centers.

5.1.1. Peptide cocktail

5.1.1.1. SARS-CoV-2-specific peptides (drug substance)

Each volunteer enrolled in the P-pVAC-SARS-CoV-2 trial will receive 6 promiscuous HLA-DR peptides (240 µg each) derived from different proteins of SARS-CoV-2. Details on drug substance can be found in Table 3.

5.1.1.1. TLR1/2 ligand XS15 (drug substance)

The lipopeptide XS15 (50 µg), chemical name N-Palmitoyl-S-[2,3-bis(palmitoyloxy)-(2R)-propyl]-(R)-cysteinyl-GDPKHPKSF, a water-soluble synthetic Pam_3Cys-derivative is a TLR1/2 ligand that will be included as an adjuvant in the peptide cocktail.

5.1.2. Montanide ISA 51 VG

Prior to application, the peptide cocktail (consisting of 6 SARS-CoV-2-specific HLA-DR peptides and the TLR1/2 ligand XS15) will be emulsified in a water-oil emulsion 1:1 with Montanide ISA 51 VG. Montanide ISA 51 VG is based on a blend of mannide monooleate surfactant and mineral oil and has been used as an adjuvant in more than 200 human vaccine trials. Montanide ISA 51 VG is rendering stable water-in-oil emulsions when mixed with water-based antigenic media.

Table 3: SARS-CoV-2 specific HLA-DR vaccine peptides

sequence	HLA restriction	peptide length	position	protein	protein name	protein class
ASWFTALTQHGKEDL	DR	15	50-64	ORF9	nucleocapsid protein	structural
LLLLDRLNQLESKMS	DR	15	221-235	ORF9	nucleocapsid protein	structural
ITRFQTLLALHRSYL	DR	15	235-249	ORF9	spike protein	structural
LSYYKLGASQRVAGD	DR	15	176-190	ORF5	membrane protein	structural
FYVYSRVKNLNSSRV	DR	15	56-70	ORF4	membrane protein	structural
SKWYIRVGARKSAPL	DR	15	43-57	ORF8	n.a.	non-structural

5.2. Manufacturing of the Investigational Medicinal Product

5.2.1. SARS-CoV-2-specific peptides (drug substance)

All SARS-CoV-2 vaccine peptides are manufactured by the Wirkstoffpeptidlabor, University of Tübingen, Auf der Morgenstelle 15, 72076 Tübingen, Germany. The Wirkstoffpeptidlabor holds certificates for the production of GMP grade synthetic peptides and for the formulation of multi-peptide vaccine cocktails including the TLR1/2 ligand XS15. All peptides are synthetic peptides manufactured by well-established solid phase peptide synthesis (SPPS) procedures using Fmoc chemistry.

5.2.2. XS15 (drug substance)

XS15 is delivered as bulkware in GMP-quality from the external manufacturer Bachem AG, Hauptstrasse 144, CH-4416 Bubendorf in active ingredient quality.

Bachem's manufacturing process is described in a separate "Documentation on XS15 Hydrochloride" of 31.05.2018 by the company. The Wirkstoffpeptidlabor performs a second lyophilization as additional manufacturing step. This manufacturing step is divided into four sub-steps: Reconstitution, combining, aliquoting and lyophilization.

5.2.3. Montanide ISA 51 VG

Montanide is manufactured by Seppic and by the rewarding manufacturer Elaiapharm, respectively.

5.2.4. Peptide cocktail CoVac-1 (drug product)

The peptide cocktail is manufactured by the Wirkstoffpeptidlabor by aseptic filling at the GMP-Center of the University Hospital Tuebingen. Each peptide is solubilized in DMSO and sterile filtered, the obtained peptide solutions are pooled. Water is added and the obtained solution is sterile filtered and filled into single dose vials.

5.3. Labeling of the Investigational Medicinal Product

5.3.1. Peptide cocktail

Peptide cocktails (including the TLR1/2 ligand XS15) will be packaged into sterile containers labeled with an identification code definitely assignable to the P-pVAC-SARS-CoV-2 study and a vial number that will be assigned to the individual study volunteer. The trial medication will be labeled according to § 5 of GCP-V. Samples of the labels are filed in the trial master file (TMF).

The peptide vaccine cocktail will be packaged together with Montanide ISA 51 VG and the mixing equipment into the “mixing kit” and shipped from the *Wirkstoffpeptidlabor* of the Department of Immunology, Tübingen to the pharmacy of the participating center. Shipment will be documented according to standard operation procedures (SOP). The “mixing kit” will be shipped using isolated packaging with an automated temperature control system, whose logging data have to be returned to the Wirkstoffpeptidlabor of the Department of Immunology together with the acknowledgement of receipt after delivery of the consignment. The device will be read out to document the correct storage temperatures during shipment. Data will be documented according to SOP. The shipment will be performed by an associate of the Wirkstoffpeptidlabor of the Department of Immunology, Tübingen.

5.3.2. Montanide ISA 51 VG

Montanide ISA 51 VG is packed by Seppic and Elaiapharm. Montanide will be packaged together with the peptide cocktail and the mixing equipment into the “mixing kit” and shipped from the Wirkstoffpeptidlabor of the Department of Immunology, Tübingen to the pharmacy of the participating center, as described above.

5.4. Storage of the Investigational Medicinal Product

Trial medication will be stored at the pharmacy of the participating center and must be kept in a locked area with access restricted to designated trial staff. The “mixing kit” including the peptide cocktail and Montanide ISA 51 VG must be stored in accordance with manufacturer’s instructions at -20°C and dry. The investigator must ensure that the investigational products are stored according to the sponsor’s instructions (temperature, light and humidity) and should control the integrity of the packaging upon receipt. If concerns about the quality or appearance of the investigational products arise, the products may not be dispensed. In this case, the principal investigator must be contacted immediately.

5.5. Drug Accountability, Therapy Compliance and Disposal

The investigator or the site personnel will keep an account of the trial medication and acknowledge the receipt of all shipments of the trial medication. Trial medication will be ordered by the investigator and delivered by the Wirkstoffpeptidlabor to the pharmacy of the participating center. The investigator will document the date of dispensary, subject identification, batch/serial numbers or other identification of trial medication. Upon completion or termination of the study, all unused “mixing kits” have to be returned to the Wirkstoffpeptidlabor of the Department of Immunology. The returned products must be accompanied by adequate documentation and identified clearly with trial site and patient number. The return of any unused study medication must be coordinated by the responsible study monitor/study nurse/pharmacy. Empty packaging does not have to be returned. The disposal is in the responsibility of the study center according to the German laws and local and institutional guidelines and procedures for litter disposal.
In case of SAEs related to the vaccination peptides or adjuvant, the study medication will be returned to the Wirkstoffpeptidlabor of the Department of Immunology, Tübingen for further analysis. The returns will be documented according to SOP.

The returned charges will be locked and deleted according to SOP. A declassification of a drug for clinical use for an application in *in vitro* research experiments is not touched by the declaration. This declassification will be documented. Unused charges of vaccination peptides will be returned to the Wirkstoffpeptidlabor of the Department of Immunology, Tübingen and will be stored.

All waste will be discharged according to German waste laws (date of issue 27.09.1994).

The IMP CoVac-1 may only be applied to subjects included in the P-pVAC-SARS-CoV-2 trial. Other individuals must not receive peptides produced for the P-pVAC-SARS-CoV-2 trial.

Investigational products must be dispensed only by trained and authorized personnel according to legal regulations. Physicians outside the study facility may not apply the study drugs.

5.6. Method of Treatment Assignment

After screening and enrolment, volunteers will be assigned to treatment with CoVac-1.

5.7. Dose Schedule

The CoVac-1 vaccine (500 µl) will be administered subcutaneously. Emulsification will be performed by the pharmacy of the participating center according to the “Anmischanleitung Montanide-Emulsion” provided with the “Mixing Kit” by the Wirkstoffpeptidlabor of the Department of Immunology Tübingen. Final vaccine drug product has to be stored at room temperature and to be administered within 24 h after mixing of the components. For qualification of the pharmacy and study center staff regarding ordering and mixing of the peptide vaccine cocktail with Montanide ISA 51 VG, a controlled dry run process will be performed.

The mixing of the peptide vaccine cocktail and Montanide ISA 51 VG will be performed by local pharmacy and the investigator will be provided with a syringe containing the final drug product CoVac-1. A subcutaneous injection of 500 µl (approx. 240 µg per peptide, 50µg XS15) will be applied. A single vaccination per patient will be conducted.

Vaccination instruction

Peptide vaccines should be injected into the skin at the lower part of the abdomen of the volunteers. The site of vaccination (right or left) will be determined by the investigator. At investigators discretion antihistamins such as 4 mg dimetindene can be applied as i.v. injection or infusion about 30 minutes prior to application of the vaccine.

5.7.1. Dose modifications for peptide vaccine

No dose modification is planned in this trial.

5.7.2. Side effects

5.7.2.1. Side effects of peptide vaccination

Peptide vaccination is generally well tolerated. Mild reactions at local vaccination sites are the most common side effects, followed by fatigue[73] [84]. Peptide vaccination can lead to immediate anaphylactic reactions with elevation of heart rate, hyperhidrosis and subjective feeling of dizziness, in rare cases with concomitant drop in blood pressure[63] [63] [73]. Cutaneous erythema at the vaccination site was observed more frequently and may persist for up to five weeks. Also, there is a risk of granuloma formation. Some of the patients reported one episode of fever not lasting more than two days. No grade III or IV toxicities were observed in former peptide vaccination studies, including an early trial with a peptide based malaria

vaccine, which only reported mild local reactions in approximately 50% of volunteers[63 70 73]. Furthermore, no signs for the development of antibody-dependent enhancement (ADE) was reported. Of note, side effects in the reported studies are most likely attributable to the applied adjuvants.

In our ongoing iVAC-CLL01 study using peptide cocktails, most of the patients experienced mild local skin reactions at the vaccination site. No anaphylactic or allergic reaction, or other AE related to the peptide vaccine was observed.

In the P-pVAC-SARS-CoV-2 study, patients will be monitored for heart rate, blood pressure, temperature and subjective well-being after vaccination for at least 2 hours. The volunteers will be discharged after documentation of these parameters. More detailed information on CoVac-1 vaccine peptides is provided with the current IB (Version 1.0).

5.7.2.2. Side effects of XS15

The TLR 1/2 ligand XS15 will be administered subcutaneously together with the SARS-CoV-2 specific peptides emulsified in Montanide ISA 51 VG. XS15 was never used in a clinical trial before. Common side effects of other TLR ligands used for peptide vaccination are reported to be usually mild, comprising local skin reactions, fatigue, flu-like symptoms like fever, muscular pain and ague. TLR ligands can worsen pre-existing autoinflammatory skin disorders.

Previous application of XS15 in a healthy volunteer and cancer patients (within the scope of individual healing attempts) did, besides local reactions at the vaccination site including formation of granuloma, not cause relevant systemic side effects, in particular no allergic or anaphylactic reactions. More detailed information on XS15 is provided with the current IB (1.0. 27 May 2020).

5.7.2.3. Side effects of Montanide ISA 51 VG

Montanide ISA51 is an oil adjuvant suitable for human injection that will be administered together with the SARS-CoV-2 specific peptides and XS15 subcutaneously. Montanide ISA 51 VG was used as an adjuvant in more than 100 peptide vaccination. Most common side effects are injection site reaction (68%) including granuloma development, fatigue (54%), fever (41%), gastrointestinal disorders (32%) and injection site or local erythema (28%)[83]. In general, the observed AEs from controlled trials involving non-healthy as well as healthy individuals were mild to moderate in intensity. Further side effects rarely reported were erythema nodosum (2/36 patients, 5%)[85] and the development of sterile abscesses at injection site[86].

More detailed information on Montanide ISA 51 VG is provided with the current IB (Version 3291/GB/03/June 2019).

6. Study Procedures and Examination Method

This study will consist of the following consecutive phases: Study entry, vaccination/treatment and follow-up. Time-points and trial procedures are listed in Table 1.

6.1. Study Entry

6.1.1. Volunteer's Informed Consent

Subjects are informed both in writing and verbally by the investigator before any study-specific procedure is performed. Each volunteer will be informed about the modalities of the clinical study in accordance with the provided volunteer information. The volunteer is given sufficient time (≥ 24 h) to consider participation in the clinical trial and to ask for additional advise if needed. Informed consent from the volunteer will be obtained using a form approved by the responsible EC. The volunteer and informing investigator must each personally date and sign the informed consent form containing an integrated declaration on data privacy protection. The original signed document will be part of the investigator's site file and retained with it, a copy including the insurance policy of the trial will be handed to the volunteer. The informed consent process is documented in the volunteer records.

6.1.2. Screening

Screening will be performed within *one* week (7 days) prior to the administration of the CoVac-1 vaccine. After having signed the informed consent form, volunteers will undergo all assessments listed below:

- Demographics
- Medical history
- Enrolment
- Vital signs
- Physical examination
- Concomitant medications
- QoL assessment
- Hematology (local lab)
- Blood chemistry and coagulation (local lab)
- Urine analysis (local lab)
- Immunoglobulins/Immunophenotype (local lab), approximately 10 ml blood
- Testing for previous or current SARS-CoV-2 infection: 5ml serum blood will be drawn for antibody testing and a nose/throat swab* will be performed.

- HBV, HCV, HIV-1, (local lab)
- Pregnancy test

* If screening takes more than 48 hours, throat/nose swab for SARS-CoV-2 infection must be repeated. The vaccine can only be applied if a negative SARS-CoV-2 PCR test is available on the day of vaccination not older than 48 hours.

The investigator will review all information obtained from the screening procedures via an eligibility form. The investigator will confirm, in writing, whether the subject fulfils all criteria for eligibility. Volunteers who fulfil all the inclusion criteria and none of the exclusion criteria will be eligible to participate in the trial. Screening failures, i.e. screened volunteers not in compliance with all criteria, are to be excluded and the reason will be recorded in the volunteer records.

Information of volunteer's trial participation can be provided to the volunteer's general practitioner if the volunteer agrees.

6.1.3. Enrolment

A volunteer is considered for screening when he or she has signed the Informed Consent form.

In case of confirmation of volunteer's eligibility (volunteers must meet all inclusion criteria and must not meet any exclusion criteria), volunteer will be registered under a specific Vol. ID on a subjects log kept at the trial site. Only these volunteers are enrolled in the study, all others are assessed as screening failures.

The study is open-label.

6.1.4. Randomisation

No randomisation will be done in this clinical trial.

6.1.5. Concomitant Medication and Treatments

Relevant additional medications and treatments administered to the subjects on entry to the trial or at any time during the trial are regarded as concomitant medications and treatments and must be documented on the appropriate pages of the CRF.

6.1.6. Permitted Prior and Concomitant Medications and Treatments

The following concomitant medications and treatments are permitted during the trial.

Part I: No concomitant medication, apart from contraception for FCBP.

Part II & III: Any concomitant medication (already applied at screening) for e.g. other diseases are allowed except for medications stated in section 6.1.7.

6.1.7. Prohibited Prior and Concomitant Medications and Treatments

The following concomitant medications and treatments are prohibited during the trial:

- Immunosuppressive agents apart from (≤ 10 mg prednisolone or equivalent)
- During the trial, other vaccinations or non-urgent medical interventions are prohibited. Initiation of new medications, regardless of indication must be discussed with the investigator and must be noted on the participant’s record.

6.1.8. Contraception

Within this study, all FCBP must have a negative pregnancy test ≤ 7 days prior initiation of study treatment. A FCBP is defined as any female who does not meet the criteria of non-childbearing potential. These are as follows:

- documented hysterectomy, bilateral oophorectomy (ovarectomy), or bilateral tubal ligation
- post-menopausal (a practical definition accepts menopause ≥ 1 year without menses with an appropriate clinical profile, e.g. age > 45 years in the absence of hormone replacement therapy (HRT). In questionable cases, the subject must have a follicle stimulating hormone (FSH) value > 40 mIU/ml and an estradiol value < 40pg/ml.

Sexually active men and women of child-bearing potential must use two methods of reliable contraception including one highly effective (Pearl Index < 1) and one additional effective (barrier) method as described below maintained for up to 3 months after the last dose of study therapy.

The following contraceptive methods with a Pearl Index < 1 are regarded as highly-effective:

- oral hormonal contraception (‘pill’)

Please note: in case that its efficacy is impaired during the trial, e.g. due to vomiting and diarrhoea, additional/other methods as listed below are required to assure adequate safety

- dermal hormonal contraception/contraceptive plaster
- vaginal hormonal contraception (NuvaRing®)
- long-acting injectable contraceptives/implants that release progesterone (Implanon®)
- tubal ligation (female sterilization)
- intrauterine devices that release hormones (hormone spiral)
- double barrier methods

- partner's vasectomy

Additional effective (barrier) methods are:

- male condom
- diaphragm/cervical cap

The following contraceptive methods are not regarded as safe: condom plus spermicide, simple barrier methods (vaginal pessaries, condom, female condoms), copper spirals, rhythm/basal temperature method and withdrawal method (coitus interruptus).

6.2. Vaccination Phase

Vaccination phase begins as soon as possible (within 7 days) after screening and confirmation of patient's eligibility. If screening takes more than 48 hours, throat/nose swab for SARS-CoV-2 infection must be repeated. The vaccine can only be applied if a negative SARS-CoV-2 PCR test is available on the day of vaccination not older than 48 hours.

Peptide vaccines should be injected into the skin at the lower part of the abdomen of the patients. The site of vaccination (right or left) will be determined by the investigator and documented.

To minimize the risk for severe and unexpected side effects for subjects included in the study, all participants will be monitored for at least two hours after vaccination, including close monitoring of heart rate, blood pressure, temperature, oxygen saturation and subjective well-being. Each monitoring unit must be equipped with a crash cart and an intensive care team should be on standby.

Treatment and monitoring of the first volunteer are performed in an in-patient setting with access to intensive care for 24h. Close monitoring (every 30 minutes vital parameters) will be performed for the first four hours after vaccination. Thereafter, monitoring is performed at hourly intervals until 6 hours after vaccination. Thereafter every 3 hours until 24 hours after application of the vaccine.

6.2.1. Visit 1 (Vaccination) (Day 1)

- Signs/symptoms, baseline
- Vital signs, close monitoring after vaccination (blood pressure, temperature, heart rate and oxygen saturation every 30 minutes for at least 2 hours)
- Physical examination, baseline
- Assessment of concomitant medications

- AE assessment
- Hematology and blood chemistry (local lab), approximately 10 ml blood
- Vaccination (section 5.7)
- T-cell response, baseline obtained before vaccination, approximately 60 ml blood
- Serological response, baseline obtained before vaccination, approximately 15 ml blood

6.2.2. Visit 2 (Day 7 +/- 1)

- Signs/symptoms, as assessed on volunteer's diary and visit
- Vital signs,
- Physical examination, including investigation of vaccination side
- Assessment of concomitant medications
- AE assessment
- Hematology and blood chemistry (local lab), approximately 10 ml blood
- T-cell response, approximately 60 ml blood
- Serological response, approximately 15 ml blood

6.2.3. Visit 3 (Day 14 +/- 1)

- Signs/symptoms, as assessed on volunteer's diary and visit
- Vital signs,
- Physical examination, including investigation of vaccination side
- Assessment of concomitant medications
- AE assessment
- Hematology and blood chemistry (local lab), approximately 10 ml blood
- T-cell response, approximately 60 ml blood
- Serological response, approximately 15 ml blood

6.2.4. Visit 4 (Interim safety) (Day 28 +/- 2)

- Signs/symptoms, as assessed on volunteer's diary and visit
- Vital signs,

- Physical examination, including investigation of vaccination side
- Assessment of concomitant medications
- AE assessment
- Hematology and blood chemistry (local lab), approximately 10 ml blood
- T-cell response, approximately 60 ml blood
- Serological response, approximately 15 ml blood

6.2.5. Visit 5 (End of Safety follow-up = EOSf)

- Signs/symptoms, as assessed on volunteer's diary and visit
- Vital signs,
- Physical examination, including investigation of vaccination side
- Assessment of concomitant medications
- AE assessments
- Hematology and blood chemistry (local lab), approximately 10 ml blood
- T- cell response, approximately 60 ml blood
- Serological response, approximately 15 ml blood

6.2.6. Visit 6-7 (Follow-up) (Month 3 and 6 +/- 7 days)

- Medical history, anamnestic evaluation of SARS-CoV-2 specific symptoms
- T-cell response, approximately 60 ml blood
- Serological response, approximately 15 ml blood

6.2.7. Volunteer's diary/card

Each patient included in the P-pVac-SARS-CoV-2 study will receive a volunteer's card, which states that he/she is participating in the study (Appendix 13.4). This will also include a 24h emergency contact number. Furthermore, each patient will be provided with a volunteer's diary to note their symptoms daily (Appendix 13.3)

6.2.8. Unscheduled Visit

Subjects may contact the investigator at any time for an unscheduled phone or on-site visit should they experience clinical symptoms or signs following injection. At all unscheduled visits, the following minimum assessment will be performed: Questions concerning the history of the present illness as well as the subject's general health and lifestyle. Findings

resulting in (S)AEs will be documented and reported as indicated. All other symptoms/signs will be reported on the next scheduled visit on eCRF.

Upon occurrence of symptoms characteristic of SARS-CoV-2 (i. e. cough, fever (cut-off >39°C), loss of taste and smell, limb pain) at any time until day 56, subjects are supposed to get in touch with the investigator. Investigator will initiate SARS-CoV-2 testing for the volunteer (nose or mouth swab followed by PCR per institutional guidelines). If the test is positive, patients should be treated per investigators discretion. Positive results must be recorded as an AESI (section 9.1.4). Negative results will be followed by a second testing ≥ 24h later. Only upon the second negative test, patients are considered negative, all others must be reported as positive.

If participants are positively tested for SARS-CoV-2, all accompanying symptoms and treatments (e.g. hospitalisation, ICU) are recorded

Medically attended AEs and all SAEs will be recorded, and concomitant medication or vaccination will be noted. After identifying the history of the present illness and performing corresponding exams or laboratory tests, the investigator will decide on the best course of treatment according to standard medical practice.

6.3. Assessment of Efficacy

6.3.1. Efficacy Parameters

Immunological Efficacy:

Induction of SARS-CoV-2-specific $CD8^+$ and $CD4^+$ T cells is evaluated using:

- IFN-γ ELISPOT
- Intracellular cytokine staining for TNF and IFN-γ

Induction of SARS-CoV-2 specific antibodies:

- ELISA

6.3.2. Methods and Timing for Assessing, Recording, and Analysing of Efficacy Parameters

Immunological Efficacy:

Serial measurements of immunological efficacy will be performed prior to peptide vaccination (V1), and V2, V3, V4, at the end of study visit and the follow up visits as outlined in table 1. All scheduled visits have a ± 1 day window unless otherwise stated. 75ml peripheral blood (60 ml Na^+-heparin and 15 ml serum) for immunological assays will be obtained prior to vaccination as indicated in table 1. Immunological assays will be performed in the Department of Immunology or the Immunopathological Laboratory, Department of Internal Medicine, University Hospital Tuebingen based on standard SOPs.

<u>Amplification of SARS-COV-2-specific T cells:</u>

PBMCs from volunteers are pulsed the respective peptide and cultured for 12 days adding IL-2 on days 3, 5, and 7. Peptide stimulated PBMCs are analyzed by enzyme-linked immunospot (ELISPOT) assay on day 12 or by flow cytometry-based tetramer and intracellular cytokine staining as described below.

<u>IFN-γ ELISPOT assay</u>

IFN-γ ELISPOT assays are carried out as described previously.[87] In brief, 96-well nitrocellulose plates are coated with anti-IFN-γ. Plates are blocked and PBMCs (*ex vivo* or after T-cell amplification as described above) are distributed to the wells and re-stimulated with HLA class II peptides. Cytokine staining is performed after incubation period. Analysis is performed according to manufacturer's instructions. Spots are counted using an Immunospot analyzer. T cell responses are considered to be positive when the mean spot count per well is at least 3-fold higher than the mean number of spots in the negative control wells (according to the cancer immunoguiding program (CIP) guidelines).[88]

To differ between vaccine induced and natural T-cell induction by SARS-CoV-2 infection we will included, beside the T-cell epitopes included in the CoVac-1 vaccine, additional SARS-CoV-2 T-cell epitopes defined in our preclincial work in the peptide readout [24].

Cellular conversion rate (CCR) is calculated by dividing the number of volunteers with an immune response by the number of tested participants to a time point (Visit 2, 3, 4 and 5). A volunteer is considered as having developed an immune response due to immunization if *ex vivo* IFN-γ ELISPOT assay is positive (as described above) and the spot count is at least 2-fold higher than the baseline assay (Visit 1).

<u>Intracellular IFN-γ and TNF staining</u>

The frequency and functionality of peptide-specific $CD8^+$ T cells is analyzed by intracellular IFN-γ or TNF staining as described previously.[87 89] PBMCs are pulsed with individual peptide

and incubated in the presence of Brefeldin A and GolgiStop. Cells are labeled using Cytofix/Cytoperm, CD8, CD4, TNF and IFN-γ coupled to fluorochromes. Samples are evaluated on a FACS analyzer.

Enzyme-linked immunosorbent assay (ELISA)

To identify SARS-CoV-2 antibody responses induced by the vaccine, ELISA assays will be performed using serum samples (15 ml serum tube) obtained at the time points described in Table 1. Specific antibodies against the seven SARS-CoV-2 T-cell epitopes will be assessed by ELISA assay at the Department of Immunology, Tübingen. To differ between vaccine induced antibody response additional standard Elecsys® Anti-SARS-CoV-2 assay supplied by F. Hoffmann-La Roche AG, Basel, Switzerland will be performed at central laboratory of the University Hospital Tuebingen.

Occurrence or relevant (≥2-fold) increase of SARS-CoV-2 specific IgG antibodies compared to baseline are considered as positive.
In the unlikely event of antibody induction by the CoVaC-1 vaccine, neutralization capacity of antibodies will be assessed by SARS-CoV-2 Pseudovirus Neutralization Assay (CD, Creative Diagnostics®)

6.4. Assessment of Safety

6.4.1. Safety parameters

(Serious) Adverse Events (see section 9)

- Vital signs: pulse, blood pressure, temperature, and weight
- Physical examination including inspection of the vaccination side
- Clinical laboratory evaluations:
  Hematology: white blood cell (WBC), hemoglobin (Hb), platelet count, absolute neutrophil count (ANC), absolute lymphocyte count (ALC)
  Chemistry: AP, total bilirubin, AST/ SGOT, ALT/ SGPT, LDH, and uric acid, CRP, sodium, potassium, calcium, blood urea nitrogen, creatinine, glucose, C-reactive protein
- Concomitant medications
- (S)AEs by NCI CTCAE Version 5.0 and as in appendix 14.5

6.4.2. Methods and Timing for Assessing, Recording, and Analysing Safety Parameters

Serial measurements of safety will be performed at screening and at scheduled intervals throughout the duration of the study as outlined in table 1. All scheduled visits have a ± 1 day window unless otherwise stated. Abnormalities will be captured as protocol deviations. Lab abnormalities grade 1-2 are only considered AE if they fulfill one of the following criteria:

- Accompanied by clinical symptoms.
- Requiring a change in concomitant therapy (e.g. addition or change in a concomitant medication, therapy or treatment).

All Grade 3-4 laboratory abnormalities fulfilling the criteria for an SAE will be reported as SAEs and will be recorded on the AE pages of the CRF; however, those that are not deemed by the investigator to be part of a diagnosis or syndrome will not be reported to the Health Authorities in an expedited manner. Cause of death is to be recorded in the CRF and the subject's medical record.

6.5. Vaccination holding rules

Safety holding rules for each subject will apply throughout the study period until interim safety analysis (V4). Vaccination of further study subjects in the consecutive study phase will not occur until a safety review has been conducted by the DSMB and only by approval a holding rule can be resolved. If a holding rule is activated, the PI will inform the Sponsor within 48 hours. The Sponsor will inform the responsible authorities (PEI and EC).

If the DSMB permits the resumption of injections, a formal request with pertinent data must be submitted to ECs and PEI. The discontinuation of a holding rule should be communicated to all entities in the same manner and timeframe as described above.

The DSMB safety review will consider:

- The relationship of the AE or SAE to the vaccine or other possible causes of the event.
- If appropriate, additional screening or laboratory testing for other volunteers to identify those who may develop similar symptoms and alterations to the current informed consent form will be discussed.

All injected volunteers will be followed for safety until resolution or stabilization (if determined to be chronic sequelae) of their AE.

<u>The holding rules are as follow:</u>

- Solicited local ADRs: If more than 30% of injections are followed by Grade ≥3 solicited swelling or pain or Grade 4 redness beginning within 3 days after injection (day of injection and 2 subsequent days) and persisting at Grade 3 (swelling or pain)/4 (redness) for > 48 h to maximum 72 hours depending upon symptom severity and kinetics.
- Solicited systemic AEs: If more than 25% of injections are followed by Grade 3 solicited systemic AE beginning within 3 days after study injection (day of injection and 2 subsequent days) and persisting at Grade ≥ 3 for > 48 h to maximum 72 hours depending upon symptom severity and kinetics.
- Unsolicited AEs: If more than 25% of volunteers develop a Grade ≥ 3 unsolicited AE (including laboratory AE and physical observations) that is considered probably or definitely related to injection and persists at Grade 3 for > 48 to maximum 72 hours depending upon symptom severity and kinetics.
- A suspected unexpected serious adverse drug reaction (SUSAR) occurs that is life-threatening or results in death.

6.6. Premature termination of clinical trial for a trial subject

Reasons for premature termination of trial for an individual trial subject are:

1. Death
2. Withdrawal of consent
3. Volunteer lost to follow-up
4. For women, in case of pregnancy

The PI decides about withdrawal of subjects from trial treatment in case of occurrence of criteria mentioned above. In all cases, the reason for withdrawal must be recorded in the CRF and in the subject's medical records. In case of withdrawal of a subject at his/ her own request, the reason should be determined and documented.

All examinations scheduled for the last trial day will be performed and documented as far as possible, subject to consent of the volunteer. Subjects will enter the regular follow-up of the trial, unless the subject has withdrawn his/her consent to any further study-related procedure. If a subject is withdrawn from all trial-related procedures (including follow-up visits) e.g. at his/her own request, this will not result in any disadvantages for the volunteer.

All ongoing Adverse Events (AEs)/ Serious Adverse Events (SAEs) of withdrawn subjects have to be followed-up until no more signs and symptoms are verifiable or the subject is on stable condition.

Premature termination should be avoided. In case of a premature termination of study, reasons/circumstances and if applicable the final status have to be documented. If volunteers do not withdraw the consent for further follow-up, they should be followed-up as planned.

6.7. Premature closure of a trial site

Premature closure of a trial site has to be considered if:

- The recruitment rate is not sufficient
- The conduct of the study is not compliant with the protocol or the legal regulations, or
- The data quality is not sufficient

The premature closure of a site will be decided by the sponsor.

Site principal investigators may terminate his/her participation in the study. If this occurs they should provide a written statement of the reasons for terminating participation and must provide the sponsor with all available and up-to-date study data.

The sponsor may also decide to terminate participation of an investigator or study centre for the following reasons:

- Breach of agreement
- Serious non-compliance to protocol or the legal regulations
- Insufficient volunteer recruitment

If a participating center closes, or is closed, prior to termination of the whole trial, the sponsor expects that data from volunteers already entered into the trial will be reported as per protocol. Details on further treatment and follow-up of volunteers on study have to be discussed with the site principal investigator.

6.8. Premature termination of the trial

The trial may be prematurely terminated, if in the opinion of the sponsor and coordinating investigator, there is sufficient reasonable cause. Written notification documenting the reason for study termination will be provided to the investigators.

In case of the following situations a premature termination of the trial has to be considered:

- Observation of one SAE associated with administration of CoVac-1 (Statistical Stopping rule of the study)
- Serious adverse drug reactions / not justifiable toxicity
- Substantial changes in risk-benefit considerations
- New insights from other trials
- Insufficient efficacy
- Insufficient recruitment rate

The DSMB will monitor the study conduct and the safety aspects of the trial on a regular basis, and will give recommendations to the coordinating investigator/ the sponsor whether to stop the trial or to change the trial protocol. The sponsor will then decide on the actions to be taken. According to the German drug law (§42a), the trial may be suspended or prematurely terminated by decision of the competent authority (PEI).

6.9. Follow Up

Volunteers will be followed for up to 4 months after EOSf. Thereafter patients may be contacted by phone call/e-mail to assess infection with SARS-CoV-2.

6.10. End of Study for Subjects

The end of Study for a subject enrolled in this trial is defined as the last study visit.

7. Quality control and Quality assurance

7.1. Risk-based approach

During protocol development, processes and data that are critical to ensure human subject protection and the reliability of trial results were identified.

The identified risks were evaluated against existing risk controls by considering:

- The likelihood of errors occurring
- The extent to which such errors would be detectable
- The impact of such errors on human subject protection and reliability of trial results.

In case of unacceptable risks, risk reduction activities were defined and incorporated e.g. in the protocol, monitoring plan and agreements.

Results will be communicated to those who are involved in or affected by such activities.

The sponsor periodically reviews risk control measures to ascertain whether the implemented activities remain effective and relevant, taking into account emerging knowledge and experience.

7.2. Monitoring

Monitoring for this study is provided by the *Zentrum für Klinische Studien Tuebingen (ZKS Tuebingen).* The monitoring will be conducted according to *ZKS Tuebingen Internal Standard Operating Procedures (SOPs)* and a dedicated monitoring manual for the study. The monitoring timelines include, for all centres, initiation visit, regular monitor visits during the course of the trial as well as a close out visit. Usually, monitoring will end with the last visit after full documentation of the last volunteer enrolled (close out visit). All investigators agree that the monitors regularly visit the trial site, assure that the monitors will receive appropriate support in their activities and will have access to all trial-related documents.
The aims of the monitoring visits are as follows:

- Check informed consent documents
- Monitor trial subject safety (occurrence and documentation/reporting of Serious Adverse Events (SAEs) and Adverse Events (AEs)).
- Check completeness and accuracy of entries on the CRFs.
- Validate entries on the CRFs against those in the source documents (source data verification (SDV)).

- Check the Drug Account
- Check the storage conditions of the IMP
- Evaluate the progress of the trial
- Evaluate compliance with the trial protocol
- Assess whether the trial is being performed according to GCP at the trial site
- Discuss with the investigator aspects of trial conduct and any deficiencies found
- A monitoring visit report is prepared for each visit describing the progress of the clinical trial and any problems

7.3. Audits/ Inspections

In addition to the monitoring activities, audits can be conducted by the sponsor or assigned auditors. These audits may include checking the whole course of the study, documentation, trial centre, investigators and the monitor.

The competent regulatory authorities may also conduct inspections.

With his/her participation in the study, the investigator agrees to support the activities of the auditor/inspector, provide her/him with direct access to the source documents, study documentation and give her/him the opportunity to audit/inspect the study site, laboratory facilities, storage of the investigational product, etc.

7.4. Documentation: Collection, Handling, Storage and Archiving of Data

7.4.1. Case Report Form

The trial Case Report Form (CRF) is the primary data collection instrument for the trial. All data requested on the CRF must be recorded. All missing data must be explained.

For this project, electronic Case Report Forms (eCRFs) will be used. The Clinical Data Management System [secuTrial “SecuTrial”] will be used for data capture, processing and storage of study data. Data entry is performed at the investigational site by clinical staff after having received training and a user manual for the electronic CRF. Training and the user manual will detail procedures to be followed in case of technical problems. Queries resulting from edit checks and/or data verification procedures will be posted electronically in the eCRF.
The Clinical Trial Data Management System (CDMS) is validated and changes are tracked via an audit trail.

The correctness of entries in eCRFs will be confirmed by dated signature of an authorized investigator. The Principal investigator is responsible for ensuring that all sections of the eCRF are completed correctly and that entries can be verified against source data. The Principal investigator has to verify the eCRFs via dated electronic signature after completion of the eCRF.

7.4.2. Source Data

Source data is all information, original records of clinical findings, observations or other activities in a clinical trial necessary for the reconstruction and evaluation of the trial. Source data are contained in source documents. Examples of these original documents and data records include: hospital records, clinical and office charts, laboratory notes, memoranda, volunteers' diaries or evaluation checklists, pharmacy dispensing records, recorded data from automated instruments, copies or transcriptions certified after verification as being accurate and complete, x-rays, CTs, MRIs, ultrasound reports, volunteer files, and records kept at the pharmacy, at the laboratories, and at medico-technical departments involved in the clinical trial.

7.4.3. Data Handling

Authorized clinical staff at the investigational site will enter the data into the eCRF using an access controlled, audit-trailed, ICH/GCP compliant, validated system. Entered data will be subjected to plausibility checks directly implemented in the eCRF, monitoring and medical review. Implausible or missing data will be queried. Database lock will be performed after completion of data entry, data cleaning and a final data review.

7.4.4. Preparation/Handling/Storage/Accountability of biological samples

Biological samples collected under this protocol may be used in accordance with the study informed consent form to conduct protocol related safety and immunogenicity evaluations, exploratory laboratory evaluations related to the SARS-CoV-2 infection the vaccine was designed to prevent, exploratory laboratory evaluations related to vaccine research in general and for research assay validation. All biological samples obtained within the study will be identified solely by means of the individual identification code (Patient ID). Samples will be either processed directly or for PBMC and serum samples for immunogenicity analysis stored until further analyses. Storage of biological samples on a computer will be done in accordance with local data protection law and will be handled in strictest confidence.

For protection of these data, organizational procedures are implemented to prevent distribution of data to unauthorized persons. The appropriate regulations of local data legislation will be fulfilled in its entirety. Samples are stored at the Department of Immunology, Tübingen. Only investigators or their designees will have access to the samples and corresponding data. Sample tracking and preparation will be performed according to established standard operating procedures. The biological samples will be destroyed at the latest 30 years after the end of the study. If a study subject withdraws consent to participate in the study all samples taken and identifiable are destroyed without prior analysis if requested.

7.4.5. Handling of missing data and drop outs

Missing values will be predicted based on plausible assumptions that account for the uncertainty due to missing data. For patients with unknown status for the primary endpoint, i.e. a volunteer without complete follow-up and without any SAE until the last known study site contact, a detailed report on the course should be presented by the investigator and discussed concerning probable unknown SAEs and the reasons for drop-out. If substantial reason will be found that the person could have experienced a SAE, this will be interpreted as failure and the recruitment should be stopped accordingly. Otherwise the safety of the person will be interpreted as success, i.e. the subject will be interpreted to have not experienced a SAE. If this decision cannot be precisely concluded, patient will be considered as drop-out. All missing data or inconsistencies will be resolved by the responsible investigator.

7.4.6. Storage and Archiving of Data

According to the EU Clinical Trial Regulation 536/2014 all essential trial documents (e.g. CRF) will be archived for at least 25 years after the trial termination. The investigator(s) will archive all trial data (source data and Investigator Site File (ISF) including subject identification list and relevant correspondence) according to the Guideline ICH GCP (E6) and to local law or regulations.

8. Statistical Analyses

8.1. Study Population Definition

8.1.1. Sample Size and Power Consideration

In this phase I study the safety/toxicity of one vaccination will be investigated. For this purpose, it will be investigated whether the incidence of severe adverse events (SAE) associated with administration of CoVac-1 exceeds a predetermined rate of 5% (= P1 = alternative hypothesis) in the whole study population. Safety of the CoVac-1 vaccine is shown if no SAE (= P0 = null hypothesis) occurs in the study population. An evaluable sample size of 33 achieves 81.6% power to detect a difference (P1-P0) of 0.0499 using a one-sided exact test based on the binomial distribution with a target significance level of 0.05. The actual significance level achieved by this test is 0.003. These results assume that the population proportion under the null hypotheses (P0) is 0.0001. Assuming a dropout rate of 7.5% (percentage of subjects that are expected to be lost at random during the course of the study and for whom no response data concerning existence of SAE will be collected, i.e. will be treated as “missing”) the total number of 36 subjects should be enrolled in the study in order to end up with 33 evaluable subjects. Sample size computed using PASS 2020 (NCSS, LLC, Kaysville, Utah, USA).

8.2. Analysis Primary Variables

The occurrence of critical events (SAE) associated with administration of CoVac-1 should be reported to the Sponsor (section 9.3.1) and documented immediately in the eCRF (within 48h). The statistical center will evaluate the occurrence of critical events using automatized alerts of the e(CRF) on a daily basis and distribute this information to the Sponsor/DSMB. If one critical event will be observed, the formal statistical stopping rule of the study is reached and no further recruitment is adequate. Otherwise the safety of the procedure will be accepted, if no out of 33 volunteers will experience a critical event.

No further statistical tests with confirmatory aim are planned.

8.3. Analysis Secondary Variables

<u>Safety</u>

The statistical analysis of the secondary endpoint will be done in a descriptive manner. No statistical tests with confirmatory aim are planned. The toxicity and safety will be described by absolute and relative frequencies using CTCAE V5.0-scoring.

Efficacy

The rate of patients with induction of peptide-specific T-cell responses within a maximum of 56 days after vaccination will be the secondary endpoint for efficacy. T-cell responses will be assessed as described in section 6.3.1

The rate of patients with induction of antibody responses within a maximum of 56 days after vaccination will be the secondary endpoint for efficacy. The antibody response will be assessed as described in section 6.3.1

8.4. Subgroup Analysis

Exploratory subgroup analyses are planned for each part (I-III) regarding primary and secondary endpoints.

8.5. Interim Analysis

The primary endpoint will be evaluated in a sequential manner after every consecutive included volunteer has reached day 28. No further formal interim efficacy analysis will be performed during the conduct of the study.

8.6. Stopping Rules

The pre-defined stopping rule for the study is reached if one critical event (SAE as defined in section 9.1.5) associated with administration of CoVac-1 will be observed in the study population resp. if the first critical event will be observed.

The sponsor has the right to terminate the trial prematurely if there are any relevant medical or ethical concerns, or for reasonable administrative reasons. If such action is taken, the reasons for terminating the trial have to be documented in detail. All volunteers who are not considered end of study must undergo a final examination, which must be documented.

Criteria for termination of the study as a whole are:

- An unacceptable profile or incidence rate of adverse events/ adverse events of special interest revealed in this or any other study in which at least one of the investigational products of this trial is administered.
- Significant number of cases of death associated with the study treatment.

- Any other factor that in the view of the sponsor constitutes an adequate reason for terminating the study as a whole.

The Sponsor has to be informed without delay if any investigator has ethical concerns.

8.7. Biometric Report

The biometric report lies within the responsibility of the biostatistician of the clinical trial. The sponsor has to make every effort to acquire a complete data set for statistical analysis. The trial report has to be completed within a reasonable time.

9. Safety

9.1. Definition of Adverse Events and Side Effects

9.1.1. Adverse Events

Any untoward clinical relevant medical occurrence in a volunteer or clinical investigation subject to whom a pharmaceutical product had been administered and which does not necessarily have a causal relationship with this treatment. An AE can therefore be any clinical relevant unfavorable and unintended sign (including an abnormal laboratory finding), clinical relevant symptom, or disease temporally associated with the use of a medicinal (investigational) product, whether or not related to the medicinal (investigational) product.

An AE may be:

- New clinical relevant symptoms/ medical conditions
- New clinical relevant diagnosis
- Clinical relevant changes of laboratory parameters
- Diseases and medical consequences of an accident
- Worsening of medical conditions/ diseases existing before clinical trial start
- Recurrence of disease
- Clinical relevant increase of frequency or intensity of episodical diseases

A pre-existing disease or symptom will not be considered an AE unless there will be an untoward change in its intensity, frequency or quality. This change will be documented by the investigator.

In general, abnormal laboratory findings or clinical events without clinical significance (based on the investigator´s judgement) should not be recorded as AEs.

Surgical procedures themselves are not AEs; they are therapeutic measures for conditions that require surgery. The condition for which the surgery is required may be an AE. Planned surgical measures permitted by the clinical trial protocol and the condition(s) leading to these measures are not AEs, if the condition leading to the measure was present prior to inclusion into the trial.

AEs are classified as "non-serious" or "serious".

9.1.2. Adverse Drug Reaction

An Adverse Drug Reaction (adverse reaction: undesirable effect) is a response to a medicinal product which is noxious and unintended. Adverse reactions may arise from use of the product within or outside terms of the marketing authorisation or from occupational

exposure. Use outside the marketing authorisation includes off-label use, overdose, misuse, abuse and medication errors.

An unexpected Adverse Drug Reaction (ADR) is a reaction which nature or severity is not consistent with the applicable product information available for the IMP.

Expected ADRs arelisted in the appropriate reference documents, e.g. Investigator's Brochures; and below:

A solicited AE/ADR is a predetermined event, which may reflect safety concerns related to the investigational product and is, at least for the local solicited AEs, expected. The solicited ADR/AEs (local and systemic) for this study include:

Local solicited ADRs:

- Swelling at site of injection
- Erythema at site of injection
- Pain at site of injection
- Formation of granuloma at the injection site

Systemic solicited AEs:

- Fever
- Chills
- Myalgia (described to the subject as generalized muscle aches)
- Arthralgia (described to the subject as generalized joint aches)
- Fatigue
- Headache
- Gastrointestinal symptoms (loss of appetite, nausea, vomiting, abdominal pain, and/or diarrhoea)

A grading for severity of ADRs can be found in appendix 14.5.

9.1.3. Expectedness

An 'unexpected' adverse event is one the nature or severity of which is not consistent with the applicable product information, e.g. Investigator's Brochure (IB). Furthermore, reports which add significant information on specificity or severity of a known adverse reaction are counted as 'unexpected' events.

9.1.4. AESI (adverse events of special interest)

An adverse event of special interest (AESI), serious or non-serious, is one of scientific and medical concern specific to the sponsor's product, for which ongoing monitoring and rapid communication (≤ 48 hours) by the investigator to the sponsor can be appropriate. Such an event might warrant further investigation in order to characterize and understand it. Depending on the nature of the event, rapid communication by the trial sponsor to other parties (e.g. regulators) might also be warranted (adapted from CIOMS 2005).

In case of the CoVac-1 vaccine in this study, AESIs include proven SARS-CoV-2 infection and potential immune mediated diseases (pIMDs, see Appendix 14.6)[90]. Instructions for management are provided in section 6.3.

With regard to trial schedule and AESI occurrence, AESIs constitute:

- Novel proven (PCR-based) SARS-CoV-2 infection accompanied by symptoms
- Novel proven (PCR-based) SARS-CoV-2 positivity without symptoms
- Novel potential immune mediated diseases (pIMD) according the listed diseases in Appendix 14.6
- Formation of granuloma at the injection site

AESIs are always to be addressed as part of the patient safety report to the DSMB (section 1.3), also non-occurrence will be mentioned. Depending on the decision of DSMB, the vaccination of further volunteers will be permanently stopped.

9.1.5. Serious Adverse Event and Serous Adverse Reaction

AEs are classified as "non-serious" or "serious".

A serious adverse event (SAE) is one that at any dose:

- Results in death.
- Is life-threatening (the term life-threatening refers to an event in which the subject was at risk of death at the time of event and not to an event which hypothetically might have caused death if it was more severe).
- Requires subject hospitalization or prolongation of existing hospitalization.
- Results in persistent or significant disability/ incapacity.
- Causes a congenital anomaly / birth defect.
- Is medically significant (e.g. suspected transmission of an infectious agent via medicinal product). Moreover, there are other situations - such as important medical events that may not be immediately life threatening or result in death or hospitalization, but may jeopardize the patient or may require intervention to prevent one of the other outcomes listed

above.Important medical event [ICH E2A; EMA/155528/2018]: Examples of such events are intensive treatment in an emergency room or at home for allergic bronchospasm; blood dyscrasias or convulsions that do not result in hospitalisation; development of drug dependency or drug abuse (Important medical event terms list (MedDRA ≥ version 23.0).

9.2. Period of Observation

For the purpose of this trial, the period of observation for collection of AEs extends from the time of administration of the IMP until Visit 5.
All AEs that occur in the course of a clinical trial regardless of the causal relationship must be monitored and followed up until the outcome is known or no more information is achievable.

9.3. Documentation and Reporting of Adverse Events

9.3.1. Documentation and Reporting of Adverse Events by the Investigator

The investigator must document all AEs that occur during the observation period set in this protocol on the pages provided in the case report form. Additional instructions may be provided in the investigator file and in the case report form itself. The following approach will be taken for documentation:
All AEs (whether serious or non-serious) must be documented on the “adverse event” page of the eCRF.
If the AE is serious, the investigator must complete, in addition to the “adverse event” page in the case report form, a “serious adverse event report form” at the time when the SAE is detected. The investigator will document the date when he/she or any employee was first aware of the report. The initial report must be as concise as possible, including reported terms according to “Common Terminology Criteria for Adverse Events (CTCAE)-List” (one term per event), details of the current illness and (S) AE, severity, serious criteria as well as an assessment of the causal relationship between the event and the trial medication.
SAE reports (initial and follow-up reports), even if they are incomplete, should be send within 24 hours upon receipt to representative of the Sponsor:

[redacted]

9.3.2. Assessment of Severity and Causality

The investigator will also provide an assessment of the severity of the event according to CTCAE criteria (Version 5.0) and causal relationship between the event and each of the investigational products or trial procedures.

AEs and SAEs should be evaluated for severity according to the following scale:

- Grade 1 Mild; asymptomatic or mild symptoms; clinical or diagnostic observations only; intervention not indicated.
- Grade 2 Moderate; minimal, local or noninvasive intervention indicated; limiting age-appropriate instrumental Activities of Daily Living (ADL).
- Grade 3 Severe or medically significant but not immediately life-threatening; hospitalization or prolongation of hospitalization indicated; disabling; limiting self care ADL.
- Grade 4 Life-threatening consequences; urgent intervention indicated.
- Grade 5 Death related to AE.

The investigator must determine the causal relationship between the administration of IMP and the occurrence of an AE/SAE as defined below:

Related: There is a reasonable possibility that the SAE may be related to the IMP (e.g. favorable temporal relationship, positive dechallenge: symptoms are receding when IMP is withdrawn or the dose reduced, positive rechallenge: symptoms are reappearing when the IMP is reintroduced or the full dose is re-administered)

Not Related: There is no reasonable possibility that the SAE is related to the IMP (e.g. there is a plausible alternative cause for the SAE that better explains the occurrence of the SAE)

Outcome of AEs

The outcome of an AE at the time of the last observation will be classified as:

**Recovered/ resolved**	All signs and symptoms of an AE disappeared without any sequels at the time of the last interrogation.
**Recovering/ resolving**	The intensity of signs and symptoms has been diminishing and/ or their clinical pattern has been changing up to the time of the last interrogation in a way typical for its resolution.
**Not recovered/ not resolved**	Signs and symptoms of an AE are mostly unchanged at the time of the last interrogation.
**Recovered/**	Actual signs and symptoms of an AE disappeared but there are sequels

**resolved with sequel**	related to the AE.
**Fatal**	Resulting in death. If there are more than one AE, only the AE leading to death (possibly related) will be characterized as 'fatal'.
**Unknown**	The outcome is unknown or implausible and the information cannot be supplemented or verified.

9.3.3. Action taken

No action will be taken with regards to the IMP as the vaccine is applied only once.

9.3.4. Sponsors Assessment of the SAEs

All SAE will be subject to a second assessment by the trial Sponsor or authorized second assessors, e.g. CI.

The second assessor will fill out a 'Second Assessment Form' for each SAE containing.

- Event serious yes/no
- Relationship between SAE and IMP/study procedure
- Expectedness of SAE according to the reference document: IB CoVac-1 peptide vaccine V1.0 dated 22.5.2020.
- Benefit / risk assessment for the trial regarding change as a result of SAE.

9.3.5. Follow-up of Initial Report

Information not available at the time of the initial report (e.g. end date for the AE or laboratory values received after the report) must be documented on a "Serious Adverse Event" form with the box "Follow-up" checked under "Report type".

All volunteers who have AEs, whether considered associated with the use of the investigational products or not, must be monitored to determine the outcome as far as possible. The clinical course of the AE will be followed up according to accepted standards of medical practice even after the end of the period of observation, until a satisfactory explanation is found or the investigator considers it medically justifiable to terminate follow-up. Should the AE result in death, a full pathologist's report should be supplied, if possible.

The sponsor will identify missing information for each SAE report and will require follow up information in regular intervals from the investigators until all queries are resolved or no further information can be reasonably expected. All responses to queries and supply of

additional information by the investigator should follow the same reporting route and timelines as the initial report.

9.3.6. Exception of reporting

As this is a prophylactic vaccination trial with application of CoVac-1 in healthy adults, no exception of reporting for AEs are made.

9.3.7. Suspected Unexpected Serious Adverse Reaction (SUSAR)

SAEs that are both suspected, i.e. possibly related to IMP, and ‘unexpected’, i.e. the nature and/ or severity of which is not consistent with the applicable product information are to be classified as Suspected Unexpected Serious Adverse Reactions (SUSARs).

In case that either the investigator who primarily reported the SAE, or the second assessor classify the SAE as ‘suspected’ (*i.e. not as "definitely not related to IMP”)* and the SAE is also unexpected, it will be categorized as a SUSAR.

All SUSARs are subject to an expedited reporting to the responsible ethics committee(s), the competent higher federal authority (PEI) and to all participating investigators.

9.3.8. Expedited Reporting to the Regulatory Authorities

Fatal and life-threatening SUSARs

The competent authority (PEI) and the EC responsible must be informed by the Sponsor of all fatal or life-threatening SUSARs. This must be done immediately, at the latest seven calendar days after becoming aware of the minimum criteria for reporting. In all cases, attempts must be made to obtain further relevant information, which must be supplied to the competent authority and the EC in overall charge within a further eight days. Furthermore, if a trial subject dies, this information must be additionally passed on to the EC responsible for the region in which the death occurred.

SUSARs that are not fatal or life-threatening

The authority (PEI) and the EC responsible will be informed without delay by the sponsor or CI of all SUSARs, at the latest within 15 calendar days of becoming aware of the minimum criteria for reporting. Further relevant details will be passed on as soon as possible.

If the information at the time of reporting is incomplete, further information to enable adequate assessment of the case will be requested from the reporter or other available sources.

9.4. Examination and Report of Changes in the Risk to Benefit Ratio

Without delay, and at the latest within 15 days of the decision for the need to do so, the Sponsor / CI will inform the competent authority (PEI), the EC responsible of any events or factors that could result in a review of the risk-benefit ratio of the IMP. These consist especially of:

- Individual reports of expected serious ADRs with an unexpected outcome.
- A clinically relevant increase in the rate of occurrence of expected ADRs.
- SUSARs in trial subjects who have already completed the follow-up period of the clinical trial (”end-of-trial visit”).
- Factors emerging in connection with trial conduct or the development of the IMP that may affect the safety of persons concerned.

9.4.1. Reporting to Data and Safety Monitoring Board

The DSMB will be informed of all safety-relevant events by the Sponsor / CI. An interim safety analysis will be sent to the DSMB after completion of Part I and Part II. The DSMB will decide on trial continuation. Additionally, the DSMB will be informed as soon as a IMP-related SAE/SUSAR occurs or a holding rule is reached. Meetings may be convened as conference calls/Emails as well as in person.

9.4.2. Report to the Investigator

The Sponsor / CI will inform investigators of all SUSARs including all relevant further information within the periods set by the authority.

If new information becomes known that is different from the scientific information given to the investigator, all investigators will be informed of this by the sponsor.

9.5. Interim Safety analysis

Two or more interim safety analyses will be undertaken to guide decision and whether to start recruitment in the consecutive trial parts. Upon completion of a study part, screening will be interrupted until safety approval of DSMB is available. The data to be evaluated by the DSMB will include (report):

- Solicited and unsolicited AEs/ADRs, AESIs and SAEs
- Review and, if necessary, assessment of (S)AE relatedness to IMP

The DSMB decision will be documented in a TMF. The information will be distributed to the study sponsor, the drug manufacturer, all investigators/trial site and the ZKS Department Pharmakovigilanz for information.

The interim safety analysis together with the DSMB decision and first data on immunogenicity of CaVac-1 will be send to the authorities (PEI and ethic committee) as a substantial amendment to gain approval for recruiting in Part II and III of the planned study. After responsible authorities approve the submitted documents, the study will continue enrolment as planned.

9.6. Annual Safety Report

Once a year, the Sponsor / CI will supply a report on the safety of trial subjects with all available relevant information concerning volunteer safety during the reference period to the competent authorities. Information required for this purpose will be made available to the ZKS by the Sponsor/ CI at the reporting date. This report will also be supplied to the responsible ethics committee.

The annual safety report will be compiled according to the corresponding ICH guideline E2F „Development Safety Update Report – DSUR“. The safety report will cover all IMPs used in this study.

9.7. Deviations from the Protocol

Any significant deviation from the protocol will be noted.

The PI or a nominated person will evaluate this deviation from the protocol and will decide on the further course of the trial for the respective subject.

9.8. Reporting of Pregnancy

<u>Maternal exposure</u>

If a volunteers becomes pregnant during the course of the study related procedures have to be discontinued immediately.

The outcome of any conception occurring from the date of the vaccination until 1 month after the application should be followed up and documented.

Pregnancy itself is not regarded as an AE unless there is a suspicion that the investigational product under study may have interfered with the effectiveness of a contraceptive

medication. Congenital abnormalities/birth defects and spontaneous miscarriages should be reported and handled as SAEs. Elective abortions without complications should not be handled as AEs. The outcome of all pregnancies (spontaneous miscarriage, elective termination, ectopic pregnancy, normal birth or congenital abnormality) should be followed up and documented even if the patient was withdrawn from the study.

If any pregnancy or suspected pregnancy occurs in the course of the study, it must be reported to ZKS Tübingen, department pharmacovigilance (on behalf of sponsor) immediately by fax (fax-number: + 49 (0)7071 29 25205) or mail (zks-pv@med.uni-tuebingen.de) on the Pregnancy Report Form.

All pregnancies should be followed up and documented, even if the patient was withdrawn from the study, until outcome (spontaneous miscarriage, elective termination, ectopic pregnancy, normal birth or congenital abnormality). The outcome must be notified immediately by the investigator to the ZKS Tübingen, department pharmacovigilance (on behalf of sponsor) within 24 hours of first knowledge as a follow-up to the initial report.

For any event during the pregnancy which meets a seriousness criterion, the Investigator will also follow the procedures for reporting SAEs (complete and send the SAE form to the Sponsor by fax within 24 hours of the Investigator's knowledge of the event).
All neonatal deaths that occur within 30 days of birth should be reported, without regard to causality, as SAEs. In addition, any infant death at any time thereafter that the Investigator suspects is related to the exposure to the study drug/IMPs should also be reported to the Sponsor by facsimile within 24 hours of the Investigators' knowledge of the event.

The same timelines apply when outcome information is available.

If the female is found not to be pregnant, continuation of the volunteer within the study will be determined by the investigator(s).

Paternal exposure

Male patients should refrain from fathering a child or donating sperm during the study and for 3 months following the vaccination.

Pregnancy of the patient’s partners is not considered to be an AE. However, the outcome of all pregnancies (spontaneous miscarriage, elective termination, ectopic pregnancy, normal birth or congenital abnormality) should if possible be followed up and documented.

The outcome of any conception occurring from the date of the first dose until 3 months after the last dose should be followed up and documented.

Information on pregnancy must be collected on the “Pregnancy Reporting Form”. In order for Sponsor or designee to collect any pregnancy surveillance information from the female

partner, the female partner must sign an informed consent form for disclosure of this information.

10. Regulatory Consideration

10.1. Ethical Conduct of Clinical Study

10.1.1. Good Clinical Practice, Declaration of Helsinki and legal Provision

The procedures set out in this trial protocol, pertaining to the conduct, evaluation, and documentation of this trial, are designed to ensure that all persons involved in the trial act according to Good Clinical Practice (GCP) and the ethical principles described in the applicable version of the Declaration of Helsinki.

10.2. Subject Information and Informed Consent

Each volunteer will be informed about the modalities of the clinical study in accordance with the provided volunteer informed consent (IC). The volunteer is to be informed both in writing and verbally by the investigator before any study-specific procedure is performed. The volunteer must be given sufficient time to decide whether to participate in this comparative study and to ask questions concerning this trial. It must also be made clear to the volunteer that he / she can withdraw from the study at any time without giving reasons and that he / she will not be in any way disadvantaged for this. The subject must give consent in writing. The volunteer and informing physician must each personally date and sign the informed consent form with an integrated declaration on data privacy protection, whereby the physician must not sign before the volunteer. Original signed documents will be part of the investigator’s file and retained with it. A copy of the signed informed consent document and study insurance policy must be given to the subject. The documents must be in a language understandable to the subject and must specify who informed the subject. The subjects will be informed as soon as possible if new information may influence his/her decision to participate in the trial. The communication of this information should be documented in the volunteer chart.

10.3. Insurance

Each volunteer is insured against any health impairment occurring as a result of participation in the study in accordance with the laws and regulations of the “German Arzneimittelgesetz”. The insurance is covered by *HDI Global SE, Am Schönenkamp 45, 40599 Düsseldorf, Policy* number 57 010311 03013/03052 and valid throughout the conduct of the study including follow-up for each individual volunteer. A copy of the insurance policy and conditions are distributed to the volunteer upon enrolment into the study and the volunteer is advised to adhere to the conditions of the insurance policy to safeguard a valid volunteer insurance.

Travel insurance will be included for all volunteers enrolled in the clinical trial.

10.4. Confidentiality

The data obtained in the course of the trial will be treated according to the European General Data Protection Regulation (Datenschutz-Grundverordnung; DS-GVO) and the applicable local data protection regulations as well as the AMG.
Subjects have to be informed about data protection in the clinical trial and to consent in writing to collect and process their personalized data as well as to transfer their pseudonymized data. The information has to be transparent, precise, easily accessible and understandable and is written in clear and simple language. The written privacy policy must be approved by the responsible ethics committee.

In order to maintain volunteer privacy, all data capture records, study drug accountability records, study reports and communications will identify the volunteer by the assigned volunteer number. The PI determines which persons are authorized to view personal data, the Volunteer Intification Log is only accessible to authorized study team members. Access rights to personal data (including pseudonymised data) are available to prevent unauthorized access to the data (both electronically and physically). Electronic systems and files are access-regulated, possibly password-protected. Documents and files are kept in lockable rooms, if necessary, cupboards with access control.

The volunteer name, initials and the full birth date should never be used in any correspondence with the Sponsor or on the Case Report Forms. The investigator will grant monitor(s) and auditor(s) and/or regulatory authorities direct access to the volunteer's original medical records for verification of data gathered on the data capture records and to audit the data collection process. Direct access includes examining, analyzing, and verifying any recorded data and reports that are important to the evaluation of the monitoring. The investigator is obliged to inform the volunteer that his/her trial-related records will be viewed without violating their confidentiality and that the collected information will only be made publicly available to the extent permitted by the applicable laws and regulations. All data will be stored either paper-based or electronically in a pseudonymous manner and handled strictly confidential. The investigators are obliged to keep all study data and information confidential and to use those data only in context with the persons involved in the trial conduct. Study material or information developed in this trial must not be available to third parties, except for official representatives of the sponsor or regulatory authorities.

Data will be processed at the study site according to the written safety concept of this institution. Access to the data will be strictly limited to authorized persons. Loss of data is excluded due to extensive back-up procedures. All legal requirements concerning data protection and confidentiality will be respected. All authorized persons are sworn to secrecy.
In the case of withdrawal of consent the stored data collected to this time point will be stored and further used. Data not necessary any longer are deleted immediately.
Collected study data will be stored for at least 25 years after the end of the trial, if there are no other regulatory archiving periods. After archiving has expired, the data will be destructed in a data protection compliant manner.

When processing personal data, the following principles must be observed (pursuant to DS-GVO Article 5 “Principles relating to processing of personal data“):

Personal data shall be:

- processed lawfully, fairly and in a transparent manner in relation to the data subject
- collected for specified, explicit and legitimate purposes and not further processed in a manner that is incompatible with those purposes
- adequate, relevant and limited to what is necessary in relation to the purposes for which they are processed
- accurate and, where necessary, kept up to date
- kept in a form which permits identification of data subjects for no longer than is necessary for the purposes for which the personal data are processed
- processed in a manner that ensures appropriate security of the personal data, including protection against unauthorised or unlawful processing and against accidental loss, destruction or damage, using appropriate technical or organisational measures

10.5. Responsibility of the the Investigator

The investigator should ensure that all persons assisting with the trial are adequately informed about the protocol, any amendments to the protocol, the trial treatments, and their trial-related duties and functions.
The investigator should maintain a list of subinvestigators and other appropriately qualified persons to whom he or she has delegated significant trial-related duties.

10.6. Registration of the Trial

Prior to the beginning of the clinical phase (First Patient In) the Sponsor / CI will register the trial in the EudraCT (2020-002502-75) as well as ClinicalTrials.gov Database.

10.7. Continuous Information to Independent Ethics Committee

According to the German Drug Law (AMG) and the GCP Ordinance, the EC and the competent authority (Paul-Ehrlich Institut, PEI) will be informed of all suspected serious unexpected adverse reactions (SUSARs). Both institutions will be informed in case the risk/ benefit assessment did change or any others new and significant hazards for subjects' safety or welfare did occur. In addition, upon activation and prior to discontinuation of a holding rule the sponsor informs the responsible authorities (section 6.5). Furthermore, a report on all observed SAEs will be submitted once a year – Annual Safety Report.
The EC and the regulatory authorities must be informed of the end of the trial. They will be provided with a summary of trial results within one year after the end of clinical phase.

10.8. Approval of Protocol and Subsequent Amendments

Before the start of the trial, the trial protocol, informed consent document, and any other appropriate documents will be submitted to the independent EC as well as to the competent authority (PEI). A written favourable vote of the EC and an (implicit) approval by the competent higher federal authority (PEI) as well as the notification of the local authorities (acc. to §67 AMG) are a prerequisite for initiation of this clinical trial. Before the first subject is enrolled in the trial, all ethical and legal requirements must be met. All planned substantial changes (see §10, (1) of German GCP-Regulation) will be submitted for approval to EC and the competent authority in writing as protocol amendments.

11. Publications

11.1. Reports

Within one year of the completion of the trial, the competent authority and the ethics committee will be supplied with a summary of the final report on the clinical trial containing the principle results.

All reports to the sponsor will be written in English language. All clinical, analytical and statistical results will be presented in a final clinical trial report (CTR). The outline of this report will accord to the ICH Topic E3.

11.2. Publication

The final results of this study will be presented at scientific meetings and published in a peer reviewed journal. All publications on result of this study should be based on the scientific reports (see 11.1) and are the responsibility of the CI. The authorship will reflect the contributions of each collaborating centre. Any publication, abstract or presentation based on patients included in this study must be approved by the CI. First safety data will be published after completion of EOSf of the last patient enrolled in the clinical trial.

No publications on planned or unplanned interim analyses (e.g. safety analysis for DSMB or provisionally results on immunological efficacy before finalization of the scientific reports) are allowed.

12. Financing

This study is financed by the “Sonderfördermaßnahme COVID-19” of the ministry of science, research and art of the state Baden-Wuerttemberg, Germany.

13. Literature

1. Mo P, Xing Y, Xiao Y, et al. Clinical characteristics of refractory COVID-19 pneumonia in Wuhan, China. *Clin Infect Dis* 2020 doi: 10.1093/cid/ciaa270 [published Online First: 2020/03/17]
2. Ng OT, Marimuthu K, Chia PY, et al. SARS-CoV-2 Infection among Travelers Returning from Wuhan, China. *N Engl J Med* 2020 doi: 10.1056/NEJMc2003100 [published Online First: 2020/03/13]
3. Khan S, Siddique R, Shereen MA, et al. The emergence of a novel coronavirus (SARS-CoV-2), their biology and therapeutic options. *J Clin Microbiol* 2020 doi: 10.1128/JCM.00187-20 [published Online First: 2020/03/13]
4. Organization WH. Report of the WHO-China Joint Mission on Coronavirus Disease 2019. 2020
5. Seder RA, Darrah PA, Roederer M. T-cell quality in memory and protection: implications for vaccine design. *Nat Rev Immunol* 2008;8(4):247-58. doi: 10.1038/nri2274 [published Online First: 2008/03/08]
6. Khan N, Best D, Bruton R, et al. T cell recognition patterns of immunodominant cytomegalovirus antigens in primary and persistent infection. *J Immunol* 2007;178(7):4455-65. doi: 10.4049/jimmunol.178.7.4455 [published Online First: 2007/03/21]
7. Hill GR, Tey SK, Beagley L, et al. Successful immunotherapy of HCMV disease using virus-specific T cells expanded from an allogeneic stem cell transplant recipient. *Am J Transplant* 2010;10(1):173-9. doi: 10.1111/j.1600-6143.2009.02872.x [published Online First: 2009/11/19]
8. Feucht J, Joachim L, Lang P, et al. Adoptive T-cell transfer for refractory viral infections with cytomegalovirus, Epstein-Barr virus or adenovirus after allogeneic stem cell transplantation. *Klin Padiatr* 2013;225(3):164-9. doi: 10.1055/s-0033-1333749 [published Online First: 2013/05/24]
9. Hanajiri R, Sani GM, Hanley PJ, et al. Generation of Zika virus-specific T cells from seropositive and virus-naive donors for potential use as an autologous or "off-the-shelf" immunotherapeutic. *Cytotherapy* 2019;21(8):840-55. doi: 10.1016/j.jcyt.2019.06.008 [published Online First: 2019/07/08]
10. Wisskirchen K, Kah J, Malo A, et al. T cell receptor grafting allows virological control of Hepatitis B virus infection. *J Clin Invest* 2019;129(7):2932-45. doi: 10.1172/JCI120228 [published Online First: 2019/05/01]
11. Hanajiri R, Sani GM, Saunders D, et al. Generation of Norovirus-Specific T Cells From Human Donors With Extensive Cross-Reactivity to Variant Sequences: Implications for Immunotherapy. *J Infect Dis* 2020;221(4):578-88. doi: 10.1093/infdis/jiz491 [published Online First: 2019/09/29]
12. Channappanavar R, Fett C, Zhao J, et al. Virus-specific memory CD8 T cells provide substantial protection from lethal severe acute respiratory syndrome coronavirus infection. *J Virol* 2014;88(19):11034-44. doi: 10.1128/JVI.01505-14 [published Online First: 2014/07/25]
13. Channappanavar R, Zhao J, Perlman S. T cell-mediated immune response to respiratory coronaviruses. *Immunol Res* 2014;59(1-3):118-28. doi: 10.1007/s12026-014-8534-z [published Online First: 2014/05/23]
14. Zhao J, Zhao J, Mangalam AK, et al. Airway Memory CD4(+) T Cells Mediate Protective Immunity against Emerging Respiratory Coronaviruses. *Immunity* 2016;44(6):1379-91. doi: 10.1016/j.immuni.2016.05.006 [published Online First: 2016/06/12]
15. Zhao J, Zhao J, Perlman S. T cell responses are required for protection from clinical disease and for virus clearance in severe acute respiratory syndrome coronavirus-infected mice. *J Virol* 2010;84(18):9318-25. doi: 10.1128/JVI.01049-10 [published Online First: 2010/07/09]

16. Liu L, Wei Q, Lin Q, et al. Anti-spike IgG causes severe acute lung injury by skewing macrophage responses during acute SARS-CoV infection. *JCI Insight* 2019;4(4) doi: 10.1172/jci.insight.123158 [published Online First: 2019/03/05]
17. Tang F, Quan Y, Xin ZT, et al. Lack of peripheral memory B cell responses in recovered patients with severe acute respiratory syndrome: a six-year follow-up study. *J Immunol* 2011;186(12):7264-8. doi: 10.4049/jimmunol.0903490 [published Online First: 2011/05/18]
18. Liu WJ, Zhao M, Liu K, et al. T-cell immunity of SARS-CoV: Implications for vaccine development against MERS-CoV. *Antiviral Res* 2017;137:82-92. doi: 10.1016/j.antiviral.2016.11.006 [published Online First: 2016/11/15]
19. Grifoni A, Weiskopf D, Ramirez SI, et al. Targets of T Cell Responses to SARS-CoV-2 Coronavirus in Humans with COVID-19 Disease and Unexposed Individuals. *Cell* 2020 doi: 10.1016/j.cell.2020.05.015 [published Online First: 2020/05/31]
20. Braun J, Loyal L, Frentsch M, et al. Presence of SARS-CoV-2 reactive T cells in COVID-19 patients and healthy donors. *medRxiv* 2020:2020.04.17.20061440. doi: 10.1101/2020.04.17.20061440
21. Vali B, Tohn R, Cohen MJ, et al. Characterization of cross-reactive CD8+ T-cell recognition of HLA-A2-restricted HIV-Gag (SLYNTVATL) and HCV-NS5b (ALYDVVSKL) epitopes in individuals infected with human immunodeficiency and hepatitis C viruses. *J Virol* 2011;85(1):254-63. doi: 10.1128/JVI.01743-10 [published Online First: 2010/10/29]
22. Acierno PM, Newton DA, Brown EA, et al. Cross-reactivity between HLA-A2-restricted FLU-M1:58-66 and HIV p17 GAG:77-85 epitopes in HIV-infected and uninfected individuals. *J Transl Med* 2003;1(1):3. doi: 10.1186/1479-5876-1-3 [published Online First: 2003/10/07]
23. Clute SC, Watkin LB, Cornberg M, et al. Cross-reactive influenza virus-specific CD8+ T cells contribute to lymphoproliferation in Epstein-Barr virus-associated infectious mononucleosis. *J Clin Invest* 2005;115(12):3602-12. doi: 10.1172/JCI25078 [published Online First: 2005/11/26]
24. Nelde A, Bilich T, Heitmann JS, et al. SARS-CoV-2 T-cell epitopes define heterologous and COVID-19-induced T-cell recognition. In: Preprint, ed. Research Square, 2020.
25. Deres K, Schild H, Wiesmuller KH, et al. In vivo priming of virus-specific cytotoxic T lymphocytes with synthetic lipopeptide vaccine. *Nature* 1989;342(6249):561-4. doi: 10.1038/342561a0 [published Online First: 1989/11/30]
26. Falk K, Rotzschke O, Rammensee HG. Cellular peptide composition governed by major histocompatibility complex class I molecules. *Nature* 1990;348(6298):248-51. doi: 10.1038/348248a0 [published Online First: 1990/11/15]
27. Rammensee HG. Survival of the fitters. *Nature* 2002;419(6906):443-5. doi: 10.1038/419443a [published Online First: 2002/10/09]
28. Rammensee H, Bachmann J, Emmerich NP, et al. SYFPEITHI: database for MHC ligands and peptide motifs. *Immunogenetics* 1999;50(3-4):213-9. doi: 10.1007/s002510050595 [published Online First: 1999/12/22]
29. Bilich T, Nelde A, Bichmann L, et al. The HLA ligandome landscape of chronic myeloid leukemia delineates novel T-cell epitopes for immunotherapy. *Blood* 2019;133(6):550-65. doi: 10.1182/blood-2018-07-866830 [published Online First: 2018/12/12]
30. Lubke M, Spalt S, Kowalewski DJ, et al. Identification of HCMV-derived T cell epitopes in seropositive individuals through viral deletion models. *J Exp Med* 2020;217(3) doi: 10.1084/jem.20191164 [published Online First: 2019/12/24]
31. Berlin C, Kowalewski DJ, Schuster H, et al. Mapping the HLA ligandome landscape of acute myeloid leukemia: a targeted approach toward peptide-based immunotherapy. *Leukemia* 2015;29(3):647-59. doi: 10.1038/leu.2014.233 [published Online First: 2014/08/06]
32. Kowalewski DJ, Schuster H, Backert L, et al. HLA ligandome analysis identifies the underlying specificities of spontaneous antileukemia immune responses in chronic

lymphocytic leukemia (CLL). *Proc Natl Acad Sci U S A* 2015;112(2):E166-75. doi: 10.1073/pnas.1416389112
33. Nastke MD, Herrgen L, Walter S, et al. Major contribution of codominant CD8 and CD4 T cell epitopes to the human cytomegalovirus-specific T cell repertoire. *Cell Mol Life Sci* 2005;62(1):77-86. doi: 10.1007/s00018-004-4363-x [published Online First: 2004/12/25]
34. Icheva V, Kayser S, Wolff D, et al. Adoptive transfer of epstein-barr virus (EBV) nuclear antigen 1-specific t cells as treatment for EBV reactivation and lymphoproliferative disorders after allogeneic stem-cell transplantation. *J Clin Oncol* 2013;31(1):39-48. doi: 10.1200/JCO.2011.39.8495 [published Online First: 2012/11/22]
35. Hilf N, Kuttruff-Coqui S, Frenzel K, et al. Actively personalized vaccination trial for newly diagnosed glioblastoma. *Nature* 2019;565(7738):240-45. doi: 10.1038/s41586-018-0810-y [published Online First: 2018/12/21]
36. Baumgaertner P, Jandus C, Rivals JP, et al. Vaccination-induced functional competence of circulating human tumor-specific CD8 T-cells. *Int J Cancer* 2012;130(11):2607-17. doi: 10.1002/ijc.26297 [published Online First: 2011/07/29]
37. Freund J. The effect of paraffin oil and mycobacteria on antibody formation and sensitization; a review. *Am J Clin Pathol* 1951;21(7):645-56. doi: 10.1093/ajcp/21.7.645 [published Online First: 1951/07/01]
38. Rammensee HG, Wiesmuller KH, Chandran PA, et al. A new synthetic toll-like receptor 1/2 ligand is an efficient adjuvant for peptide vaccination in a human volunteer. *J Immunother Cancer* 2019;7(1):307. doi: 10.1186/s40425-019-0796-5 [published Online First: 2019/11/16]
39. Alam I, Goldeck D, Larbi A, et al. Aging affects the proportions of T and B cells in a group of elderly men in a developing country--a pilot study from Pakistan. *Age (Dordr)* 2013;35(5):1521-30. doi: 10.1007/s11357-012-9455-1 [published Online First: 2012/07/20]
40. Fulop T, Larbi A, Pawelec G. Human T cell aging and the impact of persistent viral infections. *Front Immunol* 2013;4:271. doi: 10.3389/fimmu.2013.00271 [published Online First: 2013/09/26]
41. Lambkin R, Novelli P, Oxford J, et al. Human genetics and responses to influenza vaccination: clinical implications. *Am J Pharmacogenomics* 2004;4(5):293-8. doi: 10.2165/00129785-200404050-00002 [published Online First: 2004/10/07]
42. Molano A, Park SH, Chiu YH, et al. Cutting edge: the IgG response to the circumsporozoite protein is MHC class II-dependent and CD1d-independent: exploring the role of GPIs in NK T cell activation and antimalarial responses. *J Immunol* 2000;164(10):5005-9. doi: 10.4049/jimmunol.164.10.5005 [published Online First: 2000/05/09]
43. Oliveira GA, Kumar KA, Calvo-Calle JM, et al. Class II-restricted protective immunity induced by malaria sporozoites. *Infect Immun* 2008;76(3):1200-6. doi: 10.1128/IAI.00566-07 [published Online First: 2007/12/28]
44. Xu R, Johnson AJ, Liggitt D, et al. Cellular and humoral immunity against vaccinia virus infection of mice. *J Immunol* 2004;172(10):6265-71. doi: 10.4049/jimmunol.172.10.6265 [published Online First: 2004/05/07]
45. Sette A, Moutaftsi M, Moyron-Quiroz J, et al. Selective CD4+ T cell help for antibody responses to a large viral pathogen: deterministic linkage of specificities. *Immunity* 2008;28(6):847-58. doi: 10.1016/j.immuni.2008.04.018 [published Online First: 2008/06/14]
46. Shedlock DJ, Shen H. Requirement for CD4 T cell help in generating functional CD8 T cell memory. *Science* 2003;300(5617):337-9. doi: 10.1126/science.1082305 [published Online First: 2003/04/12]
47. Carvalho LH, Sano G, Hafalla JC, et al. IL-4-secreting CD4+ T cells are crucial to the development of CD8+ T-cell responses against malaria liver stages. *Nature medicine* 2002;8(2):166-70. doi: 10.1038/nm0202-166 [published Online First: 2002/02/01]

48. Kemball CC, Pack CD, Guay HM, et al. The antiviral CD8+ T cell response is differentially dependent on CD4+ T cell help over the course of persistent infection. *J Immunol* 2007;179(2):1113-21. doi: 10.4049/jimmunol.179.2.1113 [published Online First: 2007/07/10]
49. Marzo AL, Vezys V, Klonowski KD, et al. Fully functional memory CD8 T cells in the absence of CD4 T cells. *J Immunol* 2004;173(2):969-75. doi: 10.4049/jimmunol.173.2.969 [published Online First: 2004/07/09]
50. van de Berg PJ, van Leeuwen EM, ten Berge IJ, et al. Cytotoxic human CD4(+) T cells. *Curr Opin Immunol* 2008;20(3):339-43. doi: 10.1016/j.coi.2008.03.007 [published Online First: 2008/04/29]
51. Johnson AJ, Chu CF, Milligan GN. Effector CD4+ T-cell involvement in clearance of infectious herpes simplex virus type 1 from sensory ganglia and spinal cords. *J Virol* 2008;82(19):9678-88. doi: 10.1128/JVI.01159-08 [published Online First: 2008/08/01]
52. Elyaman W, Kivisakk P, Reddy J, et al. Distinct functions of autoreactive memory and effector CD4+ T cells in experimental autoimmune encephalomyelitis. *Am J Pathol* 2008;173(2):411-22. doi: 10.2353/ajpath.2008.080142 [published Online First: 2008/06/28]
53. Tsuji M, Romero P, Nussenzweig RS, et al. CD4+ cytolytic T cell clone confers protection against murine malaria. *J Exp Med* 1990;172(5):1353-7. doi: 10.1084/jem.172.5.1353 [published Online First: 1990/11/01]
54. Harrington LE, Mangan PR, Weaver CT. Expanding the effector CD4 T-cell repertoire: the Th17 lineage. *Curr Opin Immunol* 2006;18(3):349-56. doi: 10.1016/j.coi.2006.03.017 [published Online First: 2006/04/18]
55. Long QX, Tang XJ, Shi QL, et al. Clinical and immunological assessment of asymptomatic SARS-CoV-2 infections. *Nature medicine* 2020;26(8):1200-04. doi: 10.1038/s41591-020-0965-6 [published Online First: 2020/06/20]
56. Kreer C, Zehner M, Weber T, et al. Longitudinal Isolation of Potent Near-Germline SARS-CoV-2-Neutralizing Antibodies from COVID-19 Patients. *Cell* 2020 doi: 10.1016/j.cell.2020.06.044 [published Online First: 2020/07/17]
57. Steere AC, Sikand VK, Meurice F, et al. Vaccination against Lyme disease with recombinant Borrelia burgdorferi outer-surface lipoprotein A with adjuvant. Lyme Disease Vaccine Study Group. *N Engl J Med* 1998;339(4):209-15. doi: 10.1056/NEJM199807233390401 [published Online First: 1998/07/23]
58. Sigal LH, Zahradnik JM, Lavin P, et al. A vaccine consisting of recombinant Borrelia burgdorferi outer-surface protein A to prevent Lyme disease. Recombinant Outer-Surface Protein A Lyme Disease Vaccine Study Consortium. *N Engl J Med* 1998;339(4):216-22. doi: 10.1056/NEJM199807233390402 [published Online First: 1998/07/23]
59. Opie EL, Freund J. An Experimental Study of Protective Inoculation with Heat Killed Tubercle Bacilli. *J Exp Med* 1937;66(6):761-88. doi: 10.1084/jem.66.6.761 [published Online First: 1937/11/30]
60. Jensen FC, Savary JR, Diveley JP, et al. Adjuvant activity of incomplete Freund's adjuvant. *Adv Drug Deliv Rev* 1998;32(3):173-86. doi: 10.1016/s0169-409x(98)00009-x [published Online First: 2000/06/06]
61. Rammensee HG, Stevanovic S, Gouttefangeas C, et al. Designing a therapeutic SARS-CoV-2 T-cell-inducing vaccine for high-risk patient groups. *Research Square [preprint]* 2020 doi:

10.21203/rs.3.rs-27316/v1
62. Kran AM, Sorensen B, Nyhus J, et al. HLA- and dose-dependent immunogenicity of a peptide-based HIV-1 immunotherapy candidate (Vacc-4x). *AIDS* 2004;18(14):1875-83.
63. Feyerabend S, Stevanovic S, Gouttefangeas C, et al. Novel multi-peptide vaccination in Hla-A2+ hormone sensitive patients with biochemical relapse of prostate cancer.

Prostate 2009;69(9):917-27. doi: 10.1002/pros.20941 [published Online First: 2009/03/10]
64. Sato Y, Shomura H, Maeda Y, et al. Immunological evaluation of peptide vaccination for patients with gastric cancer based on pre-existing cellular response to peptide. *Cancer Sci* 2003;94(9):802-8.
65. Noguchi M, Kobayashi K, Suetsugu N, et al. Induction of cellular and humoral immune responses to tumor cells and peptides in HLA-A24 positive hormone-refractory prostate cancer patients by peptide vaccination. *Prostate* 2003;57(1):80-92. doi: 10.1002/pros.10276
66. Atsmon J, Kate-Ilovitz E, Shaikevich D, et al. Safety and immunogenicity of multimeric-001--a novel universal influenza vaccine. *J Clin Immunol* 2012;32(3):595-603. doi: 10.1007/s10875-011-9632-5 [published Online First: 2012/02/10]
67. Salk JE, Bailey ML, Laurent AM. The use of adjuvants in studies on influenza immunization. II. Increased antibody formation in human subjects inoculated with influenza virus vaccine in a water in-oil emulsion. *Am J Hyg* 1952;55(3):439-56. doi: 10.1093/oxfordjournals.aje.a119534 [published Online First: 1952/05/01]
68. Meiklejohn G. Adjuvant influenza adenovirus vaccine. *JAMA* 1962;179:594-7. doi: 10.1001/jama.1962.03050080006002 [published Online First: 1962/02/24]
69. Stern LJ, Calvo-Calle JM. HLA-DR: molecular insights and vaccine design. *Curr Pharm Des* 2009;15(28):3249-61. doi: 10.2174/138161209789105171 [published Online First: 2009/10/29]
70. Herrington DA, Clyde DF, Losonsky G, et al. Safety and immunogenicity in man of a synthetic peptide malaria vaccine against Plasmodium falciparum sporozoites. *Nature* 1987;328(6127):257-9. doi: 10.1038/328257a0 [published Online First: 1987/07/16]
71. Weihrauch MR, Ansen S, Jurkiewicz E, et al. Phase I/II combined chemoimmunotherapy with carcinoembryonic antigen-derived HLA-A2-restricted CAP-1 peptide and irinotecan, 5-fluorouracil, and leucovorin in patients with primary metastatic colorectal cancer. *Clinical cancer research : an official journal of the American Association for Cancer Research* 2005;11(16):5993-6001. doi: 10.1158/1078-0432.CCR-05-0018
72. Peoples GE, Gurney JM, Hueman MT, et al. Clinical trial results of a HER2/neu (E75) vaccine to prevent recurrence in high-risk breast cancer patients. *J Clin Oncol* 2005;23(30):7536-45. doi: 10.1200/JCO.2005.03.047
73. Walter S, Weinschenk T, Stenzl A, et al. Multipeptide immune response to cancer vaccine IMA901 after single-dose cyclophosphamide associates with longer patient survival. *Nature medicine* 2012 doi: 10.1038/nm.2883 [published Online First: 2012/07/31]
74. Mailander V, Scheibenbogen C, Thiel E, et al. Complete remission in a patient with recurrent acute myeloid leukemia induced by vaccination with WT1 peptide in the absence of hematological or renal toxicity. *Leukemia* 2004;18(1):165-6. doi: 10.1038/sj.leu.2403186
75. Oka Y, Tsuboi A, Taguchi T, et al. Induction of WT1 (Wilms' tumor gene)-specific cytotoxic T lymphocytes by WT1 peptide vaccine and the resultant cancer regression. *Proc Natl Acad Sci U S A* 2004;101(38):13885-90. doi: 10.1073/pnas.0405884101
76. Van Tendeloo VF, Van de Velde A, Van Driessche A, et al. Induction of complete and molecular remissions in acute myeloid leukemia by Wilms' tumor 1 antigen-targeted dendritic cell vaccination. *Proc Natl Acad Sci U S A* 2010;107(31):13824-9. doi: 10.1073/pnas.1008051107
77. Schmitt M, Schmitt A, Rojewski MT, et al. RHAMM-R3 peptide vaccination in patients with acute myeloid leukemia, myelodysplastic syndrome, and multiple myeloma elicits immunologic and clinical responses. *Blood* 2008;111(3):1357-65. doi: 10.1182/blood-2007-07-099366 [published Online First: 2007/11/06]
78. Schwartzentruber DJ, Lawson DH, Richards JM, et al. gp100 peptide vaccine and interleukin-2 in patients with advanced melanoma. *N Engl J Med* 2011;364(22):2119-27. doi: 10.1056/NEJMoa1012863

79. Mittendorf EA, Clifton GT, Holmes JP, et al. Clinical trial results of the HER-2/neu (E75) vaccine to prevent breast cancer recurrence in high-risk patients: from US Military Cancer Institute Clinical Trials Group Study I-01 and I-02. *Cancer* 2012;118(10):2594-602. doi: 10.1002/cncr.26574 [published Online First: 2011/10/13]
80. Weinschenk T, Gouttefangeas C, Schirle M, et al. Integrated functional genomics approach for the design of patient-individual antitumor vaccines. *Cancer Res* 2002;62(20):5818-27. [published Online First: 2002/10/18]
81. Honda-Okubo Y, Barnard D, Ong CH, et al. Severe acute respiratory syndrome-associated coronavirus vaccines formulated with delta inulin adjuvants provide enhanced protection while ameliorating lung eosinophilic immunopathology. *J Virol* 2015;89(6):2995-3007. doi: 10.1128/JVI.02980-14 [published Online First: 2014/12/19]
82. Graham BS. Rapid COVID-19 vaccine development. *Science* 2020 doi: 10.1126/science.abb8923 [published Online First: 2020/05/10]
83. van Doorn E, Liu H, Huckriede A, et al. Safety and tolerability evaluation of the use of Montanide ISA51 as vaccine adjuvant: A systematic review. *Hum Vaccin Immunother* 2016;12(1):159-69. doi: 10.1080/21645515.2015.1071455
84. Van den Heuvel MM, Burgers SA, van Zandwijk N. Immunotherapy in non-small-cell lung carcinoma: from inflammation to vaccination. *Clinical lung cancer* 2009;10(2):99-105. doi: 10.3816/CLC.2009.n.012
85. Wu Y, Ellis RD, Shaffer D, et al. Phase 1 trial of malaria transmission blocking vaccine candidates Pfs25 and Pvs25 formulated with montanide ISA 51. *PLoS One* 2008;3(7):e2636. doi: 10.1371/journal.pone.0002636
86. Carr A, Rodriguez E, Arango Mdel C, et al. Immunotherapy of advanced breast cancer with a heterophilic ganglioside (NeuGcGM3) cancer vaccine. *J Clin Oncol* 2003;21(6):1015-21. doi: 10.1200/JCO.2003.02.124
87. Widenmeyer M, Griesemann H, Stevanovic S, et al. Promiscuous survivin peptide induces robust CD4+ T-cell responses in the majority of vaccinated cancer patients. *Int J Cancer* 2012;131(1):140-9. doi: 10.1002/ijc.26365 [published Online First: 2011/08/23]
88. Britten CM, Gouttefangeas C, Welters MJ, et al. The CIMT-monitoring panel: a two-step approach to harmonize the enumeration of antigen-specific CD8+ T lymphocytes by structural and functional assays. *Cancer Immunol Immunother* 2008;57(3):289-302. doi: 10.1007/s00262-007-0378-0 [published Online First: 2007/08/28]
89. Neumann A, Horzer H, Hillen N, et al. Identification of HLA ligands and T-cell epitopes for immunotherapy of lung cancer. *Cancer Immunol Immunother* 2013;62(9):1485-97. doi: 10.1007/s00262-013-1454-2 [published Online First: 2013/07/03]
90. Tavares Da Silva F, De Keyser F, Lambert PH, et al. Optimal approaches to data collection and analysis of potential immune mediated disorders in clinical trials of new vaccines. *Vaccine* 2013;31(14):1870-6. doi: 10.1016/j.vaccine.2013.01.042 [published Online First: 2013/02/09]

Regulatory References

Medicinal Products Act (Arzneimittelgesetz), published on 12 December 2005 (Federal Law Gazette [BGBl.] Part I p. 3394), last amended by Article 3 of the Law of 18 July 2017 (BGBl. I p. 2757)

Ordinance on the implementation of Good Clinical Practice in the conduct of clinical trials on medicinal products for use in humans (GCP Ordinance - GCP-V), published on 09 August 2004 (Federal Law Gazette (BGBl.) I p. 2081), last amended by Article 8 of the Law of 19. Oktober 2012 (BGBl. I S. 2192)

REGULATION (EU) No 536/2014 OF THE EUROPEAN PARLIAMENT AND OF THE COUNCIL of 16 April 2014 on clinical trials on medicinal products for human use, and repealing Directive 2001/20/EC

Medical Devices Act (Medizinproduktegesetz), published on 07.August 2002 (Federal Law Gazette (BGBl.) I p. 3146), last amended by Article 7 of the Law of 18 July 2017 (BGBl. I p. 2757)

ICH Topic E3, Note for Guidance on Structure and Content of Clinical Study Reports (CPMP/ICH/137/95), July 1996

ICH Topic E 6 (R2), Note for Guidance on Good Clinical Practice (CPMP/ICH/135/95), December 2016

ICH Topic E 8, Note for Guidance on General Considerations for Clinical Trials (CPMP/ICH/291/95), March 1998

Clinical Trial Facilitation Group, Recommendations related to contraception and pregnancy testing in clinical trials, 15.09.2014)

EMEA-Guideline On Data Monitoring Committees (EMEA/CHMP/EWP/5872/03 Corr), January 2006

REGULATION (EU) 2016/679 OF THE EUROPEAN PARLIAMENT AND OF THE COUNCIL of 27 April 2016 on the protection of natural persons with regard to the processing of personal data and on the free movement of such data, and repealing Directive 95/46/EC (General Data Protection Regulation)

14. Appendix

14.1. Common Terminology Criteria for Adverse Events (CTCAE) Version

https://ctep.cancer.gov/protocoldevelopment/electronic_applications/docs/CTCAE_v5_Quick_Reference_5x7.pdf

14.2. List of central laboratories

[redacted]

[redacted]

[redacted]

14.3. Volunteer diary

Studie	P-pVAC-SARS-CoV-2
Probanden-ID *(vom Arzt auszufüllen)*:	[_ _ _ _] - [_ _ _]
Datum der Impfung:	[_ _] [_ _] [20_ _]

1. Richtlinien

Füllen Sie Ihr Tagebuch (**täglich**) mit Ankreuzen und gegebenenfalls weiteren Ergänzungen aus. Falls Sie eine Frage nicht beantworten können, streichen Sie diese bitte durch. Falls Sie Fragen mit „Ja“ beantworten, füllen Sie bitte weitere Angaben aus. Bei Rückfragen oder starken Beschwerden, melden Sie sich bitte an Ihrem Prüfzentrum.

2. Tag der Impfung (d1)

		Ja	Nein	Weitere Angaben
1.	Haben Sie Schmerzen an der Impfstelle?	☐	☐	______________________
2.	Ist die Impfstelle gerötet oder geschwollen?	☐	☐	______________________
3.	Haben Sie Fieber, Schüttelfrost oder Gliederschmerzen?	☐	☐	______________________
4.	Haben Sie Kopfschmerzen, Müdigkeit oder Übelkeit?	☐	☐	______________________
5.	Haben Sie andere Beschwerden?	☐	☐	______________________

3. Tag 2 nach der Impfung (d2)

		Ja	Nein	Weitere Angaben
1.	Haben Sie Schmerzen an der Impfstelle?	☐	☐	______________________
2.	Ist die Impfstelle gerötet oder geschwollen?	☐	☐	______________________
3.	Haben Sie Fieber, Schüttelfrost oder Gliederschmerzen?	☐	☐	______________________
4.	Haben Sie Kopfschmerzen, Müdigkeit oder Übelkeit?	☐	☐	______________________

		Ja	Nein	Weitere Angaben
5.	Haben Sie andere Beschwerden?	☐	☐	______

4. Tag 3 nach der Impfung (d3)

		Ja	Nein	Weitere Angaben
1.	Haben Sie Schmerzen an der Impfstelle?	☐	☐	______
2.	Ist die Impfstelle gerötet oder geschwollen?	☐	☐	______
3.	Haben Sie Fieber, Schüttelfrost oder Gliederschmerzen?	☐	☐	______
4.	Haben Sie Kopfschmerzen, Müdigkeit oder Übelkeit?	☐	☐	______
5.	Haben Sie andere Beschwerden?	☐	☐	______

5. Tag 4 nach der Impfung (d4)

		Ja	Nein	Weitere Angaben
1.	Haben Sie Schmerzen an der Impfstelle?	☐	☐	______
2.	Ist die Impfstelle gerötet oder geschwollen?	☐	☐	______
3.	Haben Sie Fieber, Schüttelfrost oder Gliederschmerzen?	☐	☐	______
4.	Haben Sie Kopfschmerzen, Müdigkeit oder Übelkeit?	☐	☐	______
5.	Haben Sie andere Beschwerden?	☐	☐	______

6. Tag 5 nach der Impfung (d5)

		Ja	Nein	Weitere Angaben
1.	Haben Sie Schmerzen an der Impfstelle?	☐	☐	______
2.	Ist die Impfstelle gerötet oder geschwollen?	☐	☐	______

3.	Haben Sie Fieber, Schüttelfrost oder Gliederschmerzen?	☐	☐	______________
4.	Haben Sie Kopfschmerzen, Müdigkeit oder Übelkeit?	☐	☐	______________
5.	Haben Sie andere Beschwerden?	☐	☐	______________

7. Tag 6 nach der Impfung (d6)

		Ja	Nein	Weitere Angaben
1.	Haben Sie Schmerzen an der Impfstelle?	☐	☐	______________
2.	Ist die Impfstelle gerötet oder geschwollen?	☐	☐	______________
3.	Haben Sie Fieber, Schüttelfrost oder Gliederschmerzen?	☐	☐	______________
4.	Haben Sie Kopfschmerzen, Müdigkeit oder Übelkeit?	☐	☐	______________
5.	Haben Sie andere Beschwerden?	☐	☐	______________

8. Tag 7 nach der Impfung (d7)

		Ja	Nein	Weitere Angaben
1.	Haben Sie Schmerzen an der Impfstelle?	☐	☐	______________
2.	Ist die Impfstelle gerötet oder geschwollen?	☐	☐	______________
3.	Haben Sie Fieber, Schüttelfrost oder Gliederschmerzen?	☐	☐	______________
4.	Haben Sie Kopfschmerzen, Müdigkeit oder Übelkeit?	☐	☐	______________
5.	Haben Sie andere Beschwerden?	☐	☐	______________

9. Tag 8 nach der Impfung (d8)

		Ja	Nein	Weitere Angaben

1.	Haben Sie Schmerzen an der Impfstelle?	☐	☐	______
2.	Ist die Impfstelle gerötet oder geschwollen?	☐	☐	______
3.	Haben Sie Fieber, Schüttelfrost oder Gliederschmerzen?	☐	☐	______
4.	Haben Sie Kopfschmerzen, Müdigkeit oder Übelkeit?	☐	☐	______
5.	Haben Sie andere Beschwerden?	☐	☐	______

10. Tag 9 nach der Impfung (d9)

		Ja	Nein	Weitere Angaben
1.	Haben Sie Schmerzen an der Impfstelle?	☐	☐	______
2.	Ist die Impfstelle gerötet oder geschwollen?	☐	☐	______
3.	Haben Sie Fieber, Schüttelfrost oder Gliederschmerzen?	☐	☐	______
4.	Haben Sie Kopfschmerzen, Müdigkeit oder Übelkeit?	☐	☐	______
5.	Haben Sie andere Beschwerden?	☐	☐	______

11. Tag 10 nach der Impfung (d10)

		Ja	Nein	Weitere Angaben
1.	Haben Sie Schmerzen an der Impfstelle?	☐	☐	______
2.	Ist die Impfstelle gerötet oder geschwollen?	☐	☐	______
3.	Haben Sie Fieber, Schüttelfrost oder Gliederschmerzen?	☐	☐	______
4.	Haben Sie Kopfschmerzen, Müdigkeit oder Übelkeit?	☐	☐	______

		Ja	Nein	Weitere Angaben
5.	Haben Sie andere Beschwerden?	☐	☐	______

12. Tag 11 nach der Impfung (d11)

		Ja	Nein	Weitere Angaben
1.	Haben Sie Schmerzen an der Impfstelle?	☐	☐	______
2.	Ist die Impfstelle gerötet oder geschwollen?	☐	☐	______
3.	Haben Sie Fieber, Schüttelfrost oder Gliederschmerzen?	☐	☐	______
4.	Haben Sie Kopfschmerzen, Müdigkeit oder Übelkeit?	☐	☐	______
5.	Haben Sie andere Beschwerden?	☐	☐	______

13. Tag 12 nach der Impfung (d12)

		Ja	Nein	Weitere Angaben
1.	Haben Sie Schmerzen an der Impfstelle?	☐	☐	______
2.	Ist die Impfstelle gerötet oder geschwollen?	☐	☐	______
3.	Haben Sie Fieber, Schüttelfrost oder Gliederschmerzen?	☐	☐	______
4.	Haben Sie Kopfschmerzen, Müdigkeit oder Übelkeit?	☐	☐	______
5.	Haben Sie andere Beschwerden?	☐	☐	______

14. Tag 13 nach der Impfung (d13)

		Ja	Nein	Weitere Angaben
1.	Haben Sie Schmerzen an der Impfstelle?	☐	☐	______
2.	Ist die Impfstelle gerötet oder geschwollen?	☐	☐	______

3.	Haben Sie Fieber, Schüttelfrost oder Gliederschmerzen?	☐	☐	______
4.	Haben Sie Kopfschmerzen, Müdigkeit oder Übelkeit?	☐	☐	______
5.	Haben Sie andere Beschwerden?	☐	☐	______

15. Tag 14 nach der Impfung (d14)

		Ja	Nein	Weitere Angaben
1.	Haben Sie Schmerzen an der Impfstelle?	☐	☐	______
2.	Ist die Impfstelle gerötet oder geschwollen?	☐	☐	______
3.	Haben Sie Fieber, Schüttelfrost oder Gliederschmerzen?	☐	☐	______
4.	Haben Sie Kopfschmerzen, Müdigkeit oder Übelkeit?	☐	☐	______
5.	Haben Sie andere Beschwerden?	☐	☐	______

16. Tag 15 nach der Impfung (d15)

		Ja	Nein	Weitere Angaben
1.	Haben Sie Schmerzen an der Impfstelle?	☐	☐	______
2.	Ist die Impfstelle gerötet oder geschwollen?	☐	☐	______
3.	Haben Sie Fieber, Schüttelfrost oder Gliederschmerzen?	☐	☐	______
4.	Haben Sie Kopfschmerzen, Müdigkeit oder Übelkeit?	☐	☐	______
5.	Haben Sie andere Beschwerden?	☐	☐	______

17. Tag 16 nach der Impfung (d16)

		Ja	Nein	Weitere Angaben

1.	Haben Sie Schmerzen an der Impfstelle?	☐	☐	____________
2.	Ist die Impfstelle gerötet oder geschwollen?	☐	☐	____________
3.	Haben Sie Fieber, Schüttelfrost oder Gliederschmerzen?	☐	☐	____________
4.	Haben Sie Kopfschmerzen, Müdigkeit oder Übelkeit?	☐	☐	____________
5.	Haben Sie andere Beschwerden?	☐	☐	____________

18. Tag 17 nach der Impfung (d17)

		Ja	Nein	Weitere Angaben
1.	Haben Sie Schmerzen an der Impfstelle?	☐	☐	____________
2.	Ist die Impfstelle gerötet oder geschwollen?	☐	☐	____________
3.	Haben Sie Fieber, Schüttelfrost oder Gliederschmerzen?	☐	☐	____________
4.	Haben Sie Kopfschmerzen, Müdigkeit oder Übelkeit?	☐	☐	____________
5.	Haben Sie andere Beschwerden?	☐	☐	____________

19. Tag 18 nach der Impfung (d18)

		Ja	Nein	Weitere Angaben
1.	Haben Sie Schmerzen an der Impfstelle?	☐	☐	____________
2.	Ist die Impfstelle gerötet oder geschwollen?	☐	☐	____________
3.	Haben Sie Fieber, Schüttelfrost oder Gliederschmerzen?	☐	☐	____________
4.	Haben Sie Kopfschmerzen, Müdigkeit oder Übelkeit?	☐	☐	____________

		Ja	Nein	Weitere Angaben
5.	Haben Sie andere Beschwerden?	☐	☐	______

20. Tag 19 nach der Impfung (d19)

		Ja	Nein	Weitere Angaben
1.	Haben Sie Schmerzen an der Impfstelle?	☐	☐	______
2.	Ist die Impfstelle gerötet oder geschwollen?	☐	☐	______
3.	Haben Sie Fieber, Schüttelfrost oder Gliederschmerzen?	☐	☐	______
4.	Haben Sie Kopfschmerzen, Müdigkeit oder Übelkeit?	☐	☐	______
5.	Haben Sie andere Beschwerden?	☐	☐	______

21. Tag 20 nach der Impfung (d20)

		Ja	Nein	Weitere Angaben
1.	Haben Sie Schmerzen an der Impfstelle?	☐	☐	______
2.	Ist die Impfstelle gerötet oder geschwollen?	☐	☐	______
3.	Haben Sie Fieber, Schüttelfrost oder Gliederschmerzen?	☐	☐	______
4.	Haben Sie Kopfschmerzen, Müdigkeit oder Übelkeit?	☐	☐	______
5.	Haben Sie andere Beschwerden?	☐	☐	______

22. Tag 21 nach der Impfung (d21)

		Ja	Nein	Weitere Angaben
1.	Haben Sie Schmerzen an der Impfstelle?	☐	☐	______
2.	Ist die Impfstelle gerötet oder geschwollen?	☐	☐	______

3.	Haben Sie Fieber, Schüttelfrost oder Gliederschmerzen?	☐	☐	______
4.	Haben Sie Kopfschmerzen, Müdigkeit oder Übelkeit?	☐	☐	______
5.	Haben Sie andere Beschwerden?	☐	☐	______

23. Tag 22 nach der Impfung (d22)

		Ja	Nein	Weitere Angaben
1.	Haben Sie Schmerzen an der Impfstelle?	☐	☐	______
2.	Ist die Impfstelle gerötet oder geschwollen?	☐	☐	______
3.	Haben Sie Fieber, Schüttelfrost oder Gliederschmerzen?	☐	☐	______
4.	Haben Sie Kopfschmerzen, Müdigkeit oder Übelkeit?	☐	☐	______
5.	Haben Sie andere Beschwerden?	☐	☐	______

24. Tag 23 nach der Impfung (d23)

		Ja	Nein	Weitere Angaben
1.	Haben Sie Schmerzen an der Impfstelle?	☐	☐	______
2.	Ist die Impfstelle gerötet oder geschwollen?	☐	☐	______
3.	Haben Sie Fieber, Schüttelfrost oder Gliederschmerzen?	☐	☐	______
4.	Haben Sie Kopfschmerzen, Müdigkeit oder Übelkeit?	☐	☐	______
5.	Haben Sie andere Beschwerden?	☐	☐	______

25. Tag 24 nach der Impfung (d24)

		Ja	Nein	Weitere Angaben

		Ja	Nein	Weitere Angaben
1.	Haben Sie Schmerzen an der Impfstelle?	☐	☐	______
2.	Ist die Impfstelle gerötet oder geschwollen?	☐	☐	______
3.	Haben Sie Fieber, Schüttelfrost oder Gliederschmerzen?	☐	☐	______
4.	Haben Sie Kopfschmerzen, Müdigkeit oder Übelkeit?	☐	☐	______
5.	Haben Sie andere Beschwerden?	☐	☐	______

26. Tag 25 nach der Impfung (d25)

		Ja	Nein	Weitere Angaben
1.	Haben Sie Schmerzen an der Impfstelle?	☐	☐	______
2.	Ist die Impfstelle gerötet oder geschwollen?	☐	☐	______
3.	Haben Sie Fieber, Schüttelfrost oder Gliederschmerzen?	☐	☐	______
4.	Haben Sie Kopfschmerzen, Müdigkeit oder Übelkeit?	☐	☐	______
5.	Haben Sie andere Beschwerden?	☐	☐	______

27. Tag 26 nach der Impfung (d26)

		Ja	Nein	Weitere Angaben
1.	Haben Sie Schmerzen an der Impfstelle?	☐	☐	______
2.	Ist die Impfstelle gerötet oder geschwollen?	☐	☐	______
3.	Haben Sie Fieber, Schüttelfrost oder Gliederschmerzen?	☐	☐	______
4.	Haben Sie Kopfschmerzen, Müdigkeit oder Übelkeit?	☐	☐	______

5.	Haben Sie andere Beschwerden?	☐	☐	______________________

28. Tag 27 nach der Impfung (d27)

		Ja	Nein	Weitere Angaben
1.	Haben Sie Schmerzen an der Impfstelle?	☐	☐	______________________
2.	Ist die Impfstelle gerötet oder geschwollen?	☐	☐	______________________
3.	Haben Sie Fieber, Schüttelfrost oder Gliederschmerzen?	☐	☐	______________________
4.	Haben Sie Kopfschmerzen, Müdigkeit oder Übelkeit?	☐	☐	______________________
5.	Haben Sie andere Beschwerden?	☐	☐	______________________

14.4. Volunteer card

[redacted]

Studienkarte

Patientenname ____

Nimmt an der **P-pVac-SARS-CoV-2 Studie** zur Evaluation eines SARS-CoV-2 Impfstoff teil und wurde einmalig mit dem Impfstoff behandelt.

Bitte kontaktieren Sie im Notfall:

____

____

Bitte tragen Sie diese Notfallkarte immer bei sich

14.5. Intensity of solicited and unsolicited local and systemic adverse events

Local solicited AEs	CTCAE Term	Grade 0<br>Normal	Grade 1<br>Mild	Grade 2<br>Moderate	Grade 3<br>Severe	Grade 4<br>Potentially life-threatening
**Erythema**	Injection site reaction	< 25 mm	25-50mm<br>Tenderness with or without associated symptoms (e.g., warmth, erythema, itching)	51-100mm<br>Pain; lipodystrophy; edema; phlebitis	> 100mm<br>Ulceration or necrosis; severe tissue damage; operative intervention indicated	Life-threatening consequences; urgent intervention indicated
**Swelling**		< 25 mm	25-50 mm and does not interfere with activity	> 50 mm or interferes with activity	Prevents daily activity	Necrosis
**Pain**	Injection site reaction	None	Tenderness with or without associated symptoms (e.g., warmth, erythema, itching)<br><br>Does not interfere with activity	Pain; lipodystrophy; edema; phlebitis<br><br>Interferes with activity	Ulceration or necrosis; severe tissue damage; operative intervention indicated<br><br>Prevents daily activity	Life-threatening consequences; urgent intervention indicated<br>Emergency room visit or hospitalization

Systemic solicited AEs	CTCAE Term	Grade 0	Grade 1	Grade 2	Grade 3	Grade 4
**Fever**		None	38.0° - 39.0°C	≥ 39.0° - 40.0°C	≥ 40.0°C for ≤ 24 hours	≥ 40.0°C for ≥ 24 hours
**Chills**		None	Mild sensation of cold; shivering; chattering of teeth	Moderate tremor of the entire body; narcotics indicated	Severe or prolonged, not responsive to narcotics	-
**Myalgia** (described to the subject as generalized muscle ches)		None	Mild pain	Moderate pain; limiting instrumental ADL	Severe pain; limiting self care ADL	-
**Arthralgie** (described to the subject as generalized joint aches)			Mild pain	Moderate pain; limiting instrumental ADL	Severe pain; limiting self care ADL	-
**Fatigue**			Fatigue relieved by rest	Fatigue not relieved by rest; limiting instrumental ADL	Fatigue not relieved by rest, limiting self care ADL	-
**Headache**		None	Mild pain	Moderate pain; limiting instrumental ADL	Severe pain; limiting self care ADL	-
**Gastrointestinal symptoms** (nausea, vomiting, abdominal pain, and/or diarrhea)	nausea	None	Loss of appetite without alteration in eating habits	Oral intake decreased without significant weight loss, dehydration or malnutrition	Inadequate oral caloric or fluid intake; tube feeding, TPN, or hospitalization indicated	-
	vomiting	None	Intervention not indicated	Outpatient IV hydration; medical intervention indicated	Tube feeding, TPN, or hospitalization indicated	Life-threatening consequences
	abdominal pain	None	Mild pain	Moderate pain; limiting instrumental ADL	Severe pain; limiting self care ADL	-
	diarrhea	None	Increase of <4 stools per day over baseline; mild increase in ostomy output compared to baseline	Increase of 4 - 6 stools per day over baseline; moderate increase in ostomy output compared to baseline; limiting instrumental ADL	Increase of ≥7 stools per day over baseline; hospitalization indicated; severe increase in ostomy output compared to baseline; limiting self care ADL	Life-threatening consequences; urgent intervention indicated

14.6. List of specific immune mediated diseases (pIMDs)

Neuroinflammatory disorders	Musculoskeletal disorders	Skin disorders	Liver disorder	Gastrointestinal disorders	Metabolic & endocrine disorders	Vasculitides	Others
Cranial nerve inflammatory disorders, including paralyses/paresis (e.g., Bell's palsy)	Systemic lupus erythematosus	Psoriasis	Autoimmune hepatitis	Crohn's disease	Autoimmune thyroiditis (including Hashimoto thyroiditis)	Large vessels vasculitis including: giant cell arteritis such as Takayasu's arteritis & temporal arteritis	Autoimmune haemolytic anaemia
Acute disseminated encephalomyelitis including site- specific variants: encephalitis, encephalomyelitis, myelitis, myeloradiculoneuritis, cerebellitis	Systemic sclerosis (with limited or diffuse cutaneous involvement)	Vitiligo	Primary biliary cirrhosis	Ulcerative colitis	Grave's or Basedow's disease		Autoimmune thrombocytopenia
Multiple sclerosis	Dermatomyositis	Erythema nodosum	Primary sclerosing cholangitis	Ulcerative proctitis	Diabetes mellitus type I		Antiphospholipid syndrome
Transverse myelitis	Polymyositis		Autoimmune cholangitis.	Celiac disease	Addison's disease		Pernicious anaemia
Optic neuritis	Anti-synthetase syndrome	Cutaneous lupus erythematosus					Raynaud's phenomenon
Narcolepsy	Rheumatoid arthritis	Alopecia areata					Uveitis
	Juvenile chronic arthritis (including Still's disease)	Lichen planus					Autoimmune myocarditis/cardiomyopathy
	Polymyalgia rheumatica	Sweet's syndrome					Sarcoidosis
	Psoriatic arthropathy	Morphoea					Stevens-Johnson syndrome
	Relapsing polychondritis						Sjögren's syndrome
Myasthenia gravis (including Lambert-Eaton myasthenic syndrome)	Mixed connective tissue disorder						Idiopathic pulmonary fibrosis
							Goodpasture syndrome
Immune mediated peripheral neuropathies and plexopathies, (including Guillain-Barré syndrome, Miller Fisher syndrome and other variants, chronic inflammatory demyelinating polyneuropathy, multifocal motor neuropathy and polyneuropathies associated with monoclonal gammopathy)	Spondyloarthritis, including ankylosing spondylitis, reactive arthritis (Reiter's Syndrome) and undifferentiated spondyloarthritis	Autoimmune bullous skin diseases (including pemphigus, pemphigoid & dermatitis herpetiformis)				Medium sized and/or small vessels vasculitis including: polyarteritis nodosa, Kawasaki's disease, microscopic polyangiitis, Wegener's granulomatosis, Churg–Strauss syndrome (allergic granulomatous angiitis), Buerger's disease (thromboangiitis obliterans), necrotising vasculitis & anti-neutrophil cytoplasmic antibody (ANCA) positive vasculitis (type unspecified), Henoch-Schonlein purpura, Behcet's syndrome, leukocytoclastic vasculitis	Autoimmune glomerulonephritis (including IgA nephropathy, glomerulonephritis rapidly progressive, membranous glomerulonephritis, membranoproliferative glomerulonephritis, & mesangioproliferative glomerulonephritis)

Adapted from Tavares Da Silva, F et al., Optimal approaches to data collection and analysis of potential immune mediated disorders in clinical trials of new vaccines, Vaccine, 2013 [90]

[redacted]

[redacted]

[redacted]

[redacted]

[redacted]

[redacted]

[redacted]

[redacted]

[redacted]

[redacted]

[redacted]

-

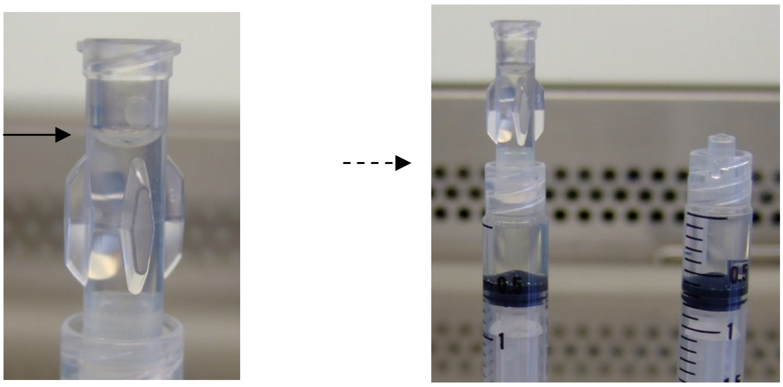

[redacted]

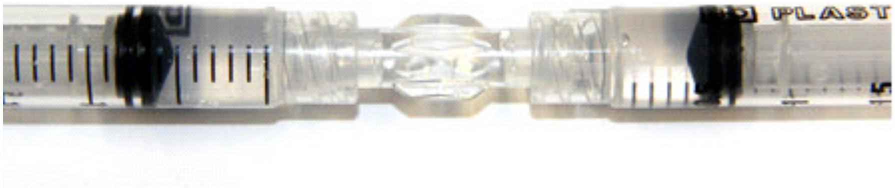

[redacted]

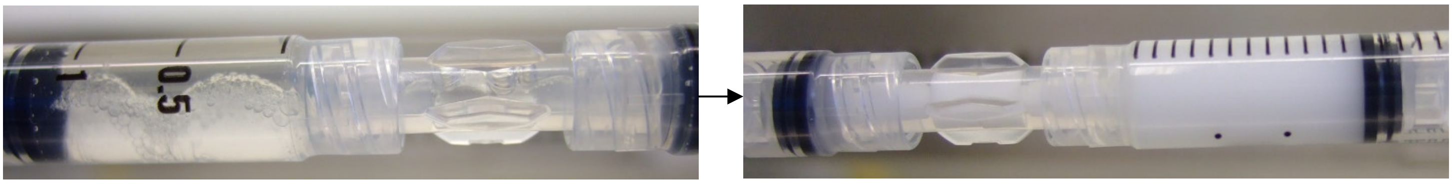

[redacted]

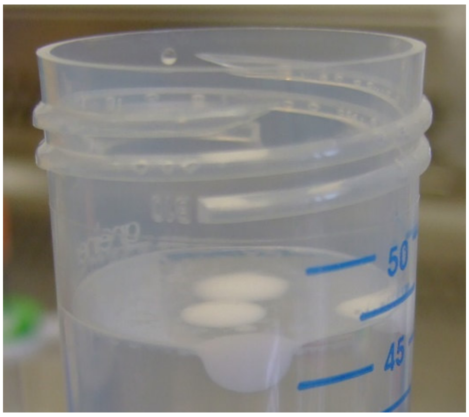

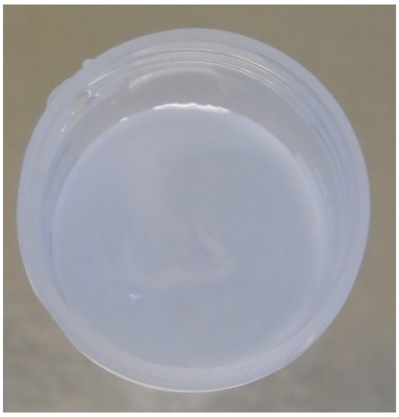

[redacted] [redacted]

[redacted]

[redacted]
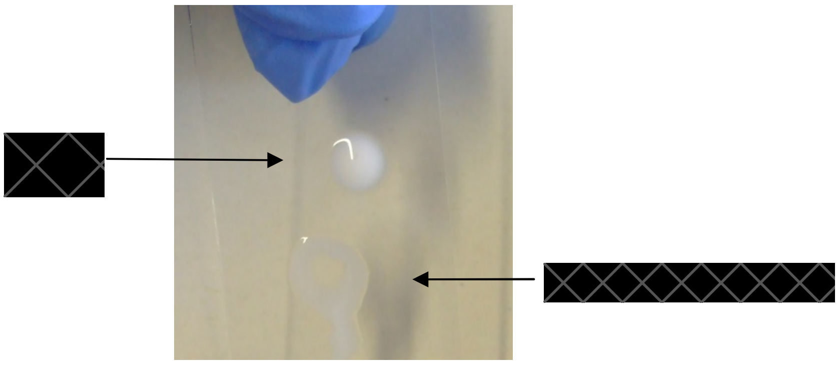
[redacted]

[redacted]

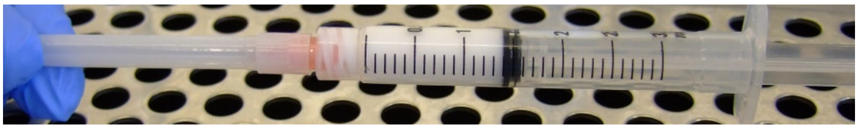

[redacted]

[redacted]

[redacted]

P-pVAC-SARS-CoV-2: Phase I single-center safety and immunogenicity trial of multi-peptide vaccination to prevent COVID-19 infection in adults

Statistical Analysis Plan (SAP)

Version: Final

[redacted]

Confidentiality statement

The contents of this document are the property of the applicants at the Eberhard-Karls University of Tübingen Germany. The contents may not be used or disclosed in any way without the express permission of the applicants.

Note

This statistical analysis plan was established according to the SOP BI03 (17.05.2019) of the ZKS Tübingen. The analysis tables and listings will be independently validated by a second statistician according the SOP BI06 of the ZKS Tübingen.

Content

Abbreviations 4
1 Introduction 6
1.1 Background 6
1.2 Purpose of the Trial 6
2 Study Objectives 6
2.1 Primary Objective 6
2.2 Secondary Objectives 6
2.3 Explorative Objectives 6
3 General Study Design and Plan 7
3.1 Overall Trial Design 7
3.2 Inclusion-Exclusion Criteria 8
3.2.1 Inclusion Criteria 8
3.2.2 Exclusion Criteria 8
3.3 Method of Treatment Assignment 10
3.4 Study Drug Administration 10
3.5 Study Procedures 10
3.6 Endpoints 13
3.6.1 Primary Endpoint 13
3.6.2 Secondary Endpoints 13
3.6.3 Exploratory Endpoints 13
4 Sample Size and Power Consideration 14
5 Data Collection and Storage 14
6 General Considerations 14
6.1 Timing of Analyses 14
6.2 Analysis Population 14
6.3 Missing Data and Drop Outs 15
6.4 Results of the Interim Analysis and the Safety Interim Analysis 15
7 Summary of Study Data 17
7.1 Subject Disposition 17
7.2 Derived Variables 17
7.3 Protocol Violations 17
7.4 Demographic and Baseline Variables 18
7.5 Medical History and Medications 18
7.6 Treatment Compliance 18
8 Efficacy Analyses 19
8.1 Primary Endpoint 19
8.3 Secondary Endpoints 19
8.4 Exploratory Endpoints 19
8.5 Subgroup analysis 20
10 Safety Analyses 20
11 Reporting Conventions 20
12 Technical Details 20
13 Sumamry of Changes to the Protocol 20
14 Data Source 21
15 Planned Tables, Figures and Listing 22

Abbreviations

ADR	Adverse Drug Reaction
ADE	Antibody-dependent Enhancement
AE	Adverse Event
AESI	Adverse Event of Special Interest
ALT	Alanine Transaminase (SGPT)
AP	Alkaline Phosphatase
aPTT	Partial Thromboplastin Time
AST	Aspartate Transaminase (SGOT)
BMI	Body Mass Index
CCR	Cellular Conversion Rate
CNS	Central Nervous System
COVID-19	Coronavirus Disease 2019
COV	Coronavirus
CMV	Cytomegalovirus
CNS	Central nervous system
CoVac-1	SARS-CoV-2-derived multi-peptide vaccine
COVID-19	Coronavirus Disease 2019
CRF	Case Report Form
CRP	C-reactive Protein
CTC(AE)	Common Toxicity Criteria (for Adverse Events)
DBL	Data Base Look
DSMB	Data and Safety Monitoring Board
EC	Ethic Committee
ECOG	Eastern Cooperative Oncology Group
eCRF	electronic Case Report Form
EOS	End of Study
EOSf	End of Safety follow-up
FCBP	Female of Child Bearing Potential
γ-GT	Gamma Glutamyltransferase
GFR	Glomerular Filtration Rate
HBV	Hepatitis B Virus
HCV	Hepatitis C Virus
HIV	Human Immunodeficiency Virus
HLA	Human Leukocyte Antigen System
IFN	Interferon
Ig	Immunoglobulin
IL	Interleukin
LDH	Lactate Dehydrogenase
NYHA	New York Heart Association
pAVK	Peripheral Artery Disease
PCR	Polymerase Chain Reaction
PEI	Paul-Ehrlich-Institut
SAE	Serious Adverse Event
SARS-CoV-2	Severe Acute Respiratory Syndrome – Coronavirus 2
SAP	Statistical Analysis Plan

TLR	Toll-like Receptor
TNF	Tumour Necrosis Factor
ULN	Upper Limit of Normal

1 Introduction

This SAP is based on the study protocol Version 1.4 as of 8 March 2021 (EudraCT Nr. 2020-002502-75). Text citation directly from the study protocol is given in italic letters.

1.1 Background

The novel coronavirus SARS-CoV-2 causes the COVID-19 disease, … and has spread to a worldwide pandemic. T cells play the central role in SARS-CoV-2 infection and COVID-19 disease. The main goal of this study is to develop a vaccine candidate that induces superior SARS-VoV-2 T-cell immunity to better combat COVID-19.

The aim of this study is to investigate the safety and immunogenicity of a peptide vaccine consisting of SARS-CoV-2 specific HLA class II peptides in volunteers without prior or current SARD-CoV-2 infection.

The trial has been conceptualized to prove safety and immunogenicity of a peptide vaccine against SARS-CoV-2. The focus in the study population is set to older participants. This is of special interest as these people are considered to be at high risk for severe disease. Vaccination will be conducted in **two** *different healthy volunteer cohorts (Part I* **and II**[1]) with healthy adults aged 18 – 55 years in Part I, and adults aged 56 – 80 years in Part II.

1.2 Purpose of the Trial

This trial will be performed *to evaluate the safety and immunogenicity of a single use of a SARS-CoV-2-derived multi-peptide vaccine* (CoVac-1) *in combination with the TLR1/2 ligand XS15 in adults.*

2 Study Objectives

2.1 Primary Objective

The primary objective of this trial is to evaluate the safety and tolerability of the CoVac-1 vaccine, a single dose SARS-CoV-2 specific multi-peptide vaccine combined with the TLR1/2 ligand XS15 emulsified in Montanide ISA 51 VG in adults.

2.2 Secondary Objectives

Secondary objectives of this trial are to evaluate the efficacy of the CoVac-1 vaccine in terms of induction of SARS-CoV-2 specific T-cells.

2.3 Explorative Objectives

Explorative objectives are the duration and characteristics of T-cell responses and the analysis of induction of antibody responses to single SARS-CoV-2 t-cell epitopes included in the CoVac-1 vaccine.

[1] Original text in the protocol: "*Vaccination will be conducted in three different healthy volunteer cohorts (Part I - III*), *each followed by an interim safety analysis before proceeding: • Part I: Healthy adults aged 18 – 55 years • Part II: Adults aged 56 – 80… .* This text was modified in the SAP by the authors accordingly (changes in **bold**).

3 General Study Design and Plan

3.1 Overall Trial Design

This is an interventional, open-label, phase I trial evaluating the CoVac-1 vaccine, a single dose SARS-CoV-2 specific multi-peptide vaccine combined with the TLR1/2 ligand XS15 emulsified in Montanide ISA 51 VG in adults. The study is divided into two parts, which will recruit consecutively. Prior to initiation of the next part, the previous part must have completed recruiting, and day 28 of the last patient enrolled must have passed. After interim safety analysis and approval from the authorities, the next study part starts recruiting (Figure 1).

The first volunteer included in the trial will be hospitalized after vaccination and closely monitored. This patient is observed until day 28 and possibly arising safety issues are reported to and decided on by the Sponsor. Thereafter, no more than one subject per day will be treated/vaccinated. 28 days following vaccination of the 12th volunteer, there will be an interim analysis of safety and a safety review by the data safety monitoring board (DSMB) as well as a substantial amendment to the regulatory authorities (PEI and EC) before proceeding to Part II. Part II must not start recruiting prior to approval by authorities. Volunteers of part II are treated simultaneously.

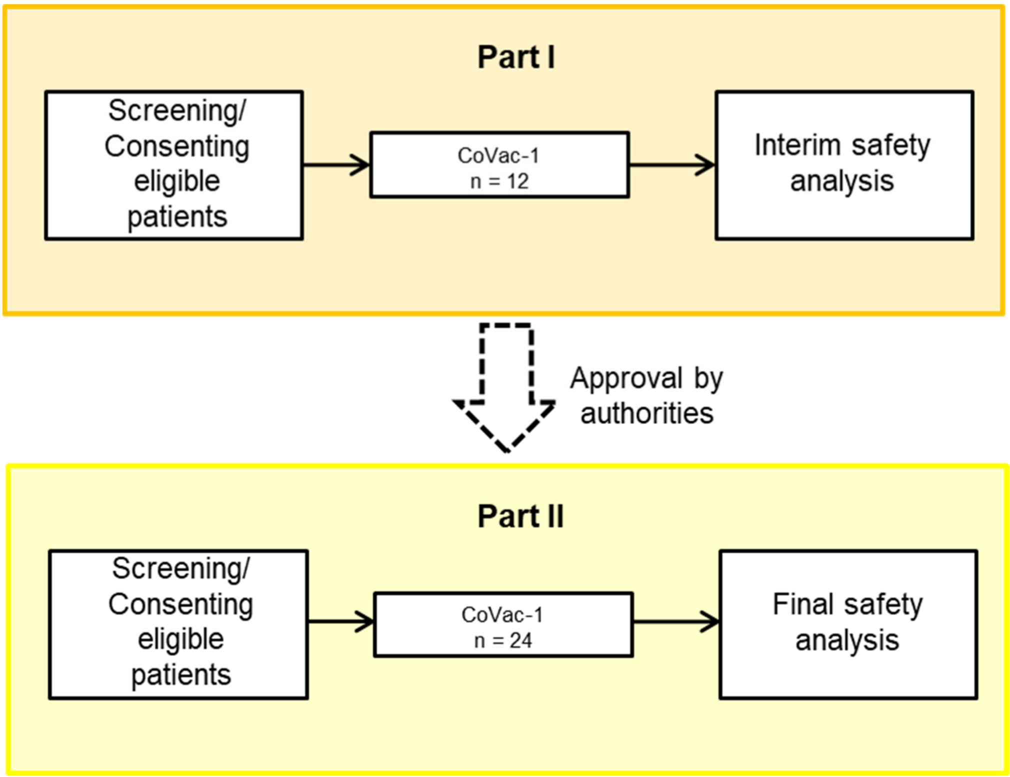

Figure 1 ***Overall Study design***

3.2 Inclusion-Exclusion Criteria

3.2.1 Inclusion Criteria

Subjects meeting all of the following criteria will be considered for admission to the trial:

1. *Adult male or non-pregnant, non-lactating female.*
    1. *Part I: Age 18 - 55 at the time of screening*
    2. *Part II: Age 56 - 80 years at the time of screening*
2. *Pre-existing medical condition*
    1. *Part I: Free of clinically significant health problems, as determined by pertinent medical history and clinical examination at study screening*
    2. *Part II: With or without pre-existing medical condition, not requiring change in therapy or hospitalization before enrollment*
3. *Ability to understand and voluntary sign the informed consent form.*
4. *Ability to adhere to the study visit schedule and other protocol requirements.*
5. *FCBP and male volunteers with partners of childbearing potential, who are sexually active must agree to the use of two effective forms (at least one highly effective method) of contraception. This should be started from the signing of the informed consent and continue until three months after vaccination.*
6. *Postmenopausal or evidence of non-childbearing status. For women of childbearing potential: negative urine or serum pregnancy test within 7 days prior to study treatment. Postmenopausal or evidence of non-childbearing status is defined as:*
    1. *Amenorrhea for 1 year or more following cessation of exogenous hormonal treatments.*
    2. *Luteinizing hormone (LH) and Follicle stimulating hormone (FSH) levels in the post-menopausal range for women under 50.*
7. *Be willing to minimize blood and body fluid exposure of others for 7 days after vaccination*
    1. *Use of effective barrier prophylaxis, such as latex condoms, during sexual intercourse.*
    2. *Avoiding the sharing of needles, razors, or toothbrushes.*
    3. *Avoiding open-mouth kissing.*
    4. *Refrain from blood donation during the course of the study.*

3.2.2 Exclusion Criteria

Subjects presenting with any of the following criteria will not be included in the trial:

1. *Pregnant or lactating females.*
2. *Participation in any clinical study with intake of any investigational drug interfering with the study primary endpoint.*

3. *Any concomitant disease affecting the effect of the therapeutic vaccine or interfering with the study primary endpoint.*
4. *Any immunosuppressive treatment except low dose corticosteroids (≤ 10 mg prednisolone/day).*
5. *Prior or current infection with SARS-CoV-2 tested serologically or by throat/nose swab (PCR).*
6. *History of Guillain-Barré Syndrome.*
7. *Positive serological HIV, hepatitis B or C test. In case of positive HBsAg, volunteer must provide prove of hepatitis B vaccination, otherwise volunteer must be excluded.*
8. *History of relevant CNS pathology or current relevant CNS (e.g. seizure, paresis, aphasia, cerebrovascular ischemia/haemorrhage, severe brain injuries, dementia, Parkinson's disease, cerebellar disease organic brain syndrome, psychosis, coordination or movement disorder, excluding febrile seizures as child).*
9. *Baseline laboratory with lymphocytes count ≤ 1000/µl.*
10. *Only Part I: acute or chronic, clinically significant psychiatric, hematologic, pulmonary, cardiovascular, or hepatic or renal functional abnormality as determined by the Investigator based on medical history, physical exam, and/or laboratory screening test.*
11. *All parts of the clinical trial*
    - *Diabetes mellitus type II requiring drug treatment*
    - *Chronic lung disease requiring drug treatment*
    - *Any chronic liver disease or unknown abnormalities defined as:*
        - *ALT and AST ≤ 2.5 x ULN*
        - *γ-GT ≤ 2.5 x ULN*
    - *Chronic renal failure defined as GFR < 60 ml/min/1.73 m^2*
    - *Serious pre-existing cardiovascular disease such as NYHA ≥ I, coronary heart disease requiring coronary surgery or known pAVK ≥ grade 2.*
    - *Sickle cell anaemia*
    - *Obesity (as defined by age adjusted body mass index).*
12. *Hospitalization at study inclusion.*
13. *Administration of immunoglobulins and/or blood products within 120 days preceding study entry or planned administration during the study period.*
14. *History of blood donation within 30 days of enrolment or planned donations within the study period.*
15. *Known hypersensitivity to any of the components included in the CoVac-1 vaccine.*
16. *Pre-existing auto-immune disease except for Hashimoto thyroiditis and mild (not requiring immunosuppressive treatment) psoriasis.*

3.3 Method of Treatment Assignment

After screening and enrolment, volunteers will be assigned to treatment with CoVac-1.

3.4 Study Drug Administration

The CoVac-1 vaccine (500 µl) will be administered subcutaneously. A subcutaneous injection of 500 µl (approx. 250 µg per peptide, 50µg XS15) will be applied. A single vaccination per patient will be conducted.

Peptide vaccines should be injected on day 1 *into the skin at the lower part of the abdomen of the patients. The site of vaccination (right or left) will be determined by the investigator and documented.*

3.5 Study Procedures

The time points and trial procedures of this study are listed in table 1.

Table 1 Table of Events

Protocol activities and forms to be completed	***Screening***	***Vaccination phase[1]***					***Follow-up period[2]***
					Interim Safety	***EOSf***	
	≤ - 7 days	***Day 1***	***Day 7 +/- 1 days***	***Day 14 +/- 1 days***	***Day 28 +/- 2 days***	***Day 56 +/- 2 days***	***3 and 6 months after peptide vaccination***
Visit		***V1***	***V2***	***V3***	***V4***	***V5***	***V6-7***
Informed consent[3]	X						
Demographics[4]	X						
Medical history[5]	X						X
Signs/symptoms[6]		X	X	X	X	X	
Enrolment[7]	X						
	Clinical assessments						
Vital signs[8]	X	X	X	X	X		
Physical examination[9]	X	X	X	X	X		
Assessment of concomitant medications[10]	X	X	X	X	X	X	
AE assessments[11]		X	X	X	X	X	X
	Laboratory assessments						
Hematology (local lab)[12]	X	X	X	X	X	X	
Blood chemistry and coagulation (local lab)[13]	X	X	X	X	X	X	
Immunoglobulins/Immuno phenotype[14]	X						
Urine analysis (local lab)[15]	X						
HBV, HCV, HIV-1, (local lab)[16]	X						
Pregnancy test[17]	X						
SARS-CoV-2 testing	X[18]						

Continued Table 1 Table of Events

Protocol activities and forms to be completed	*Screening*	*Vaccination phase*[1]					*Follow-up period*[2]
					Interim Safety	*EOSf*	
	≤ - 7 days	*Day 1*	*Day 7 +/- 1 days*	*Day 14 +/- 1 days*	*Day 28 +/- 2 days*	*Day 56 +/- 2 days*	*3 and 6 months after peptide vaccination*
Visit		*V1*	*V2*	*V3*	*V4*	*V5*	*V6-7*
	Treatment						
Vaccine CoVac-1[19]		X					
	Efficacy assessment						
T-cell response[20]		X	X	X	X	X	X
Serological response[21]		X	X	X	X	X	X

Detailed information on schedule and activities are described in the footnotes.

1. *The peptide vaccination should be applied as early as possible after screening (max. 7 days) and approved eligibility of the volunteer. Vaccination phase will be 2 months and ends with the end of safety follow-up (EOSf).*
2. *Follow-up: After vaccination phase, volunteers will enter follow-up, which ends with the last visit 6 months after vaccination (V7, EOS).*
3. *Informed consent and volunteer registration: every volunteer must date and sign informed consent form to participate in this trial before starting any trial-related procedures.*
4. *Demographics: gender, year of birth, ethnicity*
5. *Medical history: The investigator has to collect information on the volunteers' medical history including prior illnesses, hospitalisations, and symptoms of a SARS-CoV-2 infection.*
6. *Signs/symptoms: vaccine-related and -unrelated signs and symptoms*
7. *Enrolment: volunteers are enrolled and registered through a screening procedure. Each volunteer will be registered under a specific Vol. ID on a subject log kept at the trial site.*
8. *Vital signs: At all visits: ECOG, temperature (in grade centigrade), blood pressure/pulse. At baseline additionally: height (in cm) and weight (in kg). At V4 and V5 additionally: weight (in kg). For detailed surveillance after vaccination, please refer to section* ***Fehler! Verweisquelle konnte nicht gefunden werden.*** *of the study protocol*
9. *Physical examination: inspection, abdominal, cardiac and lung auscultation, palpation of the abdomen and lymph node sites, neurological examination, inspection of vaccination site.*
10. *Concomitant medications should be reported in the respective CRF pages, including drugs used for treating AEs or, if applicable, chronic diseases.*
11. *AE assessments: events should be documented and recorded continuously. Volunteers have to be followed for AEs from application up to 56 days or until all drug-related toxicities have been resolved, whichever is later, or until the investigator assesses AEs as "chronic" or "stable". Each AE must be reported indicating the CTC (Version 5.0) grade. If an event stops and later restarts or CTC grading changes, all occurrences must be reported. A specific procedure for definition and reporting of SAEs is described in the protocol.*
12. *Hematology (local lab): hemoglobin (Hb), red blood cells (RBC), platelet count (PLT) white blood cells (WBC). Differential cell counts should be performed at baseline, at each visit during vaccination phase and thereafter at investigators discretion. Clinical status and laboratory parameters are to be followed using individual institutional guidelines and the best clinical judgment of the responsible physician, which can involve more frequent testing.*
13. *Blood chemistry and coagulation (local lab): Alkaline phosphatase (AP), total bilirubin, aspartate transaminase (AST/ SGOT), alanine transaminase (ALT/ SGPT), lactate dehydrogenase (LDH), and uric acid, C-reactive protein (CRP), sodium, potassium, calcium, blood urea nitrogen, creatinine, glucose: at baseline and during vaccination phase, thereafter at each visit using individual institutional guidelines and the best clinical judgment of the responsible physician, which can involve more frequent testing. Prothrombin time, aPTT, and fibrinogen will be measured at baseline and at investigator's discretion during treatment.*
14. *Immunoglobulin/immunophenotype: Assessment of IgA, IgG and IgM; lymphocyte subsets: T ($CD4^+$ and $CD8^+$) as well as B and NK cells.*
15. *Urine analysis (local lab): pH, glucose, proteins (qualitative, dipstick accepted): at baseline and at investigator's discretion during treatment*
16. *HBV, HCV and HIV-1: at baseline and thereafter at investigator's discretion*
17. *Pregnancy testing: For all FCBP, pregnancy testing has to be performed at the screening visit. Negative results must be available prior to vaccination.*

18. *SARS-CoV-2 testing: Volunteer must be tested for prior or current SARS-CoV-2 infection. Patients should be tested by serological test and throat/nose swab. If screening takes more than 48 hours, throat/nose swab for SARS-CoV-2 infection must be repeated. The vaccine can only be applied if a negative SARS-CoV-2 PCR test is available on the day of vaccination not older than 48 hours.If patients develop SARS-CoV-2 typical symptoms until vaccination, testing should be repeated.*
19. *Vaccine CoVac-1: Peptide vaccination should be started as soon as possible after the screening visit. Peptide vaccination will be performed once.*
20. *T-cell response: 60 ml of heparin blood for immunomonitoring and analysis of peptide specific T-cell response will be analyzed by the Walz lab, KKE Translational Immunology at the Department of Immunology, Tuebingen (central laboratory). Blood will be taken before peptide vaccination on V1, and during vaccination phase and follow-up at each visit.*
21. *Serological response: 10 ml of serum for analysis of serological response will be analysed by the Immunopathological Laboratory, University Hospital Tuebingen (central laboratory). Blood will be taken before peptide vaccination on V1, and during vaccination phase and follow-up at each visit.*

3.6 Endpoints

3.6.1 Primary Endpoint

The primary endpoint will be *the nature, frequency, and severity of AEs and/or SAEs associated with administration of CoVac-1:*

- *<u>Solicited</u>: ADRs/AE occurring from the time of each injection throughout 28 days following the procedure, facilitated by use of a volunteer diary.*
- *<u>Unsolicited</u>: AEs from the time of injection throughout 56 days following injection.*
- *Incidence of AESIs until the final study visit for each subject.*

3.6.2 Secondary Endpoints

The secondary endpoints will be *the development of a CoVac-1 specific T-cell response to at least one of the single SARS-CoV-2 T-cell epitopes included in the CoVac-1 vaccine on visit 2, 3, 4, 5 measured by IFN-γ ELISpot ex vivo and after in vitro T-cell amplification (compared to visit 1). This includes cellular conversion rate (CCR) at visit 2, 3, 4, 5 after immunization.*

3.6.3 Exploratory Endpoints

As exploratory endpoints the following parameters will be analyzed.

- *characteristic of T-cell response on visit 2, 3, 4, 5 measured by ELISpot/ICS, this includes:*
  - *Phenotyping of SARS-CoV-2 specific T-cells (CD4, CD8 etc.) by flow cytometry*
  - *Characterization of cytokine profiles of SARS-CoV-2 specific T-cells (TNF, IFN, IL-2, CD107a etc.) by intracellular cytokine staining*
  - *Recognition rate defined as percentage of peptides including a T-cell response in one individual*
  - *Intensity of T-cell response to a single SARS-CoV-2 T-cell epitope included in the CoVac-1 vaccine.*
- *Induction of long-term SARS-CoV-2 specific T-cell response 3 and 6 months after peptide vaccination.*
- *Induction of antibodies specific to the SARS-CoV-2 T-cell epitopes included in the CoVac-1 vaccine measured by ELISA. In case of unexpected detection of CoVac-1 specific antibodies the following assays will be performed:*
  - *Individual neutralization antibody titers*
  - *Seroconversion rates*
  - *Calculation of genomic mean titers (GMT) for neutralization and binding antibodies.*
- *Biomarkers and clinical characteristics influencing immunogenicity.*

4 Sample Size and Power Consideration

In this phase I study the safety/toxicity of one vaccination will be investigated. For this purpose, it will be investigated whether the incidence of severe adverse events (SAE) associated with administration of CoVac-1 exceeds a predetermined rate of 5% (= P_1 = alternative hypothesis) in the whole study population. Safety of the CoVac-1 vaccine is shown if no SAE (= P_0 = null hypothesis) occurs in the study population. An evaluable sample size of 33 achieves 81.6% power to detect a difference (P_1-P_0) of 0.0499 using a one-sided exact test based on the binomial distribution with a target significance level of 0.05. The actual significance level achieved by this test is 0.003. These results assume that the population proportion under the null hypotheses (P_0) is 0.0001. Assuming a dropout rate of 7.5% (percentage of subjects that are expected to be lost at random during the course of the study and for whom no response data concerning existence of SAE will be collected, i.e. will be treated as "missing") the total number of 36 subjects should be enrolled in the study in order to end up with 33 evaluable subjects. Sample size computed using PASS 2020 (NCSS, LLC, Kaysville, Utah, USA).

5 Data Collection and Storage

The Clinical Data Management System [secuTrial "SecuTrial"] will be used for data capture, processing and storage of study data. Data entry is performed at the investigational site by clinical staff after having received training and a user manual for the electronic CRF. The Principal investigator is responsible for ensuring that all sections of the eCRF are completed correctly and that entries can be verified against source data.

6 General Considerations

6.1 Timing of Analyses

The final analysis will be performed after the first data base look which will be performed after regularly termination of visit 5 of the last volunteer and completed data monitoring. The data base lock will be confirmed by the sponsor, and therefore later changes of data until visit 5 are not allowed.

For the follow-up period a second data base lock will be performed after regularly termination of visit 7 of the last volunteer and completed data monitoring. The data base lock will be confirmed by the sponsor.

Both data base locks will be performed after the finalization and approval of this SAP document.

6.2 Analysis Population

The analysis population consists of all included volunteers with the exception of volunteers who withdraw their informed consent for participating furthermore and for analysis of their data during the study.

6.3 Missing Data and Drop Outs

Missing values will be predicted based on plausible assumptions that account for the uncertainty due to missing data. For patients with unknown status for the primary endpoint, i.e. a volunteer without complete follow-up and without any SAE until the last known study site contact, a detailed report on the course should be presented by the investigator and discussed concerning probable unknown SAEs and the reasons for drop-out. If substantial reason will be found that the person could have experienced a SAE, this will be interpreted as failure and the recruitment should be stopped accordingly. Otherwise the safety of the person will be interpreted as success, i.e. the subject will be interpreted to have not experienced a SAE. If this decision cannot be precisely concluded, patient will be considered as drop-out. All missing data or inconsistencies will be resolved by the responsible investigator.

[redacted]

[redacted]

[redacted]

[redacted]

[redacted]

AE	[redacted]	[redacted]	[redacted]
[redacted]	[redacted]	[redacted]	[redacted]
[redacted]	[redacted]	[redacted]	[redacted]
[redacted]	[redacted]	[redacted]	[redacted]
[redacted]	[redacted]	[redacted]	[redacted]
[redacted]	[redacted]	[redacted]	[redacted]
[redacted]	[redacted]	[redacted]	[redacted]
[redacted]	[redacted]	[redacted]	[redacted]
[redacted]	[redacted]	[redacted]	[redacted]
[redacted]	[redacted]	[redacted]	[redacted]
[redacted]	[redacted]	[redacted]	[redacted]
[redacted]	[redacted]	[redacted]	[redacted]
[redacted]	[redacted]	[redacted]	[redacted]
[redacted]	[redacted]	[redacted]	[redacted]
[redacted]	[redacted]	[redacted]	[redacted]
[redacted]	[redacted]	[redacted]	[redacted]
[redacted]	[redacted]	[redacted]	[redacted]
[redacted]	[redacted]	[redacted]	[redacted]
[redacted]	[redacted]	[redacted]	[redacted]
[redacted]	[redacted]	[redacted]	[redacted]

[redacted]

[redacted]

[redacted]	[redacted]	[redacted]	[redacted]
[redacted]	[redacted]	[redacted]	[redacted]
[redacted]	[redacted]	[redacted]	[redacted]
[redacted]	[redacted]	[redacted]	[redacted]
[redacted]	[redacted]	[redacted]	[redacted]
[redacted]	[redacted]	[redacted]	[redacted]
[redacted]	[redacted]	[redacted]	[redacted]
[redacted]	[redacted]	[redacted]	[redacted]
[redacted]	[redacted]	[redacted]	[redacted]
[redacted]	[redacted]	[redacted]	[redacted]
[redacted]	[redacted]	[redacted]	[redacted]
[redacted]	[redacted]	[redacted]	[redacted]
[redacted]	[redacted]	[redacted]	[redacted]

[redacted]

[redacted]

[redacted]

[redacted]

[redacted]

[redacted]

[redacted]

[redacted]

[redacted]

7 Summary of Study Data

All continuous variables will be summarized using the following descriptive statistics: n (non-missing sample size), mean, standard deviation, median, maximum and minimum, and quartiles. The frequency and percentages (based on the non-missing sample size) of observed levels will be reported for all categorical measures. In general, all data will be listed, sorted by subject, and when appropriate by visit number within subject. All summary tables will be structured with a column for each part in the order (part I, part II, all) and will be annotated with the total population size relevant to that table/treatment, including any missing observations.

7.1 Subject Disposition

The subject disposition during the study will be shown in a CONSORT 2010 flow diagram with information to the number of volunteers enrolled (assessed for eligibility), with follow-up, and analyzed. This flow diagram will be established with 2 arms, for part I and part II separately. The number of volunteers reaching the various stages of the trial as well as the number of drop outs and for what reason will be given. Additionally, for all included volunteers the time participating in the trial will be shown. Flow charts will be established for patient numbers until V5, and for patient numbers until V7. The summary statistics will be in accordance with section 7.

7.2 Derived Variables

Within the analysis of this trial the following derived variables will be performed:

- Study duration: time from V1 to V4
- Study duration: time from V1 to V5
- Study duration with follow up: time form V1 to V7

7.3 Protocol Deviation

Protocol deviations will be categorized as major or minor prior to the database lock. Major protocol deviations are defined as follows:

- Missing visit V1, V2, V3, V4, and V5
- No vaccination or vaccination within > 7 days after screening
- Incorrect application of CoVac-1 (not abdominal)

Minor protocol deviations are defined as follows:

- Missing of V6 or V7
- Deviation from the given visit windows of ± 1 day (V2 and V3), and ± 2 day (for V4 and V5) for the scheduled visits.
- Compliance to concomitant medication
- No blood sample at a visit
- Application of another COVID-19 vaccine until V7

7.4 Demographic and Baseline Variables

The following variable groups will be considered as demographic and baseline variables:

- Demographic characteristics (age, age class for part II (56 – 64, ≥ 65), gender, ethnicity)
- Anamnestic characteristics (BMI, BMI classified (≤ 24.9, 25.0 – 29.9, ≥ 30.0), vital signs)
- Laboratory parameters (hematology, blood parameters, immunoglobulins/immuno-phenotype, urine parameters)

These variables were recorded at screening visit (see table 1). The summary statistics will be produced in accordance with section 7.

7.5 Medical History and Medications

Within the baseline visit the following variable groups were documented for medical history and treatment:

- Medical history
- Concomitant medications

These variables were recorded at screening and for concomitant medications additionally during the study at V1 – V5 (see table 1). Data will be shown within the individual data listing.

Other concomitant medication will be documented, and in case of deviation from the study protocol -

- *immunosuppressive agents apart from ≤ 10 mg prednisolone or equivalent*
- *other vaccination during the trial*
- *non-urgent medical interventions during the trial*

are prohibited - classified as major or minor protocol deviation.

Additionally *initiation of new medications, regardless of indication must be discussed with the investigator and must be noted on the participant's record*.

7.6 Treatment Compliance

Treatment compliance with respect to the study medication doesn't matter because there is only one administration (visit 1).

8 Efficacy Analyses

8.1 Primary Endpoint

The occurrence of critical events (SAE) associated with administration of CoVac-1 should be reported to the Sponsor and documented immediately in the eCRF (within 48h). The statistical center will evaluate the occurrence of critical events using automatized alerts of the e(CRF) on a daily basis and distribute this information to the Sponsor/DSMB. If one critical event will be observed, the formal statistical stopping rule of the study is reached and no further recruitment is adequate. Otherwise the safety of the procedure will be accepted, if no out of 33 volunteers will experience a critical event.

Safety of the CoVac-1 vaccine will be statistically evaluated by means of a one-sided exact test based on the binomial distribution with a target level of 0.05 to show that no SAE observed in the study population statistically confirms that the population proportion under the null hypotheses is ≤ 0.0001 (see Chapter 4).

No further statistical tests with confirmatory aim are planned.

8.2 Secondary Endpoints

<u>Safety</u>

The statistical analysis of the secondary endpoint will be done in a descriptive manner. No statistical tests with confirmatory aim are planned. The toxicity and safety will be described by absolute and relative frequencies using CTCAE V5.0-scoring.

<u>Immunological Efficacy</u>

The rate of patients with induction of peptide-specific T-cell responses within a maximum of 56 days after vaccination will be the secondary endpoint for efficacy. T-cell responses will be assessed by:

- *IFN-γ ELISPOT*
- *Intracellular cytokine staining for TNF and IFN-γ*

The rate of patients with induction of antibody responses within a maximum of 56 days after vaccination will be the secondary endpoint for efficacy. The antibody response will be assessed by ELISA.

The measurement of T-cell response will be operationalized as given in section 3.6.2. Analysis will be performed by relative and absolute frequencies of volunteers with at least one positive of the six single SARS-CoV-2 T-cell epitopes at each visit.

Cellular conversation rate (CCR) at visit 2, 3, 4 and 5 after immunization: CCR will be calculated by dividing the number of volunteers with an immune response by the number of tested volunteers at a visit.

8.3 Exploratory Endpoints

To characterize the T-cell response and induction of antibody response to single SARS-CoV-2 epitopes included in the CoVac-1 vaccine the parameters given in section 3.6.3 will be analyzed.

8.4 Subgroup analysis

According to the study protocol *exploratory subgroup analyses are planned for each part (I and II) regarding primary and secondary endpoints*.

9 Safety Analyses

According to the study protocol (Version 1.4, 08.03.2021) the following parameters will be analyzed within the safety analysis.

(Serious) Adverse Events:

- *Vital signs: pulse, blood pressure, temperature, and weight*
- *Physical examination including inspection of the vaccination side*
- *Clinical laboratory evaluations:*
  Hematology: with blood cell (WBC), hemoglobin (Hb), platelet count, absolute neutrophil count (ANC), absolute lymphocyte count (ALC)
  Chemistry: AP, total bilirubin, AST/SGOT, ALT/SGPT, LDH, and uric acid, CRP, sodium, potassium, calcium, blood urea nitrogen, creatinine, glucose
- *Concomitant medications*
- *(S)AEs by NCI CTCAE Version 5.0 and as in appendix 14.5* of the study protocol.

The summary statistics will be produced in accordance with section 7 for (S)AEs. Other data will be shown within the individual data listing.

10 Reporting Conventions

P-values ≥ 0.001 will be reported to 3 decimal places; p-values less than 0.001 will be reported as “< 0.001”. The mean, standard deviation, and any other statistics other than quantiles, will be reported to one decimal place greater than the original data. Quantiles, such as median, or minimum and maximum will use the same number of decimal places as the original data. Estimated parameters, not on the same scale as raw observations (e.g. regression coefficients) will be reported to 3 significant figures.

11 Technical Details

At the time of writing the statistical analysis plan SAS Version 9.4 is used.

For data capture secuTrial® database version 5.6.2.3 is used.

12 Summary of Changes to the Protocol

The following changes to the study protocol will be performed:

- Despite the given information that “… *vaccination will be conducted to three different healthy volunteer cohorts (Part I – III)…”* the vaccination was conducted in two different healthy volunteer cohorts (Part I and Part II) with adults aged 18 - 55 years in Part I, and adults aged 56 - 80 years in Part II (see section 1.1).

- According to section 6.1 for the final analysis of the study data two data base lock time points will be performed. First for patients with regularly termination of visit 5 of the last volunteer and completed data monitoring, and second for patients with regularly termination of visit 7 of the last volunteer and completed data monitoring.

13 Data Source

The study data were hold within a secuTrial® data base. For the final analyses of the study the following data files will be transferred 1 : 1 to the statistical analysis system SAS:

- Screening: mnpp085scr, including emnpp085othcm, emnpp085actmed
- Enrolment: mnpp085enrol
- Baseline (Vaccination): mnpp085v1b, including emnpp085actmed; mnpp085v1v, including emnpp085vsfind, emnpp085vs
- Visit 2 – 5: mnpp085v2, including emnpp085vsfind, emnpp085mednew, emnpp085medstop, emnpp085medchange
- Visit 6 – 7: mnpp085fu, including emnpp085othcm
- End of study: mnpp085es
- T-cell response: mnpp085immo
- AE: mnpp085ae, including emnpp085aec

No other data sources will be used within the final data analyses.

14 Planned Tables, Figures and Listing

Table 2 Planned tables, figures and listing for the statistical study report

Number	Title	Parameters
**1**	**Study Information**	
Figure 1	CONSORT 2010 flow diagram	Screened, eligible, enrolment; population divided up to the groups Part I and Part II
1.1	Observation of study entry	
1.1.1	Inclusion criteria	Inclusion criteria 1 – 8
1.1.2	Exclusion criteria	Exclusion criteria 1 – 16
1.2	Study termination	
1.2.1	Number of visits	Visits
1.2.2	Study duration per patient (distribution)	Time from visit 1 to visit 5
1.2.3	Follow-up visits per patient (distribution)	Time from visit 1 to visit 7
1.2.4	End of study or termination, drop out	Reason for end of study
**2**	**Descriptive Analysis** (for each parameter all visits)	
2.1	Demographic characteristics	Age, age in classes, gender, ethnicity
2.2	Anamnestic characteristics	BMI, BMI classes
2.3	Medical history	Chronic headache – other study relevant co-morbidities
2.4	Vital signs	Body temperature, heart rate, systolic and diastolic blood pressure, ECOG, body weight
2.5	Vital signs right after vaccination	Body temperature, heart rate, systolic and diastolic blood pressure, oxygen saturation<br>each after 30, 60, 90, 120 minutes
2.6	Laboratory assessments	Hematology,<br>Blood chemistry and coagulation,<br>Immunoglobulins/ immunophenotype,<br>Urine analysis
2.7	Signs and symptoms	Fever – diarrhea
2.8	Physical examination	
2.9	Vaccination	Dimetinden, location of vaccination
2.9.1	Investigation of vaccination site	Formation of granuloma – other suspicious findings at the injection site
2.9.2	Specific immune mediated diseases	Neuroinflammatory disorder – other immune mediated disease
2.9.3	Novel SARS-CoV-2 positivity/infection	Occurrence, accompanied by symptoms
2.10	Concomitant medication	Change since last assessment, new concomitant medication
2.11	T-cell response	Ex vivo ELISPOT<br>ELISPOT after 12-d IVS
2.12	Health conditions	Chronic headache – other study relevant co-morbidities
2.13	Adverse events/serious adverse events	CTCAE term, grading, SAE, relationship, outcome

Continued Table 2 Planned tables, figures and listing for the statistical study report

Number	Title	Parameters
**3**	**Statistical Analysis**	
3.1	Primary endpoint (documented within the visit eCRF)	Number of solicited and unsolicited ADRs or AEs,<br>Incidence of AESIs
3.2	Secondary endpoints	T-cell response: IFN-γ ELISpot, cellular conversion rate
3.3	Exploratory endpoints	Phenotyping<br>Cytokine profiles<br>Recognition rate<br>Intensity of T-cell response<br>Induction of long-term T-cell response<br>Induction of specific antibodies<br>Biomarkers<br>Clinical characteristics
**4**	**Safety Analysis**	
4.1	AEs	Max. severity CTC grading, duration, frequencies, related und unrelated, outcome
4.2	SAEs	Max. severity CTC grading, duration, frequencies, related und unrelated, outcome
**5**	**Individual Data Listing**	
5.1	Visit 0 (Screening and Enrolment)	
5.2	Visit 1 (Baseline-Vaccination)	
5.3	Visit 2	
5.4	Visit 3	
5.5	Visit 4	
5.6	Visit 5	
5.7	Visit 6	
5.8	Visit 7	
5.9	End of study (termination)	
5.10	T-cell response	
5.11	AEs/SAEs	
